# ST6GAL1 promotes cancer stem-like cell-associated paclitaxel resistance through the EGFR-mTOR-SOX2/BMI1 axis in non-small cell lung cancer

**DOI:** 10.20517/cdr.2026.66

**Published:** 2026-08-31

**Authors:** Yuxi Yang, Binghui Liang, Weijie Hong, Sau Har Lee, Dongfang Tang, Shuxian Xie, Changtai Qin, Liu Shen, Zhumei Sun, Xiaofeng Yan, Hua Li, Xiaoling Wang, Xudong Hu, Tingjie Ye, Wei Zhang, Wei Xu

**Affiliations:** ^1^School of Integrative Medicine, Shanghai University of Traditional Chinese Medicine, Shanghai 201203, China.; ^2^School of Biosciences, Faculty of Health and Medical Sciences, Taylor’s University, Subang Jaya 47500, Malaysia.; ^3^Department of Thoracic Surgery, Huadong Hospital affiliated with Fudan University, Shanghai 200040, China.; ^4^Department of Surgical Oncology, Shanghai Lung Cancer Center, Shanghai Chest Hospital, Shanghai Jiao Tong University School of Medicine, Shanghai 200030, China.; ^5^School of Biological Sciences, University of California San Diego, San Diego, CA 92093, USA.; ^6^Department of Clinical Laboratory, Peking University Shenzhen Hospital, Shenzhen 518036, Guangdong, China.; ^#^These authors contributed equally to this work.

**Keywords:** Non-small cell lung cancer, acquired paclitaxel resistance, cancer stem-like cell, stemness, N-glycosylation, ST6GAL1

## Abstract

**Aim:** Despite the contribution of cancer stem-like cells (CSLCs) to acquired paclitaxel resistance in non-small cell lung cancer (NSCLC), the biomarkers and regulatory mechanisms sustaining their stemness under chemotherapy pressure remain poorly understood. This study aimed to identify the stemness-maintaining programs underlying CSLC-associated paclitaxel resistance.

**Methods:** Paclitaxel-resistant NSCLC cell models were established. RNA-seq data from resistant spheres and adherent resistant cells were integrated with Gene Ontology/Kyoto Encyclopedia of Genes and Genomes/gene set enrichment analysis and patient transcriptomic datasets to identify CSLC maintenance-associated candidate biomarkers. Inhibitors, sphere-forming assays, CD104^-^CD166^+^CD49f^hi^ flow cytometry, reverse transcription quantitative polymerase chain reaction, western blotting, and ST6GAL1 knockdown or overexpression were used for functional and mechanistic validation. Sambucus nigra agglutinin lectin blotting was performed to assess epidermal growth factor receptor (EGFR) α2,6-sialylation. Clinical relevance was assessed using ST6GAL1 immunohistochemistry on 46 clinical lung tumor tissues.

**Results:** Paclitaxel-resistant NSCLC cells exhibited enhanced sphere formation and CD104^-^CD166^+^CD49f^hi^ expansion. N-glycosylation was activated in resistant spheres. A seven-gene N-glycosylation signature was identified as a CSLC-associated candidate biomarker in acquired paclitaxel resistance. Inhibiting N-glycosylation suppressed the EGFR-mTOR-SOX2/BMI1 axis, decreased CSLCs, and restored paclitaxel sensitivity. ST6GAL1 regulated EGFR α2,6-sialylation. ST6GAL1 depletion also decreased EGFR abundance, suppressed mTOR-SOX2/BMI1 signaling, and sensitized paclitaxel-resistant spheres to paclitaxel rather than adherent cells. ST6GAL1 expression, which was higher in tumors from patients who underwent chemotherapy, showed a trend toward poorer survival among chemotherapy-treated patients. The seven-gene signature was associated with shorter disease-free survival but not overall survival in lung cancer patients.

**Conclusions:** ST6GAL1-mediated α2,6-sialylation of EGFR contributes to the maintenance of CSLC-associated paclitaxel resistance through mTOR-SOX2/BMI1 signaling. These findings identify ST6GAL1-dependent EGFR sialylation as a potential therapeutic target in CSLC-associated chemoresistance.

## INTRODUCTION

Lung cancer is the leading cause of cancer mortality worldwide, and non-small cell lung cancer (NSCLC) accounts for most lung cancer cases^[1,2]^. Although paclitaxel-based chemotherapy remains an important therapeutic option for patients with advanced NSCLC, acquired resistance substantially limits its clinical benefits^[3,4]^. Therefore, understanding the mechanisms of acquired paclitaxel resistance is critical for developing more effective treatments.

Cancer stem cells play a central role in cancer initiation, therapeutic resistance, and recurrence because of their capacity for self-renewal and differentiation^[5,6]^. A CD104^-^CD166^+^CD49f^hi^ cancer stem-like cell (CSLC) subpopulation has been shown to possess stem-like properties and enhanced chemoresistance in lung cancer^[7]^. Serum-free sphere culture is widely used to enrich CSLCs, and sphere-forming ability (SFA) is considered a functional indicator of stemness^[7]^. Importantly, CSLCs are implicated in intrinsic chemoresistance^[8]^ and in acquired resistance during cancer treatment^[9]^, suggesting that the persistence of stem-like cells is a key cellular basis of NSCLC paclitaxel resistance and highlighting the need to define CSLC-maintaining molecular programs during chemotherapy.

Previous acquired chemoresistance studies have largely focused on molecular differences between resistant cells and their parental sensitive counterparts^[10,11]^, while investigations into lung CSLCs have mainly emphasized mechanisms of stemness maintenance^[12,13]^. However, the biological programs maintaining stem-like resistant subpopulations under chemotherapy pressure remain insufficiently defined. More importantly, studies integrating these research directions to identify factors that specifically maintain CSLC-like states in acquired drug-resistant tumor cells are rare. Therefore, rather than simply comparing bulk resistant and parental tumor cells, identifying stemness-maintaining markers in acquired resistant NSCLC may provide a more precise understanding of treatment failure. Here, we used RNA-seq to compare paclitaxel-resistant adherent cells and spheres to identify the pathways and regulatory markers that maintain stem-like properties in chemoresistant NSCLC.

N-glycosylation, a key post-translational modification of eukaryotic secretory and membrane-associated proteins^[14]^, involves the transfer of N-linked oligosaccharides to asparagine residues in nascent polypeptides^[15,16]^. Increasing evidence implicates aberrant N-glycosylation in cancer progression and chemoresistance^[17,18]^, including NSCLC chemoresistance^[19]^. ST6GAL1, a key glycosyltransferase, catalyzes the addition of α2,6-linked sialic acid to terminal galactose residues on N-glycan chains during terminal glycan remodeling. ST6GAL1 has been shown to promote chemoresistance in multiple cancers^[20,21]^ and to enhance stem-like phenotypes in colon and breast cancer^[22,23]^. However, it is unclear whether N-glycosylation, particularly ST6GAL1-mediated glycosylation, promotes acquired paclitaxel resistance by sustaining CSLC phenotypes in NSCLC. Its clinical relevance in chemotherapy-treated lung cancer also warrants further examination.

This study investigated the mechanisms that sustain stem-like properties in paclitaxel-resistant NSCLC. Based on a transcriptomic comparison of paclitaxel-resistant adherent cells and spheres, N-glycosylation was identified as being associated with CSLC enrichment in chemoresistant cells. We also examined the role of ST6GAL1 and its underlying mechanism in this process. Together, these findings provide mechanistic insight into glycosylation-dependent maintenance of stem-like resistant states and highlight ST6GAL1 as a potential target against paclitaxel-resistant NSCLC, which warrants further investigation.

## METHODS

### Drug preparation and storage

Paclitaxel (MCE, Shanghai, China), afatinib (MCE), rapamycin (RA; MCE), dorsomorphin (DO; MCE), and tunicamycin (TM; Selleck, Shanghai, China) were dissolved in dimethyl sulfoxide at stock concentrations of 10 mM, and aliquots were stored at -20 °C.

### Cell culture

A549 (RRID: CVCL_0023) and H1299 (RRID: CVCL_0060) cells were obtained from the American Type Culture Collection (VA, USA) and cultured in RPMI-1640 (Gibco, MA, USA) supplemented with 10% fetal bovine serum (GeminiBio, CA, USA) at 37 °C with 5% CO_2_ in a humidified incubator (Thermo Fisher Scientific, MA, USA). Paclitaxel-resistant cell lines were established through stepwise exposure to increasing concentrations of paclitaxel (12, 23, 59, and 117 nM) during an initial 28-day induction period, followed by continued selection with 117 nM paclitaxel for approximately three months. The cells were maintained at each concentration until tolerance was reached; if > 50% cell death occurred, paclitaxel was temporarily withdrawn to allow recovery. Cells exhibiting stable tolerance to 117 nM paclitaxel after selection for approximately four months were designated A549-TR and H1299-TR. Parental A549 and H1299 cells were derived from early-passage frozen stocks and used in experiments within 20 passages. Passage 1 resistant cell lines with confirmed paclitaxel resistance were maintained for stabilization and used within 15 passages after resistance was established. All human cell lines were authenticated via short tandem repeat (STR) profiling (Shanghai Fuheng Biotechnology Co., Ltd.) in November 2023. The STR profiles matched the reference profiles of the corresponding cell lines. Mycoplasma contamination was assessed using polymerase chain reaction (PCR) and monitored monthly.

### Establishment of knockdown and overexpression cells

The ST6GAL1 shRNA lentiviruses were purchased from GENEWIZ Company (Suzhou, China) with the following target sequences: *shST6GAL1-1* (CGCTGCTCTATGAGAAGAATT) and *shST6GAL1*-2 (CCTAAGCATGAACAAGTACAA). The ST6GAL1 overexpression plasmid was constructed with the ST6GAL1 coding sequence (CCDS3285.1) and packaged into lentiviral particles by GENEWIZ. The cells were digested with 0.25% trypsin (Gibco, USA) and resuspended at 2-5 × 10^4^/mL. Next, 2 mL of the cell suspension was seeded into six-well plates. After overnight incubation, 20 μL of lentivirus and 2 μL of polybrene were added to the wells. Infected cells were selected using 2 μg/mL puromycin per well after 2-3 days.

### Cell counting kit-8 assay

3,000 cells were seeded in 96-well plates and incubated overnight. The medium was then replaced with TM-, RA-, DO-, or paclitaxel-containing media, followed by incubation for 48 h. Next, 10 μL of Cell Counting Kit-8 reagent (Selleck, Shanghai, China) was added to each well, followed by incubation for 2-4 h. Absorbance was then measured at 450 nm on a microplate reader (Agilent, VT, USA).

### Sphere-forming assay

Cells were digested with 0.25% trypsin and resuspended in 1x phosphate-buffered saline (PBS) at a final density of 1 × 10^5^/mL. Next, 30 μL of cell suspension (3,000 cells per well) was seeded into Corning 24-well ultra-low-attachment plates and cultured in 500 μL of sphere-forming medium containing DMEM-F12 (Gibco), 1x B27 (Absin, Shanghai, China), 10 ng/mL epidermal growth factor (Absin), and 10 ng/mL basic fibroblast growth factor (Absin). These cells were then cultured at 37 °C for 5 days. In treatment assays, TM, RA, DO, and paclitaxel were added at the beginning of culture. At the fixed endpoint on day 5 after seeding, spheres with a diameter of > 50 μm were imaged and quantified using ImageJ. SFA was assessed by sphere count. The reported n values represent independent biological replicates.

### Quantitative reverse transcription polymerase chain reaction

Total RNA was extracted using TRIzol reagent (Sigma, MO, USA) and reverse-transcribed using a StarScript II first-strand cDNA synthesis kit (Yeason, Shanghai, China) following the manufacturer’s instructions. Reverse transcription quantitative polymerase chain reaction (RT-qPCR) was performed using ChamQ Universal SYBR qPCR Master Mix (Vazyme, Nanjing, China) with GAPDH as the reference gene. The RT-qPCR primer sequences are presented in Supplementary File 1.

### Immunoprecipitation

Cells were washed with ice-cold phosphate-buffered saline and lysed in immunoprecipitation buffer [50 mM Tris-HCl (pH 7.4; Sangon Biotech, Shanghai, China), 150 mM NaCl (Sangon Biotech), 1% Triton X-100 (Sinopharm Chemical Reagent Co., Ltd., Beijing, China), and 1 mM EDTA (Sinopharm Chemical Reagent Co., Ltd.)], supplemented with protease inhibitors (Selleck). Lysates were incubated on ice for 45 min with gentle mixing, then centrifuged at 14,000 rpm (4 °C) for 10 min. The supernatants were collected, and protein concentrations were measured using a bicinchoninic acid assay (Beyotime, Shanghai, China). Equal amounts of protein were adjusted to the same volume with lysis buffer. Lysates were then precleared with 10 μL of protein A/G agarose beads (PR40025; Proteintech, Wuhan, China) for 30 min at 4 °C with rotation. After centrifugation, 100 μL of each supernatant was retained as input. 1 mg of each lysate was incubated overnight with 2 μg of anti-epidermal growth factor receptor (EGFR) antibody at 4 °C. Species-matched IgG (98136-1-RR; Proteintech) was used as the negative control. Washed protein A/G agarose beads were then added to the samples, followed by incubation for 4 h at 4 °C with rotation. The beads were collected via centrifugation, washed three times with ice-cold immunoprecipitation buffer, and resuspended in protein loading buffer. Proteins were eluted by heating, followed by Western blot analysis. Sambucus nigra agglutinin (SNA) lectin blotting was used to assess α2,6-linked sialylation of immunoprecipitated EGFR. Briefly, the membranes were incubated with biotinylated SNA (B-1305-2; Vector Laboratories, CA, USA) at 4 μg/mL, followed by incubation with Streptavidin-HRP Conjugate (HRP-PF00030; Proteintech) for signal detection. Band intensities were quantified using Lane 1D software (Beijing Sage Creation Science Co., Ltd., Beijing, China), and the SNA signal was normalized to the corresponding immunoprecipitated EGFR signal.

### Western blot

Total protein was extracted using a lysis buffer (X-Blot, Suzhou, China). The lysates were subsequently centrifuged at 14,000 rpm for 10 min at 4 °C. Proteins were then denatured, resolved using 10% sodium dodecyl sulfate-polyacrylamide gel electrophoresis, and transferred onto polyvinylidene difluoride membranes. Membranes were incubated overnight at 4 °C with primary antibodies, followed by incubation with horseradish peroxidase (HRP)-conjugated secondary antibodies for one hour at room temperature. Signal was developed using an enhanced chemiluminescence solution (X-blot), and bands were imaged on a Tanon Chemi Dog 5200T system (Tanon, Shanghai, China). Band intensities were quantified using Lane 1D software (SAGE, Beijing, China). Relative protein expression was determined by normalizing target signal intensities to GAPDH, β-actin, and β-tubulin. Because total mechanistic target of rapamycin (mTOR) and AMP-activated protein kinase (AMPK) levels varied in some assays, phosphorylated mTOR and AMPK were normalized to the corresponding loading controls, as described in previous studies^[24,25]^. The antibodies used in this study are provided in Supplementary File 2.

### Flow cytometric analysis

Cells were dissociated into single-cell suspensions, resuspended in 1X PBS containing 2% fetal bovine serum, and 200 μL of the cell suspension was incubated with 4 μL of CD104-FITC (BioLegend, CA, USA), CD166-PE (BioLegend), or CD49f-APC (BioLegend) for 15 min at 4 °C in the dark. After centrifugation at 350 × *g* at 4 °C for 5 min, the cells were suspended in PBS, transferred to a flow cytometry tube, and analyzed on a CytoFLEX LX flow cytometer (Beckman Coulter, Germany). Gating analysis was conducted with FlowJo (BD, OR, USA).

### RNA-seq and differential gene analysis

Total RNA was extracted using TRIzol reagent (Sigma), and sequencing was outsourced to GENEWIZ. RNA sequencing was performed using one independent biological sample per group, comprising adherent A549-TR cells and A549-TR spheres. Differential gene expression analysis between adherent A549-TR cells and A549-TR spheres was conducted using the R package edgeR. Genes with counts per million > 2 in at least one sample were retained for differential expression analysis. Differentially expressed genes were defined as those with |log2 fold change| ≥ 1 and a Benjamini–Hochberg-adjusted false discovery rate (FDR) < 0.05 and were visualized on volcano plots (the complete gene list is provided in Supplementary File 3). Gene Ontology biological process (GO-BP), molecular function (GO-MF), and Kyoto Encyclopedia of Genes and Genomes (KEGG) enrichment analyses were used to characterize the functional and pathway-level features of the differentially expressed genes, with the results provided in Supplementary File 3. Heatmap visualization and gene set enrichment analysis (GSEA) were conducted in RStudio.

### Gene set variation analysis and correlation analysis

RNA-seq data from 870 lung cancer patient samples were retrieved from The Cancer Genome Atlas (TCGA) database via the Genomic Data Commons Data Portal. The quantitative protein levels in lung cancer patients were downloaded from the cBio Cancer Genomics Portal^[26]^, while the protein levels in various cancer cell lines were obtained from the DepMap portal^[27]^. RNA-seq data and normalized protein expression data were processed in RStudio. Signaling pathways and N-glycosylation indices derived from the 19 upregulated N-glycosylation-related genes were analyzed using the gene set variation analysis (GSVA) package, as shown in Supplementary File 4. Correlation analysis was performed using the Pearson method.

### Immunohistochemistry

A total of 46 clinical lung tumor tissues were stained for ST6GAL1. The use of these clinical samples was approved by the Institutional Ethics Committee of Huadong Hospital (approval No. 20180046), and written informed consent was obtained from all patients. Antigen retrieval was conducted using the microwave-based heat-induced epitope retrieval method. Tissue microarrays were incubated overnight with primary antibodies against ST6GAL1 (Proteintech) at 4 °C, followed by incubation with HRP-conjugated secondary antibodies at 37 °C for 1 h. Signal was developed using HRP substrate 3,3′-diaminobenzidine for 2-5 min, followed by counterstaining with hematoxylin and mounting. Slides were digitized using a high-resolution whole-slide scanner (NanoZoomer series; Hamamatsu, Japan). Staining intensity was quantified based on the mean grayscale value using ImageJ and then converted into optical density values using the following formula: optical density 
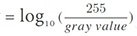
. The ST6GAL1 staining intensity and corresponding cancer tissue clinical information are presented in Supplementary File 5.

### Survival curve analysis

Overall survival (OS) and disease-free survival (DFS) analyses of genes of interest and the seven-gene glycosylation signature were performed using GEPIA2. The “Signatures” option in GEPIA2 was selected for signature analysis, and *ALG1*, *HEXA*, *MAN1C1*, *MGAT5*, *MOGS*, *ST6GAL1*, and *MGAT4A* were entered as Gene Set A. The combined signature score was generated using GEPIA2’s signature module. Quartile-based expression cut-offs were applied, and hazard ratios (HRs) were estimated within the lung adenocarcinoma and lung squamous cell carcinoma datasets. Patients with signature scores above the 75th percentile were classified into the high-signature group, while those with scores below the 25th percentile were classified into the low-signature group. Patients with intermediate scores were not included in the high *vs.* low survival comparison. HRs and 95% confidence intervals (CIs) were calculated using the Cox proportional hazards model.

For the survival analysis of chemotherapy-treated patients, 14 patients were included and stratified into high- and low-ST6GAL1 expression groups based on the median ST6GAL1 OD value of the 46 tumor samples (high, *n* = 8; low, *n* = 6; Supplementary File 5). Patients received different chemotherapy regimens according to standard clinical treatment protocols, and the specific regimens are provided in Supplementary File 5. OS was defined as the time from surgery to death or last follow-up. Kaplan-Meier analysis was performed in R (version 4.5.2) using RStudio (version 2025.9.2.418), and survival differences between groups were assessed using the log-rank test. HRs and 95%CIs were estimated using Cox proportional hazards regression.

### Effect-size estimation and forest plot analysis

Gene-specific HRs, *p*(HRs), and 95%CIs for DFS were obtained from GEPIA2 and presented as forest plots generated in STATA version 16.0 (STATA Corporation, TX, USA). An HR > 1 indicated an association between higher gene expression and shorter DFS. For RT-qPCR data, gene-specific standardized mean differences (SMDs) and corresponding 95%CIs were calculated to quantify changes in the expression of stemness-associated genes. An SMD > 0 indicated increased expression relative to the reference group, whereas an SMD < 0 indicated decreased expression.

### Statistical analysis

Data are presented as the mean ± standard error of the mean (SEM) from at least three independent replicates. Differences between two groups were compared using a two-sided Student’s *t*-test. Differences between multiple groups were compared using one-way analysis of variance (ANOVA), followed by Dunnett’s multiple comparisons. A *P* value < 0.05 indicated statistically significant differences. Error bars indicate mean ± SEM. n.s., *, and ** indicate *P* > 0.05, < 0.05, and < 0.01, respectively.

## RESULTS

### Acquired paclitaxel-resistant cells showed enrichment of CSLC populations

**Figure 1:**
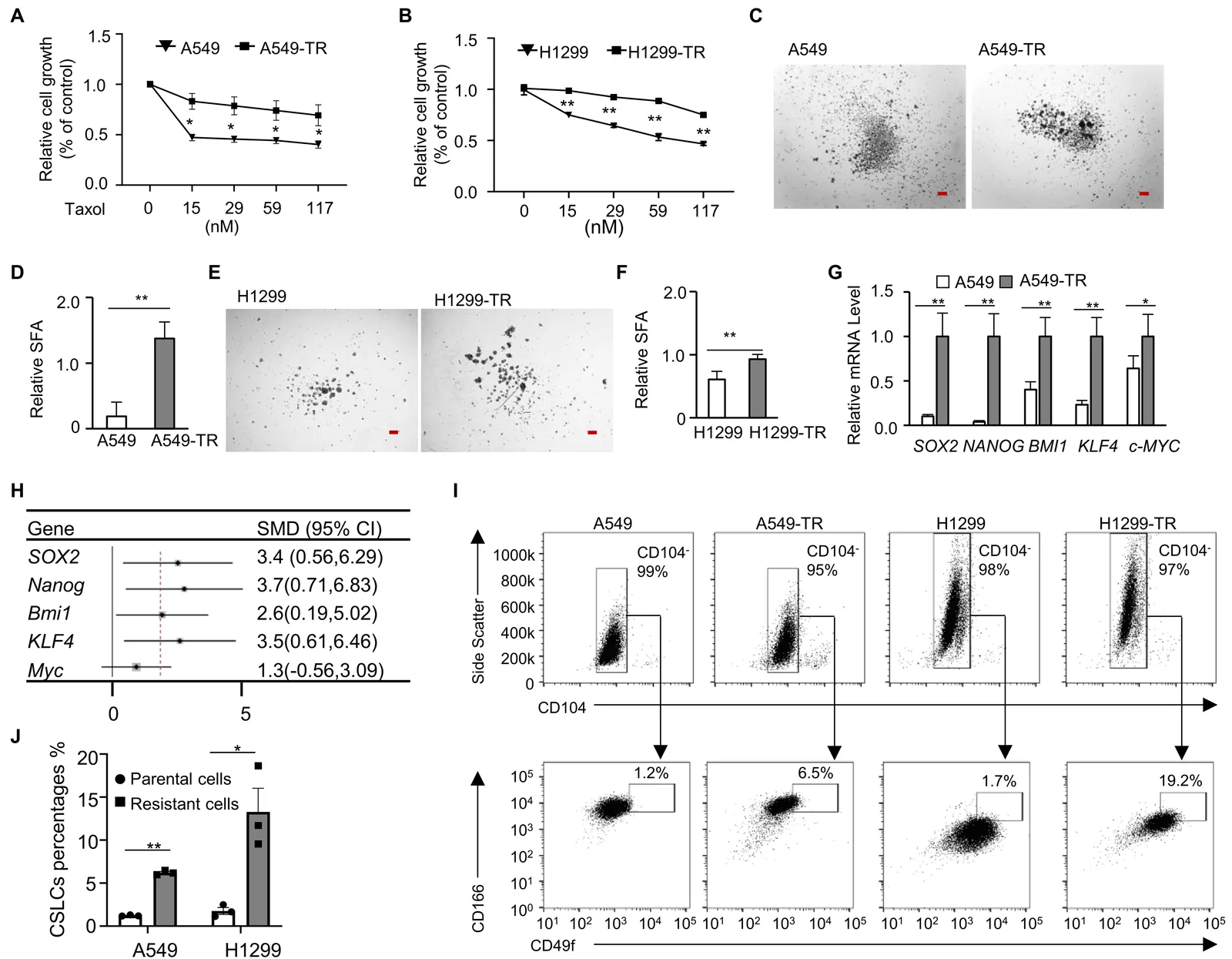
Acquired paclitaxel-resistant cells showed enrichment of CSLC populations. (A and B) Relative cell growth of A549-TR (A) and H1299-TR (B) cells treated with increasing concentrations of paclitaxel (0-117 nM) for 48 h; parental cells were the controls; *n* = 3; (C-F) Representative images (C and E) and quantification (D and F) of the relative SFA of parental and resistant cells following 117 nM paclitaxel treatment. Scale bar, 100 μm; *n* = 4; (G) RT-qPCR analysis of stemness-associated gene expression in A549-TR spheres *vs.* A549 spheres. *n* = 3; (H) Forest plot showing the gene-specific SMDs of stemness gene expression levels in resistant spheres *vs.* parental spheres; (I and J) Representative gating plots (I) and quantification (J) of CD104^-^CD166^+^CD49f^hi^ CSLC subpopulations in parental cells and resistant cells; *n* = 3. ^*^P < 0.05; ^**^P < 0.01. CSLC: Cancer stem-like cell; SFA: sphere-forming ability; RT-qPCR: reverse transcription quantitative polymerase chain reaction; SMDs: standardized mean differences; CI: confidence interval.

Paclitaxel-resistant NSCLC cell lines, A549-TR and H1299-TR, were established to investigate the molecular mechanism of acquired chemoresistance. The relative cell growth of the parental cells decreased in a dose-dependent manner upon exposure to paclitaxel, while resistant cells exhibited significantly less growth inhibition under the same treatment conditions (*P* < 0.05; Figure 1A and B), confirming the successful establishment of paclitaxel resistance. Notably, spheres derived from paclitaxel-resistant cells exhibited markedly higher chemoresistance compared with those derived from parental controls, with increased SFAs during paclitaxel treatment [Figure 1C-F]. The expression of stemness-associated transcription factors, including *SOX2*, *NANOG*, *BMI1*, *KLF4*, and *c-MYC*, was evaluated because of their established roles in cancer stemness^[28-30]^. RT-qPCR analysis showed that the five genes were significantly more highly expressed in resistant spheres, and the consistently positive gene-specific effect estimates further supported this upregulation trend [Figure 1G and H]. Flow cytometric analysis revealed a marked increase in the stem-like CD104^-^CD166^+^CD49f^hi^ subpopulation in resistant cells [Figure 1I and J]. Collectively, these results indicate that acquired paclitaxel resistance is characterized by an enrichment of CSLC subpopulations.

### The N-glycosylation process was upregulated in paclitaxel-resistant spheres

**Figure 2:**
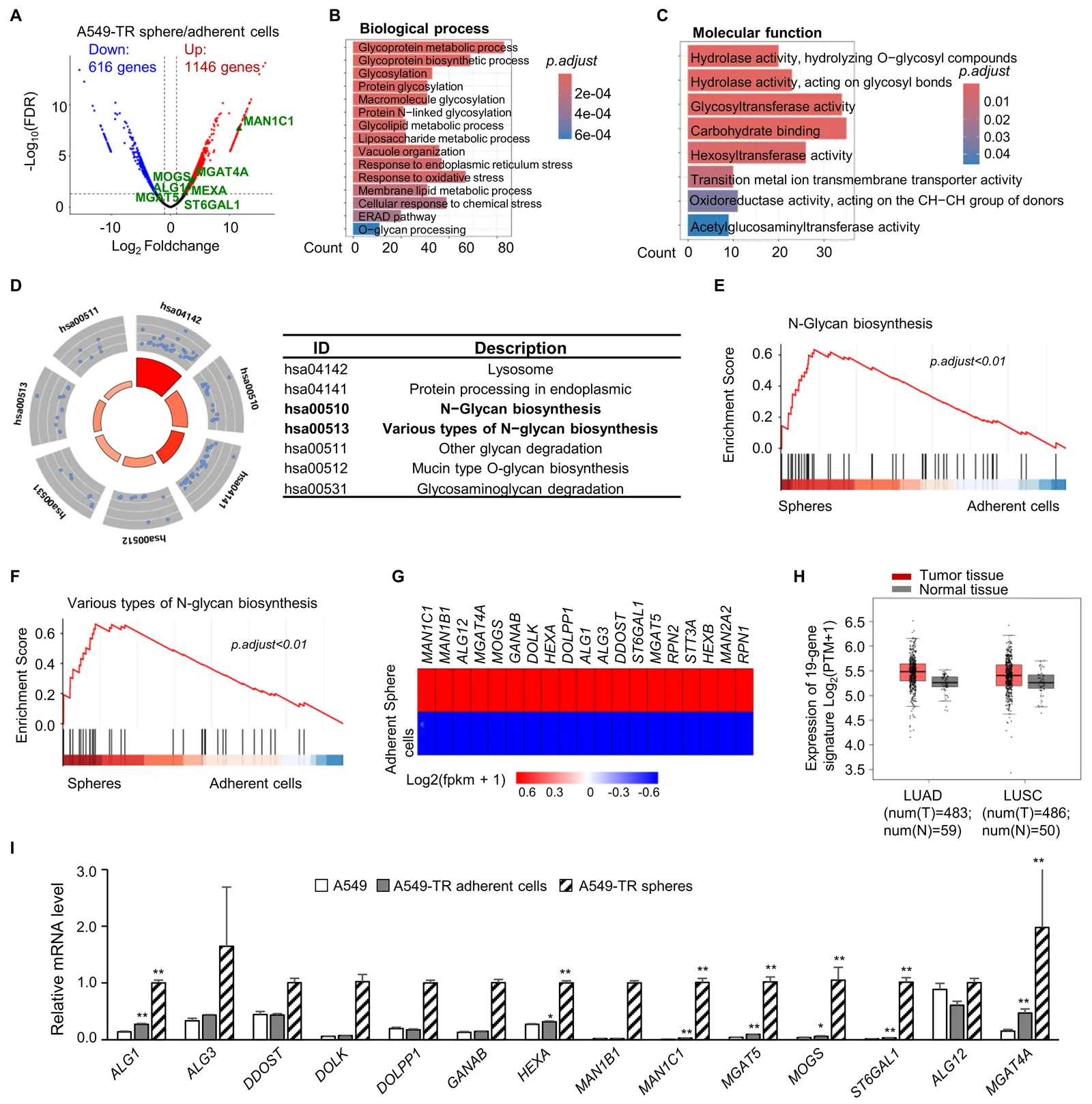
N-glycosylation was upregulated in the paclitaxel-resistant spheres. (A) Volcano plot showing differentially expressed genes in A549-TR spheres *vs.* adherent cells. Genes with a |log_2_-fold change| > 1 and FDR < 0.05 were considered significant. The blue and red dots represent down- and upregulated genes, respectively. The selected genes are labeled in green; (B and C) GO enrichment analysis of upregulated genes showing enriched terms in two categories: biological process (B) and molecular function (C). The color intensity represents the statistical significance of each term; (D) Circular plot for KEGG enrichment of the upregulated genes; (E and F) GSEA of N-glycan biosynthesis (E) and various types of N-glycan biosynthesis (F) in spheres *vs.* adherent cells. Adjusted *P* < 0.01; (G) Heatmap of FPKM values for 19 differentially expressed genes involved in N-glycan biosynthesis and various types of N-glycan biosynthesis; (H) Expression of a 19-gene upregulated N-glycosylation signature in tumor and normal tissues from the TCGA-LUAD and TCGA-LUSC cohorts; (I) RT-qPCR validation of the top 14 upregulated N-glycosylation-related genes in A549-TR adherent cells and spheres compared with parental cells, *n* = 4. ^*^P < 0.05; ^**^P < 0.01. FDR: False discovery rate; GO: Gene Ontology; KEGG: Kyoto Encyclopedia of Genes and Genomes; GSEA: gene set enrichment analysis; FPKM: fragments per kilobase of transcript per million mapped reads; TCGA: The Cancer Genome Atlas; LUAD: lung adenocarcinoma; LUSC: lung squamous cell carcinoma; RT-qPCR: reverse transcription quantitative polymerase chain reaction.

Consequently, RNA-seq analysis of A549-TR adherent cells and spheres was conducted to elucidate the molecular mechanisms underlying CSLC enrichment in paclitaxel-resistant NSCLC. Transcriptomic profiling revealed extensive gene expression changes in resistant spheres [Figure 2A]. Gene Ontology highlighted glycosylation-related pathways, including glycoprotein metabolism, protein glycosylation, and glycosyltransferase activity [Figure 2B and C]. KEGG and GSEA also revealed enrichment of N-glycan biosynthesis pathways, including those involving hsa00510 and hsa00513 [Figure 2D-F]. Hierarchical clustering analysis identified 19 N-glycosylation-associated differentially expressed genes in resistant spheres, all of which were upregulated [Figure 2G]. The upregulated 19-gene N-glycosylation signature was also elevated in lung tumor tissues compared with normal tissues [Figure 2H]. RT-qPCR validation analysis of the top 14 upregulated N-glycosylation-related genes based on RNA-seq found that *ALG1*, *HEXA*, *MAN1C1*, *MGAT5*, *MOGS*, *ST6GAL1*, and *MGAT4A* were consistently upregulated in A549-TR cells compared with parental cells, with further increases in A549-TR spheres [Figure 2I]. These results revealed a CSLC maintenance-associated seven-gene N-glycosylation signature in chemoresistant NSCLC.

### Inhibition of N-glycosylation reduced CSLCs and restored paclitaxel sensitivity of resistant cells and spheres

**Figure 3:**
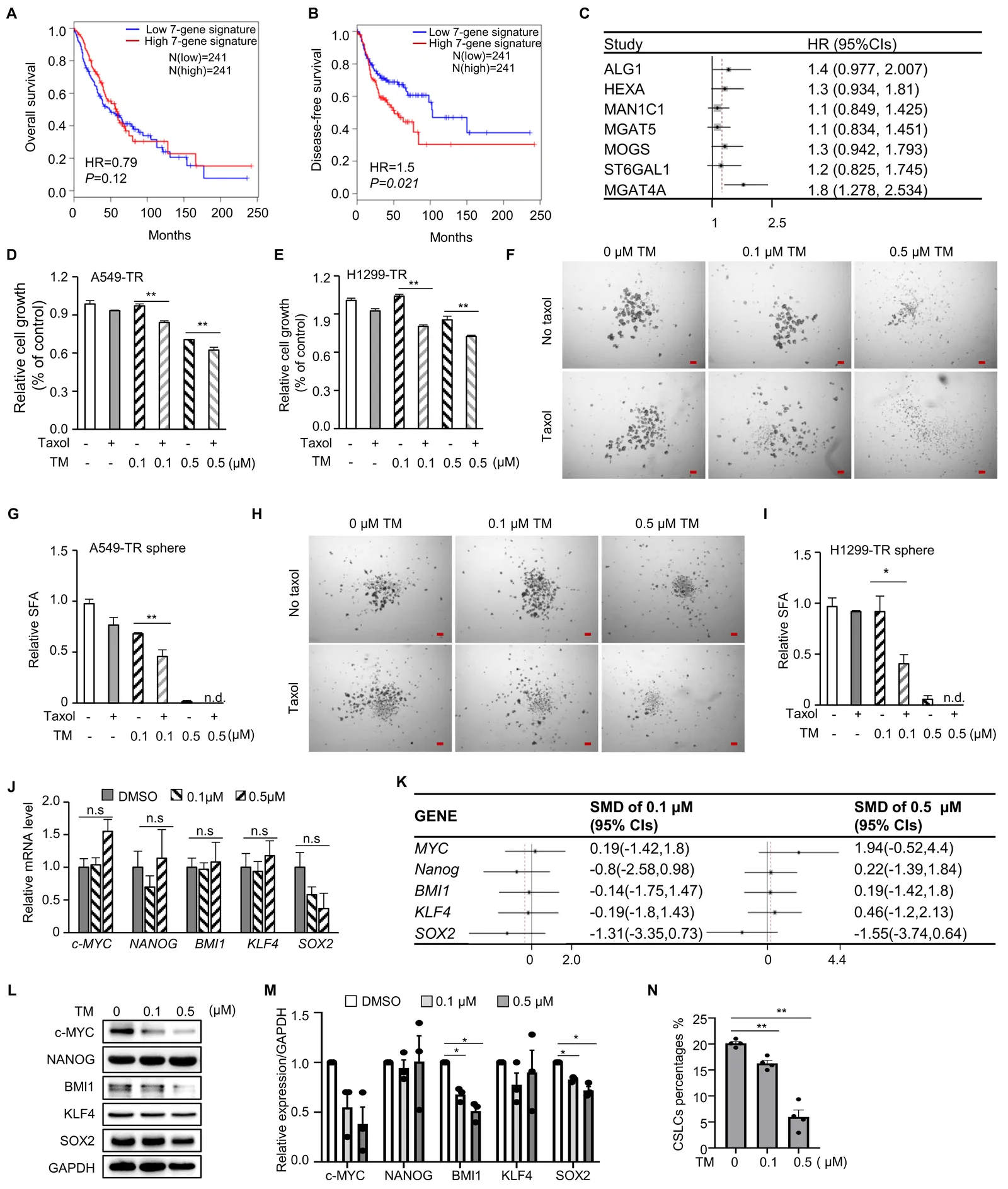
Inhibition of N-glycosylation reduced CSLC-associated phenotypes and restored paclitaxel sensitivity. (A and B) Kaplan-Meier curves showing the OS (A) and DFS (B) in lung cancer patients with high *vs.* low expression of a seven-gene N-glycosylation signature (*n* = 241 per group); (C) Forest plot showing the gene-specific HRs for DFS based on the expression of the seven N-glycosylation-related genes; (D and E) Relative cell growth of A549-TR (D) and H1299-TR (E) cells following 48 h of treatment with 117 nM paclitaxel alone or in combination with TM; *n* = 3; (F-I) Representative images and quantification of sphere formation in A549-TR (F and G) and H1299-TR (H and I) cells following combined paclitaxel and TM treatment; Scale bar, 100 μm; *n* = 3; (J) RT-qPCR analysis of stemness-associated genes in A549-TR spheres treated with TM (0.1 or 0.5 μM), *n* = 3; (K) Forest plot showing gene-specific SMDs of stemness-associated gene expression following TM treatment; (L and M) Representative western blots (L) and quantification (M) of stemness-associated proteins in A549-TR cells treated with TM (0.1 or 0.5 μM) for 48 h. Data were obtained from three independent biological replicates (*n* = 3); (N) Quantification of CD104^-^CD166^+^CD49f^hi^ CSLC subpopulations in H1299-TR cells after 48 h of TM treatment, *n* = 3. n.s., not significant; ^*^P < 0.05; ^**^P < 0.01. CSLC: Cancer stem-like cells; OS: overall survival; DFS: disease-free survival; HRs: hazard ratios; TM: tunicamycin; RT-qPCR: reverse transcription quantitative polymerase chain reaction; SMDs: standardized mean differences; CIs: confidence intervals; n.d.: not detected; SFA: sphere-forming ability; DMSO: dimethyl sulfoxide.

Next, we explored the functional relevance of the seven N-glycosylation-related genes in paclitaxel resistance. Kaplan-Meier analysis revealed that the seven-gene signature was significantly associated with shorter DFS, but not with OS [Figure 3A and B]. The consistently higher gene-specific HR estimates for DFS further supported the association of these genes with poorer DFS [Figure 3C and Supplementary Figure 1]. To assess the functional contribution of N-glycosylation to resistance, we treated parental and paclitaxel-resistant cells with TM, a selective N-glycosylation inhibitor. At 0.1 μM, TM reduced relative cell growth by > 10% in parental cells (*P* < 0.01) but had a minimal effect (< 2%) on paclitaxel-resistant cells [Supplementary Figure 2A and B]. Notably, at 0.1 μM, TM significantly restored paclitaxel sensitivity in both A549-TR and H1299-TR cells (*P* < 0.01), which was enhanced at 0.5 μM TM, indicating a dose-dependent chemoresistance reversal [Figure 3D and E]. These findings indicated that N-glycosylation contributed functionally to chemoresistance in NSCLC.

Analysis of the effects of inhibiting N-glycosylation in spheres revealed that cotreatment with paclitaxel and 0.1 μM TM significantly restored the paclitaxel sensitivity of resistant spheres, whereas 0.5 μM TM alone nearly abolished SFA [Figure 3F-I]. Mechanistic analysis revealed that TM treatment did not significantly alter the mRNA levels of stemness-associated genes, consistent with the gene-specific effect estimates at both 0.1 and 0.5 μM TM [Figure 3J and K]. In contrast, the protein levels of SOX2, c-MYC, and BMI1 were markedly reduced following TM exposure (*P* < 0.05; Figure 3L and M). Flow cytometric analysis revealed a significant reduction in the CD104^-^CD166^+^CD49f^hi^ subpopulation in TM-treated H1299-TR cells [Figure 3N and Supplementary Figure 2C]. These findings indicated that inhibiting N-glycosylation downregulated stemness-associated proteins and reduced CSLC populations, thereby restoring paclitaxel sensitivity in NSCLC cells.

### mTOR and AMPK signaling were modulated by N-glycosylation inhibition in paclitaxel-resistant lung cancer cells

**Figure 4:**
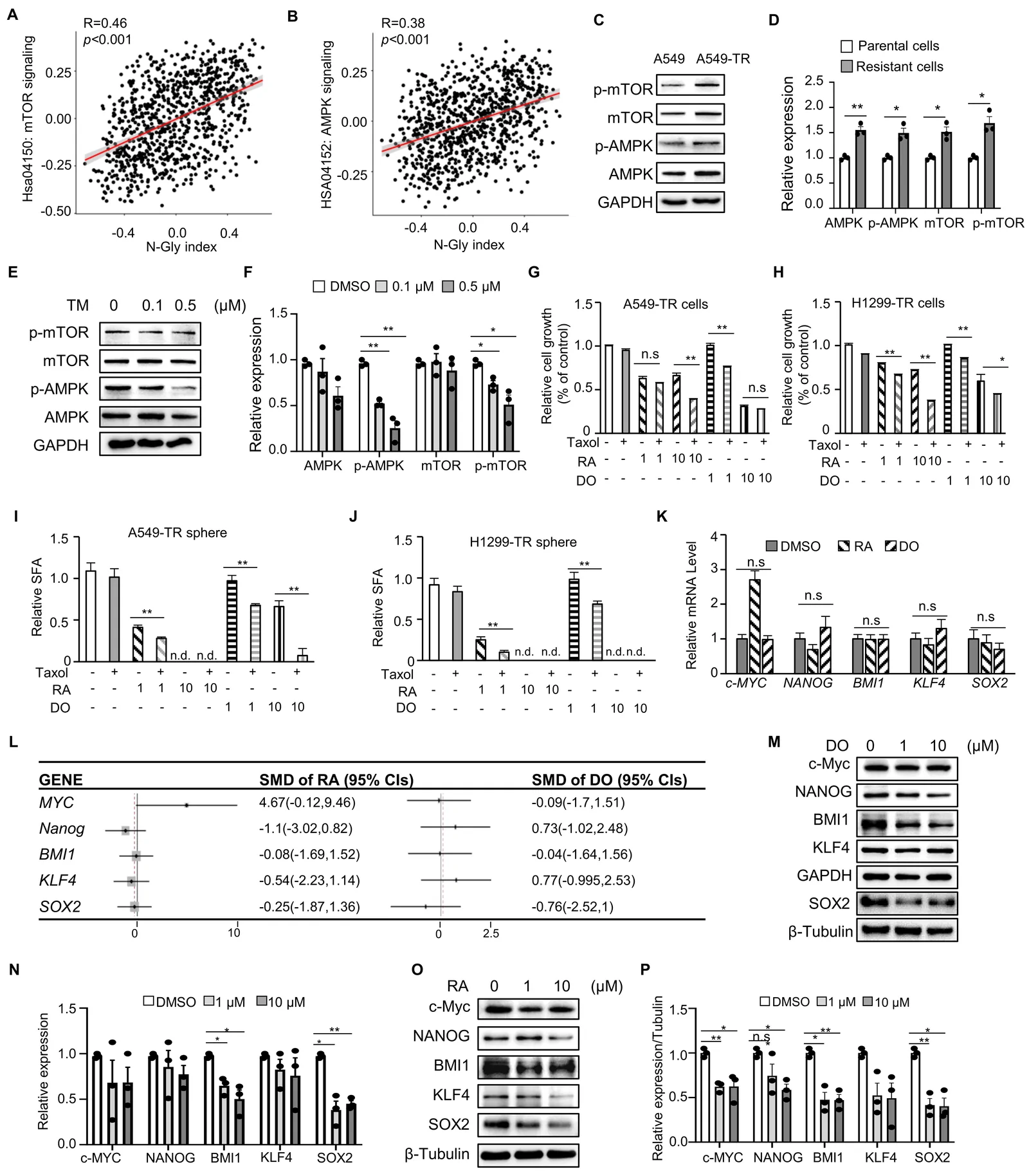
mTOR and AMPK signaling were modulated by N-glycosylation inhibition in paclitaxel-resistant lung cancer cells. (A and B) Scatter plots showing the correlation between the N-glycosylation index and selected oncogenic signaling pathways, including the mTOR (A) and AMPK (B) pathways; (C and D) Representative western blots (C) and quantification (D) of p-mTOR and p-AMPK levels in A549 and A549-TR cells, *n* = 3; (E and F) Representative western blots (E) and quantification (F) of p-mTOR and p-AMPK in A549-TR cells treated with TM (0.1 or 0.5 μM) for 48 h, *n* = 3; (G and H) Relative cell growth of A549-TR (G) and H1299-TR (H) cells following treatment with RA or DO alone or in combination with 117 nM paclitaxel for 48 h, *n* = 3; (I and J) SFAs of A549-TR (I) and H1299-TR (J) cells after treatment with RA or DO alone or in combination with paclitaxel at the indicated concentrations, *n* = 4; (K and L) RT-qPCR analysis (K) and forest plot of gene-specific SMDs (L) of the expression of stemness-associated genes in A549-TR spheres treated with 1 µM RA or DO for 48 h; *n* = 3; (M and N) Representative western blots (M) and quantification (N) of stemness-associated proteins in A549-TR cells treated with 1 or 10 µM DO for 48 h. Statistical data were from three independent biological replicates (*n* = 3); (O and P) Representative western blots (O) and quantification (P) of stemness-associated proteins in A549-TR cells treated with 1 or 10 µM RA for 48 h. Statistical data were from three independent biological replicates (*n* = 3). n.s., not significant; ^*^P < 0.05; ^**^P < 0.01. mTOR: Mechanistic target of rapamycin; AMPK: AMP-activated protein kinase; TM: tunicamycin; RA: rapamycin; DO: dorsomorphin; SFAs: sphere-forming abilities; RT-qPCR: reverse transcription quantitative polymerase chain reaction; SMDs: standardized mean differences; DMSO: dimethyl sulfoxide; n.d.: not detected; CIs: confidence intervals.

To investigate intracellular signaling pathways potentially involved in N-glycosylation-related signaling, transcriptomic correlation analysis was performed using TCGA lung cancer datasets. N-glycosylation signaling was positively correlated with several oncogenic signaling pathways [Supplementary Figure 3A]. Pearson correlation analysis revealed that N-glycosylation was positively associated with mTOR (R = 0.46) and AMPK (R = 0.38) [Figure 4A and B]. N-glycosylation was also positively associated with the Notch, sphingolipid, HIF, ErbB, VEGF, and Foxa pathways, although the association was weaker [Supplementary Figure 3B-G]. Moreover, the levels of phospho-mTOR and phospho-AMPK in paclitaxel-resistant cells were elevated by approximately 50% higher than those in parental cells [Figure 4C and D, Supplementary Figure 3H and I] and significantly lower following TM treatment [Figure 4E and F]. These findings indicated that mTOR and AMPK signaling may be downstream of the N-glycosylation process in acquired paclitaxel chemoresistance.

Paclitaxel-resistant cells were treated with RA (mTOR inhibitor) or DO (AMPK inhibitor) alone or in combination with paclitaxel to evaluate the function of mTOR and AMPK signaling in paclitaxel resistance. RA reduced relative cell growth of A549-TR and H1299-TR cells, and only 10 μM RA significantly enhanced paclitaxel-induced growth inhibition, resulting in an additional reduction of approximately 20% relative to paclitaxel treatment alone [Figure 4G and H]. In contrast, 1 µM DO alone had no inhibitory effect, but it significantly restored paclitaxel sensitivity in resistant cells [Figure 4G and H]. In sphere assays, treatment with RA at 1 µM significantly impaired sphere formation and restored paclitaxel sensitivity in resistant spheres, enhancing SFA reduction by 15% [Figure 4I and J, Supplementary Figure 4A and B]. In contrast, treatment with DO at 1 µM selectively restored paclitaxel sensitivity, with a 25% decrease in SFA, without inhibiting SFA alone [Figure 4I and J, Supplementary Figure 4A and B]. We assessed the expression of stemness-associated genes to determine whether these effects involved CSLC-associated mechanisms. RT-qPCR analysis revealed no significant changes in mRNA levels after RA or DO treatment, consistent with the gene-specific effect estimates [Figure 4K and L]. However, Western blot analysis revealed that DO significantly reduced the levels of BMI1 and SOX2 [Figure 4M and N], whereas RA suppressed the expression of stemness markers, including c-MYC, BMI1, and SOX2 [Figure 4O and P]. These findings suggested that inhibition of mTOR or AMPK restored the sensitivity of paclitaxel-resistant spheres to paclitaxel by downregulating key stemness-associated proteins such as SOX2 and BMI1.

### EGFR acted upstream of mTOR signaling in N-glycosylation-dependent CSLC-associated paclitaxel resistance

**Figure 5:**
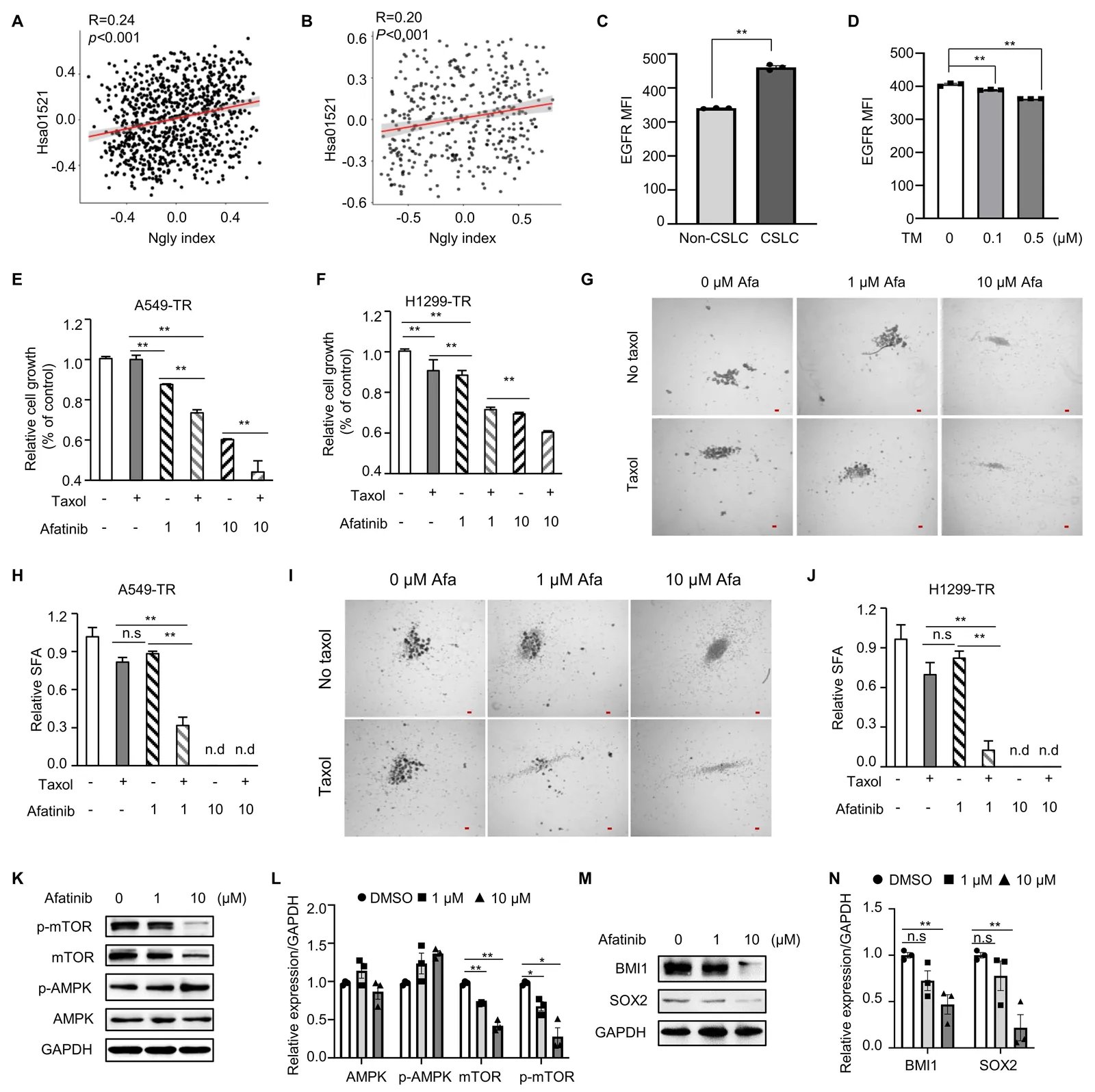
EGFR acted upstream of mTOR signaling in N-glycosylation-dependent CSLC-associated paclitaxel resistance. (A) Pearson correlation analysis of the N-glycosylation index with hsa01521 (EGFR tyrosine kinase inhibitor resistance) using RNA-seq data from 870 lung cancer samples; (B) Pearson correlation analysis of the N-glycosylation index with hsa01521 using TMT-quantified protein expression data from 378 cancer cell lines; (C) MFI of EGFR expression in CD104^-^CD166^+^CD49f^hi^ CSLCs and non-CSLCs from A549-TR cells analyzed by flow cytometry; *n* = 3; (D) MFI of EGFR expression in A549-TR cells treated with TM, *n* = 3; (E and F) Relative cell growth of A549-TR (E) and H1299-TR (F) cells treated with paclitaxel in combination with afatinib, *n* = 4; (G-J) Representative images and quantification of spheres in A549-TR (G and H) and H1299-TR (I and J) cells treated with paclitaxel in combination with afatinib; scale bar, 100 µm; *n* = 4; (K and L) Representative western blots (K) and quantification (L) of p-mTOR and p-AMPK in A549-TR cells treated with 1 or 10 µM afatinib for 48 h. Statistical data were from three independent biological replicates (*n* = 3); (M and N) Representative western blots (M) and quantification (N) of BMI1 and SOX2 in A549-TR cells treated with 1 or 10 µM afatinib for 48 h. Statistical data were from three independent biological replicates (*n* = 3). n.s., not significant; ^*^P < 0.05; ^**^P < 0.01. EGFR: Epidermal growth factor receptor; mTOR: mechanistic target of rapamycin; CSLC: cancer stem-like cell; TMT: tandem mass tag; MFI: mean fluorescence intensity; TM: tunicamycin; Afa: afatinib; n.d.: not detected; AMPK: AMP-activated protein kinase; DMSO: dimethyl sulfoxide.

Several receptors implicated in cancer progression undergo glycosylation^[31,32]^, including EGFR, which is extensively N-glycosylated on the extracellular domain^[33]^. Our analysis revealed that EGFR tyrosine kinase inhibitor resistance signaling (hsa01521) was enriched in spheres [Supplementary Figure 5A]. Moreover, N-glycosylation was positively associated with EGFR-related signaling at the transcriptomic and proteomic levels [Figure 5A and B], suggesting that EGFR signaling is involved in paclitaxel resistance. To explore whether EGFR is involved in paclitaxel resistance, EGFR expression was analyzed in CSLCs from paclitaxel-resistant cells. EGFR levels were higher in the CSLC subpopulation than in the non-CSLC subpopulation of paclitaxel-resistant cells [Figure 5C and Supplementary Figure 5B]. Notably, inhibiting N-glycosylation using TM significantly reduced EGFR expression in A549-TR cells, suggesting that N-glycosylation regulates EGFR expression in paclitaxel-resistant cells [Figure 5D and Supplementary Figure 5C]. The role of EGFR in paclitaxel resistance was further assessed by pharmacologically inhibiting EGFR using afatinib, which markedly reversed paclitaxel resistance in adherent cells [Figure 5E and F] and resensitized paclitaxel-resistant spheres to paclitaxel [Figure 5G-J]. However, at 1 μM, afatinib alone did not significantly inhibit spheres [Figure 5G-J]. Subsequent EGFR inhibition significantly decreased phospho-mTOR levels but did not significantly affect phospho-AMPK levels [Figure 5K and L], indicating that mTOR signaling rather than AMPK signaling was downstream of EGFR, which was consistent with the correlation analysis [Supplementary Figure 5D]. Furthermore, 10 μM afatinib significantly downregulated BMI1 and SOX2 [Figure 5M and N]. These findings indicated that N-glycosylation inhibition restored paclitaxel sensitivity by modulating the EGFR-mTOR-SOX2/BMI1 axis.

### ST6GAL1 sustained CSLC-associated paclitaxel resistance through the EGFR-mTOR-SOX2/BMI1 axis

**Figure 6:**
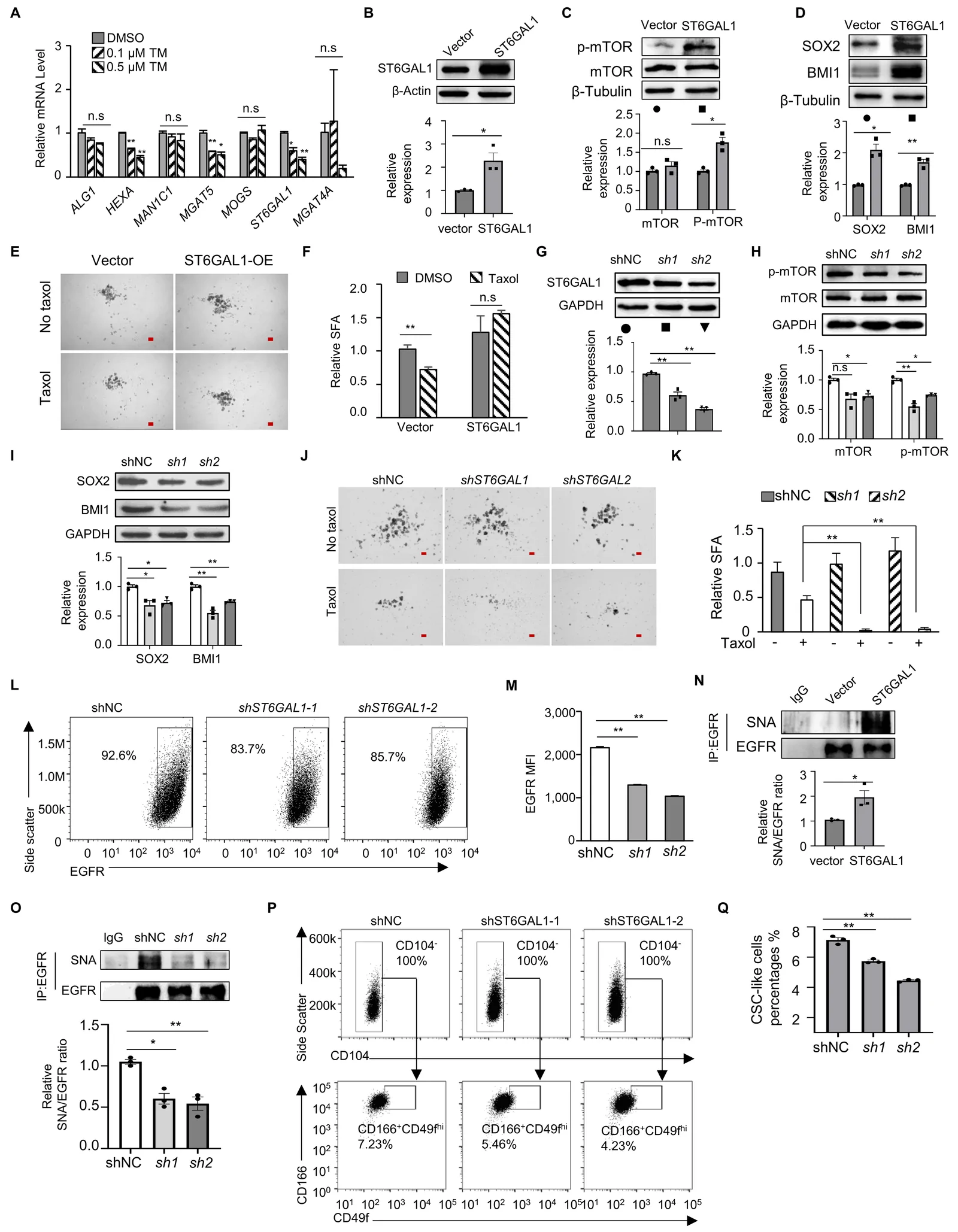
ST6GAL1 sustained CSLC-associated paclitaxel resistance through the EGFR-mTOR-SOX2/BMI1 axis. (A) RT-qPCR analysis of 7 N-glycosylation-related genes in A549-TR spheres treated with 0.1 or 0.5 μM TM, *n* = 3; (B) Representative western blots and quantification of ST6GAL1 in H1299 cells transduced with S*T6GAL1*. Statistical data were from three independent biological replicates (*n* = 3); (C and D) Representative western blots and quantification of p-mTOR (C), BMI1, and SOX2 (D) in H1299 cells transduced with *ST6GAL1*. Statistical data were from three independent biological replicates (*n* = 3); (E and F) Representative images (E) and quantification (F) of the SFA of H1299 cells transduced with *ST6GAL1* and treated with 117 nM paclitaxel, *n* = 4; (G) Representative western blots and quantification of ST6GAL1 in A549-TR cells transduced with *shST6GAL1*. Statistical data were from three independent biological replicates (*n* = 3); (H and I) Representative western blots and quantification of p-mTOR (H), BMI1, and SOX2 (I) in A549-TR cells transduced with *shST6GAL1*. Statistical data were from three independent biological replicates (*n* = 3); (J and K) Representative images and quantification of the SFA of A549-TR cells transduced with *shST6GAL1* and treated with 117 nM paclitaxel; *n* = 4; (L and M) Representative gating plots of EGFR (L) and MFI of EGFR expression (M) in A549-TR cells transduced with *shST6GAL1*, *n* =3; (N and O) Representative SNA lectin blots, corresponding EGFR immunoblots, and quantification of SNA signals normalized to immunoprecipitated EGFR in H1299 cells overexpressing ST6GAL1 (N) and A549-TR cells expressing shST6GAL1 (O). Normal IgG was used as a negative control for immunoprecipitation. Data were obtained from three independent biological replicates (*n* = 3); (P and Q) Representative gating plots (P) and quantification (Q) of CD104^-^CD166^+^CD49f^hi^ CSLC subpopulations in A549-TR cells with *ST6GAL1* knockdown, *n* = 3. n.s., not significant; ^*^P < 0.05; ^**^P < 0.01. CSLC: Cancer stem-like cell; EGFR: epidermal growth factor receptor; mTOR: mechanistic target of rapamycin; RT-qPCR: reverse transcription quantitative polymerase chain reaction; TM: tunicamycin; SFA: sphere-forming ability; MFI: mean fluorescence intensity; SNA: *Sambucus nigra* agglutinin; DMSO: dimethyl sulfoxide; sh1: *shST6GAL1-1*; sh2: *shST6GAL1-2*; IP: immunoprecipitation; CSC: cancer stem cell.

To identify the specific N-glycosylation-related genes involved in paclitaxel resistance, we screened for genes that were downregulated upon TM treatment and observed a significant decrease in *HEXA*, *MGAT5*, and *ST6GAL1* levels [Figure 6A]. Among these candidates, ST6GAL1 showed the most pronounced enrichment in paclitaxel-resistant spheres and was decreased upon treatment with TM in a concentration-dependent manner, suggesting it is closely associated with the N-glycosylation-associated paclitaxel-resistant phenotype. Previous studies have shown that ST6GAL1 is involved in the regulation of chemoresistance and stemness in ovarian^[34]^ and colorectal^[22]^ cancers and is highly expressed in NSCLC^[35]^. Based on our previous findings and other evidence, ST6GAL1 was selected for further functional validation. Notably, ST6GAL1 overexpression was confirmed by western blotting [Figure 6B], and it significantly increased phospho-mTOR levels [Figure 6C] and the expression of BMI1 and SOX2 [Figure 6D]. Based on functional assays, overexpressing ST6GAL1 did not markedly increase paclitaxel resistance in adherent parental cells [Supplementary Figure 6A and B]. However, ST6GAL1-overexpressing spheres exhibited significantly higher tolerance to paclitaxel [Figure 6E and F], indicating that the effect of ST6GAL1 was more evident in CSLC-enriched models. Furthermore, ST6GAL1 knockdown [Figure 6G] decreased phospho-mTOR levels [Figure 6H] and the expression of BMI1 and SOX2 [Figure 6I]. Consistent with the overexpression results, knockdown of ST6GAL1 did not reverse the paclitaxel resistance in adherent cells [Supplementary Figure 6C and D]. However, ST6GAL1 silencing significantly sensitized resistant spheres to paclitaxel, indicating that shST6GAL1 could reverse resistance of paclitaxel-resistant spheres [Figure 6J and K]. Moreover, ST6GAL1 knockdown significantly decreased EGFR expression in paclitaxel-resistant cells [Figure 6L and M]. SNA lectin blotting of immunoprecipitated EGFR further showed that ST6GAL1 overexpression increased EGFR α2,6-sialylation in H1299-ST6GAL1 cells [Figure 6N], whereas ST6GAL1 knockdown reduced EGFR α2,6-sialylation in A549-TR-shST6GAL1 cells [Figure 6O], supporting the role of ST6GAL1 in regulating EGFR α2,6-sialylation. Flow cytometric analysis further demonstrated a significant decrease in the CD104^-^CD166^+^CD49f^hi^ population [Figure 6P and Q], indicating a reduced CSLC subpopulation upon ST6GAL1 knockdown. These findings suggested that ST6GAL1 contributes to CSLC-mediated paclitaxel resistance by promoting EGFR α2,6-sialylation and sustaining downstream mTOR-SOX2/BMI1 signaling, rather than directly inducing broad resistance in adherent bulk cells.

### ST6GAL1 was associated with chemotherapy exposure and survival among chemotherapy-treated patients

**Figure 7:**
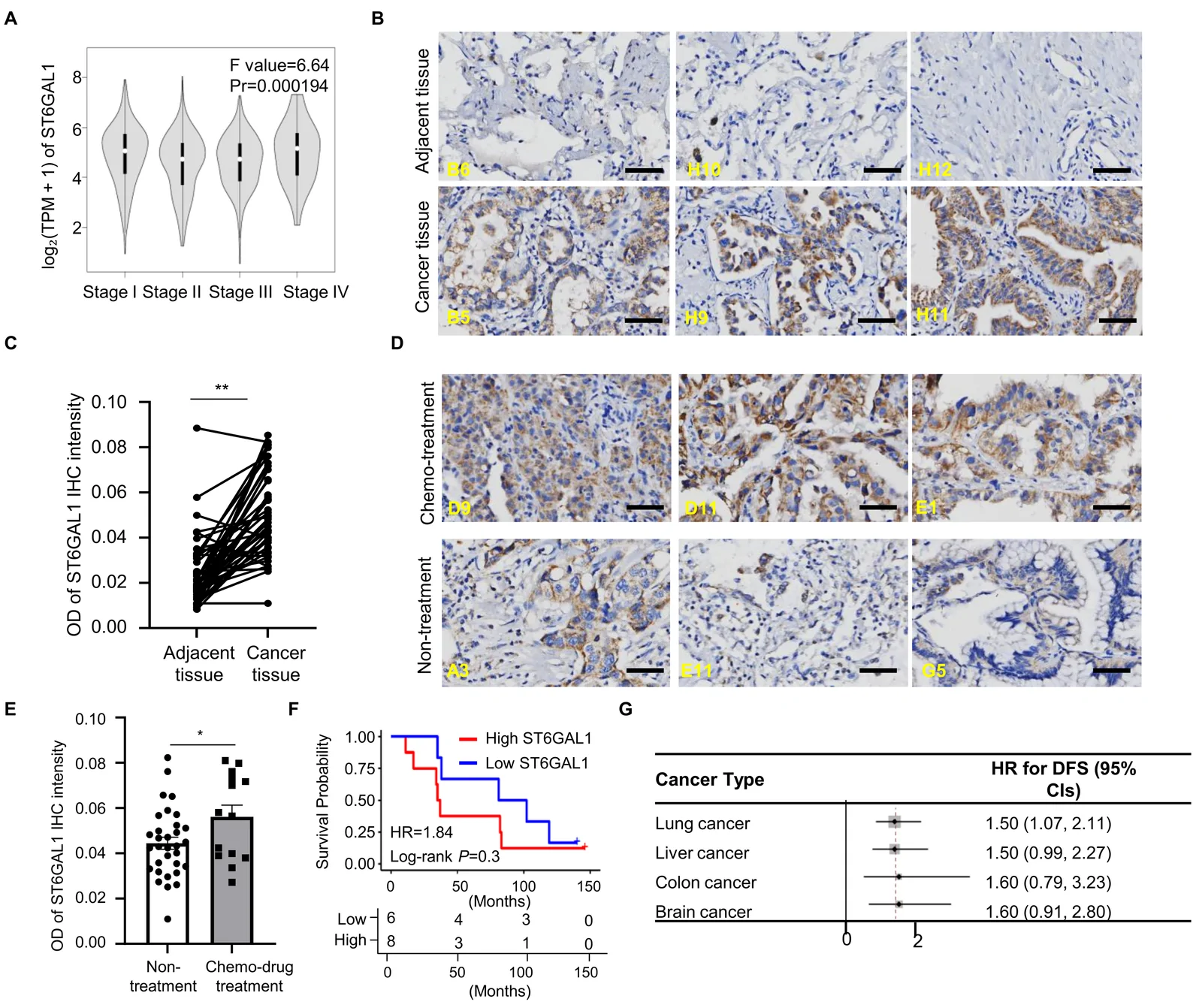
Clinical relevance of ST6GAL1 and the seven-gene N-glycosylation signature in lung cancer. (A) Violin plot showing ST6GAL1 expression across stages I-IV of lung cancer in the TCGA dataset, analyzed via GEPIA2; (B and C) Immunostaining (B) and quantification (C) of ST6GAL1 in adjacent normal tissues and tumor tissues from patients with lung cancer; scale bar, 50 µm; (D and E) Immunostaining (D) and quantification (E) of ST6GAL1 in cancer tissues from clinical lung cancer patients with or without chemotherapy; scale bar, 50 µm; (F) Post-chemotherapy survival curves of patients with high *vs.* low ST6GAL1 expression. The difference between groups was evaluated using the log-rank test, and the HR and 95%CI were estimated using Cox proportional hazards regression; (G) Forest plot showing HRs for DFS based on the expression of seven N-glycosylation-related genes across multiple cancer types. ^*^P < 0.05; ^**^P < 0.01. TCGA: The Cancer Genome Atlas; HR: hazard ratio; CI: confidence interval; DFS: disease-free survival; TPM: transcripts per million.

To evaluate the clinical relevance of ST6GAL1, we analyzed its mRNA levels in the TCGA lung cancer dataset and found that it was significantly elevated in patients with stage IV disease (*P* = 0.000194; Figure 7A). Immunohistochemical analysis revealed that ST6GAL1 expression was significantly higher in clinical lung cancer tissues than in adjacent non-tumor tissues [Figure 7B and C]. Moreover, ST6GAL1 levels were significantly higher in tumors from patients who had received chemotherapy than in those from untreated patients, indicating an association between ST6GAL1 expression and previous chemotherapy exposure [Figure 7D and E]. Survival analysis showed a lower survival probability among chemotherapy-treated patients with high ST6GAL1 expression (HR = 1.84, 95%CI: 0.57-5.90), although the difference was not statistically significant (log-rank *P* = 0.3; Figure 7F). To evaluate the broader clinical relevance of N-glycosylation-associated genes, DFS analysis was conducted across multiple cancer types. The seven-gene signature was significantly associated with DFS in patients with lung cancer (HR = 1.50, 95%CI: 1.07-2.11), whereas no significant associations were observed in patients with other cancer types [Figure 7G and Supplementary Figure 7]. These findings indicated that N-glycosylation-associated genes, particularly ST6GAL1, may have prognostic value in NSCLC.

## DISCUSSION

Our study revealed an association between acquired paclitaxel resistance and increased CSLC populations in NSCLC. Paclitaxel-resistant spheres expressed higher levels of stemness-associated factors, including SOX2 and BMI1, and exhibited an expansion of the CD104^-^CD166^+^CD49f^hi^ subpopulation. Integrative transcriptomic analysis revealed activation of N-glycosylation-related programs in paclitaxel-resistant spheres and that a seven-gene signature including ST6GAL1 was associated with shorter DFS. Inhibition of N-glycosylation or knockdown of ST6GAL1 reduced EGFR expression, suppressed mTOR signaling, downregulated SOX2/BMI1, and decreased CSLC populations. Notably, ST6GAL1 depletion had a stronger effect on paclitaxel sensitivity in resistant spheres than in adherent cells, indicating that ST6GAL1 has a stemness-associated role in chemoresistant NSCLC. Together, these findings highlight ST6GAL1-dependent EGFR α2,6-sialylation and downstream mTOR-SOX2/BMI1 signaling as a key mechanism of CSLC-associated acquired chemoresistance, suggesting it as a therapeutic target against paclitaxel-resistant NSCLC.

Previous studies have reported that CSLC enrichment is a key driver of chemoresistance, where CSLCs evade cytotoxic therapy by maintaining stem-like properties such as self-renewal and quiescence^[36-38]^. Our findings are consistent with this concept and also suggest that post-translational dysregulation may contribute to maintaining stem-like resistant states. Rather than simply comparing parental and resistant bulk-cell populations, our comparison between paclitaxel-resistant adherent cells and spheres specifically examined the stemness-associated mechanisms of acquired chemoresistance. Our transcriptomic comparison of paclitaxel-resistant adherent cells and spheres highlighted N-glycosylation, a post-translational modification that regulates the folding, stability, localization, and activity of membrane and secreted proteins^[39]^, as one of the most prominently enriched biological processes. The role of aberrant glycosylation in acquired paclitaxel-resistant NSCLC remains insufficiently defined, although it has been linked to chemoresistance and CSLC-related phenotypes. Our results advance this field by identifying N-glycosylation as a candidate mechanism of CSLC maintenance in chemoresistant NSCLC, and the functional relevance of this pathway in stem-like resistant cells was supported by subsequent ST6GAL1 validation.

Our results from integrating transcriptomic analyses of resistant adherent cells and spheres support a model in which N-glycosylation regulates CSLC-associated paclitaxel resistance through the EGFR-mTOR signaling axis. ST6GAL1, one of the N-glycosylation-associated genes, was validated as a functional regulator of stem-like properties in paclitaxel-resistant spheres. ST6GAL1 has been implicated in metastatic progression via sialylation of PECAM-1^[40]^, in EGFR activation and trafficking through EGFR sialylation^[41]^, and in sphere growth promotion and chemoresistance in ovarian cancer^[34]^. EGFR exhibited a positive correlation with N-glycosylation at the transcript and protein levels, suggesting that it is a glycosylation-responsive receptor in chemoresistant NSCLC. This possibility is supported by recent evidence that ST6GAL1-mediated sialylation promotes EGFR activation and modulates receptor trafficking dynamics^[41]^. Consistently, SNA lectin blot analysis of immunoprecipitated EGFR showed that ST6GAL1 overexpression increased EGFR α2,6-sialylation, whereas ST6GAL1 knockdown reduced EGFR α2,6-sialylation. These bidirectional findings provide biochemical evidence that ST6GAL1 regulates EGFR α2,6-sialylation in NSCLC cells. As receptor glycosylation can modulate membrane localization, stability, trafficking, and downstream signaling^[42,43]^, ST6GAL1-mediated α2,6-sialylation of EGFR may contribute to sustained EGFR signaling and therapeutic resistance in NSCLC.

EGFR levels were also increased in CSLCs, and its inhibition downregulated mTOR signaling rather than AMPK signaling, highlighting its role as a glycosylation-responsive receptor and suggesting mTOR as the major downstream effector of our model’s EGFR-dependent branch. This hypothesis is consistent with our previous observation that EGFR inhibition via afatinib attenuated stemness in chemoresistant cells^[44]^. While EGFR and mTOR signaling are widely implicated in NSCLC progression and mTOR has recently emerged as a therapeutic target in lung cancer^[45]^, our study connects this axis to glycosylation-dependent maintenance of CSLC phenotypes in acquired chemoresistance. Glycosylation regulates EGFR activity and signaling networks involved in epithelial-mesenchymal transition, CSLC maintenance, and therapy resistance^[37,46,47]^. Consistent with this broader regulatory role, our findings further highlight EGFR-mTOR signaling as a key downstream axis in N-glycosylation associated with the CSLC phenotype.

Our findings provide mechanistic evidence that N-glycosylation sustains CSLC-associated resistant states mainly through protein-level regulation rather than transcriptional activation of stemness-associated genes. Although inhibition of N-glycosylation did not significantly alter the mRNA levels of the canonical stemness transcription factors SOX2 and BMI1, their protein levels were markedly reduced, indicating protein-level regulation. Among the glycosylation-related genes, ST6GAL1 was identified as an upstream regulator of EGFR abundance and signaling. The bidirectional changes in EGFR α2,6-sialylation following ST6GAL1 overexpression or knockdown support a biochemical link between ST6GAL1-mediated sialylation and EGFR regulation, which may contribute to downstream mTOR signaling. This signaling cascade promoted SOX2/BMI1 expression and reinforced CSLC-associated phenotypes in resistant spheres. Recent evidence shows that BMI1-related signaling can contribute to osimertinib resistance^[48]^, which is consistent with BMI1’s role in NSCLC drug resistance.

ST6GAL1 overexpression did not affect paclitaxel resistance in adherent parental cells, while its knockdown had minimal effects on adherent paclitaxel-resistant cells. In contrast, ST6GAL1 overexpression increased paclitaxel resistance in spheres, and its knockdown sensitized resistant spheres to paclitaxel. This context-dependent effect is consistent with our RNA-seq results that identified ST6GAL1 by comparing paclitaxel-resistant adherent cells and spheres to determine CSLC-associated molecular features. ST6GAL1 expression increased by about 2-fold in adherent A549-TR cells *vs.* by about 32-fold in A549-TR spheres, indicating a preferential association with the sphere-enriched resistant state. Furthermore, ST6GAL1 regulated the stemness-associated proteins SOX2 and BMI1, suggesting that ST6GAL1 contributes to CSLC-mediated chemoresistance rather than general drug resistance in bulk adherent cells.

Our data suggest that mTOR and AMPK differentially contribute to chemoresistance. Recent evidence has implicated AMPK/mTOR signaling in paclitaxel resistance, highlighting the broader relevance of this pathway in tumor chemoresistance^[49]^. Although both pathways responded to N-glycosylation inhibition, only mTOR exhibited a close association with EGFR activity. These findings suggest that mTOR is the principal downstream effector of the EGFR-dependent branch, and AMPK may function as a parallel stress-adaptive pathway involved in chemoresistance. Taken together, these observations indicate that aberrant N-glycosylation promotes CSLC-mediated paclitaxel resistance through the EGFR-mTOR-SOX2/BMI1 axis and provide a mechanistic explanation for the persistence of CSLCs under chemotherapy pressure.

Our integrative analyses identified a seven-gene N-glycosylation signature significantly associated with shorter DFS in lung cancer patients, suggesting it has potential value as a prognostic, chemoresistance, and recurrence biomarker in lung cancer. However, this signature was not significantly associated with OS, suggesting it is more closely associated with recurrence or post-treatment disease control than overall mortality. Based on these findings, further preclinical evaluation of aberrant N-glycosylation and its downstream effectors in CSLC-associated paclitaxel resistance is warranted, including validation of the effects of targeting ST6GAL1 or the EGFR–mTOR axis in animal or organoid models. Consistent with this interpretation, ST6GAL1 validation in 46 clinical lung tumor tissues revealed higher expression in tumors from chemotherapy-treated patients. Along with ST6GAL1 upregulation in stage IV disease, these findings suggest ST6GAL1 may be clinically relevant in advanced and chemoresistant NSCLC.

In a previous study, ST6GAL1 knockdown enhanced chemoradiation-induced apoptosis in a patient-derived rectal cancer organoid model, whereas higher ST6GAL1 expression in post-treatment tumor specimens was associated with a poorer pathological response^[50]^. These findings further support a role for ST6GAL1 in treatment resistance. However, further validation in lung cancer organoids or xenograft models is needed to determine whether targeting ST6GAL1 can restore paclitaxel sensitivity in NSCLC by suppressing cancer stem-like properties.

This study has limitations. First, our findings were validated using *in vitro* models and clinical lung cancer tissues. While sphere cultures enrich CSLC populations, they do not fully recapitulate the tumor microenvironment’s cellular and structural complexity. Therefore, whether ST6GAL1 silencing or N-glycosylation inhibition can enhance paclitaxel sensitivity *in vivo* remains to be determined. Studies in animal models, lung cancer organoids, or tumor-derived tissue slice cultures are needed to establish the physiological and therapeutic relevance of these findings. Moreover, although SNA lectin blot analysis demonstrated that ST6GAL1 regulates EGFR α2,6-sialylation, the specific glycosylation sites and the mechanisms by which this modification affects EGFR activation remain to be determined. Finally, although we highlight ST6GAL1 as a promising therapeutic target, further investigation is required to determine its broader relevance across NSCLC subtypes and other malignancies.

In conclusion, this study identified a seven-gene N-glycosylation signature associated with the CSLC-enriched state in paclitaxel-resistant NSCLC cells. Cell culture models identified ST6GAL1 as a key regulator of CSLC-associated acquired paclitaxel resistance. Mechanistically, ST6GAL1 reinforced stem-like properties and CSLC-associated paclitaxel resistance by promoting EGFR α2,6-sialylation and increasing EGFR abundance, thereby sustaining mTOR signaling and SOX2/BMI1 expression. Moreover, the seven-gene N-glycosylation signature was associated with a shorter DFS but not with OS in lung cancer patients, supporting its potential as a biomarker of chemoresistance and recurrence risk. The analysis of 46 clinical lung tumor tissues showed that ST6GAL1 expression was associated with chemotherapy exposure and that patients with higher ST6GAL1 expression tended to have poorer post-chemotherapy survival. Together, these findings implicate ST6GAL1-dependent EGFR α2,6-sialylation and the downstream mTOR-SOX2/BMI1 axis in CSLC-associated acquired paclitaxel resistance and provide a rationale for evaluating aberrant glycosylation as a potential target for overcoming stemness-associated chemoresistance in NSCLC.

## DECLARATIONS

### Availability of data and materials

The datasets analyzed in the current study are available from the corresponding authors upon reasonable request. The raw sequence data for RNA-seq reported in this paper have been deposited in the Genome Sequence Archive in the National Genomics Data Center, China National Center for Bioinformation/Beijing Institute of Genomics, Chinese Academy of Sciences (GSA-Human: HRA011038), which is publicly accessible at https://ngdc.cncb.ac.cn/gsa-human.

### AI and AI-assisted tools statement

During the preparation of this manuscript, the authors used ChatGPT (OpenAI; model: GPT-4, released 2023-03-14) for English language editing, grammar checking, and improving sentence clarity. The authors carefully reviewed and edited all AI-generated content and accept full responsibility for the published material.

### Copyright

© The Author(s) 2026.
