# Supplementary material for: ST6GAL1 promotes cancer stem-like cell-associated paclitaxel resistance through the EGFR-mTOR-SOX2/BMI1 axis in non-small cell lung cancer
Source: Cancer Drug Resist. 2026 Aug 31;9:32. doi: 10.20517/cdr.2026.66 (PMC13589886; doi:10.20517/cdr.2026.66)
Supplement: Supplementary file 1 [file cdr-9-32-SupplementaryFigures.pdf]

Supplementary Figures

ST6GAL1 promotes cancer stem-like cell-associated paclitaxel resistance through the EGFR-mTOR-SOX2/BMI1 axis in non-small cell lung cancer

Yuxi Yang[1,#], Binghui Liang[1,#], Weijie Hong[1,#], Sau Har Lee[2], Dongfang Tang[3,4], Shuxian Xie[5], Changtai Qin[1], Liu Shen[1], Zhumei Sun[1], Xiaofeng Yan[1], Hua Li[1], Xiaoling Wang[1], Xudong Hu[1], Tingjie Ye[1], Wei Zhang[6], Wei Xu[1]

[1]School of Integrative Medicine, Shanghai University of Traditional Chinese Medicine, Shanghai 201203, China.

[2]School of Biosciences, Faculty of Health and Medical Sciences, Taylor's University, Subang Jaya 47500, Malaysia.

[3]Department of Thoracic Surgery, Huadong Hospital affiliated with Fudan University, Shanghai 200040, China.

[4]Department of Surgical Oncology, Shanghai Lung Cancer Center, Shanghai Chest Hospital, Shanghai Jiao Tong University School of Medicine, Shanghai 200030, China.

[5]School of Biological Sciences, University of California San Diego, San Diego, CA 92093, USA.

[6]Department of Clinical Laboratory, Peking University Shenzhen Hospital, Shenzhen 518036, Guangdong, China.

[#]These authors contributed equally to this work.

Correspondence to: Dr. Wei Xu, Dr. Tingjie Ye, School of Integrative Medicine, Shanghai University of Traditional Chinese Medicine, Shanghai 201203, China. E-mail: wei-xu11@tsinghua.org.cn; 0000001901@shutcm.edu.cn; Dr. Wei Zhang, Department of Clinical Laboratory, Peking University Shenzhen Hospital, Shenzhen 518036, Guangdong, China. E-mail: w-z12@tsinghua.org.cn

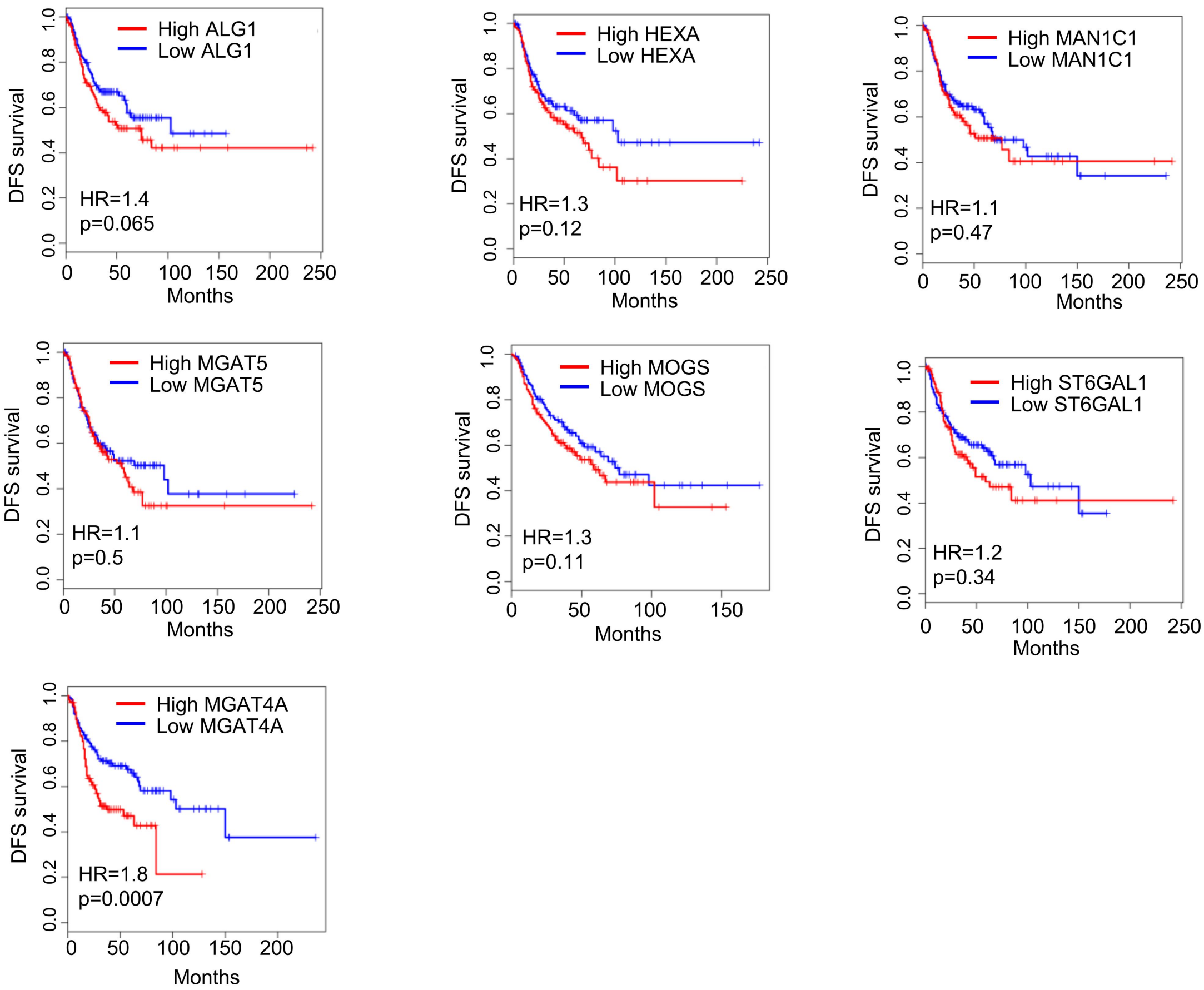

Supplementary Figure 1. Disease-free survival analysis of individual genes from the seven-gene N-glycosylation signature in lung cancer. Kaplan-Meier curves comparing disease-free survival in 241 lung cancer patients stratified by high *vs.* low expression of each of the seven upregulated N-glycosylation-related genes. DFS: Disease-free survival; HR: hazard ratio.

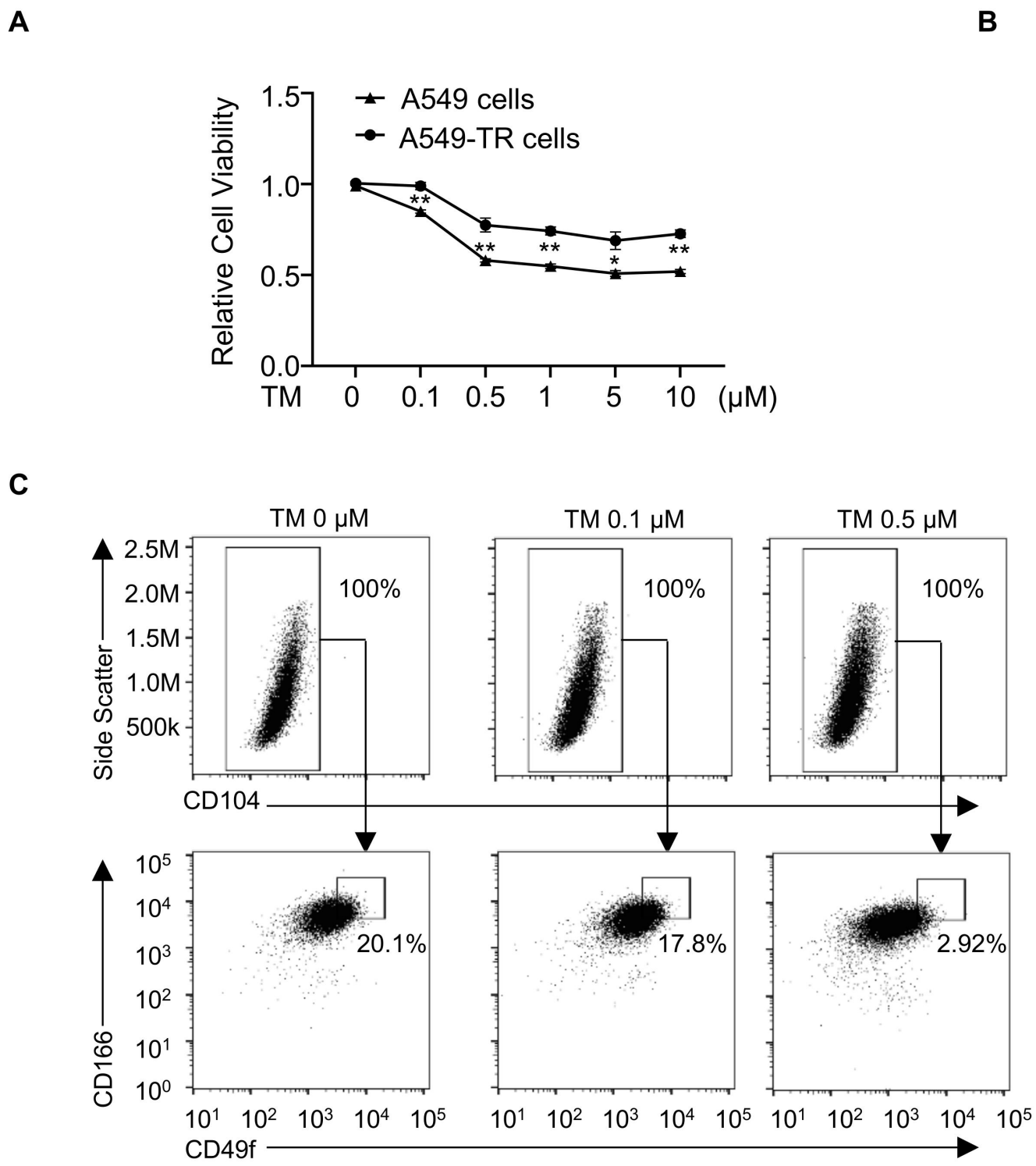

Supplementary Figure 2. Inhibition of N-glycosylation reduced CSLC-associated phenotypes. (A) Relative cell growth of A549-TR cells and A549 cells treated with different doses of TM for 48 h; $n = 3$; (B) Relative cell growth of H1299-TR cells and H1299 cells treated with different doses of TM for 48 h; $n = 3$; (C) Representative gating plots for $CD104^{-}CD166^{+}CD49f^{hi}$ CSLC subpopulations in H1299-TR cells after 48 h of TM treatment. TM, tunicamycin. $^{**}P < 0.01$; $^{*}P < 0.05$.

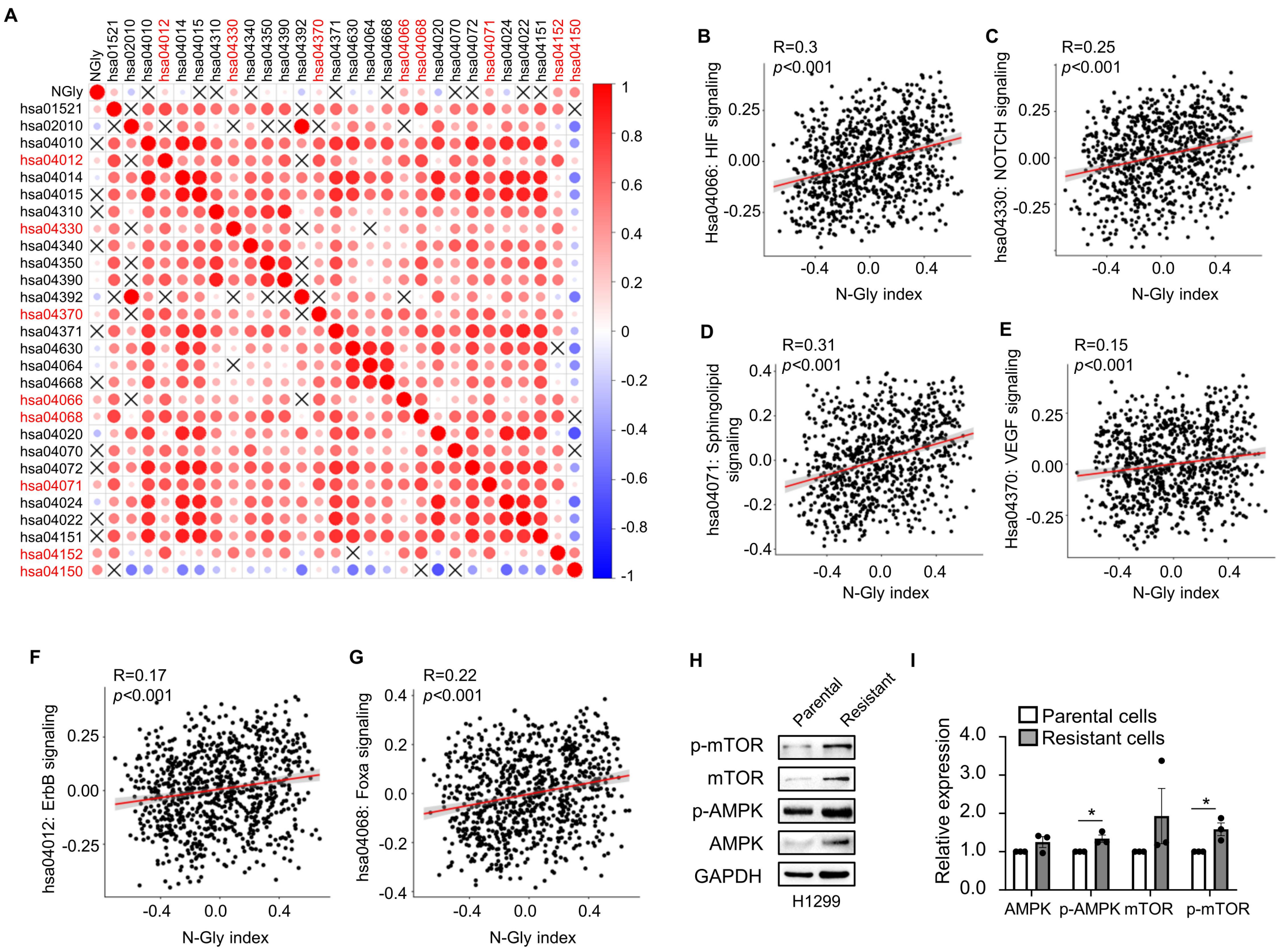

Supplementary Figure 3. mTOR and AMPK signaling were activated in paclitaxel-resistant cells and associated with N-glycosylation-related pathways. (A) Correlation heatmap showing the associations between N-glycosylation and oncogenic signaling pathways in 870 lung cancer samples from the TCGA cohort. Pearson correlation coefficients were calculated on the basis of GSVA enrichment scores for 19 upregulated N-glycosylation-related genes and gene sets representing individual oncogenic pathways. Red indicates pathways positively correlated with N-glycosylation; (B-G) Scatter plots showing the correlation between the N-glycosylation index and selected oncogenic signaling pathways, including the HIF (B), NOTCH (C), sphingolipid (D), VEGF (E), ErbB (F), and Foxa (G) pathways; (H) Representative western blot images and (I) corresponding quantification of phosphorylated mTOR (p-mTOR) and phosphorylated AMPK (p-AMPK) in parental H1299 and paclitaxel-resistant H1299-TR cells, $n = 3$. $^{*}P < 0.05$; $^{**}P < 0.01$.

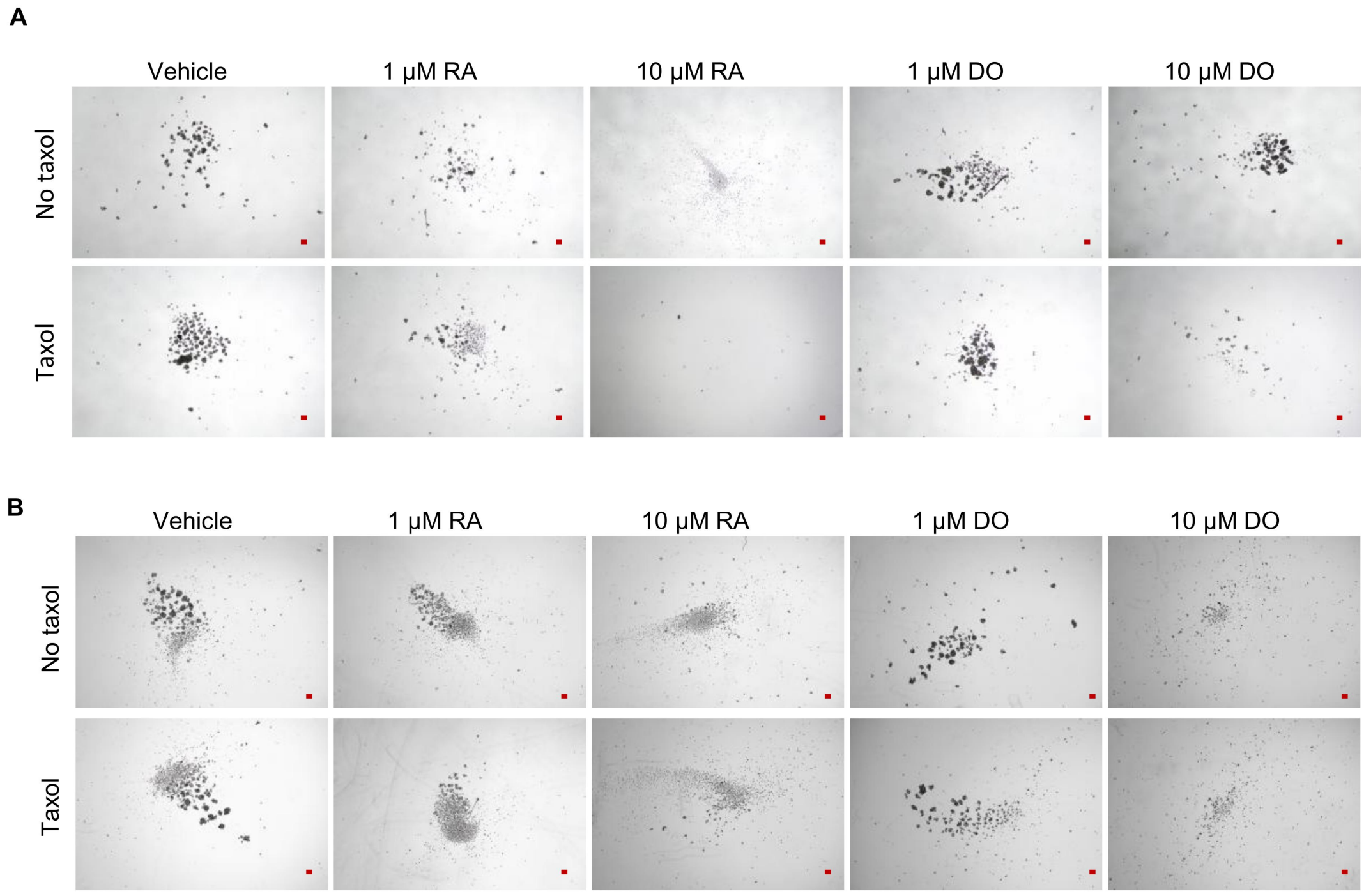

Supplementary Figure 4. mTOR and AMPK inhibition modulated paclitaxel sensitivity in resistant spheres. (A and B) Representative images showing the sphere formation of A549-TR (A) and H1299-TR (B) cells following treatment with paclitaxel alone or in combination with RA or DO at 1 or 10 µM. RA, rapamycin; DO, dorsomorphin; scale bar, 100 µm.

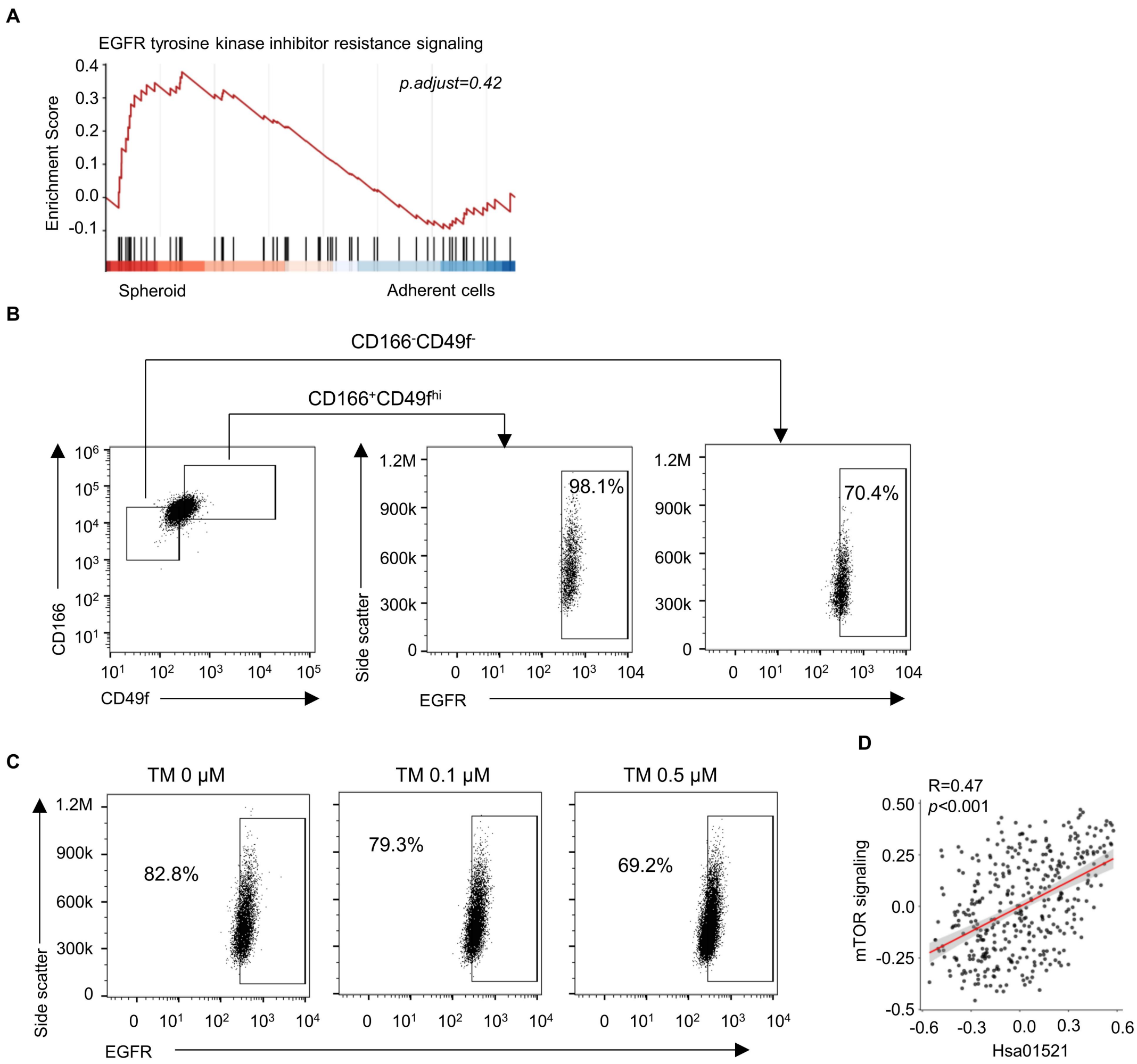

Supplementary Figure 5. EGFR was implicated as a downstream effector of N-glycosylation in paclitaxel-resistant CSLCs. (A) Gene set enrichment analysis (GSEA) plot showing EGFR tyrosine kinase inhibitor resistance signaling; (B) Representative flow cytometry plots showing EGFR expression in $CD104^{-}$-gated CSLCs ($CD166^{+}CD49f^{hi}$) and non-CSLCs ($CD166^{-}CD49f^{-}$) from A549-TR cells; (C) Representative FACS gating of EGFR expression in A549-TR cells treated with TM; TM, tunicamycin; (D) Pearson correlation analysis of mTOR signaling with hsa01521 using TMT-quantified protein data from 378 cancer cell lines.

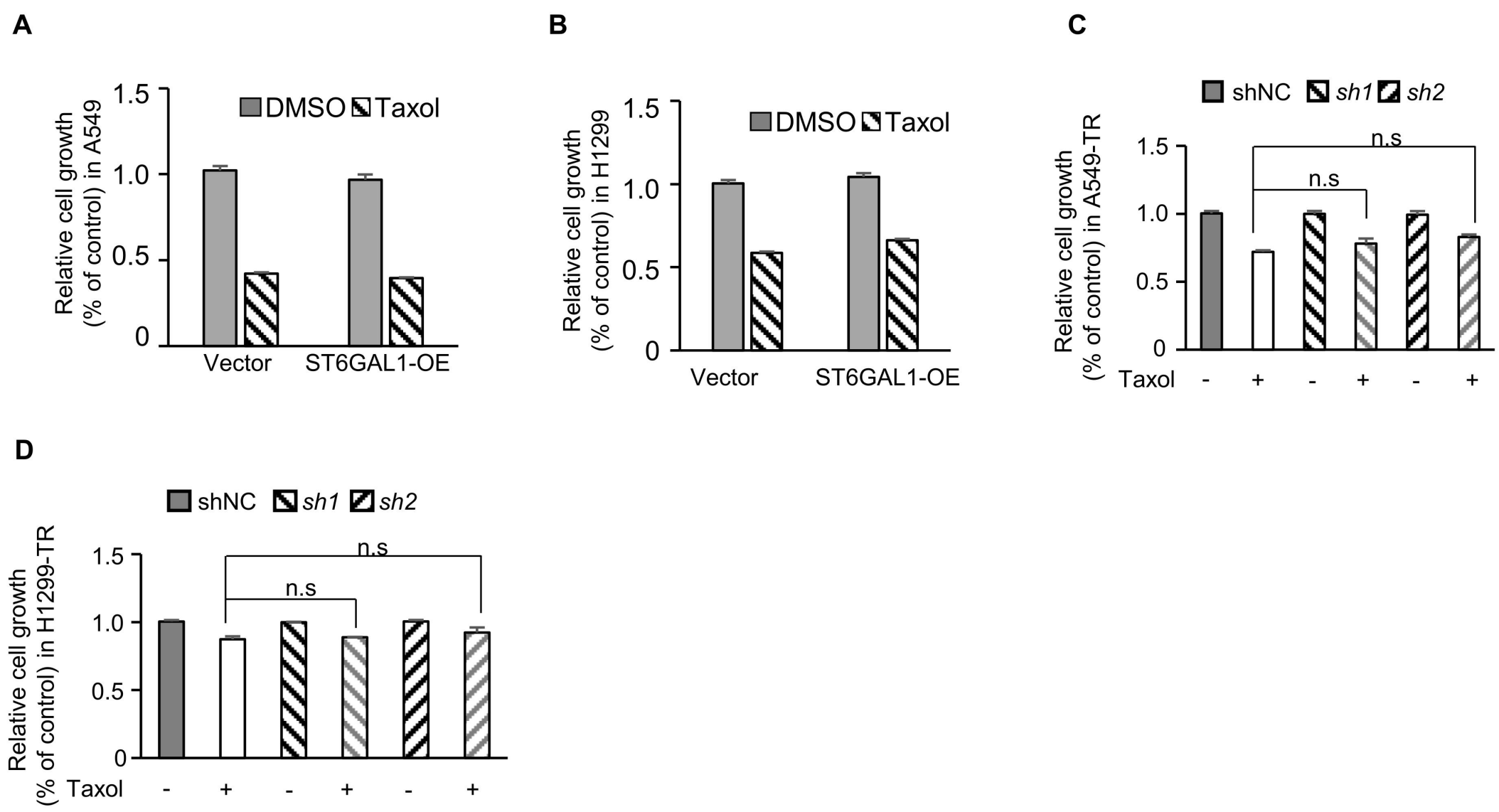

Supplementary Figure 6. Effects of ST6GAL1 modulation on the paclitaxel response of adherent cells. (A and B) Relative cell growth of A549 cells (A) and H1299 cells (B) transduced with ST6GAL1, with or without paclitaxel treatment, $n = 3$; (C and D) Relative cell growth of paclitaxel-resistant A549-TR cells (C) and H1299-TR cells (D) transduced with shST6GAL1, with or without paclitaxel treatment, $n = 3$. n.s., not significant; $^{**}P < 0.01$; $^{*}P < 0.05$.

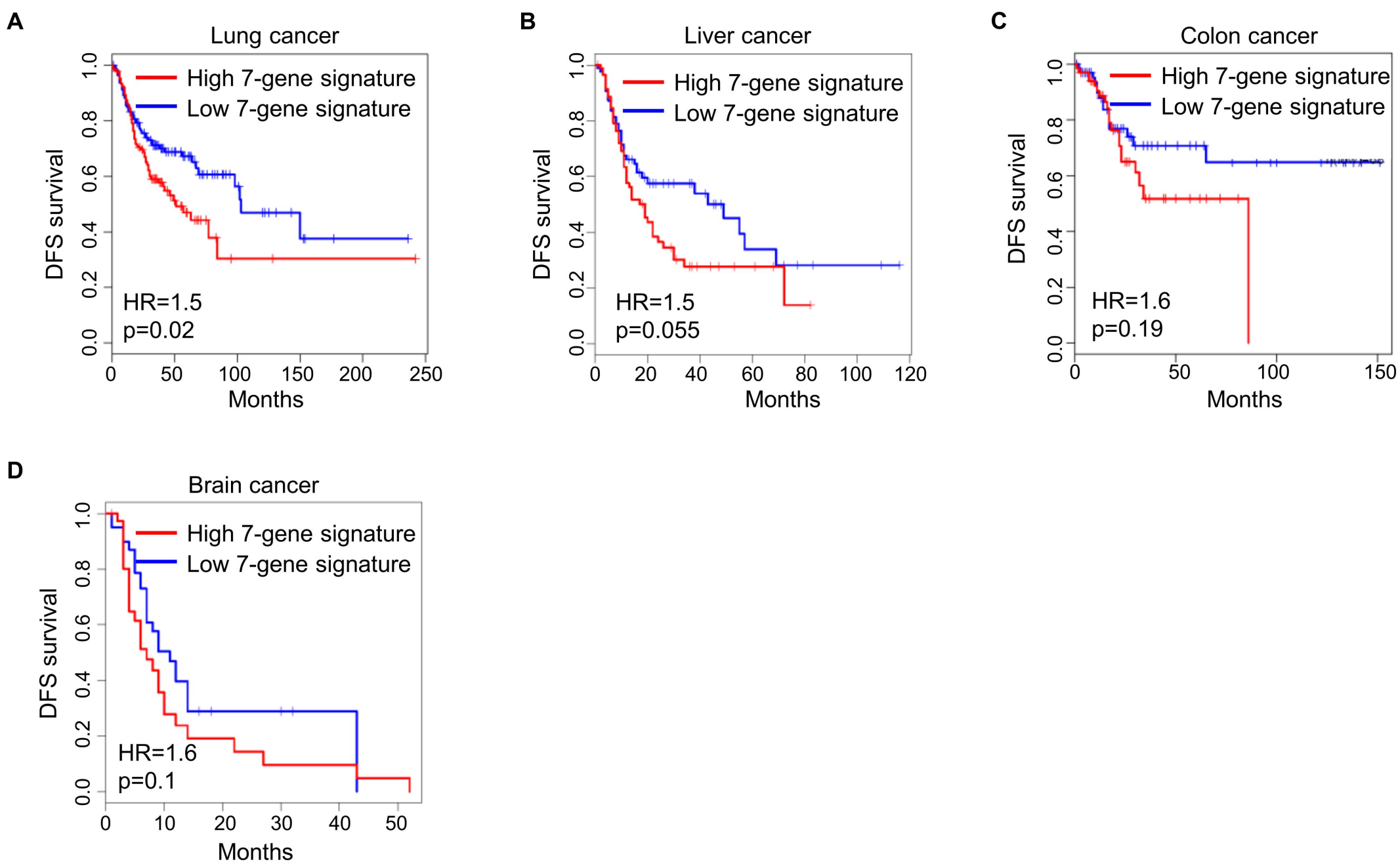

Supplementary Figure 7. Disease-free survival analysis of the seven-gene N-glycosylation signature across multiple cancer types. (A-D) Kaplan-Meier analysis of DFS in patients with lung (A), liver (B), colon (C), and brain (D) cancers stratified according to high *vs.* low expression of the seven-gene N-glycosylation signature. DFS: Disease-free survival; HR: hazard ratio.
