# Supplementary material for: ST6GAL1 promotes cancer stem-like cell-associated paclitaxel resistance through the EGFR-mTOR-SOX2/BMI1 axis in non-small cell lung cancer
Source: Cancer Drug Resist. 2026 Aug 31;9:32. doi: 10.20517/cdr.2026.66 (PMC13589886; doi:10.20517/cdr.2026.66)
Supplement: Supplementary file 3 [file cdr-9-32-Supplementaryfiles.zip › Supplementary File 2-Table S2.docx]

**Table S2.** The list of antibodies used in the study

| **Antibody** | **Vendor^1^** | **Catlog number** | **Clone / Cat. no.** | **Species^2^** | **WB** | **FACs** | **IHC** | **IP** |
| --- | --- | --- | --- | --- | --- | --- | --- | --- |
| β-Tubulin | EB | AT0050 | 6C12 | M | 1:5000 | n.a | n.a | n.a |
| GAPDH | EB | AT0049 | 3G7 | M | 1:5000 | n.a | n.a | n.a |
| β-Actin | Proteintech | 66009-1-Ig | 2D4H5 | M | 1:5000 | n.a | n.a | n.a |
| c-MYC | Proteintech | 10828-1-AP | n.a | Rb | 1:1000 | n.a | n.a | n.a |
| BMI1 | Proteintech | 10832-1-AP | n.a | Rb | 1:1000 | n.a | n.a | n.a |
| KLF4 | Proteintech | 11880-1-AP | n.a | Rb | 1:500 | n.a | n.a | n.a |
| NANOG | Proteintech | 14295-1-AP | n.a | Rb | 1:500 | n.a | n.a | n.a |
| SOX2 | Santa Cruz | sc-365964 | A-5 | M | 1:500 | n.a | n.a | n.a |
| mTOR | Proteintech | 66888-1-Ig | 1G11A3 | M | 1:2000 | n.a | n.a | n.a |
| p-mTOR | Proteintech | 67778-1-Ig | 2A12G3 | M | 1:1000 | n.a | n.a | n.a |
| p-AMPKα | CST | 2535 | 40H9 | Rb | 1:1000 | n.a | n.a | n.a |
| AMPKα | Proteintech | 10929-2-AP | n.a | Rb | 1:1000 | n.a | n.a | n.a |
| ST6GAL1 | Proteintech | 14355-1-AP | n.a | Rb | 1:1000 | n.a | 1:400 | n.a |
| EGFR | Proteintech | 18986-1-AP | n.a | Rb | 1:1000 | n.a | n.a | 2µg per 1 mg lysate |
| Streptavidin | Proteintech | HRP-PF00030 | n.a | n.a | 1:4000 | n.a | n.a | n.a |
| SNA | Vector Laboratories | B-1305-2 | n.a | n.a | 4 µg/mL | n.a | n.a | n.a |
| EGFR | Biolegend | 352913 | AY13 | M | n.a | 1:80 | n.a | n.a |
| CD104 | Biolegend | 327807 | 58XB4 | M | n.a | 1:50 | n.a | n.a |
| CD166 | Biolegend | 343905 | 3A6 | M | n.a | 1:50 | n.a | n.a |
| CD49f | Biolegend | 313605 | GoH3 | Rat | n.a | 1:50 | n.a | n.a |
|  | | 1. CST, Cell Signaling Technology; EB, Engibody Biotechnology (Beijing, China); | | | | |  |  |
|  | | 2. M, mouse; Rb, rabbit; | |  |  |  |  |  |
