# Supplementary material for: ST6GAL1 promotes cancer stem-like cell-associated paclitaxel resistance through the EGFR-mTOR-SOX2/BMI1 axis in non-small cell lung cancer
Source: Cancer Drug Resist. 2026 Aug 31;9:32. doi: 10.20517/cdr.2026.66 (PMC13589886; doi:10.20517/cdr.2026.66)
Supplement: Supplementary file 3 [file cdr-9-32-Supplementaryfiles.zip › Supplementary File 3-GO_analysis for 2Foldchange_genes.docx]

| **2Foldchenge_gene list** | | | | | | | | | | | | | | | |
| --- | --- | --- | --- | --- | --- | --- | --- | --- | --- | --- | --- | --- | --- | --- | --- |
| **Ensembl_ID** | **Log**  **FC** | **Ensembl_ID** | **Log**  **FC** | **Ensembl_ID** | **Log**  **FC** | **Ensembl_ID** | **Log**  **FC** | **Ensembl_ID** | **Log**  **FC** | **Ensembl_ID** | **Log**  **FC** | **Ensembl_ID** | **Log**  **FC** | **Ensembl_ID** | **Log**  **FC** |
| ENSG00000002330.13_9 | 2.987 | ENSG00000160131.13_5 | -2.399 | ENSG00000118939.17_8 | -2.378 | ENSG00000197070.14_5 | 6.070 | ENSG00000100600.15_6 | 3.173 | ENSG00000172159.16_5 | 2.360 | ENSG00000138180.16_4 | -6.823 | ENSG00000242282.6_9 | -2.540 |
| ENSG00000002587.10_7 | 4.490 | ENSG00000160145.15_5 | -3.624 | ENSG00000119242.8_7 | 7.034 | ENSG00000197119.13_6 | 3.600 | ENSG00000100629.17_8 | -2.833 | ENSG00000172164.15_7 | 2.490 | ENSG00000138182.14_3 | -4.232 | ENSG00000242574.9_8 | 8.849 |
| ENSG00000002919.14_3 | 3.313 | ENSG00000160180.15_5 | 12.466 | ENSG00000119326.15_6 | -2.519 | ENSG00000197142.10_2 | 10.829 | ENSG00000100726.15_4 | 3.881 | ENSG00000172572.6_4 | 3.169 | ENSG00000138207.14_6 | 6.959 | ENSG00000242612.7_5 | 3.655 |
| ENSG00000003436.16_7 | 2.634 | ENSG00000160181.9_5 | 10.241 | ENSG00000119335.17_4 | -3.130 | ENSG00000197249.14_7 | 15.003 | ENSG00000100749.8_5 | -3.043 | ENSG00000172663.9_8 | 2.539 | ENSG00000138376.11_6 | -2.713 | ENSG00000243137.8_7 | -5.590 |
| ENSG00000005206.17_5 | 2.854 | ENSG00000160182.3_5 | 3.583 | ENSG00000119431.9_3 | 3.562 | ENSG00000197299.12_5 | -3.430 | ENSG00000100897.18_8 | 3.614 | ENSG00000172732.12_5 | 2.569 | ENSG00000138449.11_5 | 4.099 | ENSG00000243279.4_6 | 2.451 |
| ENSG00000005238.19_4 | 3.001 | ENSG00000160200.18_4 | 4.139 | ENSG00000119632.4_6 | -2.474 | ENSG00000197301.7_8 | -4.088 | ENSG00000100911.16_6 | -2.861 | ENSG00000172748.14_7 | -2.759 | ENSG00000138587.6_5 | -3.074 | ENSG00000243364.8_5 | 3.058 |
| ENSG00000005486.17_4 | 3.944 | ENSG00000160200.18_4 | 4.139 | ENSG00000119688.21_8 | 2.499 | ENSG00000197417.9_6 | -10.211 | ENSG00000100926.15_9 | 3.135 | ENSG00000172794.20_6 | 6.615 | ENSG00000138668.19_8 | -2.377 | ENSG00000243649.9_5 | 7.815 |
| ENSG00000005882.12_7 | 3.357 | ENSG00000160207.9_6 | -2.818 | ENSG00000119705.10_8 | -2.399 | ENSG00000197467.15_6 | -5.646 | ENSG00000100949.14_4 | 2.906 | ENSG00000172828.13_6 | 4.519 | ENSG00000138675.16_4 | -4.069 | ENSG00000243710.7_6 | -3.194 |
| ENSG00000005893.16_5 | 2.501 | ENSG00000160226.16_7 | 5.535 | ENSG00000119977.21_8 | 2.484 | ENSG00000197479.7_5 | 2.848 | ENSG00000100991.12_6 | 3.873 | ENSG00000172889.16_6 | 5.336 | ENSG00000138696.11_7 | 2.514 | ENSG00000243955.6_5 | 11.340 |
| ENSG00000006606.8_4 | -4.721 | ENSG00000160285.15_5 | 3.234 | ENSG00000120162.10_5 | -2.830 | ENSG00000197498.13_4 | -2.524 | ENSG00000101003.10_4 | -2.706 | ENSG00000172893.16_7 | 3.228 | ENSG00000138744.16_9 | 3.281 | ENSG00000244038.10_7 | 2.561 |
| ENSG00000006634.8_4 | -2.412 | ENSG00000160410.15_5 | 3.715 | ENSG00000120539.15_4 | -2.509 | ENSG00000197562.10_5 | 10.414 | ENSG00000101049.17_8 | -2.867 | ENSG00000172932.16_6 | 3.548 | ENSG00000138764.15_6 | 2.932 | ENSG00000244067.3_5 | 6.119 |
| ENSG00000007080.11_4 | 2.555 | ENSG00000160446.19_8 | 4.311 | ENSG00000120594.17_4 | 3.468 | ENSG00000197746.14_6 | 3.924 | ENSG00000101057.16_4 | -3.736 | ENSG00000172936.15_8 | 2.406 | ENSG00000138772.13_6 | -3.838 | ENSG00000244161.1_7 | 3.006 |
| ENSG00000007541.17_5 | 5.966 | ENSG00000160613.13_6 | 2.784 | ENSG00000120708.17_7 | -2.881 | ENSG00000197766.8_5 | 3.355 | ENSG00000101294.18_6 | 2.998 | ENSG00000172992.12_6 | 2.622 | ENSG00000138778.13_6 | -4.783 | ENSG00000244255.5_8 | -2.743 |
| ENSG00000007952.18_6 | 5.964 | ENSG00000160712.13_5 | 2.794 | ENSG00000120802.13_6 | -2.584 | ENSG00000197858.11_4 | 3.721 | ENSG00000101439.9_6 | 3.504 | ENSG00000173068.18_8 | -2.435 | ENSG00000138821.13_6 | 3.254 | ENSG00000244560.7_6 | 5.400 |
| ENSG00000007968.7_5 | -11.760 | ENSG00000160818.16_5 | -2.530 | ENSG00000121060.18_8 | -3.734 | ENSG00000197948.11_5 | 2.834 | ENSG00000101447.15_5 | -4.129 | ENSG00000173077.16_6 | 5.649 | ENSG00000139112.11_5 | 2.381 | ENSG00000245248.8_8 | -2.806 |
| ENSG00000008513.16_9 | 3.667 | ENSG00000160886.13_5 | -3.530 | ENSG00000121152.10_6 | -4.891 | ENSG00000198026.8_5 | 2.510 | ENSG00000101474.12_6 | 2.930 | ENSG00000173137.12_4 | 4.233 | ENSG00000139178.11_7 | 2.659 | ENSG00000245848.3_4 | 3.047 |
| ENSG00000008710.20_7 | 4.622 | ENSG00000160932.11_6 | 4.492 | ENSG00000121211.8_5 | -5.094 | ENSG00000198056.15_9 | -2.705 | ENSG00000101670.12_7 | 3.625 | ENSG00000173153.16_6 | 3.064 | ENSG00000139187.10_5 | 3.708 | ENSG00000246334.2_7 | -4.554 |
| ENSG00000009709.12_4 | 3.173 | ENSG00000161204.11_4 | 2.506 | ENSG00000121621.7_5 | -3.294 | ENSG00000198125.13_6 | 4.712 | ENSG00000101911.12_4 | -3.480 | ENSG00000173163.10_6 | -2.403 | ENSG00000139192.12_5 | 3.514 | ENSG00000246859.3_8 | 4.459 |
| ENSG00000010310.9_6 | 3.933 | ENSG00000161277.11_5 | 4.429 | ENSG00000121680.16_5 | 2.573 | ENSG00000198183.12_4 | 16.005 | ENSG00000102119.11_4 | 2.997 | ENSG00000173207.13_4 | -3.049 | ENSG00000139318.8_6 | 3.799 | ENSG00000247095.3_5 | 3.536 |
| ENSG00000010361.14_7 | 4.879 | ENSG00000161395.14_6 | 2.504 | ENSG00000121716.20_7 | 3.274 | ENSG00000198276.16_6 | 2.412 | ENSG00000102181.21_7 | 4.075 | ENSG00000173237.4_3 | -2.439 | ENSG00000139351.15_6 | -2.760 | ENSG00000247596.9_6 | 4.518 |
| ENSG00000011009.11_4 | 7.423 | ENSG00000161677.12_5 | 3.692 | ENSG00000121858.11_5 | 2.779 | ENSG00000198355.5_4 | 2.711 | ENSG00000102271.14_6 | -6.355 | ENSG00000173432.12_5 | 4.199 | ENSG00000139354.11_9 | -4.038 | ENSG00000248098.12_5 | 4.007 |
| ENSG00000011028.14_5 | 2.625 | ENSG00000161860.8_7 | -10.198 | ENSG00000122035.7_3 | 4.081 | ENSG00000198356.12_7 | 3.302 | ENSG00000102312.22_6 | 5.486 | ENSG00000173486.13_4 | 2.946 | ENSG00000139410.15_9 | 2.487 | ENSG00000248167.7_4 | -12.854 |
| ENSG00000011426.11_8 | -5.050 | ENSG00000161888.11_9 | -3.381 | ENSG00000122483.17_6 | -3.001 | ENSG00000198443.6_4 | 3.905 | ENSG00000102316.17_4 | 3.238 | ENSG00000173801.17_7 | 5.310 | ENSG00000139508.15_4 | 2.587 | ENSG00000248487.9_5 | 4.211 |
| ENSG00000011485.15_4 | 2.551 | ENSG00000161955.16_4 | 3.226 | ENSG00000122574.10_2 | 3.868 | ENSG00000198520.12_9 | 2.996 | ENSG00000102359.7_4 | 4.271 | ENSG00000174028.6_2 | -3.779 | ENSG00000139547.7_4 | 11.290 | ENSG00000248698.6_7 | 2.993 |
| ENSG00000012048.23_8 | -4.190 | ENSG00000162032.16_6 | 5.047 | ENSG00000122694.16_4 | -2.912 | ENSG00000198554.12_9 | -3.092 | ENSG00000102384.13_3 | -3.791 | ENSG00000174080.11_6 | 3.718 | ENSG00000139618.15_5 | -3.686 | ENSG00000248771.5_9 | 6.269 |
| ENSG00000012822.16_7 | 3.159 | ENSG00000162063.13_7 | -2.439 | ENSG00000122861.16_5 | -2.393 | ENSG00000198590.12_7 | 2.620 | ENSG00000102452.17_6 | -2.636 | ENSG00000174227.16_5 | 3.105 | ENSG00000139625.13_6 | 3.594 | ENSG00000249209.2_8 | -2.633 |
| ENSG00000013306.16_8 | 3.606 | ENSG00000162076.13_7 | 2.954 | ENSG00000122952.17_7 | -3.334 | ENSG00000198610.11_6 | 4.350 | ENSG00000102934.10_5 | 2.872 | ENSG00000174233.11_5 | 2.492 | ENSG00000139636.15_6 | 5.038 | ENSG00000249279.6_7 | -4.811 |
| ENSG00000014216.16_7 | 4.586 | ENSG00000162078.11_5 | 6.277 | ENSG00000123219.13_8 | -3.341 | ENSG00000198624.13_5 | 3.199 | ENSG00000103024.7_6 | 2.809 | ENSG00000174371.17_5 | -5.351 | ENSG00000139679.15_4 | 3.371 | ENSG00000249565.2_5 | -2.435 |
| ENSG00000014257.16_4 | 10.839 | ENSG00000162144.10_6 | 3.286 | ENSG00000123358.20_8 | 10.264 | ENSG00000198715.13_10 | 3.451 | ENSG00000103051.20_8 | 2.648 | ENSG00000174473.16_4 | 3.261 | ENSG00000139725.8_6 | -2.408 | ENSG00000249592.6_9 | 4.052 |
| ENSG00000015413.9_6 | 10.960 | ENSG00000162241.12_4 | 2.526 | ENSG00000123384.14_5 | 3.405 | ENSG00000198722.14_6 | 3.145 | ENSG00000103066.13_7 | 5.230 | ENSG00000174521.7_3 | 2.700 | ENSG00000139734.19_7 | -5.050 | ENSG00000249773.3_6 | -10.851 |
| ENSG00000015676.18_5 | 2.466 | ENSG00000162300.13_6 | 2.942 | ENSG00000123444.14_8 | 2.451 | ENSG00000198746.13_6 | 3.981 | ENSG00000103152.12_4 | 4.744 | ENSG00000174567.8_5 | 2.486 | ENSG00000139865.16_3 | 3.955 | ENSG00000249786.7_9 | 6.183 |
| ENSG00000018236.15_7 | 3.393 | ENSG00000162396.6_5 | 3.448 | ENSG00000123737.13_5 | -2.599 | ENSG00000198774.5_6 | 3.882 | ENSG00000103187.8_6 | -3.280 | ENSG00000174669.12_5 | 3.681 | ENSG00000140092.14_6 | 4.858 | ENSG00000249867.6_9 | -2.571 |
| ENSG00000019991.18_8 | 3.604 | ENSG00000162415.7_6 | 2.561 | ENSG00000123815.12_8 | 3.653 | ENSG00000198788.7 | 11.902 | ENSG00000103196.12_6 | -3.234 | ENSG00000174851.16_6 | 3.055 | ENSG00000140297.13_7 | 3.022 | ENSG00000250021.7_9 | 10.639 |
| ENSG00000021300.14_7 | 2.687 | ENSG00000162496.9_6 | -2.857 | ENSG00000123838.11_5 | 3.254 | ENSG00000198826.11_6 | -4.396 | ENSG00000103227.19_8 | 5.649 | ENSG00000174903.16_5 | 5.222 | ENSG00000140379.8_5 | -6.371 | ENSG00000250222.2_8 | -3.083 |
| ENSG00000021488.13_5 | 2.980 | ENSG00000162512.16_7 | 3.731 | ENSG00000123843.13_6 | -10.635 | ENSG00000198830.11_4 | -3.698 | ENSG00000103245.14_8 | 4.070 | ENSG00000174915.12_4 | 2.771 | ENSG00000140398.14_5 | 3.945 | ENSG00000250329.1_4 | 4.931 |
| ENSG00000023445.16_5 | -3.414 | ENSG00000162522.11_4 | 3.528 | ENSG00000123975.5_4 | -2.544 | ENSG00000198832.10_6 | 2.945 | ENSG00000103316.11_5 | 3.274 | ENSG00000174938.14_8 | 3.921 | ENSG00000140400.17_9 | 3.655 | ENSG00000250548.7_9 | -4.182 |
| ENSG00000024526.17_6 | -4.603 | ENSG00000162591.16_5 | -3.955 | ENSG00000123989.14_6 | 3.934 | ENSG00000198846.6_6 | 2.659 | ENSG00000103460.17_5 | 5.170 | ENSG00000175063.17_4 | -5.292 | ENSG00000140416.23_9 | -3.372 | ENSG00000250644.3_8 | 12.172 |
| ENSG00000026025.16_7 | -3.493 | ENSG00000162736.17_7 | 3.828 | ENSG00000124107.5_5 | 8.226 | ENSG00000198855.7_4 | 2.699 | ENSG00000103507.14_7 | 2.752 | ENSG00000175155.10_6 | 4.252 | ENSG00000140451.13_5 | -3.728 | ENSG00000250722.6_9 | 4.050 |
| ENSG00000029153.14_7 | -2.580 | ENSG00000162755.14_5 | 2.897 | ENSG00000124155.18_8 | 3.735 | ENSG00000198894.8_8 | -4.815 | ENSG00000103528.17_5 | 3.730 | ENSG00000175213.3_4 | 3.095 | ENSG00000140465.14_6 | 3.705 | ENSG00000250838.1_8 | -3.106 |
| ENSG00000030110.13_5 | 3.364 | ENSG00000162804.14_6 | 3.216 | ENSG00000124299.15_6 | 3.691 | ENSG00000198901.14_7 | -2.992 | ENSG00000103995.14_8 | -2.933 | ENSG00000175224.16_7 | 2.855 | ENSG00000140474.14_6 | 4.055 | ENSG00000251095.7_8 | 3.786 |
| ENSG00000030582.18_5 | 5.647 | ENSG00000162814.11_4 | -2.498 | ENSG00000124575.7_4 | -4.344 | ENSG00000198917.13_8 | 9.903 | ENSG00000104147.9_5 | -3.943 | ENSG00000175274.18_8 | 3.390 | ENSG00000140479.17_6 | 4.699 | ENSG00000251191.8_7 | 3.875 |
| ENSG00000032444.16_7 | 2.832 | ENSG00000163131.11_6 | 2.788 | ENSG00000124664.11_6 | 12.261 | ENSG00000198925.12_8 | 6.330 | ENSG00000104237.11_8 | -5.166 | ENSG00000175283.8_4 | 3.178 | ENSG00000140511.12_5 | -5.238 | ENSG00000251201.8_5 | 2.601 |
| ENSG00000033011.13_6 | 2.907 | ENSG00000163319.11_6 | -2.355 | ENSG00000124702.18_4 | 4.892 | ENSG00000198952.8_5 | 2.896 | ENSG00000104321.11_5 | -2.421 | ENSG00000175305.17_4 | -3.889 | ENSG00000140525.18_7 | -2.576 | ENSG00000251349.3_4 | -10.673 |
| ENSG00000033627.16_5 | 2.553 | ENSG00000163346.17_6 | 4.258 | ENSG00000124731.13_6 | -3.389 | ENSG00000198959.12_5 | -2.408 | ENSG00000104447.12_6 | 2.464 | ENSG00000175567.10_9 | 2.464 | ENSG00000140534.14_5 | -3.041 | ENSG00000251537.4_8 | 3.914 |
| ENSG00000035499.13_5 | -2.416 | ENSG00000163364.10_8 | -2.617 | ENSG00000124787.14_6 | -2.491 | ENSG00000200879.1 | -3.198 | ENSG00000104490.18_8 | 3.426 | ENSG00000175592.9_6 | -3.416 | ENSG00000140688.17_6 | 11.659 | ENSG00000251648.2_6 | 6.373 |
| ENSG00000035664.11_7 | 2.524 | ENSG00000163507.14_7 | -2.819 | ENSG00000124882.4_4 | -4.662 | ENSG00000203485.13_5 | 2.547 | ENSG00000104522.16_6 | 2.748 | ENSG00000175634.15_6 | 3.239 | ENSG00000140859.16_7 | 3.049 | ENSG00000253368.4_6 | -2.856 |
| ENSG00000038295.8_6 | 4.116 | ENSG00000163516.14_5 | 2.795 | ENSG00000125122.15_7 | 4.259 | ENSG00000203647.2_6 | 5.218 | ENSG00000104537.17_8 | 3.325 | ENSG00000175643.10_5 | -5.255 | ENSG00000140961.14_6 | 3.042 | ENSG00000253559.1_6 | -3.123 |
| ENSG00000038358.15_5 | 3.078 | ENSG00000163517.15_6 | 3.526 | ENSG00000125148.7_4 | -3.094 | ENSG00000203709.12_9 | 3.064 | ENSG00000104679.11_9 | 2.388 | ENSG00000175832.13_6 | 2.528 | ENSG00000140983.14_5 | 4.351 | ENSG00000253796.2_8 | 2.872 |
| ENSG00000039068.19_7 | 3.414 | ENSG00000163521.16_6 | 2.510 | ENSG00000125149.12_7 | 2.658 | ENSG00000203760.8_2 | -4.424 | ENSG00000104738.18_8 | -2.737 | ENSG00000175874.10_5 | -6.565 | ENSG00000141002.20_6 | 2.424 | ENSG00000253954.3_5 | -2.457 |
| ENSG00000039560.14_7 | -2.694 | ENSG00000163535.18_7 | -4.017 | ENSG00000125304.10_6 | 3.034 | ENSG00000203761.5_6 | 3.231 | ENSG00000104760.17_6 | 5.338 | ENSG00000176046.9_4 | 2.673 | ENSG00000141012.13_6 | 2.709 | ENSG00000254017.1_3 | 2.918 |
| ENSG00000040275.17_8 | -3.043 | ENSG00000163631.17_9 | 10.823 | ENSG00000125449.7_4 | 3.911 | ENSG00000203879.12_4 | 2.601 | ENSG00000104805.16_8 | 5.121 | ENSG00000176108.9_6 | 4.275 | ENSG00000141295.14_7 | 3.816 | ENSG00000254285.3_4 | 2.771 |
| ENSG00000041353.10_6 | -3.821 | ENSG00000163703.19_5 | 3.464 | ENSG00000125459.17_6 | 2.726 | ENSG00000203993.6_9 | 3.627 | ENSG00000104835.15_5 | 3.537 | ENSG00000176153.12_4 | 4.612 | ENSG00000141338.14_8 | 2.687 | ENSG00000254692.1_9 | -15.385 |
| ENSG00000041988.15_6 | 2.564 | ENSG00000163735.7_5 | -3.865 | ENSG00000125538.12_8 | 2.818 | ENSG00000203999.9_7 | 3.046 | ENSG00000104853.16_7 | 3.113 | ENSG00000176208.9_4 | -2.967 | ENSG00000141349.8_3 | 4.429 | ENSG00000254858.10_4 | 2.933 |
| ENSG00000042980.13_4 | 3.176 | ENSG00000163808.17_6 | -4.737 | ENSG00000125637.16_7 | 4.685 | ENSG00000204128.6_4 | 11.450 | ENSG00000104870.13_8 | 4.276 | ENSG00000176387.7_4 | 11.027 | ENSG00000141469.18_7 | 2.556 | ENSG00000255036.6_9 | 10.486 |
| ENSG00000044090.10_4 | 2.650 | ENSG00000163814.8_6 | -3.976 | ENSG00000125652.8_4 | 2.376 | ENSG00000204231.10_4 | 3.122 | ENSG00000104883.8_6 | 4.100 | ENSG00000176473.13_5 | 2.603 | ENSG00000141570.11_6 | 4.103 | ENSG00000255071.3_8 | 10.586 |
| ENSG00000047457.14_6 | 5.815 | ENSG00000163870.16_8 | 3.234 | ENSG00000125734.15_9 | 4.156 | ENSG00000204257.15_6 | 3.935 | ENSG00000104886.12_5 | 3.049 | ENSG00000176771.17_9 | -4.742 | ENSG00000141580.16_6 | 2.571 | ENSG00000255198.4_5 | -3.113 |
| ENSG00000049192.15_6 | 3.005 | ENSG00000163918.10_7 | -2.367 | ENSG00000125743.10_4 | -2.610 | ENSG00000204308.8_4 | 2.754 | ENSG00000104907.12_6 | 3.029 | ENSG00000176890.16_4 | -4.759 | ENSG00000141736.13_6 | 2.512 | ENSG00000255339.6_10 | -2.435 |
| ENSG00000049239.13_4 | 2.587 | ENSG00000163930.10_5 | 2.650 | ENSG00000125753.14_4 | 3.056 | ENSG00000204310.13_6 | 2.499 | ENSG00000104946.13_6 | 3.506 | ENSG00000176912.5_8 | -2.497 | ENSG00000141756.19_8 | 3.818 | ENSG00000255408.4_5 | 4.280 |
| ENSG00000049323.16_6 | -2.684 | ENSG00000163945.18_7 | 10.241 | ENSG00000125780.12_5 | 12.146 | ENSG00000204348.10_6 | 2.983 | ENSG00000104957.14_6 | 2.774 | ENSG00000176978.14_6 | 3.313 | ENSG00000141934.10_7 | 3.666 | ENSG00000255508.7_8 | 4.565 |
| ENSG00000050344.9_4 | -3.222 | ENSG00000163947.11_8 | 2.410 | ENSG00000125841.13_5 | 4.013 | ENSG00000204382.7 | -2.569 | ENSG00000104960.15_3 | 2.513 | ENSG00000176986.16_5 | 2.733 | ENSG00000141959.17_3 | 3.297 | ENSG00000255641.1_7 | 11.237 |
| ENSG00000051180.17_5 | -4.329 | ENSG00000163956.13_7 | 3.047 | ENSG00000125845.7_5 | -2.942 | ENSG00000204382.7 | -2.569 | ENSG00000104969.10_6 | 3.490 | ENSG00000177042.14_5 | 2.440 | ENSG00000141971.13_6 | 2.508 | ENSG00000255737.2_5 | 5.257 |
| ENSG00000051341.14_6 | -4.755 | ENSG00000164010.15_9 | 2.618 | ENSG00000125954.12_10 | 12.989 | ENSG00000204386.11_6 | 2.959 | ENSG00000104980.8_6 | 2.669 | ENSG00000177106.16_8 | 2.555 | ENSG00000142102.16_10 | 3.077 | ENSG00000256028.2_7 | 3.476 |
| ENSG00000051523.11_6 | 5.617 | ENSG00000164032.12_6 | -3.057 | ENSG00000125967.16_5 | 2.594 | ENSG00000204387.14_7 | 2.432 | ENSG00000105063.19_8 | 3.013 | ENSG00000177181.15_6 | 10.211 | ENSG00000142185.16_8 | 3.829 | ENSG00000256061.7_10 | -2.509 |
| ENSG00000053747.16_5 | -3.470 | ENSG00000164047.6_5 | 10.839 | ENSG00000125999.11_5 | 7.372 | ENSG00000204388.7_6 | 2.641 | ENSG00000105135.16_5 | 2.502 | ENSG00000177239.15_7 | 4.151 | ENSG00000142197.12_5 | 2.723 | ENSG00000256092.2_8 | -3.193 |
| ENSG00000055044.11_4 | -3.413 | ENSG00000164050.13_5 | 3.186 | ENSG00000126351.13_3 | 4.656 | ENSG00000204394.13_7 | 2.677 | ENSG00000105223.20_5 | 7.558 | ENSG00000177359.20_8 | -2.723 | ENSG00000142327.13_7 | 3.581 | ENSG00000256268.2_7 | -11.008 |
| ENSG00000056291.17_4 | 4.099 | ENSG00000164054.15_6 | 3.813 | ENSG00000126709.15_5 | 3.182 | ENSG00000204427.12_6 | 7.277 | ENSG00000105229.7_4 | 3.568 | ENSG00000177595.18_6 | 2.824 | ENSG00000142530.11_7 | 3.461 | ENSG00000256500.6_7 | 10.690 |
| ENSG00000057294.15_7 | 3.793 | ENSG00000164056.11_6 | 3.550 | ENSG00000126768.12_4 | 2.793 | ENSG00000204525.16_4 | 2.938 | ENSG00000105289.15_5 | 7.392 | ENSG00000177628.16_6 | 2.636 | ENSG00000142552.8_6 | 11.638 | ENSG00000256806.6_5 | 3.716 |
| ENSG00000058056.9_5 | -2.390 | ENSG00000164062.13_5 | 3.871 | ENSG00000126787.13_6 | -4.914 | ENSG00000204564.12_6 | 3.087 | ENSG00000105388.16_5 | 10.421 | ENSG00000177674.16_7 | 3.023 | ENSG00000142731.11_5 | -3.583 | ENSG00000257017.4 | 13.615 |
| ENSG00000058262.10_4 | 3.119 | ENSG00000164081.12_8 | 2.363 | ENSG00000126903.16_6 | 2.492 | ENSG00000204682.8_7 | -2.569 | ENSG00000105402.8_7 | 4.332 | ENSG00000177707.11_7 | -2.757 | ENSG00000142856.16_4 | -2.355 | ENSG00000257315.2_5 | 4.614 |
| ENSG00000058673.16_7 | -3.164 | ENSG00000164087.8_7 | -2.955 | ENSG00000126934.14_6 | 3.098 | ENSG00000204843.12_7 | 2.530 | ENSG00000105404.11_6 | 3.145 | ENSG00000177830.18_8 | 3.018 | ENSG00000142864.14_6 | -2.358 | ENSG00000257379.1_7 | 4.324 |
| ENSG00000059122.16_3 | 3.093 | ENSG00000164106.8_5 | 3.456 | ENSG00000126953.8_7 | -3.095 | ENSG00000204852.16_6 | 4.059 | ENSG00000105520.10_4 | 10.679 | ENSG00000177951.17_4 | 3.013 | ENSG00000142945.13_6 | -3.399 | ENSG00000257588.1_8 | 2.618 |
| ENSG00000059573.9_5 | 2.499 | ENSG00000164109.14_5 | -4.176 | ENSG00000127324.9_5 | 4.262 | ENSG00000204965.9_6 | 5.073 | ENSG00000105568.18_6 | 3.092 | ENSG00000178105.11_6 | -3.481 | ENSG00000142973.14_5 | 11.709 | ENSG00000257594.4_7 | 2.652 |
| ENSG00000060642.11_7 | 2.799 | ENSG00000164125.15_7 | 11.676 | ENSG00000127325.19_7 | -4.572 | ENSG00000205236.6_7 | 11.109 | ENSG00000105612.9_5 | 3.221 | ENSG00000178397.13_10 | -7.757 | ENSG00000143093.15_5 | 3.221 | ENSG00000257732.1_7 | -10.171 |
| ENSG00000060656.20_5 | 2.588 | ENSG00000164188.8_6 | 10.072 | ENSG00000127329.15_7 | 2.699 | ENSG00000205336.13_8 | -3.882 | ENSG00000105726.17_5 | 2.813 | ENSG00000178467.18_5 | 2.576 | ENSG00000143127.13_4 | 3.104 | ENSG00000257831.1_7 | 3.414 |
| ENSG00000063180.9_5 | 3.599 | ENSG00000164199.18_10 | 3.749 | ENSG00000127419.17_9 | 2.774 | ENSG00000205403.13_5 | -2.663 | ENSG00000105732.13_6 | -2.883 | ENSG00000178498.16_7 | 2.790 | ENSG00000143178.13_5 | 2.659 | ENSG00000258017.2_8 | -3.194 |
| ENSG00000063587.15_5 | -2.492 | ENSG00000164236.12_4 | -4.140 | ENSG00000127423.11_5 | -3.966 | ENSG00000205426.10_5 | -2.451 | ENSG00000105855.10_4 | 5.334 | ENSG00000178502.6_5 | 10.264 | ENSG00000143224.18_7 | 2.871 | ENSG00000258122.1_8 | -4.297 |
| ENSG00000063587.15_5 | -2.492 | ENSG00000164266.10_4 | 3.437 | ENSG00000127452.9_9 | 3.316 | ENSG00000205560.13_5 | 4.564 | ENSG00000105894.12_5 | -4.501 | ENSG00000178538.10_5 | -2.790 | ENSG00000143228.13_6 | -4.906 | ENSG00000258181.1_9 | -3.061 |
| ENSG00000064199.6_2 | -2.678 | ENSG00000164509.15_5 | -5.729 | ENSG00000127837.9_4 | 3.854 | ENSG00000205595.3 | -2.957 | ENSG00000105974.12_6 | -3.710 | ENSG00000178764.8_5 | 4.801 | ENSG00000143303.12_7 | 2.488 | ENSG00000258429.2_6 | -10.299 |
| ENSG00000064545.15_9 | 5.768 | ENSG00000164520.11_5 | -3.885 | ENSG00000127948.16_6 | 2.981 | ENSG00000205643.11_6 | 2.420 | ENSG00000106003.13_5 | 2.579 | ENSG00000178921.14_9 | -2.545 | ENSG00000143368.10_7 | 3.516 | ENSG00000258488.2_6 | 2.946 |
| ENSG00000064601.19_5 | 3.520 | ENSG00000164611.13_5 | -5.270 | ENSG00000128274.17_6 | 5.205 | ENSG00000205809.9_6 | 4.978 | ENSG00000106066.15_6 | 2.496 | ENSG00000178927.18_9 | 2.489 | ENSG00000143374.17_5 | 2.583 | ENSG00000258555.6_4 | 4.532 |
| ENSG00000064651.14_5 | 4.125 | ENSG00000164683.18_6 | 4.823 | ENSG00000128309.16_3 | 2.452 | ENSG00000206341.7_4 | 3.064 | ENSG00000106268.15_6 | -3.032 | ENSG00000178977.3_5 | 3.304 | ENSG00000143401.15_9 | -2.602 | ENSG00000258628.1_6 | -6.037 |
| ENSG00000064687.13_4 | 2.703 | ENSG00000164687.11_4 | -2.509 | ENSG00000128408.9_5 | -4.510 | ENSG00000206561.13_9 | 10.839 | ENSG00000106290.15_8 | 3.788 | ENSG00000178980.15_8 | 2.665 | ENSG00000143409.15_8 | 2.677 | ENSG00000258634.3_7 | 2.834 |
| ENSG00000064961.19_5 | 2.520 | ENSG00000164741.15_11 | -10.482 | ENSG00000128510.12_6 | -5.119 | ENSG00000206712.1 | -2.700 | ENSG00000106348.18_9 | 3.190 | ENSG00000178999.13_7 | -4.712 | ENSG00000143416.21_8 | 4.815 | ENSG00000258643.5_6 | 3.198 |
| ENSG00000065029.15_6 | 2.429 | ENSG00000164880.16_5 | 4.545 | ENSG00000128590.5_4 | 2.424 | ENSG00000207088.1 | -2.498 | ENSG00000106351.13_5 | 4.041 | ENSG00000179008.9_6 | -3.097 | ENSG00000143418.19_7 | 2.417 | ENSG00000258653.3_8 | 3.363 |
| ENSG00000065054.14_6 | 3.679 | ENSG00000164896.20_6 | 4.370 | ENSG00000128944.13_7 | -2.424 | ENSG00000210156.1 | -2.418 | ENSG00000106399.11_5 | -2.494 | ENSG00000179046.8_4 | -2.851 | ENSG00000143443.10_4 | 2.858 | ENSG00000258655.2_7 | 3.386 |
| ENSG00000065268.11_4 | 5.277 | ENSG00000164916.11_5 | 3.387 | ENSG00000128965.13_6 | 3.633 | ENSG00000210176.1 | -2.761 | ENSG00000106462.11_6 | -3.474 | ENSG00000179083.6_3 | -3.324 | ENSG00000143476.18_4 | -3.044 | ENSG00000258727.1_6 | 4.273 |
| ENSG00000065325.13_5 | 3.706 | ENSG00000164976.9_6 | 2.577 | ENSG00000129103.18_5 | 2.827 | ENSG00000210184.1 | -3.202 | ENSG00000106537.8_4 | 3.484 | ENSG00000179104.9_4 | 5.588 | ENSG00000143494.15_6 | -3.452 | ENSG00000258780.1_4 | -2.882 |
| ENSG00000065328.16_4 | -5.324 | ENSG00000165006.14_5 | 3.065 | ENSG00000129195.16_7 | -3.021 | ENSG00000210191.1 | -3.503 | ENSG00000106628.10_7 | 2.550 | ENSG00000179115.11_5 | 2.492 | ENSG00000143512.13_5 | 5.258 | ENSG00000258790.1_6 | 2.420 |
| ENSG00000065833.9_5 | 2.415 | ENSG00000165046.12_4 | -3.498 | ENSG00000129245.12_5 | 2.772 | ENSG00000211445.12_9 | 5.622 | ENSG00000106723.17_4 | -2.369 | ENSG00000179163.11_4 | 2.649 | ENSG00000143537.13_4 | 4.095 | ENSG00000258818.4_5 | 3.860 |
| ENSG00000066230.12_8 | -4.175 | ENSG00000165061.15_6 | 3.552 | ENSG00000129255.16_5 | 3.433 | ENSG00000211448.13_8 | -4.022 | ENSG00000106780.9_5 | 2.589 | ENSG00000179218.14_3 | 2.777 | ENSG00000143570.18_7 | 2.732 | ENSG00000258881.6_8 | -2.640 |
| ENSG00000066279.18_6 | -6.071 | ENSG00000165091.18_5 | 3.257 | ENSG00000129295.9_7 | -2.475 | ENSG00000213024.12_7 | -2.649 | ENSG00000106789.13_5 | 4.248 | ENSG00000179222.18_5 | 3.329 | ENSG00000143761.16_5 | 2.813 | ENSG00000258984.5_3 | 4.061 |
| ENSG00000067221.14_8 | 2.550 | ENSG00000165102.15_6 | 2.614 | ENSG00000129514.8_6 | 2.732 | ENSG00000213030.5_3 | -10.323 | ENSG00000106927.12_5 | 4.868 | ENSG00000179242.16_7 | -4.648 | ENSG00000143933.18_7 | -3.037 | ENSG00000259024.6_5 | 2.433 |
| ENSG00000067248.10_6 | -2.700 | ENSG00000165272.16_8 | 12.798 | ENSG00000129521.15_6 | 5.075 | ENSG00000213066.13_6 | -2.490 | ENSG00000107175.12_5 | 2.730 | ENSG00000179262.10_5 | 3.950 | ENSG00000143977.14_5 | -2.663 | ENSG00000259040.5_4 | 12.484 |
| ENSG00000067798.16_6 | -2.438 | ENSG00000165282.14_7 | 2.982 | ENSG00000129680.16_7 | -2.384 | ENSG00000213204.8_6 | -2.850 | ENSG00000107551.21_8 | 2.416 | ENSG00000179364.13_7 | 3.812 | ENSG00000144057.15_5 | -2.660 | ENSG00000259112.2_5 | -2.524 |
| ENSG00000067992.15_8 | -2.781 | ENSG00000165304.8_5 | -3.695 | ENSG00000129810.15_8 | -5.052 | ENSG00000213390.11_8 | -2.732 | ENSG00000107829.15_7 | 2.895 | ENSG00000179526.17_5 | 3.242 | ENSG00000144063.4_4 | 2.621 | ENSG00000259171.1_8 | 15.667 |
| ENSG00000068001.14_8 | 2.430 | ENSG00000165376.11_5 | 4.700 | ENSG00000129925.11_6 | 3.403 | ENSG00000213406.3_2 | -2.944 | ENSG00000107872.12_5 | 2.951 | ENSG00000179532.13_7 | 2.434 | ENSG00000144063.4_4 | 2.621 | ENSG00000259207.9_5 | -3.166 |
| ENSG00000068383.19_5 | -3.104 | ENSG00000165458.14_8 | 2.987 | ENSG00000129933.21_5 | 2.862 | ENSG00000213614.10_8 | 3.026 | ENSG00000107902.14_4 | -2.408 | ENSG00000179542.16_5 | 3.345 | ENSG00000144158.4_5 | -2.559 | ENSG00000259345.7_9 | -4.280 |
| ENSG00000068489.13_6 | -5.514 | ENSG00000165480.16_5 | -4.878 | ENSG00000129991.13_7 | -2.916 | ENSG00000213672.8_9 | 3.024 | ENSG00000108262.15_6 | 2.694 | ENSG00000179674.4_5 | -4.568 | ENSG00000144354.14_5 | -2.660 | ENSG00000259439.2_8 | 2.562 |
| ENSG00000068903.20_7 | 4.268 | ENSG00000165490.13_6 | -3.649 | ENSG00000130175.9_7 | 4.443 | ENSG00000213676.13_8 | 4.487 | ENSG00000108479.12_7 | 3.061 | ENSG00000179750.16_5 | -5.072 | ENSG00000144455.14_6 | 2.827 | ENSG00000259768.6_9 | -2.744 |
| ENSG00000068971.14_5 | 3.631 | ENSG00000165502.7_6 | -2.438 | ENSG00000130193.8_4 | 2.405 | ENSG00000213683.4_5 | -2.442 | ENSG00000108602.18_6 | 3.063 | ENSG00000179913.11_5 | 10.598 | ENSG00000144455.14_6 | 2.827 | ENSG00000259781.1_4 | -3.250 |
| ENSG00000070413.20_7 | 3.262 | ENSG00000165525.18_5 | -2.507 | ENSG00000130270.16_4 | -3.449 | ENSG00000213707.2_4 | -3.941 | ENSG00000108639.7_6 | 6.090 | ENSG00000179914.5_5 | 11.051 | ENSG00000144554.10_3 | -2.794 | ENSG00000260007.3_8 | -2.873 |
| ENSG00000070526.15_6 | 10.187 | ENSG00000165795.23_7 | 11.912 | ENSG00000130305.17_5 | 2.489 | ENSG00000213809.9_6 | 12.758 | ENSG00000108679.13_6 | 4.894 | ENSG00000179950.14_6 | 2.412 | ENSG00000144560.15_8 | 2.710 | ENSG00000260586.2_8 | 3.982 |
| ENSG00000070540.13_7 | 2.840 | ENSG00000165821.12_9 | 10.211 | ENSG00000130313.7_5 | 2.675 | ENSG00000213809.9_6 | 12.758 | ENSG00000108784.10_5 | 2.904 | ENSG00000180616.9_6 | 4.318 | ENSG00000144677.15_5 | 2.457 | ENSG00000260711.2_8 | 5.551 |
| ENSG00000071054.16_8 | -2.612 | ENSG00000165915.14_4 | 3.257 | ENSG00000130396.20_8 | -2.490 | ENSG00000213918.10_6 | 2.729 | ENSG00000108840.15_6 | 3.251 | ENSG00000180730.5_4 | -3.141 | ENSG00000144749.13_4 | 3.053 | ENSG00000260756.1_6 | -6.083 |
| ENSG00000071073.13_6 | 3.697 | ENSG00000165929.13_6 | 6.274 | ENSG00000130513.6_5 | 3.938 | ENSG00000213928.9_5 | 4.980 | ENSG00000108846.16_4 | 2.804 | ENSG00000180901.11_4 | 2.472 | ENSG00000144891.17_3 | 11.612 | ENSG00000260886.2_7 | -3.247 |
| ENSG00000071553.17_6 | 5.231 | ENSG00000166164.15_7 | -3.267 | ENSG00000130522.6_6 | 3.175 | ENSG00000213983.12_6 | 4.584 | ENSG00000109065.12_8 | 3.809 | ENSG00000181381.13_5 | -2.434 | ENSG00000145029.14_5 | 3.123 | ENSG00000260927.2_7 | 3.318 |
| ENSG00000071575.11_3 | 3.355 | ENSG00000166169.17_8 | 2.559 | ENSG00000130635.16_7 | -3.244 | ENSG00000213995.11_4 | 2.890 | ENSG00000109066.14_4 | 3.344 | ENSG00000181523.13_7 | 3.484 | ENSG00000145220.14_7 | -2.445 | ENSG00000261061.1_7 | -2.860 |
| ENSG00000072071.16_7 | 2.745 | ENSG00000166313.19_8 | 3.507 | ENSG00000130707.18_4 | 3.434 | ENSG00000214063.11_5 | 6.025 | ENSG00000109072.14_7 | 4.319 | ENSG00000181544.15_7 | -3.591 | ENSG00000145386.10_5 | -2.978 | ENSG00000261408.7_7 | 3.539 |
| ENSG00000072201.14_7 | 3.257 | ENSG00000166333.14_5 | 2.657 | ENSG00000130714.17_5 | 3.122 | ENSG00000214160.10_5 | 2.740 | ENSG00000109089.7_3 | 6.361 | ENSG00000181625.17_6 | 2.902 | ENSG00000145703.16_6 | 2.713 | ENSG00000261474.1_7 | 3.377 |
| ENSG00000072571.20_7 | -4.785 | ENSG00000166451.13_4 | -4.703 | ENSG00000130768.15_5 | 12.410 | ENSG00000214176.9_7 | 2.812 | ENSG00000109107.14_7 | 2.555 | ENSG00000181625.17_6 | 2.902 | ENSG00000145920.15_6 | 3.731 | ENSG00000261553.5_7 | -2.596 |
| ENSG00000072958.8_6 | 2.887 | ENSG00000166557.14_5 | 2.929 | ENSG00000130787.14_4 | 2.690 | ENSG00000214274.9_4 | 3.985 | ENSG00000109220.11_6 | 2.873 | ENSG00000181634.8_5 | 5.689 | ENSG00000146233.8_5 | 2.537 | ENSG00000261578.1_6 | 2.539 |
| ENSG00000073169.14_7 | 2.489 | ENSG00000166579.15_7 | 3.399 | ENSG00000130827.6_5 | 3.075 | ENSG00000214517.10_5 | 2.398 | ENSG00000109321.11_5 | -2.907 | ENSG00000181649.8_7 | 3.036 | ENSG00000146250.7_4 | 5.690 | ENSG00000261713.6_6 | 5.396 |
| ENSG00000073282.13_5 | 4.757 | ENSG00000166619.15_9 | 2.477 | ENSG00000130830.15_7 | -3.726 | ENSG00000214654.8_6 | -3.941 | ENSG00000109511.12_5 | -6.916 | ENSG00000181789.14_4 | 2.551 | ENSG00000146263.11_5 | -2.903 | ENSG00000261771.5_8 | 11.206 |
| ENSG00000073350.14_5 | 2.459 | ENSG00000166670.10_5 | -5.059 | ENSG00000131067.17_5 | 3.436 | ENSG00000214655.10_7 | 2.942 | ENSG00000109586.12_6 | 2.894 | ENSG00000181830.9_7 | 3.102 | ENSG00000146374.14_5 | -5.149 | ENSG00000261786.1_6 | -2.398 |
| ENSG00000073578.17_6 | 2.569 | ENSG00000166736.11_5 | 10.264 | ENSG00000131080.15_6 | 2.498 | ENSG00000214706.10_5 | 3.274 | ENSG00000109674.4_4 | -5.361 | ENSG00000182087.14_9 | 3.768 | ENSG00000146410.12_9 | -3.962 | ENSG00000262304.2_8 | 10.701 |
| ENSG00000073849.15_5 | 2.463 | ENSG00000166803.13_7 | -5.057 | ENSG00000131153.9_6 | -5.283 | ENSG00000214900.11_8 | -3.710 | ENSG00000109736.15_6 | 6.384 | ENSG00000182095.14_7 | 2.632 | ENSG00000146540.15_7 | 3.095 | ENSG00000262454.4_5 | -2.596 |
| ENSG00000074416.14_10 | 2.667 | ENSG00000166828.3_4 | 11.308 | ENSG00000131165.15_5 | 2.674 | ENSG00000214900.11_8 | -3.710 | ENSG00000109738.11_4 | 2.405 | ENSG00000182118.8_6 | -2.452 | ENSG00000146670.10_6 | -3.468 | ENSG00000262814.8_5 | 3.041 |
| ENSG00000074842.8_7 | 4.337 | ENSG00000166845.15_8 | -3.751 | ENSG00000131389.17_6 | 2.520 | ENSG00000215018.10_7 | 5.183 | ENSG00000109805.10_4 | -5.304 | ENSG00000182195.9_6 | 3.076 | ENSG00000146830.10_5 | 4.497 | ENSG00000263080.1_8 | -6.222 |
| ENSG00000075131.10_8 | -2.858 | ENSG00000166888.12_7 | 2.387 | ENSG00000131408.15_10 | 7.728 | ENSG00000215032.2_4 | -3.238 | ENSG00000109819.9_5 | 4.557 | ENSG00000182481.9_5 | -2.588 | ENSG00000146859.6_4 | 2.963 | ENSG00000263155.6_8 | 3.999 |
| ENSG00000075142.14_5 | -2.398 | ENSG00000166920.12_4 | -13.150 | ENSG00000131470.14_4 | -2.890 | ENSG00000215033.3 | -4.396 | ENSG00000109906.14_7 | 3.786 | ENSG00000182580.3_4 | 10.524 | ENSG00000147027.4_5 | 3.427 | ENSG00000263639.7_5 | 10.610 |
| ENSG00000075218.19_5 | -4.982 | ENSG00000166922.8_3 | -4.313 | ENSG00000131669.10_5 | 2.538 | ENSG00000215182.6 | 12.460 | ENSG00000110011.13_4 | 3.721 | ENSG00000182704.8_6 | 2.621 | ENSG00000147642.17_9 | 4.513 | ENSG00000263809.1_8 | -4.408 |
| ENSG00000075234.17_6 | 2.536 | ENSG00000166923.12_5 | -7.699 | ENSG00000131747.15_6 | -6.019 | ENSG00000215482.3_5 | -2.614 | ENSG00000110013.13_5 | 4.395 | ENSG00000182718.17_9 | -2.621 | ENSG00000147697.9_5 | 4.320 | ENSG00000264350.1_4 | -2.739 |
| ENSG00000075426.12_5 | 4.978 | ENSG00000167034.10_5 | -3.086 | ENSG00000131748.16_8 | 4.854 | ENSG00000215788.10_9 | 4.073 | ENSG00000110025.13_5 | -6.284 | ENSG00000182858.14_8 | 4.099 | ENSG00000147813.16_7 | 4.786 | ENSG00000264391.1 | -2.868 |
| ENSG00000076356.7_5 | 4.467 | ENSG00000167118.11_4 | 2.981 | ENSG00000131781.13_6 | 4.802 | ENSG00000215837.7_2 | 10.080 | ENSG00000110042.8_4 | 2.755 | ENSG00000182871.16_6 | 10.316 | ENSG00000148053.16_4 | 4.197 | ENSG00000265190.6_4 | -2.841 |
| ENSG00000076382.17_8 | -2.631 | ENSG00000167130.18_6 | 2.964 | ENSG00000131797.12_5 | 3.039 | ENSG00000216490.4_5 | 2.418 | ENSG00000110080.18_7 | 2.811 | ENSG00000182934.12_6 | 2.701 | ENSG00000148180.19_6 | 2.486 | ENSG00000265972.6_5 | 5.176 |
| ENSG00000076604.15_5 | 2.498 | ENSG00000167394.13_5 | 10.257 | ENSG00000131873.7_6 | -2.994 | ENSG00000216775.3_6 | 3.047 | ENSG00000110104.13_6 | 2.544 | ENSG00000183010.17_8 | 4.759 | ENSG00000148344.11_5 | -2.788 | ENSG00000266088.6_8 | -3.399 |
| ENSG00000076864.19_8 | 4.429 | ENSG00000167491.17_6 | 2.421 | ENSG00000131979.19_6 | 2.452 | ENSG00000217555.13_5 | -2.612 | ENSG00000110171.20_7 | 3.771 | ENSG00000183023.18_6 | -4.265 | ENSG00000148346.12_4 | 3.001 | ENSG00000266278.1_6 | -3.284 |
| ENSG00000076928.18_4 | 3.117 | ENSG00000167508.12_6 | 3.363 | ENSG00000132004.13_5 | 3.806 | ENSG00000217801.10_7 | -3.379 | ENSG00000110328.6_6 | 2.824 | ENSG00000183048.12_5 | 2.867 | ENSG00000148356.13_4 | 2.859 | ENSG00000266835.6_6 | 2.659 |
| ENSG00000076944.16_7 | 2.489 | ENSG00000167513.9_6 | -4.306 | ENSG00000132024.17_5 | 3.006 | ENSG00000219200.6 | 2.859 | ENSG00000110427.16_6 | -5.127 | ENSG00000183072.10_7 | 3.437 | ENSG00000148773.14_6 | -5.967 | ENSG00000266962.2_7 | 3.190 |
| ENSG00000077063.11_8 | 4.723 | ENSG00000167536.14_5 | 11.641 | ENSG00000132341.12_4 | -2.527 | ENSG00000219435.6_8 | -4.649 | ENSG00000110455.14_7 | 3.030 | ENSG00000183087.15_5 | 3.836 | ENSG00000148848.14_7 | -3.630 | ENSG00000267216.1_9 | 3.572 |
| ENSG00000077092.19_6 | -4.981 | ENSG00000167578.18_5 | 4.046 | ENSG00000132376.20_6 | 2.552 | ENSG00000220785.7_6 | 11.062 | ENSG00000110628.16_8 | 4.808 | ENSG00000183111.12_4 | 3.386 | ENSG00000149136.9_6 | -2.444 | ENSG00000267278.6_7 | 3.527 |
| ENSG00000077152.11_7 | -2.492 | ENSG00000167601.12_4 | -4.413 | ENSG00000132394.11_4 | 2.843 | ENSG00000221944.8_6 | -10.525 | ENSG00000110723.12_9 | 3.134 | ENSG00000183287.14_10 | -11.545 | ENSG00000149150.9_8 | 10.839 | ENSG00000267279.2_10 | -7.863 |
| ENSG00000077420.16_5 | -4.224 | ENSG00000167614.14_6 | 3.832 | ENSG00000132471.12_9 | 2.876 | ENSG00000221988.13_7 | 2.559 | ENSG00000110841.14_9 | -2.417 | ENSG00000183401.12_8 | 3.906 | ENSG00000149182.15_6 | 3.128 | ENSG00000267281.2_9 | 2.550 |
| ENSG00000077514.9_7 | -2.425 | ENSG00000167641.11_5 | -4.135 | ENSG00000132475.10_6 | -3.029 | ENSG00000222009.8_3 | -3.660 | ENSG00000110881.12_6 | 4.116 | ENSG00000183461.12 | -3.333 | ENSG00000149260.18_7 | 2.612 | ENSG00000267374.2_10 | -4.191 |
| ENSG00000078061.13_4 | 3.038 | ENSG00000167642.13_7 | 2.353 | ENSG00000132481.7_7 | 3.196 | ENSG00000222041.11_5 | -2.905 | ENSG00000110921.14_7 | 3.695 | ENSG00000183508.5_5 | 5.381 | ENSG00000149294.16_7 | 2.994 | ENSG00000267504.1_7 | 4.296 |
| ENSG00000078319.9_7 | -2.390 | ENSG00000167670.16_4 | -2.727 | ENSG00000132507.18_4 | 3.547 | ENSG00000223477.3 | -4.899 | ENSG00000111142.14_5 | -2.822 | ENSG00000183682.8_5 | 3.772 | ENSG00000149428.19_9 | 3.230 | ENSG00000267645.5_6 | 3.023 |
| ENSG00000078596.11_5 | 5.690 | ENSG00000167693.17_6 | -2.572 | ENSG00000132561.14_8 | 4.029 | ENSG00000223572.10_3 | 2.516 | ENSG00000111206.13_6 | -2.697 | ENSG00000183779.7_4 | 5.648 | ENSG00000149541.10_5 | 3.245 | ENSG00000267838.2_7 | 2.659 |
| ENSG00000078804.13_5 | 4.311 | ENSG00000167716.18_4 | 3.310 | ENSG00000132589.16_5 | 3.661 | ENSG00000223572.10_3 | 2.516 | ENSG00000111247.14_3 | -4.109 | ENSG00000183856.11_5 | -3.803 | ENSG00000149743.14_6 | 2.824 | ENSG00000268104.3_5 | 10.996 |
| ENSG00000078898.7_4 | 10.793 | ENSG00000167740.10_4 | 2.393 | ENSG00000132635.17_6 | 3.816 | ENSG00000223705.9_4 | 2.721 | ENSG00000111261.14_7 | 2.692 | ENSG00000184489.12_6 | 10.195 | ENSG00000149761.9_7 | 3.744 | ENSG00000268107.6_8 | 3.026 |
| ENSG00000078902.16_6 | 2.504 | ENSG00000167747.14_7 | 4.201 | ENSG00000132639.13_4 | -2.635 | ENSG00000223784.2_6 | -2.706 | ENSG00000111271.15_6 | 3.155 | ENSG00000184500.16_8 | 2.991 | ENSG00000149792.9_3 | 3.052 | ENSG00000268471.6_7 | 2.771 |
| ENSG00000079112.10_7 | 5.944 | ENSG00000167767.14_6 | -10.089 | ENSG00000132740.9_3 | 3.733 | ENSG00000224259.7_6 | 2.797 | ENSG00000111275.13_5 | 3.075 | ENSG00000184530.9_4 | 10.131 | ENSG00000149798.5_4 | 10.147 | ENSG00000268942.2_4 | -2.950 |
| ENSG00000079215.14_5 | -3.490 | ENSG00000167775.11_6 | 3.552 | ENSG00000132768.14_4 | 2.424 | ENSG00000224660.2_7 | 2.517 | ENSG00000111276.11_7 | 4.735 | ENSG00000184588.18_7 | 2.632 | ENSG00000149809.16_6 | 4.005 | ENSG00000269559.2_6 | -4.534 |
| ENSG00000079482.13_6 | 2.507 | ENSG00000167778.9_5 | 4.424 | ENSG00000132793.11_3 | 2.873 | ENSG00000224805.2_7 | -3.696 | ENSG00000111319.13_8 | 2.968 | ENSG00000184619.7_7 | 2.534 | ENSG00000149923.14_4 | 3.342 | ENSG00000269693.1_7 | 10.434 |
| ENSG00000079689.14_4 | 13.287 | ENSG00000167779.9_5 | -3.530 | ENSG00000132837.15_6 | 3.443 | ENSG00000224864.4_5 | -4.803 | ENSG00000111348.9_6 | -3.640 | ENSG00000184661.14_4 | -4.740 | ENSG00000149929.16_5 | -2.567 | ENSG00000269955.2_5 | 11.771 |
| ENSG00000080189.15_7 | 2.725 | ENSG00000167861.16_5 | 4.468 | ENSG00000132879.14_6 | 2.848 | ENSG00000224870.7_8 | 2.865 | ENSG00000111653.20_6 | 2.586 | ENSG00000184752.13_6 | -2.595 | ENSG00000149948.13_8 | -4.850 | ENSG00000270181.3_8 | 12.938 |
| ENSG00000080371.6_4 | 5.663 | ENSG00000167912.6_7 | 3.881 | ENSG00000132963.8_4 | -2.640 | ENSG00000225131.2_4 | -3.021 | ENSG00000111725.11_5 | 3.187 | ENSG00000184840.12_4 | 2.894 | ENSG00000150165.13 | -2.654 | ENSG00000270299.1_8 | -8.577 |
| ENSG00000080986.13_6 | -5.517 | ENSG00000167930.17_7 | 3.282 | ENSG00000132965.9_3 | -5.279 | ENSG00000225159.1_4 | -2.619 | ENSG00000111780.8_8 | -10.089 | ENSG00000185033.14_6 | 3.227 | ENSG00000150551.11_5 | -5.698 | ENSG00000270344.3_7 | 2.603 |
| ENSG00000081177.18_4 | 2.401 | ENSG00000167965.18_7 | 3.641 | ENSG00000132967.9_4 | -4.055 | ENSG00000225573.4_4 | -3.288 | ENSG00000111913.20_9 | 6.387 | ENSG00000185055.11_7 | -2.600 | ENSG00000150630.4_5 | -11.095 | ENSG00000270580.5_9 | 3.005 |
| ENSG00000081692.12_5 | 2.611 | ENSG00000167972.14_7 | 2.650 | ENSG00000133110.15_6 | 4.045 | ENSG00000225663.8_9 | 2.631 | ENSG00000112029.10_6 | -2.561 | ENSG00000185128.11 | 4.742 | ENSG00000150782.12_6 | -2.631 | ENSG00000270800.3_6 | -2.645 |
| ENSG00000082126.18_7 | -3.557 | ENSG00000167995.17_4 | -10.845 | ENSG00000133302.13_5 | -2.356 | ENSG00000225746.12_10 | -2.988 | ENSG00000112312.10_5 | -2.849 | ENSG00000185324.22_6 | 3.490 | ENSG00000151131.11_7 | -2.786 | ENSG00000271204.1_8 | 3.690 |
| ENSG00000082515.18_5 | -2.406 | ENSG00000168000.14_9 | 3.402 | ENSG00000133317.15_3 | -5.296 | ENSG00000226380.9_10 | 2.430 | ENSG00000112379.9_4 | 4.482 | ENSG00000185339.9_5 | 5.687 | ENSG00000151135.10_4 | 5.039 | ENSG00000271303.2_5 | 13.432 |
| ENSG00000083444.17_5 | 3.283 | ENSG00000168036.18_9 | 2.442 | ENSG00000133321.11_5 | -2.640 | ENSG00000226471.6_8 | 2.819 | ENSG00000112473.18_6 | 3.075 | ENSG00000185359.13_6 | 4.063 | ENSG00000151388.11_7 | -10.970 | ENSG00000271741.1_7 | 10.568 |
| ENSG00000083838.16_5 | 4.141 | ENSG00000168078.10_5 | -5.346 | ENSG00000133710.16_6 | 4.857 | ENSG00000226674.11_9 | -5.472 | ENSG00000112576.13_5 | 4.243 | ENSG00000185480.11_6 | -3.291 | ENSG00000151466.12_8 | -2.568 | ENSG00000272221.1_7 | 2.579 |
| ENSG00000084636.18_7 | 2.767 | ENSG00000168096.15_8 | 4.177 | ENSG00000133884.10_5 | 2.770 | ENSG00000226953.8_7 | -6.745 | ENSG00000112742.10_5 | -4.130 | ENSG00000185499.16_7 | 2.814 | ENSG00000151617.17_7 | 5.494 | ENSG00000272430.1_8 | -2.740 |
| ENSG00000084652.16_4 | 3.147 | ENSG00000168101.14_6 | 2.761 | ENSG00000134057.15_5 | -4.946 | ENSG00000227051.6_4 | 3.380 | ENSG00000112773.16_10 | 2.847 | ENSG00000185567.7_5 | -2.391 | ENSG00000151632.17_6 | 2.691 | ENSG00000272647.3_6 | -11.217 |
| ENSG00000085117.12_6 | 3.571 | ENSG00000168209.5_5 | 2.751 | ENSG00000134258.12 | 10.195 | ENSG00000227640.3_6 | -4.452 | ENSG00000112852.7_5 | 3.451 | ENSG00000185624.15_5 | 3.489 | ENSG00000151725.12_9 | -4.658 | ENSG00000272990.1_6 | 2.436 |
| ENSG00000085840.13_5 | -2.922 | ENSG00000168243.11_6 | -3.232 | ENSG00000134285.11_7 | 2.449 | ENSG00000228063.2_9 | -2.941 | ENSG00000112902.12_7 | 4.024 | ENSG00000185630.19_6 | -2.636 | ENSG00000151729.11_4 | 2.639 | ENSG00000273003.1_3 | 11.742 |
| ENSG00000085998.14_5 | 3.256 | ENSG00000168306.13_5 | -2.834 | ENSG00000134339.9_7 | 3.925 | ENSG00000228192.8_8 | -3.185 | ENSG00000112984.12_5 | -3.888 | ENSG00000185697.16_6 | -6.157 | ENSG00000151790.9_4 | -3.358 | ENSG00000273045.6_6 | 3.176 |
| ENSG00000086544.3_4 | 3.047 | ENSG00000168398.6_4 | 3.017 | ENSG00000134531.10_6 | -4.046 | ENSG00000228434.1_8 | 3.127 | ENSG00000113108.19_4 | 2.539 | ENSG00000185760.15_6 | -2.920 | ENSG00000151835.16_7 | -2.532 | ENSG00000273079.5_7 | -3.683 |
| ENSG00000086548.9_6 | 6.081 | ENSG00000168418.7_4 | -10.504 | ENSG00000134569.10_6 | 2.917 | ENSG00000228612.2_5 | -10.834 | ENSG00000113119.12_3 | 2.694 | ENSG00000185862.7_6 | -2.752 | ENSG00000151849.15_5 | -2.730 | ENSG00000273154.3_9 | 4.123 |
| ENSG00000087074.8_5 | 2.699 | ENSG00000168453.15_6 | 4.665 | ENSG00000134575.13_7 | 3.846 | ENSG00000228649.9_8 | -3.276 | ENSG00000113249.13_6 | -2.382 | ENSG00000185909.15_4 | 3.206 | ENSG00000152049.6_3 | 4.073 | ENSG00000273162.1_6 | -4.017 |
| ENSG00000087076.9_6 | 2.681 | ENSG00000168591.16_6 | 2.682 | ENSG00000134690.11_4 | -2.517 | ENSG00000228716.7_6 | -4.167 | ENSG00000113368.12_4 | -3.986 | ENSG00000186111.10_5 | 3.773 | ENSG00000152127.9_5 | 2.407 | ENSG00000274049.4_3 | 12.172 |
| ENSG00000087245.13_7 | 3.682 | ENSG00000168675.18_6 | 2.522 | ENSG00000134824.14_6 | 2.391 | ENSG00000228742.11_8 | -10.347 | ENSG00000113460.13_4 | -3.044 | ENSG00000186185.14_5 | -6.649 | ENSG00000152137.7_4 | 2.678 | ENSG00000274070.2_4 | 3.168 |
| ENSG00000087448.11_5 | -7.018 | ENSG00000168676.11_6 | 4.799 | ENSG00000134827.8_5 | 4.244 | ENSG00000229097.1_4 | -3.049 | ENSG00000113532.13_5 | 3.948 | ENSG00000186193.9_5 | -2.494 | ENSG00000152217.19_8 | 2.732 | ENSG00000274561.1_6 | 3.054 |
| ENSG00000087586.17_4 | -3.225 | ENSG00000168685.15_6 | -11.524 | ENSG00000134853.12_7 | 3.769 | ENSG00000229308.1_6 | -5.188 | ENSG00000113716.13_5 | 2.983 | ENSG00000186198.4_4 | 3.798 | ENSG00000152253.9_6 | -6.347 | ENSG00000274750.2_4 | 2.607 |
| ENSG00000088298.13_5 | 2.557 | ENSG00000168748.14_4 | -4.654 | ENSG00000134899.22_7 | -2.440 | ENSG00000229344.1_4 | -2.417 | ENSG00000113916.18_6 | 5.265 | ENSG00000186204.15_6 | 4.452 | ENSG00000152527.14_7 | 3.454 | ENSG00000274810.4_5 | -11.286 |
| ENSG00000088298.13_5 | 2.557 | ENSG00000168763.16_4 | -3.142 | ENSG00000134910.14_7 | 2.365 | ENSG00000229425.3_8 | 4.504 | ENSG00000113924.12_6 | 4.538 | ENSG00000186377.8_4 | 2.744 | ENSG00000152580.8_4 | 3.803 | ENSG00000275395.6_7 | 7.667 |
| ENSG00000088325.16_4 | -3.964 | ENSG00000168874.13_4 | 3.060 | ENSG00000135046.14_5 | -3.887 | ENSG00000229425.3_8 | 4.504 | ENSG00000114395.10_4 | 2.374 | ENSG00000186469.8_5 | -3.931 | ENSG00000152939.17_8 | 4.233 | ENSG00000276043.5_7 | -4.023 |
| ENSG00000088340.17_7 | 4.952 | ENSG00000168890.14_7 | 3.265 | ENSG00000135074.16_6 | -4.921 | ENSG00000229656.7_7 | -3.494 | ENSG00000114405.10_3 | -2.463 | ENSG00000186635.14_6 | 3.415 | ENSG00000153246.13_6 | 3.690 | ENSG00000276368.2_4 | -4.751 |
| ENSG00000088836.14_10 | 4.483 | ENSG00000168936.11_6 | 3.118 | ENSG00000135124.14_4 | 3.603 | ENSG00000230330.1_5 | -4.031 | ENSG00000114544.16_6 | 2.759 | ENSG00000186807.9 | -2.898 | ENSG00000154222.14_5 | 3.578 | ENSG00000277215.2_8 | -4.189 |
| ENSG00000089163.4_3 | 4.626 | ENSG00000169116.11_4 | 5.493 | ENSG00000135127.11_6 | 7.274 | ENSG00000230453.9_3 | -3.704 | ENSG00000114626.18_6 | 2.553 | ENSG00000186871.7_7 | -6.130 | ENSG00000154269.15_5 | 5.078 | ENSG00000277726.4_8 | 2.527 |
| ENSG00000089199.10_7 | 4.292 | ENSG00000169169.14_7 | -4.782 | ENSG00000135324.6_4 | 3.554 | ENSG00000230562.3_4 | -2.482 | ENSG00000114650.20_8 | 2.605 | ENSG00000187051.9_5 | 2.516 | ENSG00000154545.16_4 | 2.620 | ENSG00000278023.7_7 | -4.058 |
| ENSG00000089248.7_5 | 3.212 | ENSG00000169213.7_5 | -2.837 | ENSG00000135373.12_5 | 3.593 | ENSG00000230789.1_6 | 2.636 | ENSG00000114779.19_7 | 2.439 | ENSG00000187091.13_5 | 4.200 | ENSG00000154556.18_9 | 2.468 | ENSG00000278238.1_6 | -2.437 |
| ENSG00000089597.18_6 | 3.517 | ENSG00000169220.18_4 | 10.301 | ENSG00000135443.8_4 | -3.420 | ENSG00000231131.8_8 | -6.717 | ENSG00000114786.16_8 | 2.836 | ENSG00000187105.9_5 | 3.005 | ENSG00000154723.12_6 | -2.395 | ENSG00000278619.5_4 | 3.255 |
| ENSG00000089685.15_7 | -5.674 | ENSG00000169223.15_7 | 3.406 | ENSG00000135476.12_5 | -4.986 | ENSG00000231160.10_8 | 3.547 | ENSG00000114812.13_4 | 3.498 | ENSG00000187134.14_5 | 3.070 | ENSG00000154839.10_5 | -5.097 | ENSG00000278949.1_6 | 4.729 |
| ENSG00000089693.10_5 | 4.284 | ENSG00000169231.13_5 | 3.146 | ENSG00000135480.16_5 | -3.025 | ENSG00000231298.7_8 | -4.971 | ENSG00000114993.17_7 | 3.804 | ENSG00000187189.11_5 | 5.769 | ENSG00000155093.19_7 | 3.693 | ENSG00000278991.1_6 | -2.430 |
| ENSG00000090432.7_5 | 2.958 | ENSG00000169507.9_7 | 3.150 | ENSG00000135506.16_7 | 2.500 | ENSG00000231609.7_8 | 2.631 | ENSG00000115053.16_6 | -2.500 | ENSG00000187243.16_9 | 3.402 | ENSG00000155256.17_5 | 2.502 | ENSG00000279110.1_7 | 3.051 |
| ENSG00000090581.10_5 | 3.674 | ENSG00000169554.21_8 | -3.061 | ENSG00000135617.4_5 | 3.004 | ENSG00000231903.1_9 | -5.176 | ENSG00000115109.14_4 | 2.693 | ENSG00000187244.12_5 | 3.008 | ENSG00000155660.11_8 | 2.514 | ENSG00000279253.1_6 | 4.760 |
| ENSG00000090889.12_4 | -5.656 | ENSG00000169607.13_5 | -4.873 | ENSG00000135643.5_4 | 3.632 | ENSG00000231991.4_3 | -2.671 | ENSG00000115163.15_5 | -2.751 | ENSG00000187510.9_8 | 10.803 | ENSG00000155792.10_4 | 3.015 | ENSG00000279673.1_6 | -2.453 |
| ENSG00000091583.11_6 | 2.793 | ENSG00000169679.15_8 | -3.999 | ENSG00000135821.19_6 | 4.871 | ENSG00000232300.1_6 | 3.384 | ENSG00000115216.14_5 | 2.996 | ENSG00000187609.16_6 | 3.752 | ENSG00000155961.5_5 | 2.756 | ENSG00000280191.3_6 | -10.158 |
| ENSG00000092470.12_5 | -3.251 | ENSG00000169682.18_6 | 3.600 | ENSG00000135905.19_6 | -3.004 | ENSG00000232611.1_8 | 4.108 | ENSG00000115255.11_7 | 5.312 | ENSG00000187689.10_5 | -10.171 | ENSG00000156298.12_4 | 2.837 | ENSG00000280219.1_6 | 3.456 |
| ENSG00000092621.12_5 | 2.523 | ENSG00000169692.13_5 | 4.908 | ENSG00000135916.16_7 | 5.349 | ENSG00000233384.3_9 | -3.656 | ENSG00000115275.14_8 | 3.542 | ENSG00000187720.14_6 | 2.597 | ENSG00000156345.17_4 | 2.797 | ENSG00000280537.2_7 | -11.184 |
| ENSG00000092853.14_6 | -5.300 | ENSG00000169727.12_6 | 3.712 | ENSG00000136098.17_6 | -2.501 | ENSG00000233559.2_6 | 2.640 | ENSG00000115307.17_5 | 2.557 | ENSG00000187736.12_5 | 5.241 | ENSG00000156802.13_6 | -2.995 | ENSG00000281649.2_6 | 2.592 |
| ENSG00000093009.11_4 | -4.249 | ENSG00000169891.18_4 | 3.440 | ENSG00000136108.15_8 | -2.666 | ENSG00000233578.1_4 | 2.706 | ENSG00000115339.14_6 | 3.283 | ENSG00000187838.17_8 | 6.528 | ENSG00000156873.16_7 | 2.416 | ENSG00000282034.1_10 | 11.071 |
| ENSG00000093010.14_6 | 4.512 | ENSG00000170004.17_8 | 2.484 | ENSG00000136122.18_6 | -2.794 | ENSG00000233750.3_5 | -4.676 | ENSG00000115380.20_6 | -4.624 | ENSG00000187908.19_7 | -4.249 | ENSG00000156875.14_5 | 2.505 | ENSG00000282057.1_10 | -3.260 |
| ENSG00000094804.12_7 | -3.048 | ENSG00000170099.6_6 | 4.868 | ENSG00000136156.15_7 | 2.446 | ENSG00000234327.8_8 | -2.538 | ENSG00000115414.20_8 | 2.591 | ENSG00000187951.11_6 | -2.422 | ENSG00000156876.10_6 | -2.581 | ENSG00000282304.1_8 | 5.222 |
| ENSG00000095303.17_8 | 3.877 | ENSG00000170190.16_7 | 10.499 | ENSG00000136231.14_5 | -2.618 | ENSG00000234460.2_6 | -5.926 | ENSG00000115423.19_5 | -2.473 | ENSG00000187954.12_6 | 3.081 | ENSG00000156966.7_4 | 3.121 | ENSG00000283239.1_3 | -11.821 |
| ENSG00000095321.17_9 | 3.132 | ENSG00000170231.16_3 | -2.937 | ENSG00000136807.14_7 | 2.628 | ENSG00000234494.7_9 | -2.602 | ENSG00000115457.10_7 | 11.260 | ENSG00000188107.15_6 | 12.260 | ENSG00000156970.13_5 | -3.036 | ENSG00000283515.1_8 | -9.348 |
| ENSG00000095906.17_6 | 3.217 | ENSG00000170266.16_6 | 3.676 | ENSG00000136824.19_5 | -2.974 | ENSG00000234494.7_9 | -2.602 | ENSG00000115525.18_8 | -2.888 | ENSG00000188242.4_9 | 11.576 | ENSG00000157216.15_7 | 2.945 | ENSG00000284057.1_4 | 11.687 |
| ENSG00000096006.11_4 | 11.178 | ENSG00000170312.16_5 | -4.378 | ENSG00000136828.19_6 | 2.524 | ENSG00000234664.1_3 | -3.774 | ENSG00000115556.14_6 | 2.839 | ENSG00000188375.5_6 | -3.312 | ENSG00000157240.4_4 | 3.347 | ENSG00000284194.3_7 | 3.112 |
| ENSG00000099139.14_7 | 3.293 | ENSG00000170345.10_5 | 3.278 | ENSG00000136840.19_6 | 4.206 | ENSG00000234705.2_9 | -3.374 | ENSG00000115806.13_4 | 2.893 | ENSG00000188549.12_8 | 4.383 | ENSG00000157388.19_9 | 6.552 | ENSG00000284308.1_6 | 2.822 |
| ENSG00000099203.7_6 | 3.760 | ENSG00000170373.8_4 | 13.389 | ENSG00000136856.18_6 | 2.741 | ENSG00000234753.5_8 | -4.258 | ENSG00000115963.13_4 | -2.603 | ENSG00000188603.20_8 | 3.964 | ENSG00000157456.8_5 | -4.705 | ENSG00000284762.1_3 | 10.249 |
| ENSG00000099219.14_5 | 3.654 | ENSG00000170412.18_9 | 3.014 | ENSG00000136867.11_4 | 3.984 | ENSG00000234779.1_6 | -10.535 | ENSG00000116035.4_7 | 5.312 | ENSG00000188735.13_4 | 3.452 | ENSG00000157500.12_6 | -2.559 | ENSG00000284776.1_4 | -10.851 |
| ENSG00000099282.10_5 | 3.690 | ENSG00000170421.12_8 | 3.275 | ENSG00000136877.15_6 | 3.430 | ENSG00000235043.3_4 | 2.981 | ENSG00000116128.11_6 | 3.456 | ENSG00000188959.9_4 | 3.628 | ENSG00000157514.16_8 | 3.155 | ENSG00000284946.1_3 | -4.372 |
| ENSG00000099377.14_6 | 2.654 | ENSG00000170523.3_5 | -3.090 | ENSG00000136942.15_5 | -3.394 | ENSG00000235257.9_10 | 4.326 | ENSG00000116133.13_6 | 2.987 | ENSG00000188985.6_3 | -5.059 | ENSG00000157593.19_6 | 3.874 | ENSG00000284969.1_5 | -2.642 |
| ENSG00000099797.15_6 | 2.778 | ENSG00000170703.15_4 | 4.609 | ENSG00000136943.11_5 | -2.494 | ENSG00000235306.2_5 | -11.038 | ENSG00000116260.17_4 | 3.681 | ENSG00000189057.11_7 | -9.302 | ENSG00000157613.11_7 | 5.734 | ENSG00000284981.1_2 | 5.445 |
| ENSG00000099817.12_5 | 3.110 | ENSG00000170860.4_4 | -2.644 | ENSG00000136999.5_4 | 3.040 | ENSG00000236345.1_7 | 6.935 | ENSG00000116584.20_8 | 2.537 | ENSG00000189058.9_7 | 6.637 | ENSG00000157637.13_7 | 2.387 | ENSG00000285077.2_4 | -3.016 |
| ENSG00000099849.15_6 | 4.492 | ENSG00000170899.11_6 | 3.230 | ENSG00000137070.18_6 | 5.285 | ENSG00000236603.2_2 | -2.609 | ENSG00000116691.11_4 | 4.118 | ENSG00000189077.11_6 | 2.801 | ENSG00000157654.19_7 | -3.184 | ENSG00000285253.1_3 | -3.501 |
| ENSG00000099901.17_8 | -2.652 | ENSG00000170961.7_5 | -4.748 | ENSG00000137103.20_8 | 2.772 | ENSG00000236682.2_8 | -2.913 | ENSG00000116786.13_6 | 3.178 | ENSG00000189114.8_7 | -8.999 | ENSG00000157833.13_5 | 3.044 | ENSG00000285367.1_3 | -3.275 |
| ENSG00000099956.20_6 | 2.371 | ENSG00000171097.14_8 | 6.514 | ENSG00000137171.15_6 | 2.701 | ENSG00000236751.2_8 | -4.304 | ENSG00000116830.12_4 | -2.591 | ENSG00000189143.9_4 | 2.838 | ENSG00000157881.16_8 | 11.836 | ENSG00000285517.1_4 | -2.400 |
| ENSG00000100023.19_5 | 2.401 | ENSG00000171130.18_6 | 2.397 | ENSG00000137266.14_6 | 3.377 | ENSG00000236901.6_5 | 2.707 | ENSG00000116857.17_6 | 3.214 | ENSG00000189221.9_2 | 2.507 | ENSG00000157978.12_4 | 3.046 | ENSG00000285517.1_4 | -2.400 |
| ENSG00000100031.19_7 | 4.310 | ENSG00000171159.5_4 | 2.825 | ENSG00000137267.6_5 | 2.434 | ENSG00000237082.1_5 | 3.769 | ENSG00000116863.11_4 | 2.608 | ENSG00000189319.14_6 | 2.856 | ENSG00000158055.16_3 | 2.715 | ENSG00000285730.1_3 | -3.005 |
| ENSG00000100056.12_8 | 3.426 | ENSG00000171163.16_6 | 4.268 | ENSG00000137285.10_5 | 2.555 | ENSG00000237289.10_3 | 3.374 | ENSG00000117151.13_6 | 3.091 | ENSG00000189339.12_5 | -2.572 | ENSG00000158106.14_5 | 2.567 | ENSG00000285802.1_3 | -5.517 |
| ENSG00000100097.12_4 | -3.721 | ENSG00000171206.14_4 | 2.465 | ENSG00000137434.11_6 | -2.884 | ENSG00000237289.10_3 | 3.374 | ENSG00000117228.10_5 | -2.654 | ENSG00000189403.15_6 | -3.616 | ENSG00000158290.18_7 | -2.715 | ENSG00000285816.1_3 | 3.044 |
| ENSG00000100116.16_6 | 2.733 | ENSG00000171208.10_6 | -2.508 | ENSG00000137460.8_3 | 3.467 | ENSG00000237523.2_6 | -2.426 | ENSG00000117298.16_7 | 2.723 | ENSG00000196154.12_6 | 3.914 | ENSG00000158402.20_10 | -5.328 | ENSG00000285827.1_1 | -6.505 |
| ENSG00000100162.15_5 | -3.365 | ENSG00000171222.10_4 | 2.899 | ENSG00000137486.17_9 | 2.856 | ENSG00000237543.1 | -10.224 | ENSG00000117362.13_7 | 2.851 | ENSG00000196155.13_6 | 3.067 | ENSG00000158773.14_6 | 10.627 | ENSG00000285839.1_2 | 4.232 |
| ENSG00000100219.16_8 | 3.994 | ENSG00000171241.9_6 | -3.825 | ENSG00000137672.13_6 | -2.696 | ENSG00000237883.2_9 | -2.798 | ENSG00000117399.14_4 | -4.493 | ENSG00000196187.12_7 | 2.520 | ENSG00000158828.8_5 | 2.810 | ENSG00000285841.1_3 | -10.236 |
| ENSG00000100243.21_6 | 4.618 | ENSG00000171298.13_6 | 4.518 | ENSG00000137700.18_10 | 3.866 | ENSG00000237991.3_4 | -3.088 | ENSG00000117410.14_5 | 2.431 | ENSG00000196517.13_8 | 5.143 | ENSG00000159069.14_7 | 5.284 | ENSG00000286075.1_3 | 10.685 |
| ENSG00000100263.14_6 | 2.914 | ENSG00000171320.15_5 | -3.884 | ENSG00000137710.17_7 | -2.531 | ENSG00000238000.1_5 | -2.824 | ENSG00000117472.10_4 | 4.218 | ENSG00000196535.16_9 | 2.431 | ENSG00000159167.12_5 | 2.836 | ENSG00000286185.1_2 | -10.075 |
| ENSG00000100284.21_7 | 5.188 | ENSG00000171346.16_7 | -2.579 | ENSG00000137752.24_8 | -3.391 | ENSG00000238266.2_5 | -7.226 | ENSG00000117632.23_6 | -3.648 | ENSG00000196549.11_6 | 11.138 | ENSG00000159184.8_4 | 5.572 | ENSG00000286388.1_1 | 3.442 |
| ENSG00000100299.18_5 | 4.589 | ENSG00000171443.7_4 | 4.227 | ENSG00000137801.11_5 | -2.957 | ENSG00000239382.10_8 | 3.698 | ENSG00000117643.14_4 | 11.570 | ENSG00000196550.10_3 | -2.635 | ENSG00000159199.14_6 | -2.570 | ENSG00000286587.1_1 | -2.468 |
| ENSG00000100300.18_5 | 2.714 | ENSG00000171462.15_7 | 3.333 | ENSG00000137804.14_8 | -5.099 | ENSG00000240065.8_5 | -3.533 | ENSG00000117650.13_5 | -5.347 | ENSG00000196576.15_6 | 3.481 | ENSG00000159214.13_5 | 3.198 | ENSG00000287001.1_2 | -4.364 |
| ENSG00000100359.21_5 | 7.064 | ENSG00000171552.13_7 | 3.597 | ENSG00000137807.15_8 | -2.877 | ENSG00000240476.1_6 | -6.601 | ENSG00000117724.13_4 | -6.354 | ENSG00000196584.3_5 | -2.406 | ENSG00000159259.8_5 | -2.591 | ENSG00000287179.1_1 | 11.023 |
| ENSG00000100439.10_3 | 3.707 | ENSG00000171557.17_7 | 3.192 | ENSG00000137812.20_8 | -4.969 | ENSG00000240898.1_5 | 3.176 | ENSG00000117983.17_6 | 8.516 | ENSG00000196611.5_5 | -10.405 | ENSG00000159708.18_5 | 8.005 | ENSG00000287287.1_2 | -9.320 |
| ENSG00000100445.17_8 | 3.027 | ENSG00000171560.16_6 | 3.992 | ENSG00000137868.19_7 | 2.644 | ENSG00000241388.5_6 | 2.809 | ENSG00000117984.14_7 | 7.302 | ENSG00000196639.6_4 | 3.093 | ENSG00000159713.11_5 | 7.946 | ENSG00000287625.1_1 | -4.249 |
| ENSG00000100461.18_7 | 2.898 | ENSG00000171564.11_3 | 2.808 | ENSG00000137878.17_6 | 5.127 | ENSG00000241399.7_6 | 2.844 | ENSG00000118162.14_5 | 2.652 | ENSG00000196656.7_4 | -3.204 | ENSG00000159763.4_4 | 5.938 | ENSG00000287650.1_2 | 12.562 |
| ENSG00000100479.13_5 | -3.141 | ENSG00000171848.15_6 | -4.964 | ENSG00000137962.13_5 | 3.000 | ENSG00000241489.8_9 | 11.871 | ENSG00000118193.12_5 | -4.946 | ENSG00000196668.4_4 | -4.226 | ENSG00000159788.19_9 | 2.527 | ENSG00000287916.1_1 | -14.577 |
| ENSG00000100490.9_4 | -2.623 | ENSG00000171877.21_8 | -4.109 | ENSG00000137962.13_5 | 3.000 | ENSG00000241945.8_4 | 6.742 | ENSG00000118402.6_5 | 2.985 | ENSG00000196811.13_6 | 2.703 | ENSG00000159884.12_6 | 3.539 | ENSG00000288558.1_2 | -10.060 |
| ENSG00000100504.17_5 | -2.754 | ENSG00000172006.12_4 | 2.702 | ENSG00000137965.11_4 | -4.075 | ENSG00000242147.2_9 | -7.463 | ENSG00000118523.6_5 | -3.167 | ENSG00000196998.19_9 | 2.398 | ENSG00000160058.18_4 | 2.589 | ENSG00000288568.1_1 | 3.682 |
| ENSG00000100526.20_6 | -4.900 | ENSG00000172037.14_5 | 2.932 | ENSG00000138092.11_5 | -2.675 | ENSG00000242147.2_9 | -7.463 | ENSG00000118705.17_5 | 2.387 | ENSG00000197061.5_4 | -3.056 |  |  |  |  |
| ENSG00000100558.9_6 | -2.715 | ENSG00000172046.19_7 | 3.052 | ENSG00000138160.6_4 | -4.316 | ENSG00000242265.5_5 | -2.801 |  |  |  |  |  |  |  |  |

| **GO_Biological Process_results** | | | | | | | | | | | |
| --- | --- | --- | --- | --- | --- | --- | --- | --- | --- | --- | --- |
| **ID** | **Description** | **Gene**  **Ratio** | **BgRatio** | **Rich**  **Factor** | **Fold**  **Enrichment** | **zScore** | **pvalue** | **p.adjust** | **qvalue** | **geneID** | **Count** |
| GO:0009100 | glycoprotein metabolic process | 63/930 | 377/18860 | 0.167 | 3.389 | 10.671 | 1.04E-17 | 5.28E-14 | 4.76E-14 | HS3ST1/ST3GAL1/ALG1/ABCA7/MGAT4A/ST6GAL1/ITM2A/SLC35H1/POMGNT1/EDEM2/MMP24-AS1-EDEM2/GANAB/GNPTG/CST3/PORCN/NAGLU/GALNT7/ST3GAL4/GALNT18/ST8SIA4/MOGS/GALNT3/MAN1C1/RPN2/CHPF/TM9SF2/NECAB3/MPDU1/PRKCSH/POMT1/FBXO44/STT3A/ITM2C/ITM2B/ST6GALNAC4/BMPR1B/SLC39A8/GCNT3/PCSK6/B3GAT3/EDNRA/MGAT5/B3GNT7/SLC35B2/NCSTN/HGSNAT/DOLPP1/CTNNB1/GLB1/GALNTL6/RAB1B/DOLK/MAN1B1/TMTC2/B3GNT3/SGSH/ALG12/SLC51B/RNF5/HEXA/ALG3/DDOST/GALNT4 | 63 |
| GO:0009101 | glycoprotein biosynthetic process | 49/930 | 303/18860 | 0.162 | 3.280 | 9.110 | 1.75E-13 | 4.44E-10 | 4.00E-10 | HS3ST1/ST3GAL1/ALG1/ABCA7/MGAT4A/ST6GAL1/ITM2A/SLC35H1/POMGNT1/GANAB/GNPTG/GALNT7/ST3GAL4/GALNT18/ST8SIA4/MOGS/GALNT3/MAN1C1/RPN2/CHPF/TM9SF2/NECAB3/MPDU1/PRKCSH/POMT1/STT3A/ITM2C/ITM2B/ST6GALNAC4/BMPR1B/SLC39A8/GCNT3/B3GAT3/MGAT5/B3GNT7/SLC35B2/NCSTN/DOLPP1/CTNNB1/GALNTL6/DOLK/TMTC2/B3GNT3/ALG12/SLC51B/HEXA/ALG3/DDOST/GALNT4 | 49 |
| GO:0070085 | glycosylation | 33/930 | 202/18860 | 0.163 | 3.313 | 7.527 | 1.23E-09 | 2.08E-06 | 1.88E-06 | ST3GAL1/ALG1/ST6GALNAC1/MGAT4A/ST6GAL1/SLC35H1/POMGNT1/COG4/LMF1/GALNT7/ST3GAL4/GALNT18/ST8SIA4/GALNT3/MAN1C1/RPN2/POMT1/STT3A/ST6GALNAC4/GCNT3/B3GAT3/MGAT5/B3GNT7/DOLPP1/GALNTL6/MAN1B1/TMTC2/B3GNT3/ALG12/SLC51B/ALG3/DDOST/GALNT4 | 33 |
| GO:0006486 | protein glycosylation | 31/930 | 187/18860 | 0.166 | 3.362 | 7.392 | 2.71E-09 | 2.75E-06 | 2.48E-06 | ST3GAL1/ALG1/ST6GALNAC1/MGAT4A/ST6GAL1/POMGNT1/LMF1/GALNT7/ST3GAL4/GALNT18/ST8SIA4/GALNT3/MAN1C1/RPN2/POMT1/STT3A/ST6GALNAC4/GCNT3/B3GAT3/MGAT5/B3GNT7/DOLPP1/GALNTL6/MAN1B1/TMTC2/B3GNT3/ALG12/SLC51B/ALG3/DDOST/GALNT4 | 31 |
| GO:0043413 | macromolecule glycosylation | 31/930 | 187/18860 | 0.166 | 3.362 | 7.392 | 2.71E-09 | 2.75E-06 | 2.48E-06 | ST3GAL1/ALG1/ST6GALNAC1/MGAT4A/ST6GAL1/POMGNT1/LMF1/GALNT7/ST3GAL4/GALNT18/ST8SIA4/GALNT3/MAN1C1/RPN2/POMT1/STT3A/ST6GALNAC4/GCNT3/B3GAT3/MGAT5/B3GNT7/DOLPP1/GALNTL6/MAN1B1/TMTC2/B3GNT3/ALG12/SLC51B/ALG3/DDOST/GALNT4 | 31 |
| GO:0006487 | protein N-linked glycosylation | 21/930 | 93/18860 | 0.226 | 4.579 | 7.880 | 3.59E-09 | 3.03E-06 | 2.73E-06 | ST3GAL1/ALG1/MGAT4A/ST6GAL1/GANAB/GNPTG/ST8SIA4/MOGS/MAN1C1/RPN2/MPDU1/PRKCSH/STT3A/SLC39A8/MGAT5/DOLPP1/DOLK/ALG12/HEXA/ALG3/DDOST | 21 |
| GO:0006664 | glycolipid metabolic process | 22/930 | 114/18860 | 0.193 | 3.914 | 7.106 | 3.31E-08 | 2.40E-05 | 2.16E-05 | PIGQ/ST3GAL1/PIGV/ITGB8/NAGLU/ST3GAL4/ST8SIA4/PIGT/TM9SF2/A4GALT/ST6GALNAC4/SUMF1/PGAP3/PIGO/GLB1/PIGG/DOLK/GBA1/FUCA1/GPAA1/NEU1/HEXA | 22 |
| GO:1903509 | liposaccharide metabolic process | 22/930 | 115/18860 | 0.191 | 3.880 | 7.054 | 3.91E-08 | 2.48E-05 | 2.23E-05 | PIGQ/ST3GAL1/PIGV/ITGB8/NAGLU/ST3GAL4/ST8SIA4/PIGT/TM9SF2/A4GALT/ST6GALNAC4/SUMF1/PGAP3/PIGO/GLB1/PIGG/DOLK/GBA1/FUCA1/GPAA1/NEU1/HEXA | 22 |
| GO:0007033 | vacuole organization | 36/930 | 270/18860 | 0.133 | 2.704 | 6.423 | 5.60E-08 | 3.15E-05 | 2.84E-05 | LAMP2/GRN/ATP6V0A1/WIPI1/ATP6AP1/TP53INP2/TOM1/NAGLU/TMEM9/ATP6V0B/CTSD/TMEM175/CHMP1A/ACP2/GABARAPL1/ULK3/WDR45B/LRSAM1/DEPTOR/PINK1/HID1/SPNS1/GAA/RAB1B/ATG13/NUPR1/CHMP6/GBA1/PACS2/CLN3/TMEM63A/WDR45/ATG9A/RNF5/HEXA/RNASEK | 36 |
| GO:0034976 | response to endoplasmic reticulum stress | 37/930 | 288/18860 | 0.128 | 2.605 | 6.252 | 9.70E-08 | 4.92E-05 | 4.43E-05 | RHBDD2/BAK1/ABCA7/PPP1R15A/EDEM2/MMP24-AS1-EDEM2/ERP29/ERMP1/XBP1/HM13/SGTA/CREB3/AUP1/GORASP2/MAN1C1/DNAJB9/CHAC1/NR1H2/FBXO44/OS9/RCN3/HYOU1/PDIA4/CREB3L1/ANKZF1/TMUB2/TMEM129/BCL2L1/USP19/NUPR1/MAN1B1/CALR/TMEM259/P4HB/FICD/RNF5/ATF6B | 37 |
| GO:0006979 | response to oxidative stress | 47/930 | 418/18860 | 0.112 | 2.280 | 6.028 | 1.20E-07 | 5.54E-05 | 5.00E-05 | PDK2/PPP5C/HGF/BAK1/TMEM161A/HYAL2/SIRT2/MMP2/SLC4A11/COMT/PTGS1/ERMP1/XBP1/DCAF11/HM13/AMBP/NAGLU/PPARGC1A/ADPRS/GGT7/GCH1/FADS2/PDGFRA/FBLN5/NEIL1/CBX8/TRPM2/NAPRT/LCN2/EDNRA/PLA2R1/PINK1/ANKZF1/RGS14/FOS/UCP2/GPX2/PYCR1/APOD/FCHSD1/MB/HSPA1B/GPX3/SELENOP/HP/TXNIP/SRXN1 | 47 |
| GO:0006643 | membrane lipid metabolic process | 31/930 | 229/18860 | 0.135 | 2.745 | 6.052 | 3.30E-07 | 0.000139657 | 0.000125932 | PIGQ/ST3GAL1/PIGV/TECR/PLA2G15/ITGB8/NAGLU/ST3GAL4/ST8SIA4/ELOVL4/PIGT/TM9SF2/A4GALT/SMPDL3B/ST6GALNAC4/NAAA/ABCA8/PLPP2/CERS2/SUMF1/PGAP3/PIGO/GLB1/PIGG/DOLK/GBA1/FUCA1/PSAP/GPAA1/NEU1/HEXA | 31 |
| GO:0062197 | cellular response to chemical stress | 39/930 | 335/18860 | 0.116 | 2.361 | 5.724 | 5.92E-07 | 0.000231004 | 0.000208301 | BAD/PDK2/NOX1/PPP5C/HGF/TMEM161A/SIRT2/MMP2/SLC4A11/ERMP1/XBP1/TSPO/HM13/AMBP/NAGLU/PPARGC1A/ARHGEF2/ADPRS/GDF15/NINJ1/GCH1/FADS2/PDGFRA/FBLN5/CBX8/TRPM2/LCN2/EDNRA/PLA2R1/PINK1/ANKZF1/MLST8/BDKRB2/FOS/PYCR1/CLN3/MB/HSPA1B/SRXN1 | 39 |
| GO:0036503 | ERAD pathway | 20/930 | 115/18860 | 0.174 | 3.527 | 6.190 | 8.01E-07 | 0.000290283 | 0.000261755 | RHBDD2/EDEM2/MMP24-AS1-EDEM2/XBP1/HM13/SGTA/AUP1/MAN1C1/DNAJB9/FBXO44/OS9/RCN3/ANKZF1/TMUB2/TMEM129/USP19/MAN1B1/CALR/TMEM259/RNF5 | 20 |
| GO:0016266 | O-glycan processing | 11/930 | 39/18860 | 0.282 | 5.720 | 6.720 | 1.86E-06 | 0.000613789 | 0.000553467 | ST3GAL1/ST6GAL1/POMGNT1/GALNT7/ST3GAL4/GALNT3/ST6GALNAC4/GCNT3/B3GNT7/B3GNT3/GALNT4 | 11 |

| **GO_Molecular Function_results** | | | | | | | | | | | |
| --- | --- | --- | --- | --- | --- | --- | --- | --- | --- | --- | --- |
| **ID** | **Description** | **Gene**  **Ratio** | **Bg**  **Ratio** | **Rich**  **Factor** | **Fold**  **Enrichment** | **zScore** | **pvalue** | **p.adjust** | **qvalue** | **geneID** | **Count** |
| GO:0004553 | hydrolase activity, hydrolyzing O-glycosyl compounds | 20/975 | 92/18662 | 0.2174 | 4.1610 | 7.1360 | 0.0000 | 0.0000 | 0.0000 | HYAL2/EDEM2/MMP24-AS1-EDEM2/GANAB/NAGLU/MOGS/ADPRS/CTBS/MAN1C1/MAN2C1/PGGHG/GLB1L/MYORG/GLB1/GAA/MAN1B1/GBA1/FUCA1/NEU1/HEXA | 20 |
| GO:0016798 | hydrolase activity, acting on glycosyl bonds | 23/975 | 131/18662 | 0.1756 | 3.3605 | 6.3656 | 0.0000 | 0.0001 | 0.0001 | HYAL2/EDEM2/MMP24-AS1-EDEM2/GANAB/MPG/NAGLU/MOGS/ADPRS/CTBS/MAN1C1/SMPDL3B/NEIL1/MAN2C1/PGGHG/GLB1L/MYORG/GLB1/GAA/MAN1B1/GBA1/FUCA1/NEU1/HEXA | 23 |
| GO:0016757 | glycosyltransferase activity | 34/975 | 267/18662 | 0.1273 | 2.4374 | 5.5541 | 0.0000 | 0.0005 | 0.0004 | PIGQ/ST3GAL1/ALG1/PIGV/SIRT2/ST6GALNAC1/MGAT4A/ST6GAL1/POMGNT1/SIRT4/LFNG/GALNT7/ST3GAL4/GALNT18/ST8SIA4/GALNT3/RPN2/CHPF/A4GALT/POMT1/STT3A/ST6GALNAC4/GCNT3/B3GAT3/MGAT5/B3GNT7/GALNTL6/GBA1/TMTC2/B3GNT3/ALG12/HEXA/ALG3/GALNT4 | 34 |
| GO:0030246 | carbohydrate binding | 35/975 | 282/18662 | 0.1241 | 2.3756 | 5.4649 | 0.0000 | 0.0005 | 0.0004 | PKD1/MRC2/CNTN1/H6PD/DGCR2/ADGRL1/PLOD1/POMGNT1/GANAB/AMBP/GALK1/VTN/GALNT7/GALNT18/GALNT3/EGLN3/OS9/SLC2A8/KLRG1/MAN2C1/PFKL/PLA2R1/ZG16B/LMAN2/GAA/GALNTL6/CHID1/P4HTM/CALR/ITLN1/KLRC2/KLRK1/KLRC4-KLRK1/CD302/GALNT4 | 35 |
| GO:0016758 | hexosyltransferase activity | 26/975 | 197/18662 | 0.1320 | 2.5262 | 5.0559 | 0.0000 | 0.0025 | 0.0023 | PIGQ/ALG1/PIGV/MGAT4A/POMGNT1/LFNG/GALNT7/GALNT18/GALNT3/RPN2/CHPF/A4GALT/POMT1/STT3A/GCNT3/B3GAT3/MGAT5/B3GNT7/GALNTL6/GBA1/TMTC2/B3GNT3/ALG12/HEXA/ALG3/GALNT4 | 26 |
| GO:0046915 | transition metal ion transmembrane transporter activity | 10/975 | 44/18662 | 0.2273 | 4.3501 | 5.2235 | 0.0001 | 0.0115 | 0.0107 | ATP13A1/SLC39A7/SLC31A2/SLC40A1/SLC39A8/SLC46A3/TRPM2/SLC39A1/SLC39A13/TTYH1 | 10 |
| GO:0016627 | oxidoreductase activity, acting on the CH-CH group of donors | 11/975 | 60/18662 | 0.1833 | 3.5091 | 4.5704 | 0.0002 | 0.0338 | 0.0314 | SDHA/CRAT/TECR/ACAD10/DHCR24/PPOX/TM7SF2/AKR1C2/DHCR7/AKR1C1/DECR2 | 11 |
| GO:0008375 | acetylglucosaminyltransferase activity | 9/975 | 44/18662 | 0.2045 | 3.9151 | 4.5452 | 0.0004 | 0.0460 | 0.0429 | PIGQ/MGAT4A/POMGNT1/LFNG/GCNT3/MGAT5/B3GNT7/B3GNT3/HEXA | 9 |

| **GO_KEGG enrichment result** | | | | |
| --- | --- | --- | --- | --- |
| **category** | **ID** | **term** | **adj_pval** | **genes** |
| KEGG | hsa04142 | Lysosome | 1.40E-09 | LAMP2, ATP6V0A1, CTSA, HYAL2, ATP6AP1, AP1M1, GNPTG, ARSA, LGMN, PLA2G15, DNASE2, NAGLU, ATP6V0B, CTSD, ACP2, GALNS, SUMF1, CTSS, HGSNAT, GLB1, GAA, CTSF, GBA1, FUCA1, SGSH, CLN3, PSAP, NEU1, HEXA, AP1G2, PPT2 |
| KEGG | hsa00510 | N-Glycan biosynthesis | 1.86E-05 | ALG1, MGAT4A, ST6GAL1, GANAB, MOGS, MAN1C1, RPN2, STT3A, MGAT5, DOLPP1, DOLK, MAN1B1, ALG12, ALG3, DDOST |
| KEGG | hsa04141 | Protein processing in endoplasmic reticulum | 7.78E-05 | CAPN1, BAK1, SEC61A1, PPP1R15A, EDEM2, MMP24-AS1-EDEM2, ERP29, GANAB, XBP1, MOGS, MAN1C1, RPN2, PRKCSH, STT3A, OS9, HYOU1, PDIA4, LMAN2, SEC24C, MAN1B1, CALR, RAD23A, P4HB, RNF5, HSPA1B, ATF6B, DDOST |
| KEGG | hsa00512 | Mucin type O-glycan biosynthesis | 0.001509288 | ST3GAL1, ST6GALNAC1, GALNT7, GALNT18, GALNT3, ST6GALNAC4, GCNT3, GALNTL6, B3GNT3, GALNT4 |
| KEGG | hsa00531 | Glycosaminoglycan degradation | 0.002988064 | HYAL2, NAGLU, GALNS, HGSNAT, GLB1, SGSH, HEXA |
| KEGG | hsa00513 | Various types of N-glycan biosynthesis | 0.004301365 | ALG1, MGAT4A, MAN1C1, RPN2, STT3A, MAN1B1, ALG12, HEXA, ALG3, DDOST |
| KEGG | hsa00511 | Other glycan degradation | 0.014409567 | MAN2C1, GLB1, GBA1, FUCA1, NEU1, HEXA |
