# Supplementary material for: ST6GAL1 promotes cancer stem-like cell-associated paclitaxel resistance through the EGFR-mTOR-SOX2/BMI1 axis in non-small cell lung cancer
Source: Cancer Drug Resist. 2026 Aug 31;9:32. doi: 10.20517/cdr.2026.66 (PMC13589886; doi:10.20517/cdr.2026.66)
Supplement: Supplementary file 3 [file cdr-9-32-Supplementaryfiles.zip › Supplementary File 4-GSVA Score.docx]

| **GSVA Score for N-glycosylation index and signalings** | | | | | | | | | | | | | | | | | | | | | | | | | | | | | |
| --- | --- | --- | --- | --- | --- | --- | --- | --- | --- | --- | --- | --- | --- | --- | --- | --- | --- | --- | --- | --- | --- | --- | --- | --- | --- | --- | --- | --- | --- |
| **lung cancer sample ID** | **Ngly** | **hsa01521** | **hsa02010** | **hsa04010** | **hsa04012** | **hsa04014** | **hsa04015** | **hsa04310** | **hsa04330** | **hsa04340** | **hsa04350** | **hsa04390** | **hsa04392** | **hsa04370** | **hsa04371** | **hsa04630** | **hsa04064** | **hsa04668** | **hsa04066** | **hsa04068** | **hsa04020** | **hsa04070** | **hsa04072** | **hsa04071** | **hsa04024** | **hsa04022** | **hsa04151** | **hsa04152** | **hsa04150** |
| TCGA.05.4249.01 | 0.154 | 0.38 | 0.06 | 0.15 | 0.25 | 0.17 | 0.11 | 0.12 | 0.36 | 0.31 | 0.10 | 0.05 | 0.06 | 0.14 | 0.15 | 0.11 | 0.17 | 0.07 | 0.08 | 0.03 | 0.09 | 0.34 | 0.19 | 0.30 | 0.10 | 0.24 | 0.03 | 0.27 | 0.23 |
| TCGA.05.4382.01 | 0.073 | 0.00 | 0.15 | -0.12 | 0.07 | -0.10 | -0.14 | 0.03 | 0.15 | 0.16 | 0.00 | -0.01 | 0.15 | 0.08 | -0.03 | -0.34 | -0.44 | -0.32 | -0.08 | 0.05 | -0.19 | 0.16 | 0.00 | 0.01 | -0.07 | -0.22 | -0.27 | 0.13 | 0.07 |
| TCGA.05.4384.01 | -0.406 | -0.19 | 0.34 | 0.05 | -0.10 | 0.18 | 0.17 | 0.15 | 0.22 | 0.23 | -0.15 | 0.02 | 0.34 | -0.05 | 0.13 | 0.02 | 0.02 | -0.16 | -0.10 | -0.07 | 0.20 | 0.21 | 0.20 | 0.15 | 0.26 | 0.19 | 0.00 | -0.03 | -0.20 |
| TCGA.05.4389.01 | -0.140 | -0.43 | 0.23 | -0.01 | -0.30 | 0.06 | 0.02 | -0.11 | -0.17 | -0.04 | -0.05 | -0.27 | 0.23 | -0.11 | -0.15 | 0.19 | 0.26 | -0.02 | -0.27 | -0.23 | 0.19 | -0.15 | -0.02 | -0.22 | 0.11 | -0.01 | -0.02 | -0.39 | -0.39 |
| TCGA.05.4390.01 | -0.146 | 0.26 | -0.28 | -0.02 | 0.09 | -0.05 | -0.02 | 0.28 | 0.05 | 0.42 | 0.19 | 0.22 | -0.28 | -0.02 | -0.08 | 0.13 | 0.28 | 0.09 | 0.17 | 0.01 | -0.13 | 0.15 | 0.05 | -0.01 | 0.09 | 0.09 | 0.05 | 0.09 | -0.12 |
| TCGA.05.4398.01 | -0.291 | -0.18 | -0.01 | -0.03 | 0.11 | -0.05 | -0.09 | -0.17 | -0.14 | 0.08 | -0.19 | -0.10 | -0.01 | -0.20 | -0.27 | -0.03 | -0.01 | -0.10 | -0.34 | -0.21 | -0.09 | 0.04 | -0.17 | -0.04 | -0.05 | -0.27 | -0.16 | -0.23 | -0.03 |
| TCGA.05.4402.01 | 0.554 | 0.38 | -0.31 | -0.02 | 0.09 | -0.01 | -0.03 | -0.01 | 0.07 | 0.13 | 0.12 | 0.10 | -0.31 | 0.05 | -0.12 | -0.10 | -0.20 | 0.02 | 0.15 | -0.01 | -0.27 | 0.06 | -0.03 | 0.17 | -0.22 | -0.16 | -0.08 | 0.12 | 0.22 |
| TCGA.05.4405.01 | 0.226 | 0.54 | 0.11 | 0.21 | 0.10 | 0.30 | 0.34 | 0.32 | 0.04 | 0.18 | 0.45 | 0.29 | 0.11 | 0.10 | 0.33 | 0.37 | 0.30 | 0.27 | 0.20 | 0.23 | 0.32 | -0.14 | 0.25 | 0.19 | 0.26 | 0.36 | 0.37 | -0.11 | -0.23 |
| TCGA.05.4415.01 | -0.525 | -0.35 | -0.26 | -0.15 | -0.18 | -0.16 | -0.25 | -0.21 | -0.20 | -0.31 | -0.27 | -0.21 | -0.26 | -0.11 | -0.26 | -0.34 | -0.33 | -0.33 | -0.17 | -0.29 | 0.00 | -0.16 | -0.13 | -0.29 | -0.22 | -0.21 | -0.21 | -0.28 | -0.16 |
| TCGA.05.4417.01 | -0.301 | -0.04 | -0.03 | -0.15 | 0.14 | -0.04 | -0.12 | 0.02 | 0.25 | 0.06 | -0.11 | -0.03 | -0.03 | 0.19 | -0.16 | -0.03 | -0.03 | 0.00 | -0.08 | -0.05 | -0.18 | 0.09 | 0.09 | -0.07 | -0.08 | -0.19 | -0.11 | 0.01 | -0.05 |
| TCGA.05.4420.01 | 0.649 | -0.09 | -0.21 | -0.21 | -0.18 | -0.19 | -0.25 | -0.23 | -0.16 | -0.27 | -0.17 | -0.20 | -0.21 | -0.04 | -0.13 | -0.39 | -0.37 | -0.26 | -0.16 | -0.07 | -0.27 | -0.19 | -0.17 | 0.01 | -0.16 | -0.10 | -0.18 | 0.05 | 0.27 |
| TCGA.05.4424.01 | 0.345 | 0.26 | 0.20 | 0.13 | 0.02 | 0.26 | 0.35 | 0.18 | 0.32 | 0.11 | 0.26 | 0.17 | 0.20 | -0.02 | 0.12 | 0.03 | 0.00 | -0.09 | 0.12 | 0.12 | 0.26 | 0.23 | 0.09 | 0.29 | 0.36 | 0.37 | 0.25 | 0.28 | 0.09 |
| TCGA.05.4427.01 | -0.520 | -0.31 | 0.10 | -0.11 | -0.11 | 0.10 | -0.01 | -0.25 | -0.16 | -0.03 | -0.24 | -0.32 | 0.10 | -0.19 | -0.01 | 0.09 | -0.10 | -0.32 | -0.15 | 0.00 | 0.06 | -0.05 | -0.04 | -0.29 | 0.04 | 0.03 | -0.11 | -0.20 | -0.20 |
| TCGA.05.4432.01 | 0.448 | 0.01 | -0.17 | -0.05 | -0.05 | -0.02 | 0.06 | -0.07 | 0.28 | 0.26 | 0.11 | 0.05 | -0.17 | -0.02 | 0.05 | -0.10 | 0.09 | -0.07 | -0.11 | 0.02 | -0.01 | 0.23 | 0.02 | 0.02 | 0.00 | 0.08 | -0.09 | 0.25 | 0.07 |
| TCGA.05.5425.01 | -0.423 | 0.31 | 0.14 | 0.30 | 0.22 | 0.33 | 0.36 | 0.26 | 0.25 | 0.39 | 0.27 | 0.26 | 0.14 | 0.19 | 0.34 | 0.35 | 0.07 | 0.16 | -0.08 | 0.28 | 0.38 | 0.36 | 0.36 | 0.25 | 0.40 | 0.43 | 0.15 | 0.18 | -0.15 |
| TCGA.05.5428.01 | 0.353 | 0.38 | -0.28 | 0.09 | 0.24 | 0.09 | 0.09 | 0.31 | 0.23 | 0.43 | 0.44 | 0.35 | -0.28 | 0.07 | 0.31 | 0.35 | 0.40 | 0.36 | 0.26 | 0.27 | 0.02 | 0.26 | 0.16 | 0.23 | 0.13 | 0.27 | 0.29 | 0.21 | 0.07 |
| TCGA.18.3406.01 | -0.390 | -0.22 | 0.01 | -0.06 | -0.17 | -0.02 | -0.04 | -0.08 | -0.02 | -0.11 | -0.20 | -0.27 | 0.01 | -0.19 | -0.14 | -0.04 | 0.07 | -0.11 | -0.06 | -0.28 | 0.11 | -0.11 | 0.04 | -0.24 | -0.03 | -0.11 | 0.01 | -0.16 | -0.06 |
| TCGA.18.3407.01 | 0.469 | -0.25 | 0.35 | -0.04 | 0.00 | -0.07 | -0.08 | 0.08 | 0.21 | 0.09 | -0.10 | -0.13 | 0.35 | -0.01 | -0.14 | -0.10 | -0.04 | -0.13 | -0.26 | -0.15 | -0.08 | -0.13 | -0.02 | -0.08 | -0.10 | -0.28 | -0.17 | -0.15 | -0.09 |
| TCGA.18.3408.01 | 0.088 | 0.23 | 0.28 | -0.12 | -0.04 | -0.01 | -0.07 | 0.04 | -0.32 | 0.12 | 0.16 | 0.08 | 0.28 | -0.21 | -0.04 | -0.04 | -0.27 | -0.21 | 0.02 | 0.07 | -0.03 | 0.05 | -0.03 | 0.09 | -0.10 | -0.01 | 0.00 | -0.02 | -0.07 |
| TCGA.18.3409.01 | -0.395 | 0.41 | 0.31 | 0.39 | 0.29 | 0.40 | 0.44 | 0.34 | 0.25 | 0.48 | 0.40 | 0.34 | 0.31 | 0.19 | 0.41 | 0.42 | 0.17 | 0.27 | 0.11 | 0.28 | 0.45 | 0.38 | 0.41 | 0.30 | 0.43 | 0.46 | 0.35 | 0.23 | -0.26 |
| TCGA.18.3410.01 | -0.217 | 0.14 | 0.50 | 0.11 | -0.12 | 0.18 | 0.20 | 0.16 | 0.00 | 0.26 | 0.21 | 0.12 | 0.50 | -0.09 | 0.11 | 0.24 | 0.27 | 0.12 | -0.11 | 0.12 | 0.26 | 0.22 | 0.13 | 0.14 | 0.20 | 0.33 | 0.22 | 0.07 | -0.24 |
| TCGA.18.3411.01 | 0.406 | 0.14 | -0.08 | -0.03 | 0.10 | -0.04 | 0.03 | -0.23 | -0.07 | -0.28 | -0.34 | -0.22 | -0.08 | 0.30 | -0.20 | 0.01 | 0.23 | 0.27 | 0.11 | 0.07 | -0.22 | 0.03 | -0.06 | 0.26 | -0.20 | -0.15 | -0.09 | 0.17 | 0.19 |
| TCGA.18.3412.01 | 0.021 | -0.21 | 0.00 | -0.11 | -0.16 | -0.13 | -0.14 | -0.05 | 0.14 | 0.24 | -0.01 | -0.10 | 0.00 | -0.30 | -0.12 | -0.30 | -0.17 | -0.16 | -0.14 | -0.07 | -0.11 | 0.03 | -0.07 | -0.08 | -0.02 | 0.12 | -0.14 | 0.01 | 0.16 |
| TCGA.18.3414.01 | -0.135 | 0.02 | -0.27 | -0.02 | -0.03 | -0.13 | -0.19 | 0.11 | -0.18 | -0.20 | 0.00 | -0.02 | -0.27 | 0.23 | -0.09 | -0.23 | -0.07 | 0.14 | -0.23 | 0.02 | -0.23 | -0.15 | -0.09 | 0.08 | -0.18 | -0.16 | -0.15 | 0.11 | 0.17 |
| TCGA.18.3415.01 | -0.207 | -0.18 | -0.20 | -0.18 | -0.11 | -0.21 | -0.12 | 0.07 | -0.16 | -0.03 | -0.02 | 0.17 | -0.20 | 0.00 | -0.16 | -0.33 | -0.31 | -0.34 | -0.03 | -0.08 | -0.16 | 0.00 | -0.18 | 0.00 | -0.17 | -0.14 | -0.14 | 0.14 | 0.20 |
| TCGA.18.3416.01 | -0.161 | -0.27 | 0.14 | 0.01 | -0.25 | -0.01 | 0.03 | -0.07 | -0.25 | -0.17 | -0.27 | -0.12 | 0.14 | -0.04 | -0.14 | 0.24 | 0.36 | 0.07 | -0.19 | -0.21 | 0.16 | -0.19 | 0.07 | -0.20 | 0.03 | -0.08 | -0.02 | -0.29 | -0.28 |
| TCGA.18.3417.01 | -0.161 | -0.50 | 0.19 | -0.22 | -0.32 | -0.11 | -0.23 | 0.05 | -0.15 | -0.09 | -0.11 | -0.07 | 0.19 | -0.34 | -0.30 | -0.14 | -0.04 | -0.15 | -0.25 | -0.22 | -0.04 | -0.22 | -0.22 | -0.36 | -0.04 | -0.05 | -0.18 | -0.19 | -0.32 |
| TCGA.18.3421.01 | -0.170 | -0.06 | -0.21 | 0.01 | 0.15 | -0.11 | -0.01 | -0.05 | -0.23 | -0.03 | -0.06 | -0.03 | -0.21 | 0.14 | 0.01 | -0.05 | 0.06 | 0.19 | 0.16 | -0.04 | -0.30 | -0.25 | 0.00 | 0.06 | -0.05 | -0.24 | -0.01 | 0.04 | 0.12 |
| TCGA.18.4083.01 | -0.324 | 0.04 | -0.07 | -0.21 | -0.19 | -0.20 | -0.28 | -0.09 | -0.21 | 0.16 | 0.07 | -0.02 | -0.07 | -0.10 | -0.06 | -0.27 | -0.32 | -0.38 | -0.08 | -0.07 | -0.15 | 0.01 | -0.19 | -0.30 | -0.13 | -0.23 | -0.19 | -0.13 | -0.07 |
| TCGA.18.4721.01 | 0.027 | -0.27 | -0.13 | -0.12 | 0.04 | -0.11 | -0.15 | -0.04 | 0.17 | -0.33 | -0.24 | -0.04 | -0.13 | 0.14 | -0.21 | -0.23 | 0.08 | 0.06 | -0.21 | -0.04 | -0.19 | -0.21 | -0.22 | -0.14 | -0.25 | -0.28 | -0.12 | -0.15 | -0.06 |
| TCGA.18.5592.01 | 0.101 | -0.31 | 0.29 | 0.18 | -0.19 | 0.20 | 0.08 | -0.20 | -0.30 | -0.04 | -0.06 | -0.24 | 0.29 | -0.04 | 0.14 | 0.33 | 0.37 | 0.30 | -0.08 | -0.04 | 0.23 | 0.04 | 0.17 | 0.19 | 0.06 | 0.21 | 0.02 | -0.18 | -0.01 |
| TCGA.21.1070.01 | -0.107 | 0.33 | 0.13 | 0.15 | 0.28 | 0.12 | 0.12 | -0.16 | -0.01 | 0.03 | -0.12 | 0.09 | 0.13 | 0.25 | 0.06 | 0.31 | 0.35 | 0.28 | 0.26 | 0.09 | 0.20 | 0.12 | 0.13 | 0.05 | 0.08 | -0.01 | 0.18 | -0.03 | -0.06 |
| TCGA.21.1071.01 | 0.150 | 0.13 | 0.19 | -0.03 | 0.21 | -0.11 | -0.06 | 0.03 | -0.02 | -0.07 | 0.00 | 0.11 | 0.19 | -0.05 | 0.03 | -0.04 | -0.08 | 0.01 | 0.07 | 0.09 | -0.19 | 0.04 | 0.07 | 0.10 | -0.14 | -0.06 | 0.01 | 0.07 | -0.10 |
| TCGA.21.1072.01 | -0.178 | -0.13 | 0.02 | 0.03 | -0.25 | 0.05 | 0.02 | -0.11 | -0.03 | -0.22 | -0.16 | -0.21 | 0.02 | 0.07 | -0.11 | 0.15 | 0.28 | -0.01 | 0.13 | -0.17 | 0.15 | 0.09 | -0.06 | -0.07 | -0.15 | 0.03 | 0.02 | -0.19 | -0.22 |
| TCGA.21.1075.01 | 0.054 | 0.21 | 0.04 | 0.15 | 0.06 | 0.14 | 0.14 | -0.04 | 0.13 | 0.01 | 0.02 | 0.11 | 0.04 | 0.15 | 0.07 | 0.39 | 0.49 | 0.36 | 0.20 | 0.15 | 0.16 | 0.14 | 0.11 | 0.05 | 0.05 | 0.07 | 0.23 | -0.20 | -0.14 |
| TCGA.21.1076.01 | -0.269 | -0.45 | -0.21 | -0.29 | -0.30 | -0.32 | -0.41 | -0.39 | -0.17 | -0.36 | -0.34 | -0.41 | -0.21 | -0.41 | -0.36 | -0.32 | -0.33 | -0.37 | -0.29 | -0.23 | -0.27 | -0.32 | -0.32 | -0.36 | -0.22 | -0.28 | -0.32 | -0.07 | 0.07 |
| TCGA.21.1077.01 | -0.064 | 0.29 | 0.10 | 0.34 | 0.19 | 0.31 | 0.38 | 0.27 | 0.31 | 0.44 | 0.29 | 0.32 | 0.10 | 0.09 | 0.37 | 0.16 | -0.07 | 0.15 | -0.03 | 0.23 | 0.34 | 0.33 | 0.33 | 0.24 | 0.36 | 0.45 | 0.17 | 0.22 | -0.05 |
| TCGA.21.1078.01 | 0.032 | -0.24 | 0.17 | -0.17 | -0.07 | -0.17 | -0.19 | 0.10 | -0.04 | 0.12 | 0.02 | 0.14 | 0.17 | -0.19 | -0.12 | -0.32 | -0.39 | -0.45 | -0.15 | -0.20 | 0.00 | -0.02 | -0.06 | -0.21 | -0.05 | -0.15 | -0.17 | -0.05 | -0.10 |
| TCGA.21.1079.01 | -0.375 | -0.11 | 0.30 | 0.16 | -0.06 | 0.20 | 0.13 | -0.09 | -0.06 | 0.16 | 0.04 | -0.15 | 0.30 | -0.04 | 0.22 | 0.33 | 0.34 | 0.16 | -0.07 | 0.12 | 0.28 | 0.22 | 0.18 | -0.02 | 0.25 | 0.22 | 0.04 | -0.09 | -0.37 |
| TCGA.21.1080.01 | -0.007 | -0.06 | -0.18 | -0.18 | -0.04 | -0.30 | -0.24 | -0.03 | 0.08 | -0.28 | -0.11 | -0.08 | -0.18 | -0.11 | -0.07 | -0.17 | -0.13 | -0.10 | -0.14 | 0.04 | -0.35 | -0.14 | -0.23 | -0.04 | -0.20 | -0.17 | -0.22 | 0.25 | 0.29 |
| TCGA.21.1081.01 | 0.013 | 0.24 | -0.39 | -0.03 | 0.18 | -0.17 | -0.04 | 0.02 | 0.24 | 0.04 | 0.10 | 0.08 | -0.39 | 0.26 | -0.15 | -0.30 | -0.21 | -0.07 | -0.08 | -0.09 | -0.31 | 0.00 | -0.02 | -0.02 | -0.15 | -0.15 | -0.09 | 0.17 | 0.34 |
| TCGA.21.1082.01 | -0.398 | -0.17 | 0.10 | 0.09 | -0.19 | 0.07 | 0.01 | 0.08 | -0.21 | 0.17 | 0.13 | 0.03 | 0.10 | 0.04 | -0.04 | 0.26 | 0.47 | 0.34 | 0.03 | 0.02 | 0.10 | -0.16 | 0.04 | -0.16 | 0.12 | -0.01 | 0.21 | -0.19 | -0.44 |
| TCGA.21.1083.01 | 0.257 | -0.30 | -0.24 | -0.16 | -0.08 | -0.22 | -0.15 | -0.23 | -0.34 | -0.50 | -0.15 | -0.15 | -0.24 | 0.04 | -0.12 | -0.15 | -0.13 | 0.03 | 0.14 | -0.02 | -0.31 | -0.31 | -0.27 | 0.04 | -0.24 | -0.26 | -0.22 | -0.06 | 0.26 |
| TCGA.21.5782.01 | 0.118 | -0.19 | 0.32 | -0.02 | 0.01 | 0.00 | 0.05 | 0.17 | -0.01 | -0.04 | 0.04 | 0.07 | 0.32 | -0.06 | -0.01 | -0.08 | -0.19 | -0.20 | 0.18 | -0.27 | 0.33 | -0.30 | 0.12 | -0.29 | 0.25 | 0.03 | 0.10 | -0.23 | -0.32 |
| TCGA.21.5783.01 | 0.099 | 0.35 | 0.34 | 0.17 | 0.16 | 0.19 | 0.16 | -0.05 | 0.12 | 0.22 | 0.04 | -0.05 | 0.34 | 0.01 | 0.18 | 0.13 | 0.21 | 0.14 | -0.16 | 0.05 | 0.12 | 0.43 | 0.19 | 0.16 | 0.11 | 0.24 | 0.05 | 0.04 | -0.05 |
| TCGA.21.5784.01 | 0.207 | -0.30 | -0.19 | -0.02 | -0.18 | 0.05 | 0.01 | 0.09 | -0.21 | -0.32 | -0.06 | 0.12 | -0.19 | 0.03 | 0.11 | 0.04 | 0.19 | 0.24 | -0.08 | -0.02 | 0.01 | -0.22 | -0.14 | 0.04 | 0.03 | -0.03 | 0.02 | -0.10 | -0.03 |
| TCGA.21.5786.01 | 0.566 | 0.53 | 0.02 | 0.23 | 0.25 | 0.09 | 0.18 | 0.31 | 0.27 | 0.03 | 0.33 | 0.43 | 0.02 | 0.23 | 0.09 | 0.30 | 0.43 | 0.46 | 0.22 | 0.22 | 0.08 | -0.06 | 0.23 | 0.23 | 0.24 | 0.06 | 0.28 | 0.05 | -0.20 |
| TCGA.21.5787.01 | -0.416 | -0.01 | -0.21 | -0.11 | 0.00 | -0.07 | -0.18 | 0.15 | 0.06 | -0.16 | -0.05 | 0.06 | -0.21 | -0.16 | -0.21 | -0.31 | -0.34 | -0.18 | -0.23 | -0.19 | -0.14 | -0.10 | -0.01 | -0.28 | -0.12 | -0.14 | -0.12 | -0.14 | -0.24 |
| TCGA.21.A5DI.01 | -0.193 | 0.19 | -0.16 | 0.28 | 0.13 | 0.21 | 0.25 | 0.04 | -0.01 | 0.09 | 0.10 | 0.11 | -0.16 | 0.29 | 0.16 | 0.27 | 0.30 | 0.29 | 0.09 | -0.04 | 0.14 | -0.07 | 0.16 | 0.19 | 0.24 | 0.16 | 0.23 | -0.09 | 0.00 |
| TCGA.22.0940.01 | -0.358 | -0.58 | -0.13 | -0.31 | -0.23 | -0.26 | -0.32 | -0.28 | -0.22 | -0.39 | -0.32 | -0.30 | -0.13 | -0.21 | -0.33 | -0.33 | -0.21 | -0.30 | -0.25 | -0.35 | -0.12 | -0.34 | -0.30 | -0.25 | -0.22 | -0.33 | -0.29 | -0.06 | 0.20 |
| TCGA.22.1000.01 | 0.006 | 0.03 | 0.20 | 0.12 | -0.01 | 0.23 | 0.15 | 0.08 | -0.06 | 0.09 | 0.17 | -0.05 | 0.20 | 0.02 | 0.18 | 0.35 | 0.26 | -0.04 | 0.06 | 0.05 | 0.24 | 0.19 | 0.18 | 0.10 | 0.12 | 0.08 | 0.14 | 0.00 | -0.22 |
| TCGA.22.1002.01 | 0.036 | -0.05 | 0.06 | -0.10 | 0.18 | -0.16 | -0.15 | 0.07 | 0.10 | -0.05 | -0.21 | 0.17 | 0.06 | 0.08 | -0.03 | -0.20 | -0.26 | 0.01 | 0.03 | -0.01 | -0.33 | 0.06 | -0.13 | -0.06 | -0.22 | -0.27 | -0.11 | 0.22 | 0.11 |
| TCGA.22.1005.01 | -0.343 | -0.50 | 0.29 | -0.08 | -0.33 | 0.04 | -0.03 | -0.12 | -0.22 | -0.20 | -0.12 | -0.32 | 0.29 | -0.15 | -0.07 | 0.23 | 0.33 | 0.10 | -0.25 | -0.21 | 0.26 | -0.24 | 0.00 | -0.28 | 0.13 | 0.07 | 0.04 | -0.40 | -0.44 |
| TCGA.22.1011.01 | -0.326 | -0.12 | 0.31 | 0.00 | 0.16 | -0.02 | -0.05 | -0.07 | 0.07 | 0.02 | -0.08 | -0.07 | 0.31 | 0.12 | 0.01 | 0.05 | 0.01 | 0.08 | -0.03 | -0.03 | 0.03 | 0.01 | 0.08 | -0.12 | 0.11 | 0.07 | -0.10 | 0.10 | 0.07 |
| TCGA.22.1016.01 | -0.095 | 0.53 | -0.19 | 0.04 | 0.34 | 0.10 | 0.01 | 0.23 | 0.05 | 0.33 | 0.33 | 0.17 | -0.19 | -0.02 | 0.30 | 0.12 | 0.01 | -0.07 | 0.26 | 0.34 | 0.03 | 0.28 | 0.09 | 0.20 | 0.16 | 0.28 | 0.19 | 0.36 | 0.21 |
| TCGA.22.1017.01 | -0.098 | 0.08 | -0.07 | 0.14 | 0.04 | 0.07 | 0.00 | 0.00 | 0.16 | -0.09 | 0.01 | 0.06 | -0.07 | -0.13 | 0.01 | 0.23 | 0.09 | 0.17 | 0.29 | 0.11 | 0.06 | 0.18 | -0.02 | 0.19 | 0.00 | -0.02 | 0.00 | 0.23 | 0.25 |
| TCGA.22.4591.01 | 0.534 | 0.29 | -0.22 | -0.14 | 0.15 | -0.14 | -0.09 | -0.10 | 0.00 | 0.09 | -0.12 | -0.03 | -0.22 | -0.03 | -0.06 | -0.27 | -0.39 | -0.19 | 0.19 | 0.10 | -0.30 | -0.05 | -0.16 | 0.03 | -0.11 | -0.15 | -0.17 | 0.24 | 0.29 |
| TCGA.22.4593.01 | 0.312 | 0.64 | -0.46 | 0.09 | 0.30 | 0.00 | 0.13 | 0.20 | 0.17 | 0.27 | 0.32 | 0.20 | -0.46 | 0.25 | 0.20 | -0.01 | -0.21 | 0.03 | 0.24 | 0.24 | 0.07 | 0.21 | 0.12 | 0.15 | 0.09 | 0.11 | 0.17 | 0.35 | 0.22 |
| TCGA.22.4595.01 | 0.428 | 0.18 | -0.28 | -0.14 | 0.28 | 0.03 | 0.06 | 0.10 | -0.09 | -0.13 | 0.12 | 0.22 | -0.28 | 0.21 | 0.13 | -0.16 | -0.11 | -0.21 | 0.12 | 0.04 | -0.12 | 0.24 | 0.14 | 0.25 | 0.04 | 0.08 | -0.04 | 0.17 | 0.27 |
| TCGA.22.4596.01 | 0.003 | -0.17 | -0.10 | -0.13 | 0.05 | -0.24 | -0.31 | 0.02 | -0.12 | 0.07 | 0.05 | 0.03 | -0.10 | -0.15 | -0.26 | -0.31 | -0.12 | -0.01 | 0.14 | -0.05 | -0.29 | -0.03 | -0.22 | -0.09 | -0.11 | -0.35 | -0.28 | -0.13 | -0.12 |
| TCGA.22.4604.01 | 0.262 | -0.12 | 0.16 | -0.03 | 0.24 | -0.06 | -0.08 | 0.06 | 0.13 | 0.24 | 0.04 | 0.15 | 0.16 | -0.02 | 0.00 | -0.18 | -0.37 | -0.18 | 0.11 | -0.08 | -0.16 | -0.07 | 0.07 | 0.03 | -0.04 | -0.20 | -0.14 | 0.17 | -0.03 |
| TCGA.22.4605.01 | 0.010 | 0.12 | 0.00 | -0.02 | 0.03 | -0.08 | 0.01 | 0.05 | -0.05 | 0.07 | 0.05 | 0.18 | 0.00 | 0.12 | 0.08 | 0.09 | -0.01 | 0.24 | 0.02 | 0.15 | 0.00 | -0.06 | -0.06 | 0.08 | 0.07 | 0.13 | 0.13 | -0.04 | -0.16 |
| TCGA.22.4607.01 | 0.164 | -0.23 | -0.39 | -0.19 | -0.08 | -0.20 | -0.21 | -0.13 | -0.33 | -0.47 | -0.05 | -0.10 | -0.39 | 0.06 | -0.07 | -0.24 | -0.22 | -0.17 | -0.13 | -0.09 | -0.19 | -0.29 | -0.22 | 0.04 | -0.29 | -0.12 | -0.13 | -0.09 | 0.19 |
| TCGA.22.4609.01 | -0.312 | -0.04 | 0.18 | 0.11 | -0.01 | 0.17 | 0.25 | 0.01 | -0.04 | 0.23 | -0.01 | 0.15 | 0.18 | -0.07 | 0.16 | 0.09 | -0.17 | -0.09 | -0.18 | 0.05 | 0.20 | 0.16 | 0.15 | 0.10 | 0.23 | 0.23 | -0.04 | 0.13 | -0.25 |
| TCGA.22.4613.01 | -0.166 | -0.24 | -0.10 | -0.15 | -0.23 | -0.13 | -0.18 | -0.08 | -0.32 | -0.18 | -0.05 | -0.06 | -0.10 | -0.07 | 0.03 | -0.14 | -0.12 | -0.29 | -0.11 | -0.14 | 0.00 | -0.27 | -0.19 | -0.13 | -0.08 | -0.17 | -0.11 | -0.14 | -0.29 |
| TCGA.22.5471.01 | 0.380 | 0.34 | 0.01 | 0.06 | -0.10 | 0.09 | 0.15 | -0.01 | 0.00 | 0.13 | -0.07 | -0.12 | 0.01 | 0.01 | 0.12 | 0.26 | 0.38 | 0.16 | 0.03 | -0.02 | 0.23 | 0.09 | 0.14 | 0.22 | 0.10 | 0.20 | 0.25 | -0.12 | -0.17 |
| TCGA.22.5472.01 | 0.240 | 0.09 | 0.08 | 0.02 | 0.17 | 0.11 | 0.10 | 0.15 | 0.38 | 0.20 | -0.02 | 0.13 | 0.08 | 0.19 | 0.00 | -0.07 | -0.15 | -0.10 | -0.13 | 0.25 | -0.04 | 0.13 | 0.13 | 0.23 | 0.08 | -0.01 | 0.05 | 0.25 | 0.04 |
| TCGA.22.5473.01 | -0.210 | 0.47 | -0.05 | 0.24 | 0.23 | 0.19 | 0.22 | 0.13 | 0.22 | 0.39 | 0.06 | 0.15 | -0.05 | 0.06 | 0.12 | 0.40 | 0.43 | 0.37 | 0.00 | 0.22 | 0.08 | 0.25 | 0.17 | 0.15 | 0.18 | 0.22 | 0.15 | 0.07 | -0.07 |
| TCGA.22.5474.01 | 0.259 | 0.38 | 0.14 | 0.14 | -0.15 | 0.12 | 0.24 | 0.21 | -0.03 | -0.02 | 0.24 | 0.30 | 0.14 | 0.12 | 0.13 | 0.12 | 0.04 | 0.21 | 0.25 | -0.04 | 0.26 | -0.37 | 0.06 | 0.03 | 0.17 | 0.21 | 0.28 | -0.10 | -0.22 |
| TCGA.22.5477.01 | 0.383 | 0.13 | -0.26 | -0.05 | 0.28 | -0.09 | -0.09 | 0.10 | 0.30 | 0.40 | 0.16 | 0.17 | -0.26 | -0.01 | 0.08 | -0.18 | -0.27 | -0.17 | 0.18 | 0.23 | -0.25 | 0.30 | -0.10 | 0.09 | -0.08 | -0.07 | -0.09 | 0.30 | 0.30 |
| TCGA.22.5478.01 | 0.130 | 0.34 | 0.09 | 0.18 | 0.22 | 0.13 | 0.17 | 0.05 | 0.30 | 0.10 | 0.25 | 0.00 | 0.09 | 0.18 | 0.15 | 0.37 | 0.41 | 0.44 | 0.15 | 0.22 | 0.10 | -0.04 | 0.29 | 0.09 | 0.15 | 0.12 | 0.27 | 0.04 | -0.26 |
| TCGA.22.5479.01 | -0.229 | -0.21 | -0.17 | -0.06 | 0.06 | -0.10 | -0.15 | -0.10 | -0.11 | -0.16 | -0.19 | 0.01 | -0.17 | 0.21 | -0.08 | -0.15 | -0.29 | -0.13 | 0.18 | -0.05 | -0.24 | 0.14 | 0.02 | 0.06 | -0.14 | -0.20 | -0.08 | 0.07 | -0.05 |
| TCGA.22.5480.01 | 0.390 | 0.52 | 0.20 | 0.34 | 0.23 | 0.34 | 0.41 | 0.23 | 0.22 | 0.45 | 0.35 | 0.13 | 0.20 | 0.26 | 0.37 | 0.40 | 0.31 | 0.20 | 0.41 | 0.22 | 0.46 | 0.21 | 0.39 | 0.28 | 0.34 | 0.41 | 0.40 | 0.18 | -0.15 |
| TCGA.22.5481.01 | -0.266 | 0.04 | 0.27 | 0.33 | 0.12 | 0.37 | 0.38 | 0.07 | 0.12 | 0.34 | 0.18 | 0.16 | 0.27 | 0.23 | 0.36 | 0.36 | 0.25 | 0.33 | -0.02 | 0.20 | 0.36 | 0.30 | 0.38 | 0.25 | 0.34 | 0.43 | 0.23 | 0.10 | -0.22 |
| TCGA.22.5482.01 | -0.346 | 0.11 | 0.32 | -0.21 | 0.03 | -0.14 | -0.09 | 0.22 | 0.35 | 0.14 | 0.03 | 0.15 | 0.32 | -0.08 | -0.01 | -0.09 | -0.03 | -0.19 | 0.25 | -0.08 | 0.00 | -0.13 | -0.08 | 0.00 | -0.07 | -0.05 | -0.02 | 0.08 | 0.04 |
| TCGA.22.5483.01 | -0.078 | -0.17 | -0.23 | -0.09 | -0.21 | -0.09 | -0.11 | -0.20 | 0.07 | -0.38 | -0.15 | -0.20 | -0.23 | -0.09 | -0.14 | -0.06 | -0.13 | -0.05 | -0.16 | -0.29 | -0.08 | -0.25 | -0.09 | -0.22 | -0.11 | -0.12 | -0.02 | -0.06 | -0.17 |
| TCGA.22.5485.01 | -0.117 | 0.03 | 0.07 | 0.20 | 0.04 | 0.21 | 0.26 | 0.21 | 0.22 | 0.38 | 0.28 | 0.14 | 0.07 | 0.17 | 0.13 | 0.31 | 0.22 | 0.09 | -0.12 | 0.11 | 0.32 | -0.06 | 0.27 | -0.21 | 0.15 | 0.11 | 0.23 | -0.11 | -0.29 |
| TCGA.22.5489.01 | 0.090 | -0.24 | 0.18 | -0.19 | -0.33 | -0.09 | -0.13 | -0.02 | -0.18 | -0.28 | 0.10 | 0.07 | 0.18 | -0.33 | 0.09 | -0.22 | -0.22 | -0.15 | -0.01 | -0.17 | -0.09 | -0.32 | -0.15 | -0.10 | -0.10 | 0.00 | -0.05 | -0.03 | -0.21 |
| TCGA.22.5492.01 | -0.099 | 0.17 | -0.14 | 0.09 | -0.01 | 0.02 | 0.01 | -0.05 | -0.10 | 0.10 | 0.02 | 0.11 | -0.14 | 0.04 | -0.02 | 0.12 | 0.28 | 0.30 | -0.05 | -0.23 | 0.07 | -0.12 | 0.05 | 0.09 | -0.02 | 0.01 | 0.19 | -0.03 | -0.03 |
| TCGA.22.A5C4.01 | -0.021 | -0.32 | -0.10 | -0.06 | -0.06 | -0.14 | -0.27 | 0.00 | -0.29 | 0.12 | -0.07 | -0.17 | -0.10 | -0.25 | -0.13 | -0.36 | -0.20 | -0.20 | -0.26 | -0.15 | 0.03 | 0.11 | -0.16 | -0.08 | 0.01 | 0.03 | -0.21 | -0.07 | -0.04 |
| TCGA.33.4532.01 | -0.418 | -0.23 | 0.01 | -0.01 | 0.00 | 0.00 | -0.08 | 0.00 | -0.10 | -0.08 | -0.08 | -0.01 | 0.01 | 0.16 | -0.16 | -0.27 | -0.09 | -0.10 | -0.13 | -0.10 | -0.18 | -0.12 | 0.01 | -0.20 | -0.03 | -0.21 | -0.06 | 0.00 | 0.17 |
| TCGA.33.4533.01 | -0.120 | -0.34 | -0.28 | -0.03 | -0.25 | 0.02 | -0.09 | -0.24 | -0.40 | -0.40 | 0.03 | -0.25 | -0.28 | -0.08 | -0.05 | 0.01 | 0.07 | 0.13 | -0.02 | -0.07 | -0.03 | -0.28 | -0.19 | 0.00 | -0.12 | -0.10 | 0.03 | -0.26 | -0.07 |
| TCGA.33.4538.01 | 0.234 | -0.04 | 0.14 | -0.05 | 0.02 | -0.10 | -0.10 | 0.04 | -0.10 | -0.08 | -0.10 | 0.10 | 0.14 | 0.12 | -0.06 | -0.24 | -0.27 | 0.10 | -0.02 | 0.08 | -0.22 | 0.11 | -0.04 | 0.15 | -0.14 | -0.18 | -0.08 | 0.15 | 0.03 |
| TCGA.33.4547.01 | -0.620 | 0.16 | 0.27 | 0.03 | 0.07 | 0.15 | 0.06 | 0.11 | -0.27 | 0.14 | 0.15 | 0.04 | 0.27 | -0.21 | 0.14 | 0.24 | -0.06 | -0.08 | -0.25 | 0.03 | 0.27 | 0.14 | 0.13 | -0.02 | 0.25 | 0.18 | 0.10 | -0.07 | -0.28 |
| TCGA.33.4582.01 | -0.141 | -0.03 | -0.46 | -0.17 | 0.23 | -0.18 | -0.24 | -0.04 | -0.08 | -0.12 | -0.01 | 0.07 | -0.46 | -0.01 | -0.15 | -0.42 | -0.37 | -0.32 | 0.19 | 0.06 | -0.40 | -0.18 | -0.26 | 0.03 | -0.23 | -0.23 | -0.30 | 0.14 | 0.26 |
| TCGA.33.4583.01 | -0.330 | 0.46 | 0.12 | 0.29 | 0.19 | 0.31 | 0.30 | 0.12 | 0.16 | 0.33 | 0.24 | 0.21 | 0.12 | 0.13 | 0.32 | 0.38 | 0.22 | 0.39 | 0.00 | 0.31 | 0.29 | 0.39 | 0.34 | 0.28 | 0.34 | 0.39 | 0.16 | 0.20 | -0.12 |
| TCGA.33.4586.01 | 0.010 | 0.11 | -0.05 | 0.01 | -0.13 | -0.01 | -0.04 | 0.16 | 0.06 | 0.16 | 0.15 | 0.04 | -0.05 | -0.08 | 0.15 | -0.03 | 0.09 | -0.08 | 0.14 | -0.02 | 0.16 | -0.14 | 0.05 | -0.21 | -0.01 | 0.10 | 0.10 | 0.11 | -0.22 |
| TCGA.33.4587.01 | -0.164 | 0.16 | 0.27 | 0.09 | 0.17 | 0.19 | 0.16 | 0.19 | -0.06 | 0.22 | 0.00 | 0.08 | 0.27 | -0.06 | -0.05 | -0.05 | 0.11 | -0.08 | -0.07 | 0.05 | 0.26 | -0.01 | 0.09 | 0.10 | 0.22 | 0.19 | 0.12 | -0.12 | -0.28 |
| TCGA.33.4589.01 | -0.350 | -0.49 | 0.31 | 0.04 | -0.09 | 0.12 | -0.03 | -0.09 | -0.14 | -0.22 | -0.26 | -0.25 | 0.31 | -0.01 | -0.02 | 0.23 | 0.38 | 0.20 | -0.16 | 0.03 | 0.18 | -0.05 | 0.10 | -0.26 | 0.17 | 0.01 | 0.00 | -0.31 | -0.38 |
| TCGA.33.6737.01 | -0.131 | -0.14 | -0.18 | 0.02 | 0.26 | 0.03 | 0.00 | 0.14 | 0.17 | 0.00 | -0.14 | 0.11 | -0.18 | 0.31 | 0.05 | -0.10 | 0.04 | 0.16 | 0.21 | 0.00 | -0.22 | 0.18 | 0.07 | 0.24 | 0.08 | -0.10 | -0.10 | 0.27 | 0.26 |
| TCGA.33.6738.01 | -0.102 | 0.30 | -0.20 | -0.04 | 0.09 | -0.03 | -0.04 | 0.35 | 0.23 | 0.31 | 0.32 | 0.30 | -0.20 | -0.02 | 0.00 | -0.35 | -0.48 | -0.39 | 0.17 | 0.10 | -0.04 | 0.07 | 0.07 | 0.05 | -0.05 | -0.08 | 0.00 | 0.20 | 0.06 |
| TCGA.33.A5GW.01 | -0.169 | -0.24 | 0.35 | 0.16 | -0.20 | 0.25 | 0.24 | 0.03 | -0.13 | -0.02 | -0.07 | 0.00 | 0.35 | 0.07 | 0.11 | 0.31 | 0.26 | 0.06 | -0.05 | -0.19 | 0.37 | 0.04 | 0.20 | 0.03 | 0.32 | 0.27 | 0.06 | -0.13 | -0.27 |
| TCGA.33.AAS8.01 | 0.092 | 0.10 | 0.04 | -0.21 | -0.18 | 0.03 | -0.05 | -0.04 | 0.00 | -0.09 | 0.04 | -0.12 | 0.04 | -0.09 | -0.01 | -0.10 | -0.25 | -0.33 | 0.00 | -0.06 | -0.01 | 0.01 | -0.05 | -0.23 | -0.05 | 0.06 | -0.03 | 0.05 | 0.02 |
| TCGA.33.AASB.01 | 0.299 | -0.35 | -0.04 | -0.15 | 0.08 | -0.25 | -0.30 | 0.03 | -0.02 | -0.34 | -0.14 | 0.04 | -0.04 | -0.05 | -0.26 | -0.35 | -0.31 | -0.09 | 0.06 | -0.08 | -0.21 | -0.19 | -0.25 | -0.19 | -0.18 | -0.20 | -0.23 | 0.20 | 0.32 |
| TCGA.33.AASD.01 | 0.220 | -0.16 | -0.52 | -0.14 | 0.04 | -0.20 | -0.27 | -0.10 | -0.13 | -0.30 | -0.01 | -0.02 | -0.52 | 0.12 | -0.20 | -0.27 | -0.36 | -0.27 | -0.27 | -0.18 | -0.18 | -0.25 | -0.30 | -0.12 | -0.27 | -0.22 | -0.23 | -0.09 | 0.19 |
| TCGA.33.AASI.01 | 0.100 | -0.39 | 0.42 | 0.00 | -0.16 | 0.16 | 0.10 | -0.16 | 0.00 | 0.19 | -0.24 | -0.31 | 0.42 | -0.19 | -0.09 | 0.30 | 0.19 | 0.19 | -0.23 | -0.15 | 0.25 | 0.04 | 0.15 | -0.16 | 0.18 | 0.07 | 0.00 | -0.27 | -0.24 |
| TCGA.33.AASJ.01 | 0.065 | 0.58 | 0.16 | 0.24 | 0.24 | 0.28 | 0.38 | 0.08 | 0.25 | 0.30 | 0.12 | 0.21 | 0.16 | 0.34 | 0.26 | 0.41 | 0.42 | 0.39 | 0.30 | 0.25 | 0.25 | 0.33 | 0.33 | 0.33 | 0.26 | 0.30 | 0.33 | 0.18 | -0.21 |
| TCGA.33.AASL.01 | -0.119 | -0.55 | -0.07 | -0.27 | -0.36 | -0.27 | -0.24 | -0.12 | -0.02 | -0.51 | -0.26 | -0.30 | -0.07 | -0.32 | -0.34 | -0.38 | -0.03 | -0.10 | -0.06 | -0.26 | -0.28 | -0.34 | -0.36 | -0.26 | -0.29 | -0.29 | -0.27 | -0.08 | -0.02 |
| TCGA.34.2596.01 | 0.443 | 0.21 | -0.13 | -0.05 | 0.37 | -0.02 | 0.03 | 0.20 | 0.19 | 0.32 | 0.24 | 0.19 | -0.13 | 0.14 | 0.33 | 0.05 | -0.14 | -0.27 | 0.15 | 0.22 | -0.03 | 0.27 | 0.20 | 0.12 | 0.05 | 0.18 | 0.04 | 0.27 | 0.23 |
| TCGA.34.2600.01 | 0.118 | -0.11 | -0.22 | -0.22 | 0.04 | -0.19 | -0.12 | -0.16 | 0.28 | -0.18 | -0.25 | -0.09 | -0.22 | -0.03 | -0.21 | -0.32 | -0.47 | -0.33 | -0.15 | -0.03 | -0.24 | 0.06 | -0.13 | -0.13 | -0.17 | -0.10 | -0.21 | 0.06 | 0.16 |
| TCGA.34.2608.01 | 0.286 | 0.39 | 0.05 | 0.13 | 0.20 | 0.25 | 0.22 | 0.26 | -0.01 | 0.20 | 0.27 | 0.20 | 0.05 | 0.16 | 0.35 | 0.09 | 0.07 | 0.05 | -0.14 | 0.07 | 0.23 | 0.13 | 0.17 | 0.29 | 0.11 | 0.24 | 0.27 | -0.09 | 0.13 |
| TCGA.34.5231.01 | -0.339 | 0.06 | 0.29 | 0.11 | 0.23 | 0.08 | 0.16 | -0.21 | 0.12 | -0.22 | -0.30 | -0.16 | 0.29 | 0.31 | -0.06 | 0.39 | 0.37 | 0.35 | 0.16 | -0.05 | 0.09 | 0.24 | 0.18 | 0.03 | -0.05 | -0.02 | 0.09 | -0.15 | 0.13 |
| TCGA.34.5232.01 | -0.142 | -0.53 | -0.47 | -0.36 | -0.42 | -0.36 | -0.37 | -0.36 | -0.39 | -0.49 | -0.20 | -0.31 | -0.47 | -0.32 | -0.22 | -0.35 | -0.38 | -0.32 | -0.14 | -0.20 | -0.37 | -0.41 | -0.38 | -0.34 | -0.38 | -0.38 | -0.30 | -0.29 | 0.11 |
| TCGA.34.5234.01 | -0.571 | 0.08 | 0.35 | -0.01 | 0.02 | 0.13 | 0.03 | -0.04 | -0.38 | 0.16 | 0.10 | -0.02 | 0.35 | -0.33 | 0.00 | 0.24 | -0.10 | -0.07 | -0.28 | -0.01 | 0.23 | 0.07 | -0.01 | -0.18 | 0.16 | 0.04 | 0.09 | -0.20 | -0.35 |
| TCGA.34.5236.01 | 0.080 | 0.22 | -0.46 | -0.16 | 0.04 | -0.19 | -0.12 | -0.23 | 0.04 | -0.35 | 0.02 | -0.17 | -0.46 | 0.14 | -0.09 | -0.01 | 0.00 | 0.08 | 0.02 | 0.01 | -0.24 | -0.22 | -0.13 | -0.07 | -0.16 | -0.28 | -0.11 | -0.01 | 0.12 |
| TCGA.34.5239.01 | -0.322 | 0.31 | 0.26 | 0.13 | 0.12 | 0.19 | 0.11 | 0.21 | -0.06 | 0.28 | 0.31 | 0.21 | 0.26 | -0.09 | 0.10 | 0.25 | -0.02 | 0.10 | -0.19 | 0.04 | 0.22 | 0.20 | 0.03 | 0.03 | 0.30 | 0.34 | 0.11 | -0.01 | -0.20 |
| TCGA.34.5240.01 | 0.418 | -0.02 | -0.14 | -0.14 | -0.06 | -0.06 | -0.15 | -0.01 | -0.27 | -0.31 | 0.06 | -0.01 | -0.14 | -0.13 | -0.02 | -0.30 | -0.36 | -0.36 | 0.06 | 0.08 | -0.30 | -0.09 | -0.21 | -0.02 | -0.30 | -0.14 | -0.07 | 0.06 | 0.31 |
| TCGA.34.5241.01 | 0.015 | 0.11 | -0.38 | -0.04 | 0.00 | -0.02 | -0.03 | -0.06 | -0.28 | -0.38 | -0.03 | 0.01 | -0.38 | 0.13 | 0.09 | 0.03 | 0.11 | 0.21 | 0.27 | 0.14 | -0.08 | -0.32 | -0.19 | 0.10 | -0.11 | 0.01 | 0.02 | -0.05 | 0.13 |
| TCGA.34.5927.01 | 0.088 | 0.02 | -0.37 | -0.11 | 0.01 | -0.19 | -0.21 | 0.02 | 0.03 | 0.28 | 0.10 | 0.02 | -0.37 | -0.18 | -0.03 | -0.32 | -0.35 | -0.33 | -0.09 | 0.04 | -0.34 | 0.08 | -0.24 | -0.24 | -0.19 | -0.18 | -0.20 | 0.01 | 0.16 |
| TCGA.34.5928.01 | 0.673 | -0.38 | -0.14 | -0.33 | -0.22 | -0.36 | -0.38 | -0.39 | -0.05 | -0.30 | -0.38 | -0.40 | -0.14 | -0.23 | -0.22 | -0.28 | -0.17 | -0.23 | -0.15 | -0.07 | -0.38 | -0.12 | -0.24 | -0.14 | -0.33 | -0.28 | -0.38 | 0.02 | 0.37 |
| TCGA.34.5929.01 | -0.200 | -0.06 | 0.14 | -0.02 | 0.03 | -0.04 | 0.05 | 0.23 | -0.01 | 0.07 | -0.06 | 0.13 | 0.14 | -0.02 | 0.08 | -0.01 | 0.15 | 0.23 | -0.15 | 0.01 | 0.09 | -0.05 | 0.01 | 0.01 | 0.15 | 0.02 | -0.01 | -0.10 | -0.25 |
| TCGA.34.8454.01 | -0.093 | -0.23 | -0.18 | -0.20 | -0.21 | -0.26 | -0.27 | -0.07 | -0.17 | -0.04 | -0.08 | -0.09 | -0.18 | -0.21 | -0.11 | -0.37 | -0.22 | -0.25 | -0.08 | -0.01 | -0.16 | -0.03 | -0.21 | -0.06 | -0.16 | -0.08 | -0.24 | 0.03 | 0.29 |
| TCGA.34.8455.01 | 0.100 | -0.19 | -0.04 | -0.14 | -0.01 | -0.12 | -0.09 | -0.10 | 0.04 | -0.10 | -0.19 | -0.13 | -0.04 | -0.17 | -0.06 | -0.32 | -0.39 | -0.38 | -0.24 | -0.11 | -0.03 | -0.06 | -0.15 | -0.16 | -0.10 | 0.04 | -0.17 | -0.06 | -0.02 |
| TCGA.34.8456.01 | -0.098 | 0.54 | 0.33 | 0.29 | 0.39 | 0.24 | 0.28 | 0.38 | 0.16 | 0.46 | 0.42 | 0.31 | 0.33 | 0.19 | 0.38 | 0.25 | 0.25 | 0.33 | 0.34 | 0.34 | 0.17 | 0.40 | 0.31 | 0.37 | 0.23 | 0.26 | 0.32 | 0.38 | 0.11 |
| TCGA.34.A5IX.01 | -0.414 | 0.30 | -0.12 | 0.04 | 0.16 | -0.07 | -0.11 | 0.02 | 0.02 | -0.02 | 0.11 | 0.26 | -0.12 | 0.13 | -0.19 | 0.03 | 0.11 | 0.37 | 0.15 | 0.14 | -0.11 | -0.27 | -0.06 | -0.09 | -0.15 | -0.24 | 0.04 | 0.02 | 0.07 |
| TCGA.37.4129.01 | -0.198 | -0.43 | -0.39 | -0.20 | -0.19 | -0.20 | -0.27 | -0.22 | 0.13 | -0.25 | -0.22 | -0.28 | -0.39 | -0.18 | 0.00 | -0.46 | -0.55 | -0.47 | -0.32 | -0.20 | -0.07 | 0.12 | -0.21 | -0.12 | -0.04 | 0.02 | -0.35 | -0.05 | 0.31 |
| TCGA.37.A5EL.01 | -0.180 | 0.12 | 0.41 | 0.11 | -0.11 | 0.14 | 0.15 | 0.01 | -0.22 | 0.06 | -0.15 | -0.15 | 0.41 | -0.10 | 0.13 | 0.13 | 0.04 | -0.03 | -0.19 | -0.06 | 0.32 | 0.01 | 0.20 | -0.01 | 0.16 | 0.23 | 0.05 | -0.08 | -0.19 |
| TCGA.37.A5EM.01 | 0.465 | 0.06 | 0.23 | 0.14 | 0.03 | 0.21 | 0.27 | 0.32 | 0.31 | 0.30 | 0.28 | 0.14 | 0.23 | 0.03 | 0.36 | 0.31 | 0.26 | 0.05 | 0.25 | 0.12 | 0.37 | -0.01 | 0.30 | 0.03 | 0.31 | 0.37 | 0.33 | -0.06 | -0.24 |
| TCGA.37.A5EN.01 | -0.485 | 0.21 | 0.35 | 0.32 | 0.21 | 0.35 | 0.36 | 0.18 | -0.13 | 0.31 | 0.20 | 0.22 | 0.35 | 0.09 | 0.31 | 0.49 | 0.39 | 0.30 | 0.00 | 0.07 | 0.42 | 0.17 | 0.29 | 0.11 | 0.42 | 0.36 | 0.27 | 0.01 | -0.34 |
| TCGA.38.4625.01 | 0.145 | -0.05 | 0.26 | 0.07 | -0.07 | 0.08 | 0.11 | 0.14 | -0.05 | -0.25 | -0.15 | -0.06 | 0.26 | -0.01 | -0.07 | -0.12 | -0.06 | -0.25 | 0.11 | -0.19 | 0.33 | -0.16 | 0.01 | -0.11 | 0.10 | 0.02 | 0.10 | -0.17 | -0.26 |
| TCGA.38.4626.01 | 0.461 | 0.46 | 0.04 | 0.27 | 0.23 | 0.28 | 0.28 | -0.11 | 0.00 | 0.27 | -0.04 | -0.15 | 0.04 | 0.33 | 0.23 | 0.41 | 0.18 | 0.29 | 0.18 | 0.25 | 0.16 | 0.35 | 0.37 | 0.30 | 0.18 | 0.37 | 0.15 | 0.30 | 0.33 |
| TCGA.38.4627.01 | 0.415 | 0.39 | 0.07 | 0.21 | 0.03 | 0.25 | 0.33 | 0.25 | -0.02 | 0.24 | 0.35 | 0.19 | 0.07 | 0.13 | 0.34 | 0.15 | -0.01 | 0.11 | 0.20 | 0.17 | 0.35 | -0.06 | 0.24 | 0.31 | 0.29 | 0.37 | 0.32 | -0.01 | -0.06 |
| TCGA.38.4628.01 | -0.234 | 0.42 | 0.16 | 0.05 | 0.30 | 0.06 | 0.03 | 0.30 | 0.22 | 0.31 | 0.26 | 0.33 | 0.16 | 0.13 | 0.12 | -0.27 | -0.30 | -0.09 | 0.09 | 0.13 | -0.06 | 0.15 | 0.21 | 0.20 | 0.07 | -0.03 | 0.09 | 0.28 | 0.24 |
| TCGA.38.4629.01 | 0.275 | 0.35 | -0.39 | 0.13 | 0.14 | 0.12 | 0.18 | -0.13 | -0.14 | -0.27 | 0.02 | 0.03 | -0.39 | 0.25 | 0.18 | 0.21 | 0.36 | 0.25 | 0.38 | 0.12 | 0.10 | -0.07 | 0.08 | 0.27 | 0.01 | 0.12 | 0.22 | 0.07 | 0.27 |
| TCGA.38.4630.01 | -0.330 | 0.10 | 0.34 | 0.26 | 0.02 | 0.26 | 0.21 | 0.23 | 0.02 | 0.34 | 0.13 | 0.11 | 0.34 | 0.21 | 0.13 | 0.44 | 0.40 | 0.35 | -0.11 | 0.10 | 0.31 | 0.14 | 0.26 | 0.20 | 0.24 | 0.12 | 0.20 | -0.02 | 0.00 |
| TCGA.38.4631.01 | -0.556 | 0.28 | 0.31 | 0.30 | 0.15 | 0.35 | 0.35 | 0.18 | -0.07 | 0.32 | 0.19 | 0.25 | 0.31 | 0.10 | 0.28 | 0.39 | 0.25 | 0.24 | -0.04 | 0.16 | 0.45 | 0.17 | 0.34 | 0.08 | 0.41 | 0.37 | 0.27 | 0.10 | -0.39 |
| TCGA.38.4632.01 | 0.154 | -0.51 | 0.07 | -0.20 | -0.22 | -0.22 | -0.31 | -0.23 | -0.26 | -0.27 | -0.28 | -0.27 | 0.07 | -0.18 | -0.19 | -0.23 | 0.04 | -0.10 | -0.30 | -0.06 | -0.26 | 0.12 | -0.29 | -0.15 | -0.28 | -0.12 | -0.33 | -0.09 | 0.22 |
| TCGA.38.6178.01 | 0.144 | -0.23 | -0.12 | -0.15 | 0.10 | -0.25 | -0.20 | 0.05 | 0.11 | -0.22 | -0.30 | 0.02 | -0.12 | 0.00 | -0.16 | -0.19 | -0.19 | 0.10 | -0.17 | -0.06 | -0.26 | 0.00 | -0.08 | -0.07 | -0.17 | -0.22 | -0.18 | 0.08 | 0.19 |
| TCGA.38.7271.01 | 0.256 | 0.30 | -0.05 | 0.21 | 0.34 | 0.06 | 0.12 | 0.32 | 0.22 | 0.08 | 0.15 | 0.37 | -0.05 | 0.38 | 0.08 | 0.03 | 0.18 | 0.28 | 0.20 | 0.04 | 0.09 | 0.16 | 0.17 | 0.22 | 0.06 | 0.01 | 0.11 | 0.15 | 0.00 |
| TCGA.39.5011.01 | -0.260 | 0.09 | 0.34 | -0.01 | -0.04 | 0.18 | 0.15 | 0.04 | -0.20 | 0.43 | 0.08 | -0.10 | 0.34 | -0.02 | 0.16 | 0.17 | 0.10 | 0.02 | -0.11 | -0.05 | 0.24 | 0.28 | 0.20 | 0.02 | 0.19 | 0.23 | 0.07 | -0.02 | -0.19 |
| TCGA.39.5016.01 | 0.269 | -0.10 | 0.00 | -0.14 | 0.14 | -0.24 | -0.19 | 0.01 | 0.13 | 0.05 | -0.18 | 0.01 | 0.00 | -0.17 | -0.04 | -0.12 | 0.11 | -0.04 | -0.14 | 0.06 | -0.32 | 0.20 | -0.13 | 0.11 | -0.30 | -0.19 | -0.09 | 0.19 | 0.41 |
| TCGA.39.5019.01 | -0.216 | -0.40 | 0.02 | -0.04 | -0.41 | 0.02 | -0.14 | -0.25 | -0.48 | -0.27 | -0.18 | -0.27 | 0.02 | -0.25 | -0.04 | 0.17 | 0.24 | 0.02 | -0.29 | -0.18 | -0.01 | -0.31 | -0.12 | -0.15 | -0.07 | -0.12 | -0.16 | -0.35 | -0.22 |
| TCGA.39.5021.01 | -0.016 | -0.06 | -0.02 | -0.02 | -0.13 | -0.16 | -0.14 | -0.28 | -0.13 | -0.17 | -0.30 | -0.27 | -0.02 | 0.01 | -0.14 | 0.03 | -0.06 | 0.02 | 0.00 | -0.11 | -0.21 | 0.07 | -0.15 | -0.04 | -0.20 | -0.08 | -0.10 | 0.05 | 0.11 |
| TCGA.39.5022.01 | -0.068 | 0.45 | 0.19 | 0.09 | 0.25 | 0.10 | 0.15 | 0.10 | 0.28 | -0.13 | -0.12 | 0.03 | 0.19 | 0.21 | 0.15 | -0.21 | -0.06 | 0.06 | 0.35 | 0.21 | -0.05 | 0.06 | 0.05 | 0.21 | -0.05 | -0.03 | 0.11 | 0.28 | -0.03 |
| TCGA.39.5024.01 | 0.251 | 0.49 | -0.30 | 0.14 | 0.32 | -0.08 | -0.01 | -0.01 | 0.17 | -0.03 | 0.10 | 0.22 | -0.30 | 0.27 | -0.12 | 0.02 | -0.02 | 0.27 | 0.31 | 0.22 | -0.30 | 0.12 | 0.08 | 0.10 | -0.18 | -0.22 | 0.12 | 0.10 | 0.17 |
| TCGA.39.5027.01 | 0.517 | 0.08 | 0.35 | 0.04 | 0.19 | 0.01 | 0.01 | -0.14 | 0.24 | 0.07 | -0.28 | -0.28 | 0.35 | 0.05 | -0.07 | 0.12 | 0.26 | 0.17 | 0.13 | 0.18 | -0.04 | 0.36 | 0.01 | 0.16 | -0.02 | -0.08 | -0.14 | 0.16 | 0.19 |
| TCGA.39.5028.01 | -0.176 | 0.09 | -0.18 | -0.07 | 0.16 | -0.05 | -0.29 | 0.07 | -0.29 | -0.26 | 0.08 | 0.06 | -0.18 | 0.00 | -0.02 | 0.12 | -0.16 | 0.02 | -0.06 | 0.08 | -0.12 | -0.01 | -0.08 | 0.04 | -0.15 | -0.14 | 0.12 | -0.10 | 0.09 |
| TCGA.39.5029.01 | 0.270 | 0.33 | -0.04 | 0.03 | 0.28 | 0.06 | 0.02 | 0.07 | 0.30 | -0.16 | 0.09 | 0.16 | -0.04 | 0.30 | 0.11 | -0.03 | 0.05 | 0.04 | 0.18 | 0.23 | -0.08 | 0.01 | 0.03 | 0.27 | 0.04 | -0.12 | 0.07 | 0.23 | 0.30 |
| TCGA.39.5030.01 | 0.370 | 0.40 | 0.09 | 0.20 | 0.35 | 0.27 | 0.29 | 0.28 | 0.32 | 0.19 | 0.30 | 0.29 | 0.09 | 0.28 | 0.33 | 0.21 | 0.42 | 0.36 | 0.35 | 0.23 | 0.19 | 0.31 | 0.36 | 0.30 | 0.13 | 0.34 | 0.30 | 0.25 | 0.15 |
| TCGA.39.5031.01 | -0.046 | 0.01 | -0.40 | 0.02 | -0.05 | -0.03 | 0.08 | 0.34 | -0.07 | 0.06 | 0.39 | 0.33 | -0.40 | 0.04 | 0.13 | -0.09 | -0.22 | -0.07 | -0.08 | 0.00 | 0.03 | -0.17 | -0.06 | 0.16 | 0.22 | 0.06 | -0.04 | -0.05 | -0.12 |
| TCGA.39.5034.01 | 0.190 | -0.07 | 0.22 | -0.13 | -0.11 | -0.10 | -0.06 | 0.13 | -0.16 | -0.13 | 0.07 | 0.09 | 0.22 | -0.27 | 0.02 | -0.23 | -0.15 | -0.11 | -0.05 | -0.10 | 0.05 | -0.02 | -0.02 | 0.00 | -0.14 | 0.13 | 0.06 | -0.13 | 0.03 |
| TCGA.39.5035.01 | -0.352 | 0.27 | 0.28 | 0.26 | 0.14 | 0.35 | 0.37 | 0.34 | 0.12 | 0.28 | 0.34 | 0.36 | 0.28 | 0.08 | 0.25 | 0.35 | 0.32 | 0.25 | -0.01 | 0.10 | 0.45 | 0.09 | 0.30 | 0.09 | 0.41 | 0.32 | 0.36 | -0.11 | -0.43 |
| TCGA.39.5036.01 | 0.309 | -0.23 | -0.13 | -0.19 | 0.04 | -0.28 | -0.30 | -0.20 | -0.12 | -0.32 | -0.15 | -0.07 | -0.13 | -0.15 | -0.12 | -0.41 | -0.31 | -0.22 | 0.05 | 0.02 | -0.33 | -0.31 | -0.30 | 0.06 | -0.23 | -0.33 | -0.26 | 0.06 | 0.24 |
| TCGA.39.5037.01 | -0.077 | -0.28 | 0.24 | -0.15 | -0.18 | -0.12 | -0.15 | -0.04 | -0.09 | -0.42 | -0.20 | -0.06 | 0.24 | -0.12 | -0.15 | 0.01 | 0.22 | 0.08 | -0.08 | -0.12 | -0.06 | -0.30 | -0.27 | -0.20 | -0.06 | -0.07 | -0.01 | -0.17 | -0.16 |
| TCGA.39.5039.01 | 0.295 | 0.08 | 0.36 | -0.01 | 0.28 | 0.04 | 0.12 | 0.08 | -0.03 | -0.07 | 0.16 | 0.20 | 0.36 | 0.03 | 0.10 | -0.01 | 0.03 | 0.01 | 0.32 | 0.17 | -0.02 | -0.14 | 0.07 | 0.16 | 0.03 | 0.10 | 0.12 | 0.24 | 0.12 |
| TCGA.39.5040.01 | -0.268 | -0.44 | 0.01 | -0.02 | -0.11 | 0.02 | 0.03 | -0.05 | 0.02 | -0.11 | 0.06 | -0.11 | 0.01 | -0.05 | -0.23 | 0.07 | 0.27 | 0.04 | -0.25 | -0.21 | -0.01 | -0.27 | -0.03 | -0.09 | -0.01 | -0.08 | 0.07 | -0.23 | -0.20 |
| TCGA.43.2576.01 | -0.074 | -0.11 | -0.32 | 0.03 | 0.08 | 0.01 | -0.03 | 0.38 | 0.00 | 0.32 | 0.31 | 0.24 | -0.32 | -0.03 | 0.19 | -0.13 | -0.38 | -0.26 | -0.13 | 0.04 | 0.13 | -0.15 | -0.04 | -0.01 | 0.20 | 0.21 | 0.05 | -0.05 | -0.18 |
| TCGA.43.2578.01 | 0.316 | -0.40 | -0.18 | -0.29 | -0.17 | -0.23 | -0.19 | 0.02 | 0.07 | 0.15 | -0.04 | 0.04 | -0.18 | -0.05 | -0.23 | -0.43 | -0.45 | -0.46 | -0.09 | -0.16 | -0.21 | -0.29 | -0.13 | -0.22 | -0.22 | -0.24 | -0.22 | -0.07 | 0.18 |
| TCGA.43.2581.01 | -0.435 | -0.05 | 0.13 | 0.18 | -0.04 | 0.22 | 0.27 | 0.18 | 0.03 | 0.31 | 0.10 | 0.24 | 0.13 | -0.06 | 0.12 | 0.00 | -0.29 | -0.18 | -0.22 | -0.04 | 0.35 | 0.05 | 0.12 | 0.01 | 0.33 | 0.28 | 0.06 | 0.07 | -0.37 |
| TCGA.43.3394.01 | 0.446 | 0.59 | -0.05 | 0.32 | 0.42 | 0.28 | 0.32 | 0.39 | 0.28 | 0.00 | 0.35 | 0.41 | -0.05 | 0.35 | 0.32 | 0.34 | 0.29 | 0.45 | 0.41 | 0.33 | 0.16 | 0.14 | 0.24 | 0.35 | 0.24 | 0.27 | 0.37 | 0.25 | 0.11 |
| TCGA.43.3920.01 | -0.263 | 0.27 | 0.33 | 0.12 | 0.13 | 0.26 | 0.23 | 0.03 | 0.04 | 0.37 | 0.09 | 0.02 | 0.33 | -0.03 | 0.14 | 0.34 | 0.41 | 0.27 | -0.14 | 0.06 | 0.26 | 0.40 | 0.23 | 0.16 | 0.28 | 0.30 | 0.11 | 0.04 | -0.33 |
| TCGA.43.5668.01 | 0.399 | 0.43 | -0.16 | 0.01 | 0.23 | -0.01 | 0.11 | 0.22 | 0.22 | 0.29 | 0.23 | 0.26 | -0.16 | 0.09 | 0.13 | -0.12 | -0.32 | -0.03 | 0.35 | 0.26 | -0.04 | 0.15 | 0.14 | 0.17 | -0.01 | 0.13 | 0.04 | 0.32 | 0.24 |
| TCGA.43.5670.01 | 0.329 | 0.14 | -0.30 | -0.08 | -0.03 | -0.18 | -0.25 | 0.02 | -0.10 | -0.23 | 0.03 | 0.11 | -0.30 | -0.19 | -0.28 | -0.07 | -0.12 | 0.05 | -0.20 | -0.09 | -0.28 | -0.15 | -0.26 | -0.04 | -0.15 | -0.33 | -0.14 | -0.20 | 0.08 |
| TCGA.43.6143.01 | 0.099 | 0.28 | 0.26 | 0.38 | 0.20 | 0.36 | 0.37 | 0.25 | 0.24 | 0.36 | 0.31 | 0.19 | 0.26 | 0.25 | 0.36 | 0.45 | 0.43 | 0.43 | 0.13 | 0.14 | 0.40 | 0.26 | 0.41 | 0.30 | 0.34 | 0.44 | 0.29 | 0.12 | -0.25 |
| TCGA.43.6647.01 | -0.217 | 0.31 | 0.34 | 0.28 | 0.13 | 0.30 | 0.34 | 0.12 | 0.16 | 0.10 | 0.20 | 0.15 | 0.34 | 0.19 | 0.27 | 0.46 | 0.51 | 0.32 | 0.19 | 0.17 | 0.36 | 0.14 | 0.34 | 0.27 | 0.27 | 0.28 | 0.36 | -0.23 | -0.36 |
| TCGA.43.6770.01 | 0.037 | 0.18 | 0.04 | 0.22 | -0.02 | 0.14 | 0.16 | 0.14 | -0.20 | -0.28 | 0.19 | 0.18 | 0.04 | 0.19 | 0.11 | 0.01 | 0.20 | 0.25 | 0.13 | 0.06 | 0.24 | -0.38 | 0.06 | 0.11 | 0.16 | 0.18 | 0.28 | -0.06 | -0.17 |
| TCGA.43.6771.01 | 0.097 | 0.55 | -0.36 | 0.22 | 0.39 | 0.21 | 0.21 | 0.18 | 0.44 | 0.05 | 0.17 | 0.32 | -0.36 | 0.43 | 0.16 | 0.27 | 0.34 | 0.39 | 0.38 | 0.22 | -0.08 | 0.21 | 0.33 | 0.36 | 0.06 | 0.00 | 0.17 | 0.36 | 0.32 |
| TCGA.43.6773.01 | 0.034 | -0.05 | 0.07 | 0.01 | -0.24 | 0.07 | 0.01 | 0.05 | -0.38 | -0.13 | 0.11 | -0.02 | 0.07 | -0.13 | -0.02 | -0.01 | -0.03 | 0.04 | -0.01 | -0.07 | 0.06 | -0.14 | -0.14 | 0.07 | 0.04 | 0.11 | 0.03 | -0.14 | 0.08 |
| TCGA.43.7656.01 | -0.531 | 0.11 | 0.33 | 0.31 | 0.11 | 0.35 | 0.37 | 0.21 | 0.12 | 0.34 | 0.20 | 0.24 | 0.33 | 0.16 | 0.33 | 0.37 | 0.19 | 0.21 | -0.12 | 0.17 | 0.44 | 0.23 | 0.35 | 0.10 | 0.40 | 0.36 | 0.23 | 0.02 | -0.36 |
| TCGA.43.7657.01 | -0.009 | 0.03 | 0.07 | 0.21 | 0.06 | 0.23 | 0.23 | 0.03 | -0.05 | -0.02 | 0.12 | 0.00 | 0.07 | 0.10 | 0.24 | 0.21 | -0.14 | 0.09 | -0.01 | 0.09 | 0.28 | 0.13 | 0.24 | 0.19 | 0.31 | 0.35 | 0.04 | 0.16 | -0.03 |
| TCGA.43.7658.01 | -0.339 | 0.09 | 0.16 | 0.34 | 0.05 | 0.36 | 0.38 | 0.29 | 0.12 | 0.26 | 0.16 | 0.27 | 0.16 | 0.22 | 0.34 | 0.28 | 0.07 | 0.16 | -0.01 | 0.15 | 0.37 | 0.10 | 0.25 | 0.23 | 0.36 | 0.38 | 0.24 | 0.11 | -0.30 |
| TCGA.43.8115.01 | -0.484 | 0.03 | -0.28 | -0.11 | -0.14 | -0.12 | -0.02 | 0.26 | -0.02 | 0.22 | 0.18 | 0.15 | -0.28 | -0.13 | 0.00 | -0.19 | -0.41 | -0.37 | -0.31 | -0.02 | -0.04 | -0.15 | -0.02 | -0.30 | -0.07 | -0.11 | -0.01 | -0.31 | 0.01 |
| TCGA.43.8116.01 | 0.247 | -0.01 | 0.01 | 0.04 | 0.18 | -0.07 | -0.13 | 0.12 | 0.18 | -0.08 | 0.02 | 0.15 | 0.01 | 0.03 | -0.01 | -0.23 | -0.15 | 0.02 | 0.18 | 0.11 | -0.22 | -0.03 | 0.09 | 0.16 | -0.15 | -0.05 | 0.02 | 0.26 | 0.29 |
| TCGA.43.8118.01 | 0.095 | 0.04 | 0.08 | -0.07 | -0.01 | 0.01 | -0.05 | -0.08 | -0.07 | -0.13 | -0.12 | -0.08 | 0.08 | -0.15 | -0.12 | -0.11 | -0.13 | -0.25 | 0.06 | -0.10 | 0.09 | 0.12 | 0.12 | -0.07 | -0.05 | -0.05 | -0.01 | -0.08 | -0.05 |
| TCGA.43.A474.01 | -0.209 | -0.33 | 0.26 | 0.02 | 0.04 | 0.07 | 0.10 | -0.18 | -0.19 | -0.30 | -0.32 | -0.21 | 0.26 | 0.09 | -0.10 | 0.25 | 0.18 | 0.04 | 0.03 | -0.18 | 0.26 | -0.15 | 0.05 | -0.12 | -0.03 | -0.08 | 0.10 | -0.17 | -0.15 |
| TCGA.43.A475.01 | -0.024 | -0.18 | -0.34 | 0.09 | -0.03 | 0.05 | 0.00 | 0.05 | -0.07 | -0.18 | 0.15 | 0.08 | -0.34 | 0.16 | -0.03 | -0.02 | -0.16 | -0.06 | 0.06 | 0.08 | -0.06 | -0.15 | 0.01 | 0.07 | -0.04 | -0.03 | 0.09 | 0.00 | 0.16 |
| TCGA.43.A56U.01 | 0.118 | 0.18 | 0.36 | 0.25 | 0.13 | 0.30 | 0.28 | 0.05 | -0.02 | 0.32 | 0.13 | -0.04 | 0.36 | 0.04 | 0.09 | 0.43 | 0.38 | 0.36 | -0.15 | 0.14 | 0.27 | 0.21 | 0.26 | 0.17 | 0.32 | 0.30 | 0.18 | 0.02 | -0.34 |
| TCGA.43.A56V.01 | -0.368 | -0.16 | -0.18 | -0.18 | 0.03 | -0.19 | -0.17 | 0.08 | -0.02 | -0.25 | -0.14 | -0.01 | -0.18 | 0.04 | -0.14 | -0.22 | -0.23 | -0.02 | 0.17 | -0.08 | -0.16 | -0.18 | -0.05 | -0.04 | -0.15 | -0.28 | -0.11 | -0.05 | -0.01 |
| TCGA.44.2655.01 | 0.056 | 0.07 | 0.16 | 0.13 | -0.03 | 0.16 | 0.09 | 0.05 | 0.03 | 0.04 | 0.14 | -0.04 | 0.16 | -0.02 | 0.17 | 0.28 | 0.27 | -0.06 | 0.04 | -0.01 | 0.22 | 0.21 | 0.16 | 0.16 | 0.10 | 0.09 | 0.12 | 0.04 | -0.10 |
| TCGA.44.2656.01 | -0.182 | 0.39 | 0.26 | -0.05 | 0.07 | 0.03 | -0.03 | -0.27 | -0.35 | -0.02 | -0.05 | -0.17 | 0.26 | -0.20 | -0.10 | 0.21 | 0.03 | -0.02 | 0.06 | 0.04 | -0.01 | 0.24 | -0.05 | -0.06 | -0.15 | -0.06 | 0.01 | -0.12 | 0.06 |
| TCGA.44.2657.01 | -0.129 | 0.37 | 0.09 | 0.22 | 0.36 | 0.22 | 0.23 | 0.26 | 0.30 | 0.15 | 0.30 | 0.32 | 0.09 | 0.29 | 0.25 | 0.35 | 0.16 | 0.26 | 0.21 | 0.22 | 0.24 | -0.07 | 0.26 | 0.17 | 0.27 | 0.19 | 0.31 | 0.10 | -0.15 |
| TCGA.44.2659.01 | 0.068 | 0.37 | 0.03 | -0.03 | -0.01 | 0.12 | 0.04 | -0.02 | -0.13 | 0.22 | -0.02 | -0.11 | 0.03 | 0.05 | 0.02 | 0.06 | -0.06 | -0.19 | -0.06 | -0.02 | 0.08 | 0.14 | 0.15 | 0.04 | -0.06 | 0.09 | -0.02 | 0.02 | 0.13 |
| TCGA.44.2661.01 | 0.516 | 0.59 | -0.23 | 0.24 | 0.26 | 0.21 | 0.29 | 0.25 | 0.46 | 0.37 | 0.31 | 0.34 | -0.23 | 0.23 | 0.32 | 0.21 | 0.03 | 0.25 | 0.34 | 0.24 | 0.11 | 0.19 | 0.22 | 0.36 | 0.12 | 0.34 | 0.32 | 0.27 | 0.09 |
| TCGA.44.2662.01 | -0.599 | 0.30 | 0.36 | 0.06 | 0.05 | 0.13 | 0.10 | -0.15 | -0.30 | 0.16 | 0.03 | -0.13 | 0.36 | -0.18 | 0.04 | 0.22 | 0.02 | 0.02 | 0.00 | 0.06 | 0.22 | 0.14 | 0.12 | -0.19 | 0.15 | 0.08 | 0.12 | -0.11 | -0.28 |
| TCGA.44.2665.01 | -0.070 | -0.40 | 0.49 | 0.14 | -0.21 | 0.15 | 0.09 | 0.08 | -0.04 | 0.27 | 0.08 | -0.15 | 0.49 | -0.13 | 0.15 | 0.22 | 0.22 | -0.11 | -0.19 | -0.09 | 0.40 | 0.14 | 0.22 | -0.17 | 0.27 | 0.27 | 0.06 | -0.08 | -0.28 |
| TCGA.44.2666.01 | -0.662 | -0.06 | 0.33 | -0.03 | 0.00 | 0.04 | -0.03 | -0.01 | -0.28 | 0.25 | 0.03 | -0.04 | 0.33 | -0.27 | -0.03 | -0.09 | -0.28 | -0.26 | -0.34 | -0.08 | 0.29 | 0.28 | 0.02 | -0.20 | 0.19 | 0.10 | -0.07 | -0.17 | -0.33 |
| TCGA.44.2668.01 | -0.023 | 0.47 | -0.15 | 0.08 | 0.09 | 0.09 | -0.08 | 0.09 | -0.06 | 0.32 | 0.25 | 0.16 | -0.15 | 0.16 | 0.12 | 0.20 | 0.18 | 0.23 | 0.25 | 0.32 | -0.13 | -0.10 | 0.05 | -0.03 | 0.02 | -0.02 | 0.17 | 0.18 | 0.20 |
| TCGA.44.3396.01 | 0.230 | 0.12 | -0.16 | -0.02 | -0.16 | -0.04 | -0.03 | -0.20 | -0.02 | -0.20 | -0.12 | -0.19 | -0.16 | 0.15 | -0.01 | 0.13 | 0.43 | 0.38 | 0.07 | -0.07 | -0.18 | -0.03 | 0.06 | 0.13 | -0.20 | -0.13 | -0.01 | -0.06 | 0.28 |
| TCGA.44.3398.01 | -0.040 | -0.22 | 0.24 | 0.18 | -0.15 | 0.18 | 0.17 | -0.03 | -0.09 | -0.17 | -0.05 | -0.20 | 0.24 | 0.19 | 0.03 | 0.28 | 0.38 | 0.28 | 0.03 | -0.11 | 0.18 | -0.05 | 0.04 | -0.07 | 0.05 | 0.03 | 0.14 | -0.30 | -0.13 |
| TCGA.44.3917.01 | 0.274 | 0.47 | 0.19 | 0.20 | 0.09 | 0.15 | 0.21 | -0.16 | 0.33 | 0.08 | 0.05 | -0.09 | 0.19 | 0.10 | 0.10 | 0.41 | 0.50 | 0.30 | 0.15 | 0.21 | 0.12 | 0.29 | 0.22 | 0.18 | 0.04 | 0.05 | 0.18 | 0.04 | -0.05 |
| TCGA.44.3918.01 | -0.638 | 0.18 | 0.38 | 0.10 | 0.12 | 0.15 | 0.11 | -0.04 | -0.23 | 0.15 | 0.23 | 0.04 | 0.38 | -0.16 | 0.05 | 0.30 | 0.07 | 0.06 | -0.15 | 0.08 | 0.21 | 0.20 | 0.15 | -0.05 | 0.17 | 0.08 | 0.10 | -0.10 | -0.27 |
| TCGA.44.3919.01 | 0.232 | 0.18 | -0.01 | 0.05 | 0.03 | 0.08 | 0.01 | -0.19 | -0.02 | 0.16 | -0.06 | -0.25 | -0.01 | 0.02 | -0.12 | 0.24 | 0.26 | 0.06 | -0.06 | -0.07 | -0.06 | 0.13 | 0.06 | 0.18 | -0.06 | -0.07 | 0.04 | -0.13 | -0.07 |
| TCGA.44.4112.01 | 0.512 | 0.25 | 0.36 | 0.11 | 0.13 | 0.18 | 0.13 | -0.18 | 0.19 | 0.24 | -0.08 | -0.23 | 0.36 | 0.21 | 0.16 | 0.13 | 0.33 | 0.36 | 0.27 | 0.28 | 0.00 | 0.20 | 0.24 | 0.09 | 0.07 | 0.10 | 0.10 | 0.35 | 0.39 |
| TCGA.44.5643.01 | -0.179 | 0.16 | -0.07 | -0.07 | 0.17 | -0.05 | -0.16 | 0.18 | 0.02 | 0.23 | -0.09 | 0.11 | -0.07 | -0.02 | 0.02 | -0.11 | 0.10 | -0.04 | 0.02 | 0.25 | -0.19 | 0.13 | -0.16 | 0.09 | -0.14 | -0.13 | -0.15 | 0.12 | 0.08 |
| TCGA.44.5644.01 | 0.381 | 0.45 | -0.20 | 0.18 | 0.40 | 0.14 | 0.24 | 0.02 | 0.23 | 0.11 | 0.15 | 0.07 | -0.20 | 0.37 | 0.21 | 0.22 | 0.27 | 0.32 | 0.42 | 0.30 | -0.14 | 0.18 | 0.23 | 0.35 | 0.04 | 0.01 | 0.19 | 0.22 | 0.23 |
| TCGA.44.5645.01 | -0.287 | 0.23 | 0.43 | -0.08 | -0.11 | 0.04 | 0.02 | -0.08 | -0.30 | 0.34 | -0.01 | -0.03 | 0.43 | -0.40 | -0.12 | 0.10 | 0.08 | -0.06 | -0.25 | -0.03 | 0.01 | 0.16 | -0.12 | -0.11 | -0.03 | -0.05 | -0.09 | -0.17 | -0.11 |
| TCGA.44.6145.01 | -0.061 | 0.34 | 0.12 | 0.20 | -0.06 | 0.27 | 0.32 | 0.28 | -0.01 | 0.24 | 0.27 | 0.07 | 0.12 | -0.01 | 0.23 | 0.35 | 0.31 | 0.25 | 0.19 | 0.15 | 0.30 | 0.09 | 0.25 | 0.11 | 0.32 | 0.24 | 0.31 | -0.03 | -0.22 |
| TCGA.44.6147.01 | 0.602 | -0.14 | 0.11 | -0.09 | 0.18 | -0.12 | -0.02 | 0.13 | 0.33 | 0.46 | 0.01 | 0.17 | 0.11 | -0.01 | 0.15 | -0.25 | -0.41 | -0.27 | -0.08 | 0.07 | -0.05 | 0.26 | 0.11 | 0.02 | -0.05 | -0.09 | -0.10 | 0.26 | 0.20 |
| TCGA.44.6774.01 | 0.053 | 0.26 | -0.34 | 0.01 | 0.15 | -0.02 | 0.01 | -0.08 | 0.16 | 0.06 | -0.04 | -0.21 | -0.34 | 0.00 | -0.14 | 0.10 | 0.04 | 0.15 | -0.07 | -0.05 | -0.30 | -0.16 | -0.02 | 0.02 | -0.15 | -0.16 | 0.02 | -0.16 | -0.04 |
| TCGA.44.6775.01 | 0.238 | -0.23 | -0.23 | -0.15 | 0.06 | -0.26 | -0.21 | -0.03 | -0.08 | 0.09 | -0.12 | 0.02 | -0.23 | 0.12 | -0.15 | -0.42 | -0.49 | -0.30 | -0.04 | -0.11 | -0.31 | 0.01 | -0.17 | -0.01 | -0.16 | -0.18 | -0.30 | 0.23 | 0.23 |
| TCGA.44.6776.01 | 0.347 | 0.16 | 0.18 | 0.01 | -0.11 | 0.02 | 0.06 | 0.10 | -0.10 | 0.04 | -0.02 | 0.12 | 0.18 | -0.07 | 0.18 | -0.23 | -0.39 | -0.33 | -0.13 | -0.12 | 0.22 | 0.01 | -0.07 | 0.07 | 0.12 | 0.21 | -0.02 | 0.04 | -0.10 |
| TCGA.44.6777.01 | 0.107 | -0.24 | -0.14 | 0.01 | -0.04 | -0.07 | 0.00 | -0.18 | 0.14 | -0.20 | -0.23 | -0.14 | -0.14 | 0.21 | -0.21 | -0.12 | -0.19 | -0.01 | -0.07 | -0.24 | -0.14 | -0.19 | 0.07 | 0.00 | -0.09 | -0.15 | -0.12 | -0.13 | 0.04 |
| TCGA.44.6778.01 | 0.121 | 0.21 | 0.17 | 0.25 | 0.19 | 0.28 | 0.32 | 0.24 | 0.22 | 0.34 | 0.16 | 0.21 | 0.17 | -0.04 | 0.30 | 0.24 | 0.01 | 0.07 | 0.02 | 0.12 | 0.32 | 0.30 | 0.23 | 0.22 | 0.34 | 0.38 | 0.14 | 0.26 | -0.14 |
| TCGA.44.6779.01 | -0.007 | 0.43 | 0.14 | 0.17 | 0.26 | 0.30 | 0.30 | 0.13 | 0.24 | -0.10 | 0.16 | 0.11 | 0.14 | 0.10 | 0.21 | 0.27 | 0.39 | 0.35 | 0.20 | 0.30 | 0.21 | 0.05 | 0.26 | 0.35 | 0.27 | 0.26 | 0.31 | 0.08 | -0.24 |
| TCGA.44.7659.01 | -0.189 | -0.25 | -0.49 | -0.35 | -0.14 | -0.30 | -0.29 | -0.10 | 0.11 | 0.04 | -0.13 | -0.16 | -0.49 | -0.35 | -0.23 | -0.41 | -0.41 | -0.42 | -0.18 | -0.14 | -0.35 | -0.07 | -0.22 | -0.25 | -0.29 | -0.26 | -0.32 | 0.02 | 0.28 |
| TCGA.44.7660.01 | -0.312 | -0.43 | -0.35 | -0.31 | -0.20 | -0.37 | -0.30 | -0.14 | 0.04 | -0.19 | -0.13 | -0.20 | -0.35 | -0.35 | -0.27 | -0.32 | -0.31 | -0.40 | -0.16 | -0.21 | -0.28 | -0.12 | -0.19 | -0.32 | -0.25 | -0.33 | -0.34 | -0.13 | 0.03 |
| TCGA.44.7661.01 | -0.113 | -0.11 | 0.16 | -0.09 | 0.24 | 0.07 | -0.02 | -0.13 | 0.02 | -0.16 | -0.19 | -0.20 | 0.16 | 0.10 | -0.04 | -0.25 | -0.40 | -0.33 | 0.12 | 0.02 | 0.05 | 0.27 | 0.06 | -0.10 | -0.16 | -0.09 | -0.09 | 0.03 | 0.14 |
| TCGA.44.7662.01 | 0.322 | -0.30 | -0.09 | -0.18 | 0.06 | -0.14 | -0.21 | -0.09 | 0.13 | -0.17 | -0.18 | -0.08 | -0.09 | 0.00 | -0.19 | -0.15 | -0.07 | -0.15 | -0.28 | -0.12 | -0.19 | 0.04 | 0.03 | -0.14 | -0.16 | -0.29 | -0.15 | 0.02 | 0.07 |
| TCGA.44.7667.01 | -0.293 | -0.16 | -0.57 | -0.20 | -0.09 | -0.19 | -0.22 | -0.17 | 0.06 | 0.01 | -0.08 | -0.12 | -0.57 | -0.17 | -0.06 | -0.39 | -0.33 | -0.30 | 0.04 | -0.05 | -0.28 | 0.02 | -0.20 | -0.05 | -0.11 | -0.08 | -0.17 | 0.17 | 0.28 |
| TCGA.44.7669.01 | -0.214 | -0.42 | -0.29 | -0.22 | 0.01 | -0.28 | -0.17 | -0.13 | 0.02 | -0.35 | -0.30 | -0.16 | -0.29 | 0.11 | -0.33 | -0.36 | -0.19 | -0.05 | -0.04 | -0.25 | -0.27 | -0.11 | -0.20 | -0.15 | -0.19 | -0.32 | -0.32 | -0.07 | 0.07 |
| TCGA.44.7670.01 | 0.448 | -0.11 | -0.14 | -0.02 | 0.10 | -0.09 | -0.05 | -0.05 | 0.06 | -0.15 | -0.19 | -0.06 | -0.14 | 0.11 | -0.19 | -0.11 | -0.10 | 0.00 | 0.02 | 0.05 | -0.19 | -0.14 | -0.20 | -0.04 | -0.08 | -0.20 | -0.12 | 0.12 | 0.32 |
| TCGA.44.7671.01 | 0.198 | -0.31 | -0.27 | -0.17 | -0.20 | -0.17 | -0.18 | -0.11 | -0.19 | -0.23 | -0.15 | -0.18 | -0.27 | -0.02 | -0.04 | -0.21 | -0.42 | -0.35 | -0.10 | -0.11 | -0.20 | -0.20 | -0.19 | -0.11 | -0.11 | -0.01 | -0.19 | 0.00 | 0.19 |
| TCGA.44.7672.01 | -0.153 | -0.14 | -0.34 | -0.18 | -0.15 | -0.24 | -0.12 | -0.09 | -0.02 | -0.21 | -0.16 | -0.10 | -0.34 | 0.01 | -0.23 | -0.24 | -0.26 | -0.24 | -0.17 | -0.30 | -0.18 | -0.19 | -0.04 | 0.03 | -0.21 | -0.28 | -0.13 | -0.01 | 0.30 |
| TCGA.44.8117.01 | -0.335 | -0.21 | -0.13 | -0.15 | 0.13 | -0.11 | -0.06 | -0.07 | 0.16 | 0.02 | 0.00 | -0.03 | -0.13 | 0.12 | -0.04 | -0.15 | -0.15 | -0.20 | 0.13 | 0.00 | -0.17 | 0.13 | -0.05 | -0.07 | 0.00 | -0.03 | -0.20 | 0.11 | 0.19 |
| TCGA.44.8119.01 | 0.172 | -0.13 | -0.30 | -0.14 | 0.03 | -0.11 | -0.08 | -0.10 | 0.04 | -0.36 | -0.16 | -0.09 | -0.30 | 0.10 | -0.31 | -0.27 | -0.43 | -0.26 | -0.03 | -0.24 | -0.08 | -0.17 | -0.11 | 0.01 | -0.20 | -0.21 | -0.15 | -0.18 | 0.20 |
| TCGA.44.8120.01 | 0.145 | 0.49 | -0.33 | 0.18 | 0.33 | 0.11 | 0.22 | 0.08 | -0.01 | 0.12 | 0.15 | 0.24 | -0.33 | 0.16 | 0.14 | 0.23 | 0.30 | 0.28 | 0.18 | 0.17 | -0.01 | 0.09 | 0.01 | 0.21 | 0.09 | -0.02 | 0.23 | 0.08 | 0.03 |
| TCGA.44.A479.01 | -0.087 | 0.04 | 0.44 | 0.05 | 0.21 | 0.15 | 0.10 | -0.07 | 0.15 | -0.04 | -0.18 | -0.11 | 0.44 | 0.18 | 0.19 | 0.25 | 0.36 | 0.20 | 0.25 | 0.06 | 0.04 | 0.27 | 0.19 | 0.01 | -0.11 | 0.02 | 0.12 | 0.21 | -0.07 |
| TCGA.44.A47A.01 | 0.312 | -0.36 | 0.16 | -0.25 | -0.13 | -0.29 | -0.32 | -0.44 | -0.25 | -0.36 | -0.22 | -0.41 | 0.16 | -0.20 | -0.23 | -0.07 | 0.06 | -0.16 | -0.09 | -0.12 | -0.33 | -0.27 | -0.31 | -0.31 | -0.30 | -0.26 | -0.30 | 0.05 | 0.22 |
| TCGA.44.A47B.01 | -0.042 | -0.29 | -0.18 | -0.27 | -0.29 | -0.34 | -0.36 | -0.32 | -0.18 | -0.42 | 0.03 | -0.20 | -0.18 | -0.15 | -0.02 | -0.45 | -0.46 | -0.35 | -0.03 | -0.15 | -0.26 | -0.11 | -0.28 | -0.21 | -0.20 | -0.08 | -0.32 | -0.01 | 0.24 |
| TCGA.44.A47G.01 | -0.185 | -0.34 | -0.22 | -0.28 | 0.01 | -0.27 | -0.34 | -0.03 | 0.13 | 0.23 | -0.17 | -0.13 | -0.22 | -0.31 | -0.17 | -0.40 | -0.33 | -0.36 | -0.32 | -0.15 | -0.31 | -0.01 | -0.23 | -0.25 | -0.26 | -0.24 | -0.32 | -0.03 | 0.02 |
| TCGA.44.A4SS.01 | -0.209 | 0.24 | 0.19 | 0.13 | 0.22 | 0.10 | 0.05 | 0.05 | -0.16 | 0.06 | 0.31 | 0.20 | 0.19 | 0.13 | 0.17 | 0.15 | 0.22 | 0.34 | 0.31 | 0.16 | -0.05 | 0.11 | 0.19 | 0.24 | 0.04 | 0.11 | 0.18 | 0.22 | -0.15 |
| TCGA.44.A4SU.01 | -0.437 | 0.09 | -0.33 | -0.09 | 0.01 | -0.17 | -0.12 | 0.16 | 0.30 | 0.23 | 0.07 | 0.18 | -0.33 | -0.04 | -0.08 | -0.38 | -0.35 | -0.30 | 0.05 | -0.12 | -0.23 | -0.14 | -0.13 | -0.10 | 0.02 | -0.20 | -0.12 | 0.00 | 0.09 |
| TCGA.46.3765.01 | 0.218 | -0.09 | -0.29 | -0.08 | -0.03 | -0.06 | -0.18 | -0.09 | -0.29 | -0.39 | -0.06 | 0.00 | -0.29 | -0.12 | -0.05 | -0.03 | -0.21 | -0.24 | 0.14 | -0.04 | -0.17 | -0.19 | -0.12 | -0.16 | -0.23 | -0.11 | -0.09 | -0.10 | 0.05 |
| TCGA.46.3766.01 | 0.267 | 0.34 | 0.23 | 0.17 | 0.12 | 0.32 | 0.34 | 0.23 | -0.12 | 0.11 | 0.38 | 0.23 | 0.23 | 0.09 | 0.37 | 0.34 | 0.24 | 0.15 | 0.19 | 0.22 | 0.33 | 0.04 | 0.24 | 0.26 | 0.27 | 0.31 | 0.37 | -0.08 | -0.27 |
| TCGA.46.3768.01 | 0.483 | -0.05 | -0.09 | -0.12 | 0.18 | -0.15 | -0.11 | -0.01 | 0.06 | 0.06 | -0.23 | 0.01 | -0.09 | -0.03 | 0.02 | -0.29 | -0.40 | -0.28 | 0.03 | 0.02 | -0.25 | 0.28 | -0.03 | 0.17 | -0.19 | -0.08 | -0.17 | 0.31 | 0.28 |
| TCGA.46.3769.01 | 0.367 | 0.48 | -0.17 | 0.00 | -0.03 | -0.06 | -0.10 | -0.15 | -0.17 | -0.25 | -0.13 | 0.05 | -0.17 | 0.06 | 0.08 | 0.13 | 0.06 | 0.07 | -0.03 | 0.13 | -0.18 | 0.15 | -0.05 | 0.15 | -0.17 | -0.12 | 0.03 | 0.03 | 0.30 |
| TCGA.46.6025.01 | -0.108 | -0.39 | 0.45 | 0.14 | -0.10 | 0.23 | 0.13 | -0.02 | -0.14 | 0.26 | 0.04 | -0.14 | 0.45 | -0.15 | 0.06 | 0.36 | 0.40 | 0.25 | -0.01 | -0.01 | 0.32 | -0.09 | 0.20 | -0.10 | 0.31 | 0.19 | 0.18 | -0.20 | -0.46 |
| TCGA.46.6026.01 | -0.460 | -0.25 | 0.20 | 0.10 | -0.01 | 0.13 | 0.07 | 0.01 | -0.37 | -0.06 | 0.05 | 0.10 | 0.20 | -0.09 | 0.07 | 0.21 | 0.20 | 0.06 | 0.08 | 0.07 | 0.26 | -0.04 | -0.06 | 0.02 | 0.12 | 0.15 | 0.14 | -0.22 | -0.30 |
| TCGA.49.4486.01 | 0.015 | -0.40 | -0.30 | -0.22 | -0.08 | -0.28 | -0.29 | -0.24 | -0.23 | -0.33 | -0.32 | -0.21 | -0.30 | -0.10 | -0.27 | -0.37 | -0.36 | -0.31 | -0.28 | -0.22 | -0.33 | -0.24 | -0.35 | -0.07 | -0.24 | -0.23 | -0.35 | 0.00 | 0.24 |
| TCGA.49.4487.01 | 0.336 | 0.23 | 0.17 | 0.03 | 0.05 | -0.02 | 0.00 | -0.24 | -0.13 | -0.23 | -0.28 | -0.22 | 0.17 | 0.00 | -0.10 | 0.22 | 0.25 | 0.00 | 0.08 | 0.01 | -0.02 | 0.15 | -0.03 | 0.05 | -0.15 | 0.00 | 0.02 | 0.05 | 0.23 |
| TCGA.49.4488.01 | -0.318 | 0.30 | -0.02 | 0.36 | 0.23 | 0.32 | 0.31 | 0.03 | 0.04 | 0.11 | 0.22 | 0.13 | -0.02 | 0.26 | 0.35 | 0.45 | 0.35 | 0.48 | 0.26 | 0.23 | 0.31 | 0.20 | 0.26 | 0.33 | 0.34 | 0.35 | 0.14 | 0.20 | 0.05 |
| TCGA.49.4490.01 | 0.252 | 0.16 | 0.05 | 0.21 | 0.14 | 0.04 | 0.00 | -0.09 | 0.27 | -0.08 | -0.08 | -0.06 | 0.05 | 0.30 | -0.01 | 0.05 | 0.00 | 0.16 | 0.09 | 0.03 | 0.00 | -0.09 | 0.14 | 0.19 | 0.03 | 0.01 | 0.02 | 0.06 | 0.10 |
| TCGA.49.4501.01 | 0.448 | 0.21 | -0.10 | 0.05 | 0.06 | 0.00 | 0.01 | -0.03 | -0.23 | 0.07 | -0.08 | -0.02 | -0.10 | 0.02 | 0.04 | 0.03 | -0.11 | -0.02 | -0.14 | 0.04 | -0.06 | 0.00 | -0.20 | 0.21 | 0.00 | 0.08 | -0.08 | -0.01 | 0.12 |
| TCGA.49.4505.01 | -0.315 | -0.02 | 0.17 | -0.15 | 0.00 | -0.07 | -0.04 | -0.12 | -0.07 | -0.12 | -0.07 | 0.04 | 0.17 | 0.10 | -0.15 | -0.14 | 0.18 | 0.08 | 0.16 | -0.08 | -0.05 | 0.12 | -0.06 | -0.08 | -0.11 | -0.28 | -0.20 | -0.02 | 0.10 |
| TCGA.49.4506.01 | 0.294 | -0.26 | 0.02 | -0.19 | -0.35 | -0.26 | -0.25 | -0.16 | -0.29 | -0.49 | -0.25 | -0.22 | 0.02 | 0.05 | -0.09 | -0.21 | 0.10 | 0.05 | 0.05 | -0.11 | -0.16 | -0.27 | -0.22 | -0.01 | -0.28 | -0.07 | -0.13 | 0.00 | 0.21 |
| TCGA.49.4507.01 | -0.266 | -0.47 | -0.26 | -0.25 | -0.37 | -0.21 | -0.25 | -0.19 | -0.18 | -0.43 | -0.22 | -0.22 | -0.26 | -0.25 | -0.23 | -0.18 | 0.21 | -0.18 | -0.13 | -0.18 | -0.08 | -0.37 | -0.22 | -0.31 | -0.18 | -0.22 | -0.21 | -0.30 | -0.11 |
| TCGA.49.4510.01 | -0.144 | 0.01 | -0.06 | -0.15 | 0.06 | -0.19 | -0.14 | 0.04 | 0.07 | -0.08 | -0.06 | 0.06 | -0.06 | 0.06 | -0.15 | -0.11 | -0.29 | -0.13 | 0.25 | 0.09 | -0.28 | -0.05 | -0.18 | 0.00 | -0.09 | -0.25 | -0.18 | 0.12 | 0.27 |
| TCGA.49.4512.01 | -0.419 | -0.30 | 0.11 | -0.19 | -0.33 | -0.19 | -0.19 | -0.30 | -0.15 | -0.28 | -0.28 | -0.27 | 0.11 | -0.21 | -0.17 | 0.15 | 0.28 | 0.08 | -0.32 | -0.21 | -0.21 | -0.16 | -0.08 | -0.39 | -0.16 | -0.30 | -0.12 | -0.37 | -0.26 |
| TCGA.49.4514.01 | -0.191 | 0.32 | 0.28 | 0.20 | 0.33 | 0.17 | 0.23 | 0.21 | 0.03 | 0.30 | 0.24 | 0.25 | 0.28 | 0.19 | 0.28 | -0.08 | -0.03 | 0.02 | 0.25 | 0.28 | 0.09 | 0.23 | 0.23 | 0.20 | 0.09 | 0.24 | 0.14 | 0.27 | 0.19 |
| TCGA.49.6743.01 | -0.001 | -0.17 | -0.17 | -0.24 | -0.18 | -0.25 | -0.22 | -0.12 | -0.07 | -0.29 | -0.12 | -0.06 | -0.17 | -0.15 | -0.04 | -0.30 | -0.25 | -0.05 | 0.07 | 0.02 | -0.30 | -0.19 | -0.22 | 0.06 | -0.16 | -0.21 | -0.21 | 0.03 | 0.22 |
| TCGA.49.6744.01 | 0.080 | 0.44 | 0.18 | 0.17 | 0.13 | 0.17 | 0.26 | 0.17 | 0.16 | 0.28 | 0.14 | 0.09 | 0.18 | 0.10 | 0.20 | 0.21 | 0.06 | 0.08 | 0.10 | 0.05 | 0.33 | 0.17 | 0.20 | 0.25 | 0.27 | 0.25 | 0.26 | -0.06 | -0.19 |
| TCGA.49.6745.01 | -0.175 | 0.56 | 0.18 | 0.19 | 0.30 | 0.27 | 0.34 | 0.27 | 0.21 | 0.33 | 0.23 | 0.30 | 0.18 | 0.09 | 0.29 | 0.40 | 0.30 | 0.25 | 0.13 | 0.26 | 0.23 | 0.28 | 0.19 | 0.35 | 0.27 | 0.21 | 0.30 | 0.17 | -0.05 |
| TCGA.49.6761.01 | 0.099 | -0.05 | -0.14 | -0.06 | 0.10 | -0.09 | -0.15 | -0.29 | -0.05 | 0.01 | -0.27 | -0.22 | -0.14 | 0.03 | -0.14 | -0.03 | 0.06 | -0.02 | 0.01 | 0.09 | -0.26 | -0.07 | -0.19 | 0.03 | -0.27 | -0.22 | -0.13 | 0.03 | 0.11 |
| TCGA.49.AAQV.01 | -0.189 | -0.46 | -0.05 | -0.25 | -0.26 | -0.21 | -0.26 | -0.40 | -0.20 | -0.11 | -0.52 | -0.45 | -0.05 | -0.13 | -0.39 | -0.18 | 0.09 | -0.09 | -0.21 | -0.32 | -0.24 | -0.07 | -0.26 | -0.29 | -0.20 | -0.32 | -0.34 | -0.25 | 0.28 |
| TCGA.49.AAR0.01 | 0.258 | 0.17 | 0.00 | 0.05 | 0.28 | -0.08 | -0.03 | 0.10 | 0.02 | 0.18 | 0.06 | 0.19 | 0.00 | 0.14 | 0.16 | -0.11 | -0.23 | 0.21 | 0.20 | 0.18 | -0.31 | 0.06 | -0.01 | 0.22 | -0.02 | 0.02 | 0.04 | 0.39 | 0.21 |
| TCGA.49.AAR3.01 | 0.080 | 0.23 | -0.49 | -0.06 | 0.23 | -0.12 | -0.07 | -0.06 | 0.00 | 0.01 | -0.08 | 0.00 | -0.49 | 0.20 | -0.12 | 0.01 | -0.06 | 0.07 | 0.06 | 0.09 | -0.32 | 0.00 | -0.15 | 0.13 | -0.21 | -0.29 | -0.11 | 0.03 | 0.23 |
| TCGA.49.AAR4.01 | -0.517 | -0.31 | 0.27 | -0.09 | -0.20 | -0.03 | -0.08 | -0.23 | -0.10 | -0.27 | -0.37 | -0.25 | 0.27 | 0.01 | -0.17 | 0.11 | 0.29 | 0.00 | -0.31 | -0.28 | 0.08 | -0.05 | 0.03 | -0.22 | 0.00 | -0.21 | -0.09 | -0.34 | -0.29 |
| TCGA.49.AAR9.01 | 0.431 | 0.50 | -0.36 | 0.07 | 0.25 | 0.08 | 0.06 | -0.03 | -0.02 | 0.14 | 0.15 | 0.09 | -0.36 | 0.23 | -0.07 | 0.00 | 0.01 | 0.00 | 0.27 | 0.20 | -0.18 | 0.24 | 0.10 | 0.11 | -0.05 | -0.08 | 0.14 | 0.22 | 0.22 |
| TCGA.49.AARE.01 | 0.077 | 0.18 | -0.27 | 0.06 | -0.02 | 0.06 | -0.06 | 0.20 | 0.09 | 0.23 | 0.33 | 0.19 | -0.27 | 0.16 | 0.14 | 0.07 | 0.27 | -0.02 | 0.14 | 0.05 | -0.04 | 0.18 | 0.06 | 0.04 | -0.22 | 0.02 | -0.02 | 0.04 | 0.02 |
| TCGA.49.AARN.01 | -0.707 | 0.38 | 0.33 | 0.15 | 0.15 | 0.17 | 0.10 | -0.09 | -0.18 | 0.19 | 0.02 | 0.02 | 0.33 | -0.04 | 0.07 | 0.37 | 0.37 | 0.34 | 0.08 | 0.14 | 0.21 | 0.29 | 0.20 | 0.00 | 0.19 | 0.05 | 0.19 | -0.10 | -0.24 |
| TCGA.49.AARO.01 | -0.403 | -0.39 | -0.14 | -0.21 | -0.19 | -0.06 | -0.20 | -0.12 | 0.13 | -0.10 | -0.29 | -0.20 | -0.14 | -0.30 | -0.14 | 0.04 | 0.16 | -0.14 | -0.32 | -0.21 | -0.09 | -0.20 | -0.17 | -0.23 | -0.04 | -0.16 | -0.22 | -0.19 | -0.17 |
| TCGA.49.AARQ.01 | -0.401 | -0.03 | -0.26 | -0.05 | -0.04 | -0.10 | -0.26 | -0.24 | -0.34 | -0.01 | -0.14 | -0.18 | -0.26 | 0.11 | -0.09 | 0.13 | 0.32 | 0.33 | 0.02 | 0.21 | -0.30 | 0.03 | -0.12 | -0.03 | -0.08 | -0.23 | -0.11 | 0.11 | -0.07 |
| TCGA.49.AARR.01 | 0.091 | -0.36 | 0.28 | -0.11 | -0.01 | -0.20 | -0.19 | -0.09 | -0.25 | 0.12 | 0.03 | -0.11 | 0.28 | 0.12 | 0.04 | -0.15 | 0.01 | 0.00 | -0.14 | -0.04 | -0.16 | 0.12 | 0.11 | -0.03 | -0.20 | -0.13 | -0.13 | 0.05 | 0.23 |
| TCGA.4B.A93V.01 | 0.209 | -0.43 | -0.06 | -0.22 | -0.27 | -0.27 | -0.23 | -0.22 | 0.01 | -0.30 | -0.14 | -0.28 | -0.06 | -0.06 | -0.09 | -0.31 | -0.13 | -0.09 | 0.04 | -0.12 | -0.17 | -0.28 | -0.23 | -0.15 | -0.31 | -0.08 | -0.22 | 0.11 | 0.34 |
| TCGA.50.5044.01 | -0.418 | -0.37 | -0.03 | -0.21 | -0.17 | -0.23 | -0.24 | -0.07 | -0.15 | -0.14 | -0.29 | -0.21 | -0.03 | -0.14 | -0.04 | -0.25 | -0.02 | -0.12 | -0.06 | -0.08 | -0.21 | -0.05 | -0.18 | -0.20 | -0.22 | -0.17 | -0.22 | -0.06 | -0.05 |
| TCGA.50.5045.01 | -0.188 | -0.52 | -0.24 | 0.03 | -0.35 | 0.05 | 0.05 | -0.15 | -0.25 | -0.36 | -0.07 | -0.24 | -0.24 | 0.03 | 0.06 | 0.18 | 0.33 | 0.19 | -0.12 | -0.14 | 0.11 | -0.30 | -0.05 | -0.03 | 0.01 | 0.05 | 0.05 | -0.38 | -0.03 |
| TCGA.50.5049.01 | 0.219 | 0.08 | -0.09 | 0.15 | -0.27 | 0.20 | 0.24 | 0.02 | -0.27 | -0.07 | 0.15 | 0.01 | -0.09 | 0.06 | 0.24 | 0.30 | 0.39 | 0.24 | -0.01 | -0.03 | 0.20 | -0.10 | 0.09 | 0.10 | 0.17 | 0.20 | 0.24 | -0.23 | -0.20 |
| TCGA.50.5051.01 | 0.530 | 0.29 | 0.09 | -0.07 | 0.12 | -0.07 | -0.10 | 0.08 | 0.05 | -0.09 | -0.18 | -0.02 | 0.09 | 0.00 | -0.05 | -0.05 | -0.17 | -0.16 | -0.07 | 0.00 | -0.13 | 0.17 | -0.09 | 0.27 | -0.10 | 0.09 | -0.12 | 0.17 | 0.17 |
| TCGA.50.5055.01 | -0.216 | -0.51 | -0.01 | 0.10 | -0.28 | 0.14 | 0.16 | 0.02 | -0.34 | -0.11 | 0.09 | -0.07 | -0.01 | -0.20 | 0.07 | 0.28 | 0.38 | 0.20 | -0.17 | -0.16 | 0.36 | -0.24 | 0.06 | -0.11 | 0.23 | 0.26 | 0.20 | -0.42 | -0.32 |
| TCGA.50.5066.01 | -0.220 | -0.10 | 0.36 | -0.04 | -0.03 | 0.02 | 0.15 | 0.14 | 0.15 | -0.14 | -0.12 | 0.08 | 0.36 | -0.07 | 0.15 | -0.16 | 0.01 | -0.17 | 0.11 | -0.03 | 0.18 | -0.12 | 0.12 | 0.03 | 0.06 | 0.00 | 0.11 | 0.08 | -0.13 |
| TCGA.50.5068.01 | 0.259 | 0.10 | -0.35 | -0.06 | -0.08 | -0.09 | -0.13 | -0.03 | -0.01 | -0.16 | 0.04 | -0.05 | -0.35 | -0.03 | -0.05 | -0.25 | -0.33 | -0.24 | -0.10 | -0.11 | -0.14 | -0.14 | -0.21 | 0.21 | -0.10 | 0.01 | -0.10 | 0.10 | 0.31 |
| TCGA.50.5072.01 | 0.215 | 0.20 | 0.05 | -0.10 | -0.12 | -0.12 | 0.00 | 0.02 | 0.19 | -0.25 | 0.25 | 0.16 | 0.05 | -0.22 | 0.00 | -0.20 | -0.21 | -0.11 | 0.03 | -0.04 | 0.03 | -0.33 | -0.25 | -0.06 | 0.13 | 0.07 | 0.09 | 0.10 | -0.05 |
| TCGA.50.5930.01 | 0.236 | -0.05 | 0.17 | 0.03 | -0.14 | 0.18 | 0.19 | 0.19 | 0.04 | -0.07 | 0.18 | 0.08 | 0.17 | -0.37 | 0.12 | 0.22 | 0.08 | -0.11 | 0.05 | 0.04 | 0.30 | -0.13 | 0.07 | -0.23 | 0.20 | 0.12 | 0.28 | -0.14 | -0.31 |
| TCGA.50.5931.01 | -0.073 | 0.35 | 0.21 | 0.24 | 0.15 | 0.19 | 0.30 | 0.40 | 0.31 | 0.18 | 0.34 | 0.40 | 0.21 | 0.20 | 0.14 | -0.02 | 0.02 | 0.12 | 0.24 | 0.09 | 0.29 | 0.04 | 0.21 | 0.00 | 0.16 | 0.10 | 0.22 | 0.01 | -0.21 |
| TCGA.50.5932.01 | 0.041 | -0.14 | -0.20 | -0.21 | -0.03 | -0.24 | -0.23 | -0.25 | -0.35 | -0.11 | -0.15 | -0.15 | -0.20 | -0.14 | -0.04 | -0.29 | -0.40 | -0.28 | -0.01 | -0.06 | -0.28 | -0.21 | -0.21 | -0.09 | -0.22 | -0.25 | -0.22 | -0.02 | 0.03 |
| TCGA.50.5933.01 | -0.325 | 0.17 | -0.17 | -0.23 | -0.12 | -0.15 | -0.14 | -0.15 | -0.09 | -0.32 | -0.38 | -0.32 | -0.17 | -0.01 | -0.10 | -0.21 | -0.09 | -0.03 | -0.02 | -0.21 | 0.00 | -0.19 | -0.23 | -0.22 | -0.12 | -0.16 | -0.10 | -0.15 | 0.15 |
| TCGA.50.5935.01 | -0.362 | -0.27 | 0.17 | 0.14 | -0.08 | 0.14 | 0.09 | 0.16 | 0.03 | 0.05 | 0.14 | 0.07 | 0.17 | -0.16 | 0.06 | 0.10 | 0.06 | -0.01 | -0.10 | -0.14 | 0.31 | -0.09 | 0.02 | 0.07 | 0.15 | 0.25 | 0.09 | -0.03 | -0.11 |
| TCGA.50.5939.01 | 0.152 | 0.14 | -0.38 | -0.06 | -0.01 | -0.07 | -0.07 | 0.12 | 0.24 | 0.34 | 0.08 | 0.21 | -0.38 | -0.04 | -0.02 | -0.24 | -0.12 | -0.19 | -0.04 | 0.07 | -0.27 | 0.11 | -0.02 | -0.04 | -0.25 | -0.12 | -0.10 | 0.15 | 0.38 |
| TCGA.50.5941.01 | 0.066 | 0.39 | 0.33 | 0.20 | 0.04 | 0.26 | 0.28 | 0.13 | -0.05 | -0.03 | -0.01 | -0.03 | 0.33 | 0.19 | 0.10 | 0.39 | 0.44 | 0.32 | 0.27 | 0.02 | 0.21 | 0.03 | 0.20 | 0.18 | 0.18 | 0.21 | 0.24 | -0.17 | -0.26 |
| TCGA.50.5942.01 | -0.272 | 0.14 | 0.26 | 0.04 | 0.01 | 0.14 | 0.08 | 0.22 | 0.10 | 0.33 | 0.20 | 0.03 | 0.26 | -0.30 | -0.02 | -0.07 | -0.20 | -0.30 | -0.25 | -0.15 | 0.30 | -0.02 | -0.02 | -0.15 | 0.19 | 0.21 | 0.03 | -0.17 | -0.31 |
| TCGA.50.5946.01 | -0.427 | 0.04 | 0.27 | -0.10 | -0.03 | -0.13 | -0.17 | -0.09 | -0.40 | 0.14 | -0.02 | -0.12 | 0.27 | -0.30 | -0.10 | -0.27 | -0.29 | -0.25 | -0.23 | -0.17 | 0.15 | 0.24 | -0.10 | -0.13 | 0.00 | 0.02 | -0.14 | -0.13 | -0.18 |
| TCGA.50.6590.01 | 0.383 | 0.42 | -0.31 | -0.02 | 0.19 | -0.15 | -0.06 | 0.20 | 0.21 | 0.20 | 0.33 | 0.21 | -0.31 | -0.07 | -0.05 | -0.13 | -0.33 | -0.20 | 0.18 | 0.14 | -0.22 | 0.11 | -0.04 | 0.01 | -0.15 | -0.15 | -0.03 | 0.19 | 0.12 |
| TCGA.50.6591.01 | 0.206 | -0.16 | -0.31 | -0.16 | -0.18 | -0.22 | -0.26 | 0.02 | -0.08 | -0.17 | -0.21 | -0.10 | -0.31 | -0.03 | -0.14 | -0.02 | 0.20 | 0.11 | -0.03 | -0.16 | -0.28 | -0.19 | -0.24 | -0.09 | -0.22 | -0.29 | -0.18 | -0.17 | 0.11 |
| TCGA.50.6592.01 | 0.265 | -0.10 | 0.06 | 0.00 | -0.06 | 0.02 | -0.01 | 0.04 | 0.27 | 0.20 | -0.18 | -0.08 | 0.06 | 0.11 | 0.04 | -0.13 | -0.23 | -0.32 | -0.17 | -0.06 | 0.09 | 0.18 | 0.07 | 0.03 | -0.05 | 0.16 | -0.08 | -0.01 | 0.18 |
| TCGA.50.6593.01 | -0.207 | 0.22 | 0.40 | 0.04 | 0.06 | 0.07 | 0.20 | 0.04 | -0.06 | 0.13 | 0.00 | 0.07 | 0.40 | 0.08 | 0.01 | -0.03 | -0.03 | -0.07 | -0.03 | -0.28 | 0.38 | -0.03 | 0.21 | 0.02 | 0.24 | 0.23 | 0.19 | -0.22 | -0.15 |
| TCGA.50.6594.01 | 0.309 | 0.47 | 0.02 | 0.07 | 0.04 | 0.13 | 0.22 | 0.23 | 0.11 | 0.33 | 0.33 | 0.25 | 0.02 | -0.19 | 0.17 | 0.00 | -0.26 | -0.21 | -0.12 | 0.15 | 0.23 | 0.02 | 0.13 | -0.03 | 0.11 | 0.23 | 0.25 | 0.18 | -0.28 |
| TCGA.50.6595.01 | 0.010 | 0.45 | 0.05 | 0.12 | 0.40 | 0.12 | 0.15 | 0.19 | 0.36 | 0.16 | 0.17 | 0.18 | 0.05 | 0.16 | 0.16 | 0.15 | -0.04 | 0.18 | 0.31 | 0.23 | 0.02 | -0.05 | 0.10 | -0.03 | 0.21 | 0.11 | 0.24 | 0.06 | -0.23 |
| TCGA.50.6597.01 | -0.244 | 0.09 | -0.10 | -0.11 | -0.14 | -0.04 | 0.02 | 0.30 | 0.33 | 0.25 | 0.17 | 0.22 | -0.10 | -0.21 | 0.10 | -0.36 | -0.39 | -0.39 | -0.25 | -0.15 | 0.15 | -0.16 | 0.00 | -0.31 | 0.25 | 0.09 | -0.02 | -0.02 | -0.19 |
| TCGA.50.6673.01 | 0.222 | -0.19 | -0.01 | -0.09 | -0.08 | -0.10 | -0.11 | -0.17 | 0.16 | -0.40 | 0.02 | -0.04 | -0.01 | -0.21 | -0.04 | -0.11 | 0.23 | 0.22 | 0.05 | -0.09 | -0.20 | -0.02 | -0.03 | -0.23 | -0.15 | -0.17 | 0.01 | -0.07 | 0.14 |
| TCGA.50.7109.01 | 0.233 | -0.04 | 0.13 | -0.08 | -0.13 | 0.00 | -0.04 | 0.00 | 0.22 | 0.26 | 0.06 | 0.04 | 0.13 | -0.13 | -0.01 | -0.01 | -0.11 | -0.05 | -0.06 | 0.06 | -0.01 | 0.02 | 0.02 | 0.06 | 0.06 | 0.14 | 0.02 | 0.12 | 0.00 |
| TCGA.50.8457.01 | 0.272 | 0.46 | -0.31 | 0.07 | 0.15 | 0.01 | 0.07 | 0.30 | 0.31 | 0.35 | 0.28 | 0.28 | -0.31 | 0.08 | 0.23 | -0.14 | -0.17 | -0.11 | 0.13 | 0.07 | -0.13 | 0.09 | 0.10 | 0.23 | -0.06 | 0.01 | 0.04 | 0.21 | 0.17 |
| TCGA.50.8460.01 | 0.100 | 0.53 | -0.03 | 0.15 | 0.03 | 0.20 | 0.21 | 0.18 | 0.19 | 0.28 | 0.21 | 0.14 | -0.03 | 0.11 | 0.27 | 0.23 | 0.11 | 0.13 | 0.13 | 0.00 | 0.21 | 0.21 | 0.22 | 0.25 | 0.20 | 0.34 | 0.24 | 0.04 | -0.20 |
| TCGA.51.4079.01 | 0.287 | 0.31 | -0.04 | 0.08 | 0.19 | -0.03 | 0.06 | 0.14 | 0.07 | -0.01 | 0.26 | 0.25 | -0.04 | 0.12 | 0.27 | 0.10 | 0.07 | 0.28 | 0.29 | 0.17 | -0.10 | -0.05 | 0.02 | 0.29 | -0.01 | 0.07 | 0.20 | 0.25 | -0.06 |
| TCGA.51.4080.01 | 0.001 | 0.25 | -0.12 | 0.01 | 0.25 | -0.09 | 0.05 | 0.08 | -0.21 | -0.15 | 0.14 | 0.17 | -0.12 | 0.07 | -0.03 | -0.19 | -0.29 | 0.12 | 0.07 | 0.14 | -0.23 | 0.01 | -0.03 | 0.18 | -0.18 | -0.07 | 0.10 | 0.19 | 0.06 |
| TCGA.51.4081.01 | -0.439 | -0.32 | -0.11 | -0.02 | -0.01 | -0.13 | -0.17 | 0.05 | -0.22 | -0.19 | -0.07 | 0.04 | -0.11 | -0.02 | -0.11 | -0.36 | -0.21 | -0.10 | -0.10 | -0.17 | -0.07 | -0.02 | -0.14 | -0.13 | -0.21 | -0.18 | -0.18 | -0.07 | -0.02 |
| TCGA.51.6867.01 | -0.271 | 0.06 | 0.33 | 0.08 | -0.15 | -0.01 | -0.04 | 0.00 | -0.09 | -0.28 | -0.15 | -0.07 | 0.33 | 0.01 | -0.20 | 0.36 | 0.39 | 0.40 | 0.11 | 0.01 | 0.15 | -0.10 | 0.07 | -0.20 | 0.02 | -0.16 | 0.16 | -0.28 | -0.30 |
| TCGA.52.7622.01 | 0.038 | -0.21 | 0.13 | 0.12 | -0.10 | 0.18 | 0.09 | 0.16 | 0.05 | 0.12 | 0.14 | 0.21 | 0.13 | -0.05 | 0.15 | 0.27 | 0.17 | -0.01 | -0.03 | 0.00 | 0.25 | 0.21 | 0.06 | 0.09 | 0.26 | 0.25 | 0.08 | -0.07 | -0.26 |
| TCGA.52.7810.01 | -0.294 | -0.17 | 0.38 | 0.04 | -0.21 | 0.15 | 0.18 | 0.07 | -0.08 | -0.18 | 0.13 | 0.05 | 0.38 | -0.36 | 0.04 | 0.22 | 0.01 | 0.04 | -0.04 | -0.05 | 0.39 | -0.18 | 0.02 | -0.13 | 0.22 | 0.19 | 0.15 | -0.32 | -0.37 |
| TCGA.52.7811.01 | 0.206 | 0.14 | -0.08 | 0.09 | 0.28 | 0.04 | 0.10 | 0.08 | 0.19 | -0.10 | 0.14 | 0.13 | -0.08 | 0.18 | 0.11 | 0.05 | 0.16 | 0.31 | 0.34 | 0.18 | 0.01 | -0.02 | 0.10 | 0.28 | -0.04 | 0.13 | 0.23 | 0.25 | 0.30 |
| TCGA.52.7812.01 | 0.415 | -0.19 | 0.16 | -0.11 | -0.10 | -0.11 | -0.19 | -0.13 | 0.16 | -0.10 | -0.12 | -0.13 | 0.16 | -0.11 | 0.00 | -0.14 | -0.12 | -0.02 | 0.17 | 0.02 | -0.14 | 0.11 | -0.01 | 0.10 | 0.02 | 0.17 | -0.10 | 0.28 | 0.21 |
| TCGA.53.7624.01 | 0.222 | 0.52 | -0.08 | -0.03 | -0.07 | -0.11 | -0.01 | 0.10 | 0.11 | -0.07 | 0.20 | 0.13 | -0.08 | -0.32 | -0.09 | -0.09 | -0.01 | -0.03 | 0.05 | -0.01 | -0.06 | -0.12 | -0.13 | -0.09 | -0.01 | 0.01 | 0.09 | 0.04 | -0.09 |
| TCGA.53.7626.01 | -0.497 | 0.00 | 0.03 | -0.13 | -0.08 | -0.15 | -0.09 | -0.18 | -0.01 | -0.18 | -0.20 | -0.27 | 0.03 | -0.21 | 0.02 | -0.14 | -0.45 | -0.45 | -0.10 | -0.11 | 0.09 | 0.04 | -0.02 | -0.13 | 0.09 | 0.11 | -0.12 | 0.00 | -0.09 |
| TCGA.53.7813.01 | 0.338 | 0.01 | -0.11 | -0.11 | 0.15 | -0.17 | -0.20 | -0.02 | -0.19 | -0.07 | 0.09 | 0.03 | -0.11 | 0.07 | 0.00 | -0.24 | -0.07 | -0.08 | -0.01 | 0.07 | -0.28 | 0.02 | -0.03 | 0.10 | -0.26 | -0.12 | -0.05 | 0.22 | 0.35 |
| TCGA.55.1592.01 | 0.485 | -0.31 | -0.24 | -0.30 | -0.24 | -0.27 | -0.18 | -0.23 | -0.17 | -0.14 | -0.33 | -0.29 | -0.24 | -0.24 | -0.42 | -0.37 | -0.35 | -0.31 | -0.34 | -0.34 | -0.43 | -0.40 | -0.36 | -0.27 | -0.30 | -0.43 | -0.36 | -0.22 | 0.02 |
| TCGA.55.1596.01 | 0.142 | -0.31 | 0.08 | -0.12 | -0.01 | -0.19 | -0.23 | -0.15 | 0.00 | -0.18 | -0.23 | -0.07 | 0.08 | -0.19 | -0.12 | -0.35 | -0.21 | -0.16 | -0.07 | 0.03 | -0.15 | 0.14 | -0.16 | -0.04 | -0.04 | -0.18 | -0.28 | 0.11 | 0.35 |
| TCGA.55.5899.01 | 0.445 | -0.32 | 0.05 | -0.06 | -0.22 | -0.07 | 0.01 | -0.05 | -0.07 | -0.17 | 0.06 | -0.03 | 0.05 | -0.21 | -0.01 | -0.33 | -0.21 | -0.27 | -0.15 | -0.11 | 0.14 | -0.06 | -0.03 | -0.07 | -0.03 | 0.13 | -0.01 | -0.02 | 0.17 |
| TCGA.55.6543.01 | 0.322 | -0.17 | -0.01 | 0.05 | 0.07 | 0.03 | 0.04 | -0.32 | -0.10 | -0.26 | -0.17 | -0.16 | -0.01 | 0.23 | -0.17 | 0.02 | 0.27 | 0.24 | 0.15 | -0.09 | -0.28 | 0.13 | 0.04 | 0.18 | -0.11 | -0.17 | -0.04 | 0.07 | 0.27 |
| TCGA.55.6642.01 | 0.554 | 0.08 | 0.06 | -0.04 | 0.27 | -0.15 | 0.03 | -0.03 | 0.12 | 0.09 | -0.12 | 0.01 | 0.06 | 0.20 | 0.08 | -0.16 | -0.20 | -0.21 | 0.31 | 0.07 | -0.09 | 0.05 | 0.11 | 0.24 | -0.23 | -0.09 | -0.01 | 0.27 | 0.26 |
| TCGA.55.6712.01 | -0.492 | 0.29 | 0.24 | 0.23 | 0.16 | 0.29 | 0.25 | 0.07 | -0.14 | 0.26 | 0.02 | 0.24 | 0.24 | 0.21 | -0.01 | 0.28 | 0.37 | 0.20 | 0.19 | 0.20 | 0.28 | -0.08 | 0.11 | 0.19 | 0.22 | 0.14 | 0.31 | -0.12 | -0.28 |
| TCGA.55.6968.01 | -0.218 | -0.03 | 0.29 | 0.05 | -0.06 | 0.17 | 0.19 | 0.01 | 0.04 | 0.23 | -0.07 | -0.07 | 0.29 | -0.12 | 0.16 | 0.19 | -0.10 | -0.15 | -0.24 | -0.12 | 0.38 | 0.07 | 0.12 | -0.04 | 0.31 | 0.24 | 0.06 | -0.10 | -0.33 |
| TCGA.55.6970.01 | 0.183 | 0.01 | 0.00 | -0.01 | 0.00 | 0.01 | -0.09 | 0.05 | 0.06 | 0.11 | -0.05 | -0.01 | 0.00 | -0.14 | 0.03 | 0.06 | 0.10 | 0.11 | 0.02 | 0.06 | -0.12 | 0.16 | -0.13 | 0.11 | -0.10 | 0.09 | 0.03 | 0.12 | 0.08 |
| TCGA.55.6971.01 | -0.100 | -0.15 | -0.25 | -0.20 | 0.00 | -0.21 | -0.20 | 0.16 | -0.31 | 0.12 | 0.03 | 0.11 | -0.25 | -0.11 | -0.24 | -0.05 | -0.17 | -0.04 | -0.06 | -0.12 | -0.23 | -0.19 | -0.15 | -0.23 | -0.12 | -0.35 | -0.05 | -0.08 | 0.12 |
| TCGA.55.6972.01 | 0.051 | 0.32 | 0.22 | 0.34 | 0.17 | 0.41 | 0.44 | 0.32 | 0.27 | 0.52 | 0.31 | 0.33 | 0.22 | 0.17 | 0.39 | 0.38 | 0.18 | 0.23 | 0.00 | 0.20 | 0.41 | 0.27 | 0.41 | 0.26 | 0.38 | 0.42 | 0.33 | 0.15 | -0.19 |
| TCGA.55.6975.01 | -0.330 | 0.29 | -0.10 | 0.04 | -0.07 | 0.17 | 0.23 | 0.29 | 0.20 | -0.06 | 0.40 | 0.39 | -0.10 | -0.11 | 0.33 | -0.03 | -0.16 | -0.05 | 0.06 | 0.07 | 0.30 | -0.24 | 0.13 | 0.06 | 0.18 | 0.33 | 0.32 | -0.03 | -0.21 |
| TCGA.55.6978.01 | -0.407 | 0.52 | -0.22 | 0.15 | 0.21 | 0.15 | 0.21 | -0.02 | 0.04 | 0.03 | 0.22 | 0.16 | -0.22 | 0.15 | 0.19 | 0.38 | 0.42 | 0.38 | 0.21 | 0.23 | 0.07 | 0.22 | 0.11 | 0.17 | 0.03 | 0.15 | 0.25 | 0.00 | -0.09 |
| TCGA.55.6979.01 | 0.291 | 0.18 | 0.04 | -0.16 | 0.08 | -0.07 | 0.00 | -0.08 | 0.09 | 0.11 | -0.08 | -0.09 | 0.04 | -0.04 | -0.08 | -0.12 | -0.25 | -0.24 | 0.01 | 0.14 | -0.22 | -0.01 | 0.16 | -0.04 | -0.16 | -0.18 | 0.00 | 0.02 | -0.13 |
| TCGA.55.6980.01 | -0.251 | -0.24 | 0.35 | 0.14 | -0.08 | 0.22 | 0.22 | -0.12 | -0.13 | -0.04 | -0.11 | -0.18 | 0.35 | 0.01 | 0.16 | 0.08 | -0.03 | -0.13 | -0.12 | -0.12 | 0.31 | 0.21 | 0.23 | 0.12 | 0.33 | 0.32 | -0.04 | -0.05 | -0.12 |
| TCGA.55.6981.01 | -0.083 | -0.38 | 0.27 | -0.23 | 0.04 | -0.14 | -0.17 | -0.14 | -0.14 | -0.10 | -0.32 | -0.15 | 0.27 | -0.17 | -0.11 | -0.30 | -0.23 | -0.24 | 0.02 | -0.15 | -0.18 | -0.08 | -0.13 | -0.21 | -0.23 | -0.27 | -0.17 | -0.03 | -0.12 |
| TCGA.55.6982.01 | -0.352 | -0.02 | 0.34 | 0.13 | 0.10 | 0.18 | 0.09 | -0.10 | -0.15 | 0.07 | -0.16 | -0.18 | 0.34 | 0.11 | -0.05 | 0.42 | 0.42 | 0.35 | 0.05 | -0.05 | 0.33 | 0.13 | 0.15 | -0.10 | 0.20 | 0.08 | 0.24 | -0.26 | -0.22 |
| TCGA.55.6984.01 | 0.311 | 0.07 | -0.15 | -0.05 | 0.14 | -0.10 | 0.00 | 0.08 | 0.33 | -0.14 | 0.13 | 0.14 | -0.15 | 0.08 | -0.08 | -0.10 | -0.26 | 0.01 | 0.14 | 0.06 | -0.27 | 0.20 | 0.02 | 0.14 | -0.12 | 0.07 | -0.02 | 0.27 | 0.15 |
| TCGA.55.6985.01 | 0.243 | -0.46 | 0.15 | -0.05 | -0.27 | 0.08 | 0.05 | 0.01 | 0.19 | 0.21 | -0.03 | -0.13 | 0.15 | -0.09 | 0.05 | -0.07 | 0.04 | -0.13 | -0.25 | -0.27 | 0.26 | 0.19 | 0.12 | -0.07 | 0.19 | 0.26 | -0.04 | -0.14 | -0.17 |
| TCGA.55.6986.01 | 0.534 | 0.57 | 0.15 | 0.19 | -0.06 | 0.16 | 0.28 | 0.07 | 0.11 | 0.42 | 0.12 | -0.05 | 0.15 | -0.04 | 0.01 | 0.20 | 0.26 | 0.32 | 0.11 | -0.01 | 0.12 | 0.22 | 0.17 | 0.07 | 0.07 | 0.13 | 0.22 | -0.10 | 0.00 |
| TCGA.55.6987.01 | 0.354 | -0.28 | 0.07 | 0.01 | -0.07 | 0.10 | 0.11 | -0.14 | 0.02 | -0.26 | -0.25 | -0.26 | 0.07 | -0.01 | -0.15 | -0.05 | 0.03 | -0.10 | -0.11 | -0.26 | 0.12 | -0.22 | 0.05 | -0.09 | -0.06 | -0.11 | 0.03 | -0.24 | -0.10 |
| TCGA.55.7227.01 | -0.364 | 0.12 | -0.01 | 0.15 | -0.05 | 0.11 | 0.15 | 0.18 | -0.08 | 0.22 | 0.21 | 0.17 | -0.01 | -0.04 | 0.10 | 0.21 | 0.26 | 0.02 | -0.02 | 0.00 | 0.31 | -0.03 | 0.06 | 0.09 | 0.21 | 0.17 | 0.20 | -0.19 | -0.39 |
| TCGA.55.7281.01 | -0.299 | -0.07 | 0.36 | 0.07 | -0.17 | 0.19 | 0.15 | 0.04 | -0.13 | 0.43 | -0.03 | 0.05 | 0.36 | -0.24 | 0.00 | 0.20 | 0.35 | 0.05 | -0.35 | -0.07 | 0.22 | 0.08 | -0.02 | -0.09 | 0.22 | 0.07 | 0.00 | -0.24 | -0.30 |
| TCGA.55.7283.01 | -0.223 | -0.10 | -0.05 | -0.02 | -0.01 | -0.06 | -0.04 | 0.01 | -0.09 | 0.02 | 0.02 | -0.09 | -0.05 | -0.08 | -0.17 | 0.06 | -0.19 | -0.14 | -0.22 | -0.17 | 0.06 | -0.03 | -0.14 | 0.01 | 0.12 | 0.13 | -0.08 | -0.07 | -0.17 |
| TCGA.55.7284.01 | -0.478 | 0.42 | 0.09 | 0.42 | 0.27 | 0.38 | 0.41 | 0.17 | 0.14 | 0.33 | 0.31 | 0.36 | 0.09 | 0.25 | 0.39 | 0.47 | 0.35 | 0.45 | 0.17 | 0.25 | 0.44 | 0.21 | 0.40 | 0.31 | 0.38 | 0.43 | 0.33 | 0.15 | -0.30 |
| TCGA.55.7570.01 | 0.223 | -0.18 | -0.27 | -0.30 | -0.24 | -0.30 | -0.33 | -0.01 | -0.07 | -0.11 | 0.06 | 0.03 | -0.27 | -0.22 | -0.31 | -0.43 | -0.37 | -0.39 | -0.18 | -0.11 | -0.29 | -0.32 | -0.30 | -0.30 | -0.30 | -0.30 | -0.25 | -0.06 | 0.06 |
| TCGA.55.7573.01 | -0.540 | 0.49 | 0.14 | 0.41 | 0.29 | 0.41 | 0.42 | 0.18 | 0.25 | 0.32 | 0.40 | 0.34 | 0.14 | 0.30 | 0.41 | 0.53 | 0.49 | 0.52 | 0.24 | 0.23 | 0.45 | 0.25 | 0.40 | 0.34 | 0.43 | 0.47 | 0.35 | 0.18 | -0.32 |
| TCGA.55.7574.01 | -0.091 | -0.19 | 0.35 | 0.10 | -0.11 | 0.15 | 0.00 | 0.10 | 0.17 | 0.27 | -0.06 | -0.02 | 0.35 | -0.04 | -0.04 | 0.23 | 0.42 | 0.12 | -0.30 | -0.04 | 0.17 | 0.26 | 0.13 | -0.04 | 0.19 | 0.13 | -0.04 | -0.10 | -0.30 |
| TCGA.55.7576.01 | -0.236 | 0.34 | -0.09 | 0.14 | 0.17 | 0.06 | 0.02 | 0.33 | 0.12 | 0.37 | 0.09 | 0.23 | -0.09 | 0.05 | -0.01 | 0.14 | -0.01 | 0.21 | 0.06 | 0.13 | 0.11 | -0.15 | -0.09 | 0.02 | 0.21 | 0.07 | 0.10 | 0.10 | -0.12 |
| TCGA.55.7724.01 | -0.006 | -0.08 | -0.08 | 0.02 | -0.07 | -0.05 | -0.05 | 0.02 | -0.02 | 0.23 | -0.01 | -0.03 | -0.08 | -0.01 | 0.00 | -0.15 | -0.15 | -0.29 | -0.34 | -0.05 | -0.04 | 0.08 | -0.10 | 0.02 | -0.04 | 0.04 | -0.16 | -0.02 | 0.12 |
| TCGA.55.7725.01 | -0.384 | -0.16 | 0.14 | -0.03 | -0.12 | 0.00 | -0.06 | -0.08 | -0.43 | -0.21 | -0.08 | -0.26 | 0.14 | -0.29 | -0.19 | 0.01 | 0.09 | -0.06 | -0.21 | -0.16 | 0.15 | -0.10 | -0.21 | -0.20 | 0.06 | 0.05 | -0.03 | -0.26 | -0.30 |
| TCGA.55.7726.01 | -0.269 | 0.13 | -0.29 | -0.03 | 0.13 | -0.06 | -0.10 | 0.17 | 0.26 | 0.47 | 0.12 | 0.10 | -0.29 | -0.09 | -0.07 | 0.39 | 0.38 | 0.27 | 0.12 | 0.04 | -0.18 | 0.16 | -0.04 | -0.03 | -0.06 | -0.09 | 0.04 | 0.07 | 0.06 |
| TCGA.55.7727.01 | -0.208 | -0.32 | 0.14 | -0.03 | -0.01 | -0.16 | -0.17 | -0.28 | 0.02 | -0.21 | -0.40 | -0.27 | 0.14 | 0.16 | -0.33 | 0.06 | 0.00 | -0.01 | -0.22 | -0.25 | -0.15 | 0.00 | -0.11 | -0.12 | -0.22 | -0.34 | -0.19 | -0.17 | -0.06 |
| TCGA.55.7815.01 | 0.153 | 0.05 | 0.41 | 0.29 | -0.01 | 0.34 | 0.32 | 0.18 | 0.17 | 0.28 | 0.16 | -0.01 | 0.41 | 0.20 | 0.31 | 0.43 | 0.33 | 0.22 | 0.17 | 0.20 | 0.45 | 0.03 | 0.35 | 0.12 | 0.33 | 0.37 | 0.36 | -0.07 | -0.29 |
| TCGA.55.7816.01 | 0.323 | 0.24 | -0.25 | 0.06 | 0.24 | -0.08 | -0.02 | 0.16 | 0.24 | 0.08 | 0.15 | 0.32 | -0.25 | -0.01 | 0.07 | -0.25 | -0.17 | -0.03 | 0.18 | 0.14 | -0.15 | 0.09 | 0.02 | 0.20 | -0.13 | 0.06 | 0.09 | 0.25 | 0.22 |
| TCGA.55.7903.01 | -0.014 | -0.07 | -0.02 | 0.28 | 0.01 | 0.25 | 0.34 | 0.08 | -0.10 | 0.06 | 0.21 | 0.11 | -0.02 | 0.14 | 0.40 | 0.23 | 0.09 | 0.24 | 0.06 | 0.08 | 0.36 | -0.03 | 0.21 | 0.29 | 0.37 | 0.43 | 0.26 | -0.07 | -0.22 |
| TCGA.55.7907.01 | -0.118 | 0.29 | -0.10 | 0.07 | 0.19 | 0.01 | 0.08 | -0.12 | 0.16 | -0.04 | 0.05 | 0.07 | -0.10 | 0.21 | 0.13 | 0.08 | 0.13 | 0.28 | 0.12 | 0.23 | -0.09 | 0.10 | -0.14 | 0.11 | -0.01 | 0.06 | 0.12 | 0.00 | -0.02 |
| TCGA.55.7910.01 | 0.254 | 0.21 | 0.13 | 0.31 | 0.15 | 0.29 | 0.28 | -0.06 | 0.29 | 0.27 | 0.04 | -0.13 | 0.13 | 0.26 | 0.30 | 0.43 | 0.43 | 0.37 | 0.11 | 0.18 | 0.30 | 0.36 | 0.36 | 0.25 | 0.25 | 0.36 | 0.14 | 0.24 | 0.10 |
| TCGA.55.7911.01 | -0.259 | -0.35 | 0.34 | -0.21 | -0.09 | -0.21 | -0.23 | -0.26 | -0.25 | -0.22 | -0.31 | -0.23 | 0.34 | 0.01 | -0.21 | 0.13 | 0.11 | -0.06 | -0.15 | -0.10 | -0.15 | -0.06 | -0.32 | -0.11 | -0.29 | -0.27 | -0.16 | -0.23 | 0.01 |
| TCGA.55.7913.01 | 0.041 | 0.00 | -0.13 | 0.15 | -0.18 | 0.22 | 0.28 | 0.16 | 0.11 | 0.17 | 0.20 | 0.17 | -0.13 | 0.06 | 0.15 | 0.17 | 0.13 | -0.02 | -0.02 | 0.02 | 0.37 | -0.09 | 0.28 | -0.06 | 0.25 | 0.17 | 0.26 | -0.25 | -0.22 |
| TCGA.55.7914.01 | -0.363 | -0.12 | 0.10 | -0.07 | -0.20 | -0.06 | -0.09 | -0.05 | -0.02 | 0.04 | -0.17 | -0.08 | 0.10 | -0.28 | -0.21 | -0.15 | 0.08 | -0.16 | -0.17 | -0.15 | 0.04 | 0.07 | -0.26 | -0.18 | -0.10 | 0.00 | -0.14 | -0.23 | -0.20 |
| TCGA.55.7994.01 | -0.193 | 0.45 | 0.07 | 0.01 | 0.08 | 0.11 | 0.07 | 0.31 | 0.19 | 0.34 | 0.34 | 0.30 | 0.07 | -0.02 | 0.15 | -0.06 | -0.02 | 0.01 | 0.04 | 0.13 | -0.02 | 0.11 | 0.07 | 0.02 | 0.12 | 0.00 | 0.08 | 0.01 | 0.07 |
| TCGA.55.7995.01 | -0.217 | -0.17 | 0.34 | -0.13 | -0.02 | -0.09 | -0.17 | -0.16 | 0.15 | 0.13 | -0.16 | -0.15 | 0.34 | -0.15 | -0.08 | -0.01 | 0.01 | 0.07 | 0.20 | 0.02 | -0.17 | 0.08 | -0.05 | -0.16 | -0.20 | -0.21 | -0.06 | 0.06 | 0.03 |
| TCGA.55.8085.01 | -0.002 | 0.42 | 0.22 | 0.31 | 0.34 | 0.20 | 0.23 | 0.24 | 0.12 | 0.04 | 0.25 | 0.30 | 0.22 | 0.33 | 0.24 | 0.25 | 0.32 | 0.33 | 0.40 | 0.21 | 0.15 | 0.04 | 0.29 | 0.27 | 0.22 | 0.23 | 0.33 | 0.32 | 0.18 |
| TCGA.55.8087.01 | -0.063 | 0.06 | -0.12 | 0.01 | -0.01 | -0.05 | -0.04 | -0.03 | 0.14 | 0.29 | -0.14 | -0.16 | -0.12 | -0.14 | -0.02 | -0.22 | -0.32 | -0.27 | -0.27 | -0.15 | -0.12 | 0.10 | 0.03 | 0.04 | 0.01 | 0.02 | -0.20 | 0.13 | 0.17 |
| TCGA.55.8089.01 | -0.004 | 0.31 | 0.13 | 0.00 | 0.08 | 0.11 | 0.00 | -0.17 | -0.03 | -0.02 | -0.11 | -0.16 | 0.13 | 0.06 | -0.05 | 0.31 | 0.44 | 0.27 | 0.11 | 0.28 | -0.01 | 0.18 | -0.03 | 0.14 | -0.04 | 0.00 | 0.07 | -0.16 | -0.23 |
| TCGA.55.8090.01 | -0.349 | 0.19 | -0.09 | 0.19 | 0.14 | 0.08 | 0.12 | 0.11 | 0.18 | 0.37 | 0.09 | 0.17 | -0.09 | 0.24 | -0.01 | -0.02 | 0.23 | 0.23 | 0.01 | 0.10 | 0.06 | 0.05 | 0.09 | 0.21 | 0.00 | 0.00 | 0.01 | -0.09 | 0.17 |
| TCGA.55.8091.01 | 0.254 | 0.16 | -0.08 | -0.03 | -0.18 | 0.07 | 0.06 | 0.15 | 0.17 | -0.18 | 0.05 | 0.04 | -0.08 | -0.10 | 0.05 | -0.02 | 0.08 | 0.11 | 0.04 | 0.02 | -0.06 | -0.17 | -0.03 | -0.05 | -0.06 | 0.10 | 0.18 | 0.07 | -0.02 |
| TCGA.55.8092.01 | 0.379 | 0.13 | 0.38 | 0.27 | -0.03 | 0.26 | 0.33 | 0.12 | 0.21 | 0.13 | 0.26 | 0.07 | 0.38 | 0.16 | 0.33 | 0.35 | 0.38 | 0.23 | 0.06 | 0.09 | 0.35 | -0.02 | 0.32 | 0.20 | 0.28 | 0.36 | 0.37 | 0.09 | -0.23 |
| TCGA.55.8094.01 | 0.510 | -0.37 | 0.07 | -0.19 | -0.16 | -0.32 | -0.34 | -0.36 | -0.23 | -0.44 | -0.23 | -0.37 | 0.07 | 0.07 | -0.27 | -0.40 | -0.42 | -0.39 | -0.10 | -0.10 | -0.19 | -0.25 | -0.27 | -0.32 | -0.04 | -0.14 | -0.27 | 0.05 | 0.26 |
| TCGA.55.8096.01 | 0.280 | 0.42 | -0.08 | 0.03 | 0.18 | 0.16 | 0.17 | 0.25 | 0.27 | 0.01 | 0.39 | 0.36 | -0.08 | 0.16 | 0.14 | 0.21 | 0.18 | 0.16 | 0.35 | 0.18 | 0.19 | 0.07 | 0.13 | 0.13 | 0.04 | 0.11 | 0.23 | 0.15 | -0.04 |
| TCGA.55.8097.01 | 0.368 | -0.20 | -0.09 | -0.08 | 0.02 | -0.11 | -0.23 | -0.14 | 0.02 | 0.02 | -0.28 | -0.14 | -0.09 | 0.03 | -0.04 | 0.11 | 0.08 | 0.03 | -0.13 | -0.02 | -0.13 | 0.10 | -0.16 | 0.22 | -0.21 | -0.04 | -0.16 | 0.02 | 0.35 |
| TCGA.55.8203.01 | 0.272 | -0.22 | 0.07 | -0.12 | -0.05 | -0.17 | -0.21 | -0.08 | 0.22 | -0.10 | -0.05 | -0.11 | 0.07 | 0.03 | -0.09 | -0.34 | -0.10 | -0.10 | 0.02 | 0.00 | -0.05 | -0.01 | -0.08 | 0.12 | -0.08 | 0.08 | -0.16 | 0.25 | 0.31 |
| TCGA.55.8204.01 | -0.512 | -0.28 | 0.23 | -0.03 | -0.16 | -0.06 | -0.18 | -0.25 | -0.41 | -0.14 | -0.30 | -0.27 | 0.23 | -0.20 | -0.28 | 0.22 | 0.13 | 0.08 | -0.34 | -0.14 | 0.14 | -0.08 | -0.20 | -0.29 | 0.09 | -0.09 | -0.18 | -0.27 | -0.23 |
| TCGA.55.8205.01 | 0.248 | 0.29 | 0.12 | 0.01 | 0.10 | 0.09 | 0.01 | -0.18 | -0.17 | -0.06 | -0.19 | -0.22 | 0.12 | 0.07 | 0.04 | 0.24 | 0.47 | 0.38 | 0.11 | 0.13 | 0.04 | 0.33 | 0.00 | 0.21 | -0.08 | 0.10 | 0.08 | 0.05 | -0.09 |
| TCGA.55.8206.01 | -0.266 | -0.07 | 0.29 | 0.06 | 0.08 | 0.00 | -0.13 | -0.10 | -0.05 | 0.03 | -0.22 | -0.24 | 0.29 | 0.03 | -0.15 | 0.13 | 0.26 | 0.00 | -0.07 | -0.11 | 0.00 | -0.01 | -0.01 | 0.03 | 0.02 | -0.01 | -0.17 | -0.07 | -0.08 |
| TCGA.55.8208.01 | 0.458 | -0.42 | 0.05 | -0.15 | -0.20 | -0.21 | -0.18 | -0.14 | 0.04 | -0.29 | -0.35 | -0.21 | 0.05 | -0.10 | -0.13 | 0.04 | 0.15 | -0.11 | -0.06 | -0.01 | -0.24 | 0.18 | -0.07 | 0.20 | -0.24 | -0.15 | -0.14 | 0.09 | 0.35 |
| TCGA.55.8299.01 | 0.248 | 0.07 | 0.48 | 0.17 | -0.03 | 0.22 | 0.22 | 0.05 | 0.00 | 0.15 | 0.20 | -0.09 | 0.48 | 0.00 | 0.17 | 0.40 | 0.41 | 0.34 | 0.16 | 0.14 | 0.25 | -0.02 | 0.23 | -0.03 | 0.18 | 0.29 | 0.24 | -0.05 | -0.18 |
| TCGA.55.8301.01 | -0.127 | -0.33 | -0.53 | -0.20 | -0.07 | -0.22 | -0.24 | -0.02 | 0.05 | -0.06 | 0.05 | -0.19 | -0.53 | -0.29 | -0.07 | -0.35 | -0.28 | -0.32 | -0.06 | 0.07 | -0.08 | -0.18 | -0.20 | -0.32 | -0.08 | -0.02 | -0.06 | -0.16 | -0.08 |
| TCGA.55.8302.01 | -0.301 | -0.14 | 0.34 | 0.11 | -0.23 | 0.22 | 0.22 | -0.14 | -0.23 | -0.06 | -0.14 | -0.24 | 0.34 | 0.01 | 0.04 | 0.24 | 0.38 | 0.25 | -0.10 | -0.19 | 0.26 | -0.24 | 0.21 | -0.08 | 0.22 | 0.09 | 0.16 | -0.33 | -0.36 |
| TCGA.55.8505.01 | 0.316 | 0.12 | 0.35 | 0.02 | -0.02 | 0.01 | 0.00 | 0.00 | 0.13 | -0.27 | 0.17 | 0.23 | 0.35 | -0.17 | -0.06 | -0.11 | 0.09 | 0.04 | 0.21 | -0.21 | 0.14 | -0.06 | 0.07 | -0.14 | -0.02 | -0.05 | 0.14 | -0.03 | 0.10 |
| TCGA.55.8507.01 | -0.204 | 0.11 | 0.34 | 0.21 | 0.01 | 0.32 | 0.31 | 0.06 | -0.04 | 0.42 | 0.15 | 0.02 | 0.34 | -0.03 | 0.23 | 0.22 | 0.08 | 0.11 | -0.07 | 0.18 | 0.34 | 0.31 | 0.29 | 0.21 | 0.31 | 0.33 | 0.14 | 0.15 | -0.08 |
| TCGA.55.8510.01 | -0.250 | -0.21 | 0.45 | 0.04 | -0.12 | 0.10 | 0.14 | -0.14 | 0.12 | -0.20 | -0.14 | -0.10 | 0.45 | -0.08 | -0.13 | 0.32 | 0.28 | 0.09 | -0.17 | -0.29 | 0.29 | -0.10 | 0.18 | -0.06 | 0.05 | -0.05 | 0.06 | -0.30 | -0.25 |
| TCGA.55.8511.01 | -0.431 | 0.26 | 0.35 | 0.34 | 0.28 | 0.35 | 0.38 | 0.24 | 0.08 | 0.29 | 0.19 | 0.29 | 0.35 | 0.23 | 0.34 | 0.45 | 0.33 | 0.38 | 0.02 | 0.22 | 0.40 | 0.28 | 0.37 | 0.23 | 0.40 | 0.40 | 0.27 | 0.23 | -0.19 |
| TCGA.55.8512.01 | -0.015 | 0.58 | -0.02 | 0.19 | 0.10 | 0.18 | 0.29 | 0.33 | 0.00 | 0.27 | 0.42 | 0.39 | -0.02 | 0.00 | 0.20 | 0.16 | 0.01 | 0.16 | 0.02 | 0.15 | 0.24 | 0.06 | 0.15 | 0.05 | 0.22 | 0.22 | 0.32 | -0.14 | -0.38 |
| TCGA.55.8514.01 | 0.573 | 0.51 | 0.22 | 0.10 | 0.21 | 0.03 | 0.15 | -0.17 | 0.14 | 0.07 | -0.11 | -0.11 | 0.22 | 0.13 | 0.20 | 0.32 | 0.16 | 0.34 | 0.28 | 0.20 | -0.01 | 0.23 | 0.24 | 0.26 | 0.02 | 0.12 | 0.14 | 0.17 | 0.34 |
| TCGA.55.8614.01 | -0.101 | 0.16 | 0.23 | 0.18 | 0.12 | 0.06 | 0.18 | 0.31 | 0.32 | 0.18 | 0.16 | 0.33 | 0.23 | 0.09 | -0.10 | 0.17 | 0.06 | 0.09 | -0.10 | 0.00 | 0.14 | -0.20 | 0.11 | -0.12 | 0.21 | 0.03 | 0.26 | -0.03 | -0.29 |
| TCGA.55.8615.01 | 0.245 | -0.23 | 0.16 | -0.08 | -0.16 | -0.09 | -0.09 | -0.20 | -0.20 | 0.00 | -0.33 | -0.26 | 0.16 | -0.21 | -0.17 | 0.06 | -0.03 | 0.01 | 0.00 | -0.20 | 0.09 | 0.06 | -0.07 | -0.08 | 0.00 | -0.06 | -0.17 | -0.13 | -0.10 |
| TCGA.55.8620.01 | 0.132 | -0.27 | -0.01 | -0.17 | -0.20 | -0.19 | -0.17 | -0.22 | -0.08 | -0.24 | -0.29 | -0.26 | -0.01 | 0.00 | -0.06 | -0.32 | -0.25 | -0.39 | -0.16 | -0.10 | -0.08 | 0.00 | -0.03 | -0.16 | -0.10 | -0.10 | -0.26 | 0.10 | 0.08 |
| TCGA.55.8621.01 | -0.344 | -0.30 | 0.46 | 0.12 | -0.11 | 0.27 | 0.24 | 0.04 | -0.03 | 0.32 | 0.05 | -0.01 | 0.46 | -0.05 | 0.25 | 0.34 | 0.20 | -0.01 | -0.10 | -0.18 | 0.39 | 0.20 | 0.25 | -0.03 | 0.31 | 0.29 | 0.15 | -0.18 | -0.33 |
| TCGA.55.A48X.01 | -0.278 | 0.20 | -0.19 | -0.09 | 0.12 | -0.17 | -0.11 | 0.03 | -0.01 | -0.14 | 0.07 | 0.12 | -0.19 | 0.20 | -0.17 | -0.21 | -0.08 | 0.14 | 0.37 | 0.14 | -0.27 | 0.10 | -0.20 | 0.08 | -0.24 | -0.18 | -0.12 | 0.13 | 0.33 |
| TCGA.55.A48Y.01 | 0.030 | 0.04 | 0.17 | 0.01 | 0.11 | 0.15 | 0.13 | 0.26 | 0.04 | 0.28 | 0.19 | 0.11 | 0.17 | 0.22 | 0.12 | 0.08 | 0.12 | -0.17 | -0.12 | 0.15 | 0.10 | 0.21 | 0.06 | -0.01 | 0.06 | -0.03 | -0.02 | 0.12 | -0.13 |
| TCGA.55.A48Z.01 | 0.410 | -0.13 | 0.04 | -0.06 | -0.22 | -0.07 | -0.10 | -0.10 | -0.17 | -0.14 | -0.03 | -0.20 | 0.04 | 0.00 | 0.04 | 0.02 | -0.11 | -0.08 | -0.19 | 0.12 | -0.13 | 0.16 | -0.09 | 0.04 | -0.04 | 0.11 | -0.08 | 0.08 | 0.01 |
| TCGA.55.A490.01 | 0.521 | 0.34 | -0.07 | 0.16 | 0.26 | 0.20 | 0.26 | 0.17 | 0.37 | 0.26 | 0.07 | 0.14 | -0.07 | 0.25 | 0.17 | 0.12 | -0.01 | 0.04 | 0.27 | 0.27 | 0.11 | 0.17 | 0.14 | 0.18 | 0.11 | 0.15 | 0.22 | 0.11 | 0.17 |
| TCGA.55.A491.01 | -0.218 | 0.12 | -0.27 | -0.05 | 0.12 | -0.11 | -0.15 | 0.24 | 0.19 | 0.19 | 0.17 | 0.15 | -0.27 | -0.09 | -0.13 | -0.28 | -0.34 | -0.32 | -0.02 | 0.05 | -0.27 | 0.08 | -0.10 | 0.01 | -0.10 | -0.22 | -0.23 | 0.14 | -0.04 |
| TCGA.55.A492.01 | 0.168 | -0.45 | 0.13 | -0.27 | -0.38 | -0.29 | -0.31 | -0.23 | -0.19 | -0.36 | -0.36 | -0.34 | 0.13 | -0.17 | -0.31 | -0.35 | -0.24 | -0.40 | -0.32 | -0.32 | -0.06 | -0.35 | -0.32 | -0.24 | -0.20 | -0.17 | -0.28 | -0.16 | -0.03 |
| TCGA.55.A494.01 | -0.016 | -0.29 | 0.08 | -0.03 | -0.02 | -0.09 | -0.10 | -0.01 | 0.20 | 0.00 | -0.10 | -0.02 | 0.08 | 0.20 | -0.06 | -0.28 | -0.24 | -0.13 | -0.15 | -0.13 | -0.07 | 0.23 | 0.03 | 0.08 | -0.06 | 0.08 | -0.15 | 0.04 | 0.18 |
| TCGA.55.A4DF.01 | 0.250 | 0.08 | -0.23 | -0.11 | -0.03 | -0.25 | -0.25 | 0.05 | -0.09 | -0.29 | 0.02 | 0.00 | -0.23 | -0.14 | -0.24 | -0.30 | -0.11 | -0.05 | -0.02 | 0.02 | -0.26 | -0.25 | -0.22 | -0.13 | -0.24 | -0.17 | -0.19 | -0.21 | 0.05 |
| TCGA.55.A4DG.01 | -0.535 | -0.24 | 0.10 | -0.14 | -0.15 | -0.13 | -0.17 | -0.11 | -0.11 | 0.13 | -0.21 | -0.23 | 0.10 | -0.20 | -0.24 | 0.03 | -0.07 | -0.26 | -0.35 | -0.24 | 0.00 | -0.03 | -0.07 | -0.28 | -0.14 | -0.15 | -0.18 | -0.23 | -0.13 |
| TCGA.55.A57B.01 | -0.171 | 0.25 | 0.20 | 0.20 | 0.20 | 0.15 | 0.16 | -0.04 | 0.20 | 0.31 | -0.07 | 0.04 | 0.20 | -0.02 | -0.04 | 0.26 | 0.06 | 0.15 | -0.16 | -0.03 | 0.13 | 0.15 | 0.10 | 0.04 | 0.13 | 0.05 | 0.10 | -0.08 | 0.01 |
| TCGA.56.1622.01 | -0.087 | -0.12 | 0.45 | 0.14 | 0.16 | 0.22 | 0.17 | -0.01 | 0.27 | 0.12 | -0.03 | -0.07 | 0.45 | 0.13 | 0.26 | 0.33 | 0.33 | 0.08 | 0.00 | 0.24 | 0.17 | 0.36 | 0.24 | 0.16 | 0.17 | 0.12 | 0.08 | 0.04 | -0.03 |
| TCGA.56.5897.01 | -0.235 | 0.21 | 0.30 | 0.18 | -0.01 | 0.24 | 0.29 | 0.17 | -0.03 | 0.20 | 0.17 | 0.07 | 0.30 | -0.15 | 0.14 | 0.33 | 0.27 | 0.22 | 0.12 | -0.03 | 0.37 | -0.08 | 0.21 | -0.08 | 0.33 | 0.24 | 0.26 | -0.26 | -0.39 |
| TCGA.56.5898.01 | 0.621 | -0.07 | -0.20 | -0.22 | -0.21 | -0.18 | -0.14 | 0.08 | -0.05 | -0.15 | 0.04 | -0.02 | -0.20 | -0.20 | -0.02 | -0.15 | -0.25 | -0.32 | -0.16 | -0.22 | -0.16 | -0.26 | -0.19 | -0.21 | -0.23 | -0.20 | -0.06 | -0.11 | -0.23 |
| TCGA.56.6545.01 | -0.314 | 0.17 | -0.11 | 0.01 | 0.12 | 0.02 | -0.02 | 0.18 | 0.00 | 0.12 | 0.15 | 0.25 | -0.11 | 0.03 | -0.04 | 0.19 | 0.30 | 0.34 | 0.07 | 0.07 | 0.07 | 0.02 | 0.10 | -0.03 | -0.02 | 0.07 | 0.14 | 0.03 | -0.27 |
| TCGA.56.6546.01 | 0.495 | 0.50 | -0.23 | 0.13 | 0.22 | 0.01 | 0.06 | 0.04 | 0.34 | -0.03 | 0.07 | 0.13 | -0.23 | 0.29 | 0.12 | -0.08 | -0.23 | -0.02 | 0.18 | 0.13 | -0.09 | 0.04 | 0.00 | 0.21 | -0.07 | 0.17 | 0.08 | 0.29 | 0.31 |
| TCGA.56.7221.01 | -0.477 | -0.12 | -0.02 | -0.16 | 0.01 | -0.20 | -0.24 | -0.02 | 0.04 | 0.14 | 0.05 | 0.06 | -0.02 | -0.08 | -0.27 | -0.31 | -0.31 | -0.30 | 0.01 | -0.14 | -0.33 | 0.04 | -0.19 | 0.03 | -0.12 | -0.40 | -0.26 | 0.07 | 0.05 |
| TCGA.56.7222.01 | 0.346 | 0.42 | 0.21 | 0.19 | 0.20 | 0.26 | 0.27 | 0.23 | 0.10 | 0.13 | 0.33 | 0.27 | 0.21 | 0.12 | 0.22 | 0.25 | 0.10 | 0.03 | 0.37 | 0.34 | 0.16 | 0.23 | 0.18 | 0.28 | 0.12 | 0.24 | 0.22 | 0.25 | 0.05 |
| TCGA.56.7223.01 | 0.084 | 0.10 | -0.29 | -0.23 | 0.04 | -0.23 | -0.24 | 0.16 | 0.34 | 0.34 | 0.05 | 0.07 | -0.29 | -0.15 | -0.23 | -0.42 | -0.51 | -0.43 | -0.14 | -0.10 | -0.38 | -0.11 | -0.20 | -0.21 | -0.29 | -0.18 | -0.22 | 0.17 | 0.34 |
| TCGA.56.7579.01 | 0.254 | -0.28 | -0.28 | -0.06 | 0.01 | -0.12 | -0.09 | -0.01 | 0.25 | -0.07 | -0.07 | 0.10 | -0.28 | 0.18 | -0.25 | -0.36 | -0.33 | -0.18 | 0.09 | -0.01 | -0.20 | -0.07 | -0.08 | -0.12 | -0.06 | -0.26 | -0.14 | 0.00 | 0.01 |
| TCGA.56.7580.01 | -0.379 | -0.17 | -0.38 | -0.24 | 0.08 | -0.25 | -0.25 | -0.13 | 0.06 | 0.24 | -0.11 | -0.03 | -0.38 | -0.28 | -0.13 | -0.37 | -0.48 | -0.33 | -0.10 | -0.14 | -0.39 | -0.10 | -0.30 | -0.23 | -0.17 | -0.25 | -0.30 | 0.18 | 0.27 |
| TCGA.56.7582.01 | 0.341 | 0.16 | 0.04 | 0.03 | 0.01 | -0.01 | -0.07 | -0.04 | -0.02 | -0.15 | -0.06 | -0.03 | 0.04 | 0.13 | 0.12 | -0.27 | -0.14 | -0.17 | -0.09 | 0.12 | -0.07 | 0.02 | -0.05 | -0.03 | -0.07 | -0.09 | 0.04 | 0.12 | 0.12 |
| TCGA.56.7730.01 | -0.087 | 0.18 | 0.20 | 0.10 | -0.15 | 0.14 | 0.13 | 0.01 | 0.20 | 0.32 | -0.16 | -0.06 | 0.20 | -0.10 | -0.11 | -0.07 | -0.09 | 0.03 | -0.15 | -0.23 | 0.11 | -0.10 | 0.19 | -0.14 | 0.16 | -0.13 | 0.11 | -0.25 | -0.22 |
| TCGA.56.7731.01 | 0.387 | 0.45 | 0.07 | 0.27 | 0.24 | 0.19 | 0.31 | 0.32 | 0.03 | 0.18 | 0.26 | 0.26 | 0.07 | 0.33 | 0.29 | 0.37 | 0.31 | 0.40 | 0.42 | 0.35 | 0.31 | -0.10 | 0.28 | 0.30 | 0.32 | 0.39 | 0.34 | 0.16 | -0.08 |
| TCGA.56.7823.01 | -0.003 | -0.17 | -0.16 | -0.05 | 0.18 | -0.06 | -0.05 | 0.14 | 0.20 | 0.05 | 0.03 | 0.13 | -0.16 | 0.17 | -0.14 | -0.17 | -0.27 | -0.15 | 0.18 | -0.02 | -0.12 | -0.02 | 0.08 | 0.13 | -0.02 | -0.20 | -0.07 | 0.10 | -0.04 |
| TCGA.56.8082.01 | 0.221 | 0.27 | -0.02 | 0.10 | 0.21 | -0.05 | -0.07 | 0.18 | 0.28 | 0.29 | 0.18 | 0.22 | -0.02 | 0.02 | -0.10 | -0.06 | 0.05 | 0.24 | -0.03 | 0.07 | -0.20 | -0.03 | -0.13 | 0.10 | -0.10 | -0.23 | -0.05 | 0.01 | 0.10 |
| TCGA.56.8201.01 | -0.029 | 0.44 | -0.08 | 0.21 | 0.14 | 0.15 | 0.21 | 0.10 | 0.24 | 0.01 | 0.16 | 0.21 | -0.08 | 0.22 | 0.25 | 0.46 | 0.47 | 0.41 | 0.18 | 0.18 | 0.16 | 0.16 | 0.27 | 0.25 | 0.13 | 0.14 | 0.27 | -0.20 | -0.13 |
| TCGA.56.8307.01 | 0.162 | 0.19 | -0.18 | 0.20 | 0.08 | 0.16 | 0.14 | -0.04 | 0.03 | 0.13 | 0.18 | -0.01 | -0.18 | 0.05 | -0.16 | 0.27 | 0.23 | 0.24 | 0.12 | 0.02 | 0.14 | 0.07 | 0.18 | 0.20 | 0.00 | 0.17 | 0.17 | -0.04 | -0.10 |
| TCGA.56.8308.01 | 0.324 | -0.07 | 0.10 | -0.10 | -0.25 | -0.10 | -0.05 | 0.01 | 0.02 | -0.12 | 0.11 | 0.01 | 0.10 | -0.18 | 0.09 | -0.05 | -0.14 | -0.12 | 0.22 | 0.05 | 0.00 | -0.19 | 0.04 | 0.07 | 0.02 | 0.20 | 0.13 | 0.14 | 0.22 |
| TCGA.56.8309.01 | 0.031 | -0.44 | -0.44 | -0.30 | -0.35 | -0.28 | -0.36 | -0.12 | -0.23 | -0.17 | -0.06 | 0.02 | -0.44 | -0.29 | -0.20 | -0.36 | -0.24 | -0.34 | -0.34 | -0.18 | -0.41 | -0.27 | -0.33 | -0.27 | -0.39 | -0.34 | -0.29 | -0.21 | 0.19 |
| TCGA.56.8504.01 | -0.430 | -0.03 | -0.22 | 0.09 | 0.00 | 0.08 | 0.02 | 0.04 | 0.05 | 0.05 | 0.00 | 0.07 | -0.22 | 0.24 | 0.16 | 0.33 | 0.47 | 0.30 | 0.25 | 0.15 | 0.06 | 0.02 | 0.08 | 0.02 | 0.11 | 0.21 | 0.17 | -0.08 | -0.01 |
| TCGA.56.8623.01 | -0.257 | -0.08 | 0.21 | 0.24 | -0.02 | 0.27 | 0.27 | -0.08 | 0.07 | 0.29 | 0.12 | -0.07 | 0.21 | -0.09 | 0.26 | 0.32 | 0.33 | 0.32 | -0.19 | 0.05 | 0.35 | 0.22 | 0.28 | 0.09 | 0.32 | 0.34 | 0.03 | -0.10 | -0.21 |
| TCGA.56.8624.01 | 0.199 | -0.27 | -0.20 | -0.30 | -0.36 | -0.26 | -0.32 | -0.10 | -0.43 | -0.37 | -0.10 | -0.15 | -0.20 | -0.24 | -0.14 | -0.27 | -0.17 | -0.23 | -0.18 | -0.16 | -0.29 | -0.38 | -0.33 | -0.29 | -0.33 | -0.27 | -0.21 | -0.20 | 0.19 |
| TCGA.56.8625.01 | -0.025 | -0.12 | 0.39 | 0.00 | -0.03 | 0.10 | 0.02 | 0.04 | 0.03 | 0.19 | -0.32 | -0.14 | 0.39 | 0.15 | -0.16 | 0.26 | 0.38 | -0.07 | -0.33 | -0.28 | 0.11 | 0.02 | 0.15 | -0.03 | 0.05 | -0.02 | 0.02 | -0.24 | -0.19 |
| TCGA.56.8626.01 | -0.186 | 0.07 | 0.22 | 0.17 | 0.14 | 0.16 | 0.19 | 0.25 | 0.22 | 0.30 | 0.13 | 0.21 | 0.22 | 0.18 | 0.11 | 0.00 | -0.10 | -0.24 | 0.04 | 0.00 | 0.28 | -0.02 | 0.21 | -0.11 | 0.25 | 0.09 | 0.20 | 0.06 | -0.19 |
| TCGA.56.8628.01 | 0.079 | -0.19 | -0.14 | -0.24 | -0.05 | -0.28 | -0.21 | -0.19 | 0.00 | -0.24 | -0.06 | 0.04 | -0.14 | -0.16 | -0.12 | -0.43 | -0.42 | -0.32 | 0.16 | -0.04 | -0.33 | 0.04 | -0.30 | -0.11 | -0.22 | -0.21 | -0.34 | 0.10 | 0.40 |
| TCGA.56.8629.01 | 0.481 | 0.38 | 0.15 | 0.00 | 0.09 | -0.18 | -0.09 | 0.07 | 0.28 | -0.20 | -0.07 | 0.01 | 0.15 | 0.08 | -0.24 | -0.12 | -0.26 | -0.05 | -0.04 | 0.08 | -0.26 | -0.17 | -0.11 | -0.12 | -0.20 | -0.34 | -0.14 | -0.04 | 0.00 |
| TCGA.56.A49D.01 | -0.384 | -0.36 | 0.27 | 0.07 | -0.20 | 0.18 | 0.07 | -0.17 | -0.21 | 0.15 | -0.12 | -0.31 | 0.27 | -0.03 | 0.11 | 0.28 | 0.38 | 0.05 | -0.18 | -0.06 | 0.21 | 0.23 | 0.25 | 0.07 | 0.19 | 0.21 | -0.11 | -0.14 | -0.19 |
| TCGA.56.A4BW.01 | -0.062 | -0.31 | -0.37 | -0.19 | -0.15 | -0.25 | -0.32 | 0.02 | -0.11 | 0.10 | -0.13 | 0.05 | -0.37 | -0.18 | -0.19 | -0.39 | -0.30 | -0.41 | -0.13 | 0.00 | -0.27 | 0.08 | -0.19 | -0.20 | -0.09 | -0.30 | -0.20 | 0.00 | 0.20 |
| TCGA.56.A4BX.01 | -0.484 | -0.25 | -0.04 | 0.17 | -0.22 | 0.14 | 0.14 | -0.20 | -0.25 | -0.23 | 0.14 | -0.08 | -0.04 | 0.02 | 0.20 | 0.25 | 0.22 | 0.34 | -0.05 | 0.01 | 0.23 | 0.01 | 0.04 | 0.02 | 0.19 | 0.25 | -0.02 | -0.10 | -0.05 |
| TCGA.56.A4BY.01 | 0.557 | -0.24 | -0.17 | 0.01 | -0.19 | -0.06 | -0.13 | 0.05 | 0.07 | 0.07 | -0.11 | -0.14 | -0.17 | -0.20 | -0.17 | -0.23 | -0.26 | -0.31 | -0.31 | -0.17 | -0.06 | -0.10 | -0.09 | -0.02 | -0.07 | -0.02 | -0.14 | 0.00 | 0.07 |
| TCGA.56.A4ZJ.01 | 0.384 | 0.28 | -0.23 | -0.05 | 0.18 | -0.17 | -0.04 | 0.14 | 0.18 | 0.06 | 0.25 | 0.26 | -0.23 | 0.16 | 0.02 | -0.23 | -0.16 | 0.01 | 0.31 | 0.09 | -0.18 | 0.01 | -0.01 | 0.26 | -0.18 | -0.08 | 0.00 | 0.32 | 0.31 |
| TCGA.56.A4ZK.01 | -0.161 | -0.52 | -0.14 | -0.27 | -0.36 | -0.24 | -0.33 | -0.31 | -0.41 | -0.48 | -0.16 | -0.27 | -0.14 | -0.22 | -0.19 | -0.04 | 0.16 | -0.06 | -0.01 | -0.19 | -0.26 | -0.36 | -0.26 | -0.25 | -0.23 | -0.28 | -0.23 | -0.23 | 0.09 |
| TCGA.56.A5DR.01 | 0.192 | 0.20 | 0.10 | 0.01 | 0.29 | -0.10 | 0.04 | 0.14 | 0.17 | -0.06 | 0.08 | 0.16 | 0.10 | 0.24 | -0.06 | -0.15 | 0.13 | 0.20 | 0.18 | 0.04 | -0.18 | 0.16 | -0.01 | 0.09 | -0.07 | -0.14 | 0.00 | 0.19 | 0.17 |
| TCGA.56.A62T.01 | -0.373 | -0.08 | 0.13 | 0.10 | -0.23 | 0.07 | -0.09 | -0.15 | -0.41 | -0.35 | -0.15 | -0.27 | 0.13 | -0.18 | 0.03 | 0.06 | 0.20 | 0.02 | -0.10 | -0.12 | 0.14 | -0.38 | -0.21 | -0.17 | -0.02 | -0.05 | 0.08 | -0.27 | -0.24 |
| TCGA.58.8386.01 | 0.177 | 0.58 | 0.04 | 0.37 | 0.31 | 0.34 | 0.40 | 0.24 | 0.38 | 0.41 | 0.28 | 0.29 | 0.04 | 0.27 | 0.35 | 0.44 | 0.22 | 0.34 | 0.19 | 0.29 | 0.38 | 0.38 | 0.40 | 0.36 | 0.37 | 0.44 | 0.23 | 0.29 | 0.02 |
| TCGA.58.8387.01 | -0.059 | -0.15 | -0.30 | -0.02 | -0.16 | -0.02 | -0.10 | -0.25 | -0.36 | -0.44 | -0.03 | -0.21 | -0.30 | 0.05 | 0.07 | 0.09 | 0.12 | 0.14 | 0.07 | -0.07 | -0.03 | -0.26 | -0.22 | 0.09 | -0.10 | -0.05 | 0.05 | -0.18 | 0.04 |
| TCGA.58.8388.01 | -0.094 | 0.28 | 0.06 | 0.25 | -0.01 | 0.16 | 0.08 | -0.03 | 0.16 | 0.00 | 0.08 | 0.15 | 0.06 | 0.14 | 0.11 | -0.02 | -0.11 | -0.02 | -0.07 | 0.17 | 0.06 | 0.14 | 0.06 | 0.35 | 0.06 | 0.13 | 0.05 | 0.13 | 0.01 |
| TCGA.58.8390.01 | 0.158 | 0.44 | 0.00 | 0.08 | 0.12 | 0.05 | 0.00 | 0.37 | 0.34 | 0.54 | 0.29 | 0.30 | 0.00 | -0.02 | 0.04 | 0.02 | 0.00 | 0.17 | -0.08 | 0.05 | -0.06 | -0.07 | 0.08 | -0.17 | 0.01 | -0.13 | 0.17 | 0.05 | 0.18 |
| TCGA.58.8391.01 | 0.008 | 0.22 | -0.09 | 0.04 | 0.04 | 0.10 | 0.21 | 0.33 | 0.13 | 0.30 | 0.29 | 0.27 | -0.09 | 0.02 | 0.23 | 0.16 | 0.20 | -0.01 | -0.09 | 0.13 | 0.21 | 0.02 | 0.14 | -0.09 | 0.14 | 0.21 | 0.15 | -0.28 | -0.35 |
| TCGA.58.8392.01 | 0.295 | 0.04 | -0.18 | 0.08 | -0.13 | -0.08 | -0.09 | -0.33 | -0.26 | -0.35 | -0.15 | -0.26 | -0.18 | -0.10 | -0.07 | 0.37 | 0.42 | 0.42 | 0.02 | -0.03 | -0.26 | -0.12 | -0.18 | 0.00 | -0.10 | -0.20 | 0.03 | -0.34 | -0.12 |
| TCGA.58.8393.01 | -0.156 | 0.07 | -0.36 | -0.14 | -0.31 | -0.05 | 0.03 | 0.01 | -0.11 | 0.05 | 0.16 | -0.04 | -0.36 | -0.34 | 0.14 | -0.24 | -0.13 | -0.14 | -0.01 | -0.08 | 0.08 | -0.25 | -0.04 | -0.31 | -0.01 | 0.09 | -0.01 | -0.06 | -0.17 |
| TCGA.58.A46J.01 | 0.198 | -0.23 | -0.14 | -0.19 | -0.03 | -0.27 | -0.19 | 0.04 | 0.01 | -0.16 | 0.05 | -0.03 | -0.14 | -0.19 | -0.29 | -0.35 | -0.33 | -0.16 | 0.02 | -0.20 | -0.25 | -0.30 | -0.18 | -0.27 | -0.19 | -0.36 | -0.17 | -0.10 | 0.12 |
| TCGA.58.A46K.01 | 0.156 | -0.19 | -0.11 | 0.07 | 0.02 | 0.01 | -0.06 | -0.04 | -0.07 | -0.18 | 0.17 | 0.01 | -0.11 | -0.03 | 0.19 | 0.12 | 0.23 | 0.31 | -0.13 | 0.06 | -0.22 | -0.07 | -0.17 | 0.10 | -0.06 | -0.06 | -0.04 | -0.02 | -0.02 |
| TCGA.58.A46L.01 | -0.457 | -0.14 | -0.30 | -0.21 | -0.04 | -0.18 | -0.32 | -0.17 | 0.01 | -0.21 | -0.29 | -0.19 | -0.30 | -0.26 | -0.21 | -0.18 | -0.16 | -0.30 | -0.30 | -0.19 | -0.22 | 0.03 | -0.15 | -0.23 | -0.30 | -0.26 | -0.27 | 0.02 | -0.20 |
| TCGA.58.A46M.01 | -0.216 | -0.27 | 0.08 | -0.28 | -0.20 | -0.33 | -0.29 | -0.17 | -0.13 | -0.12 | -0.32 | -0.09 | 0.08 | -0.38 | -0.21 | -0.22 | -0.16 | -0.11 | -0.25 | -0.23 | -0.33 | 0.05 | -0.27 | -0.16 | -0.33 | -0.26 | -0.29 | -0.11 | -0.07 |
| TCGA.58.A46N.01 | 0.012 | -0.09 | 0.15 | 0.00 | 0.22 | -0.10 | -0.09 | 0.06 | 0.03 | 0.16 | -0.15 | 0.15 | 0.15 | 0.19 | -0.09 | -0.15 | -0.25 | -0.02 | 0.17 | -0.07 | -0.14 | 0.11 | 0.06 | 0.05 | -0.18 | -0.18 | -0.03 | 0.22 | 0.19 |
| TCGA.60.2695.01 | -0.335 | -0.29 | -0.31 | -0.28 | -0.14 | -0.27 | -0.34 | -0.17 | -0.32 | -0.30 | -0.22 | -0.21 | -0.31 | -0.18 | -0.35 | -0.39 | -0.21 | -0.23 | -0.13 | -0.19 | -0.35 | -0.23 | -0.23 | -0.34 | -0.31 | -0.41 | -0.30 | -0.21 | 0.02 |
| TCGA.60.2696.01 | -0.119 | 0.31 | -0.25 | 0.07 | -0.02 | 0.08 | -0.07 | -0.14 | -0.26 | -0.40 | -0.08 | -0.17 | -0.25 | 0.34 | 0.01 | 0.06 | 0.15 | 0.26 | 0.15 | 0.25 | -0.22 | -0.08 | -0.11 | 0.16 | -0.14 | -0.08 | 0.08 | 0.13 | 0.28 |
| TCGA.60.2697.01 | -0.146 | 0.13 | 0.39 | 0.29 | 0.03 | 0.34 | 0.26 | -0.01 | -0.03 | 0.20 | 0.15 | -0.10 | 0.39 | 0.15 | 0.23 | 0.47 | 0.50 | 0.42 | 0.17 | 0.18 | 0.24 | 0.18 | 0.27 | 0.04 | 0.25 | 0.28 | 0.28 | -0.10 | -0.31 |
| TCGA.60.2698.01 | 0.364 | -0.06 | -0.03 | 0.16 | 0.08 | 0.04 | 0.04 | 0.09 | 0.08 | -0.14 | -0.02 | -0.08 | -0.03 | 0.04 | 0.12 | 0.28 | 0.24 | 0.23 | 0.15 | 0.13 | -0.07 | -0.03 | 0.02 | 0.13 | 0.12 | 0.04 | 0.08 | 0.21 | 0.10 |
| TCGA.60.2703.01 | 0.304 | 0.40 | -0.23 | 0.01 | 0.29 | -0.03 | 0.07 | 0.18 | 0.19 | 0.20 | 0.29 | 0.27 | -0.23 | 0.37 | 0.02 | -0.03 | 0.02 | 0.15 | 0.33 | 0.26 | -0.20 | 0.17 | 0.01 | 0.18 | -0.11 | -0.07 | 0.07 | 0.23 | 0.31 |
| TCGA.60.2704.01 | 0.152 | 0.45 | 0.15 | 0.25 | 0.13 | 0.26 | 0.25 | 0.21 | 0.07 | 0.43 | 0.39 | 0.20 | 0.15 | 0.05 | 0.28 | 0.41 | 0.26 | 0.21 | 0.13 | 0.28 | 0.30 | 0.33 | 0.31 | 0.08 | 0.26 | 0.33 | 0.31 | 0.05 | -0.21 |
| TCGA.60.2706.01 | 0.435 | 0.07 | 0.03 | -0.17 | 0.03 | -0.19 | -0.04 | 0.08 | 0.23 | -0.11 | -0.09 | -0.02 | 0.03 | 0.04 | -0.05 | -0.20 | -0.24 | -0.09 | 0.15 | 0.11 | -0.18 | 0.12 | -0.13 | 0.22 | -0.14 | 0.01 | -0.11 | 0.26 | 0.30 |
| TCGA.60.2707.01 | -0.395 | 0.05 | -0.08 | -0.21 | -0.13 | -0.06 | 0.01 | -0.06 | -0.16 | 0.03 | 0.15 | -0.09 | -0.08 | -0.16 | 0.03 | -0.19 | -0.18 | -0.29 | -0.09 | -0.17 | 0.05 | 0.02 | 0.00 | -0.33 | -0.08 | -0.13 | -0.10 | -0.23 | -0.15 |
| TCGA.60.2708.01 | 0.540 | 0.27 | 0.06 | 0.12 | 0.20 | 0.07 | 0.18 | 0.03 | 0.24 | 0.10 | 0.04 | 0.05 | 0.06 | 0.33 | 0.21 | 0.31 | 0.31 | 0.30 | 0.27 | 0.11 | 0.03 | 0.25 | 0.35 | 0.31 | -0.04 | 0.07 | 0.12 | 0.13 | 0.27 |
| TCGA.60.2709.01 | 0.590 | 0.06 | -0.19 | 0.08 | 0.13 | 0.12 | 0.21 | 0.01 | 0.01 | -0.06 | 0.18 | 0.15 | -0.19 | 0.12 | 0.24 | -0.02 | 0.07 | 0.10 | 0.08 | 0.14 | 0.01 | 0.32 | 0.20 | 0.30 | 0.14 | 0.22 | 0.11 | 0.23 | 0.18 |
| TCGA.60.2710.01 | -0.326 | -0.02 | -0.26 | 0.07 | 0.15 | 0.08 | 0.02 | 0.10 | 0.18 | 0.10 | 0.33 | 0.26 | -0.26 | 0.08 | 0.05 | 0.02 | 0.15 | 0.04 | -0.14 | -0.08 | -0.03 | -0.10 | 0.20 | -0.06 | 0.02 | 0.02 | 0.12 | -0.19 | -0.30 |
| TCGA.60.2711.01 | 0.485 | -0.21 | 0.16 | -0.04 | -0.27 | 0.01 | -0.02 | -0.19 | -0.30 | -0.33 | -0.12 | -0.23 | 0.16 | -0.03 | -0.02 | 0.19 | 0.32 | 0.26 | -0.08 | -0.08 | 0.00 | -0.14 | -0.13 | 0.04 | 0.09 | 0.13 | 0.05 | -0.09 | 0.01 |
| TCGA.60.2712.01 | -0.137 | 0.20 | 0.34 | 0.15 | 0.01 | 0.20 | 0.12 | 0.14 | 0.21 | 0.30 | 0.09 | -0.03 | 0.34 | -0.12 | 0.17 | 0.20 | 0.16 | -0.02 | -0.10 | -0.03 | 0.35 | 0.11 | 0.13 | 0.01 | 0.23 | 0.25 | 0.10 | 0.06 | -0.16 |
| TCGA.60.2713.01 | 0.211 | -0.24 | 0.22 | -0.22 | -0.12 | -0.26 | -0.26 | -0.23 | -0.02 | -0.30 | -0.28 | -0.21 | 0.22 | -0.26 | -0.24 | -0.45 | -0.37 | -0.31 | -0.15 | -0.18 | -0.23 | -0.08 | -0.27 | -0.32 | -0.27 | -0.28 | -0.26 | -0.10 | 0.23 |
| TCGA.60.2714.01 | 0.213 | -0.52 | 0.12 | -0.21 | -0.22 | -0.13 | -0.24 | -0.37 | -0.22 | -0.19 | -0.34 | -0.42 | 0.12 | -0.27 | -0.23 | 0.06 | 0.17 | -0.13 | -0.16 | -0.26 | 0.00 | -0.12 | -0.09 | -0.14 | -0.03 | -0.21 | -0.21 | -0.15 | -0.21 |
| TCGA.60.2715.01 | -0.234 | -0.03 | 0.24 | 0.01 | -0.25 | 0.16 | 0.11 | 0.02 | -0.05 | -0.02 | 0.26 | -0.03 | 0.24 | -0.29 | 0.04 | 0.21 | 0.28 | 0.07 | 0.09 | -0.04 | 0.30 | -0.02 | 0.16 | -0.09 | 0.22 | 0.27 | 0.22 | 0.00 | -0.20 |
| TCGA.60.2716.01 | 0.108 | -0.03 | -0.06 | -0.12 | 0.15 | -0.08 | -0.16 | 0.00 | 0.24 | -0.17 | -0.12 | 0.00 | -0.06 | -0.07 | -0.09 | -0.27 | -0.36 | -0.26 | 0.23 | 0.06 | -0.22 | 0.11 | -0.14 | 0.03 | -0.20 | -0.17 | -0.15 | 0.13 | 0.24 |
| TCGA.60.2719.01 | 0.115 | 0.19 | 0.16 | 0.06 | 0.22 | 0.13 | -0.01 | 0.12 | 0.16 | -0.15 | 0.16 | 0.05 | 0.16 | 0.18 | 0.07 | -0.03 | -0.08 | -0.05 | 0.21 | 0.14 | 0.02 | -0.01 | 0.14 | -0.08 | -0.06 | -0.05 | 0.17 | 0.01 | 0.14 |
| TCGA.60.2720.01 | -0.413 | -0.20 | -0.13 | -0.03 | -0.07 | -0.02 | -0.08 | 0.04 | -0.15 | -0.19 | -0.03 | 0.11 | -0.13 | 0.08 | -0.01 | -0.18 | 0.08 | -0.04 | 0.17 | -0.02 | -0.05 | -0.17 | -0.07 | 0.03 | -0.02 | -0.19 | 0.03 | 0.16 | -0.01 |
| TCGA.60.2721.01 | -0.238 | 0.47 | -0.08 | 0.26 | 0.03 | 0.28 | 0.30 | 0.13 | 0.27 | 0.24 | 0.19 | 0.23 | -0.08 | 0.12 | 0.14 | 0.15 | 0.16 | -0.05 | 0.15 | 0.17 | 0.20 | -0.06 | 0.21 | 0.24 | 0.17 | 0.13 | 0.30 | 0.13 | 0.07 |
| TCGA.60.2722.01 | -0.348 | -0.05 | 0.35 | 0.06 | -0.13 | 0.02 | 0.04 | -0.18 | -0.24 | -0.17 | -0.24 | -0.21 | 0.35 | 0.08 | -0.23 | 0.34 | 0.35 | 0.30 | 0.03 | -0.12 | 0.15 | 0.04 | 0.01 | -0.13 | 0.02 | -0.04 | 0.13 | -0.31 | -0.35 |
| TCGA.60.2723.01 | 0.115 | -0.16 | 0.30 | -0.01 | -0.21 | -0.06 | -0.06 | -0.19 | -0.09 | -0.12 | -0.31 | -0.29 | 0.30 | 0.01 | -0.22 | -0.04 | 0.08 | 0.10 | -0.18 | -0.28 | 0.01 | -0.18 | -0.04 | -0.13 | -0.12 | -0.14 | -0.13 | -0.16 | 0.13 |
| TCGA.60.2724.01 | 0.074 | 0.05 | 0.32 | 0.21 | 0.27 | 0.22 | 0.06 | -0.07 | 0.09 | 0.14 | -0.03 | 0.03 | 0.32 | 0.18 | 0.24 | 0.34 | 0.44 | 0.43 | 0.09 | 0.13 | -0.03 | 0.38 | 0.20 | 0.29 | -0.14 | 0.03 | 0.12 | 0.24 | 0.24 |
| TCGA.60.2725.01 | -0.090 | -0.35 | -0.20 | -0.16 | -0.18 | -0.19 | -0.26 | -0.19 | -0.33 | -0.47 | -0.07 | -0.14 | -0.20 | 0.00 | -0.14 | -0.17 | -0.21 | -0.18 | 0.12 | -0.10 | -0.21 | -0.41 | -0.30 | -0.10 | -0.21 | -0.11 | -0.09 | -0.12 | 0.16 |
| TCGA.60.2726.01 | -0.160 | 0.36 | -0.06 | -0.07 | 0.05 | 0.04 | 0.02 | -0.19 | 0.04 | 0.10 | -0.31 | -0.14 | -0.06 | 0.19 | -0.17 | 0.18 | 0.27 | 0.07 | 0.25 | -0.10 | 0.00 | 0.15 | 0.07 | 0.05 | -0.03 | -0.15 | -0.02 | -0.05 | 0.11 |
| TCGA.62.8394.01 | 0.505 | -0.32 | 0.04 | -0.09 | -0.13 | -0.13 | -0.10 | -0.31 | -0.07 | -0.32 | -0.22 | -0.36 | 0.04 | -0.17 | -0.19 | -0.03 | -0.21 | 0.09 | -0.13 | -0.25 | -0.21 | -0.34 | -0.17 | -0.22 | -0.09 | -0.22 | -0.09 | -0.18 | 0.14 |
| TCGA.62.8395.01 | -0.047 | -0.07 | 0.17 | 0.16 | 0.13 | 0.13 | 0.16 | 0.12 | 0.15 | 0.32 | 0.03 | 0.11 | 0.17 | -0.02 | 0.20 | -0.01 | -0.17 | -0.21 | 0.07 | 0.07 | 0.30 | -0.01 | 0.16 | -0.04 | 0.19 | 0.25 | 0.05 | 0.06 | 0.23 |
| TCGA.62.8397.01 | -0.403 | -0.36 | 0.29 | 0.24 | -0.10 | 0.21 | 0.13 | -0.04 | -0.28 | -0.01 | 0.04 | -0.07 | 0.29 | -0.06 | 0.21 | 0.37 | 0.41 | 0.31 | -0.15 | -0.02 | 0.35 | 0.00 | 0.21 | -0.02 | 0.33 | 0.33 | 0.05 | -0.21 | -0.28 |
| TCGA.62.8398.01 | 0.213 | 0.09 | 0.13 | -0.06 | 0.02 | -0.03 | 0.03 | -0.07 | 0.16 | -0.06 | 0.11 | -0.10 | 0.13 | -0.01 | 0.14 | -0.11 | 0.20 | 0.08 | 0.21 | 0.01 | 0.10 | 0.13 | -0.01 | -0.09 | -0.04 | 0.13 | 0.10 | 0.07 | 0.05 |
| TCGA.62.8399.01 | -0.034 | 0.47 | -0.06 | 0.04 | 0.03 | 0.07 | 0.18 | -0.03 | 0.26 | 0.05 | 0.08 | 0.07 | -0.06 | 0.08 | 0.07 | 0.13 | -0.12 | 0.14 | 0.03 | 0.01 | 0.02 | 0.15 | 0.17 | -0.18 | -0.10 | -0.05 | 0.04 | 0.05 | 0.07 |
| TCGA.62.8402.01 | -0.050 | 0.22 | 0.05 | 0.02 | 0.05 | -0.11 | -0.06 | -0.31 | -0.18 | -0.25 | -0.30 | -0.21 | 0.05 | 0.07 | -0.12 | -0.11 | 0.12 | 0.14 | 0.01 | -0.05 | 0.04 | -0.09 | -0.13 | -0.11 | -0.14 | -0.14 | 0.00 | -0.11 | 0.00 |
| TCGA.62.A46O.01 | -0.260 | 0.14 | 0.05 | -0.29 | -0.10 | -0.31 | -0.31 | -0.19 | -0.14 | -0.04 | 0.03 | -0.13 | 0.05 | -0.31 | -0.19 | -0.34 | -0.40 | -0.34 | -0.16 | -0.10 | -0.35 | 0.00 | -0.36 | -0.20 | -0.22 | -0.26 | -0.25 | 0.06 | 0.23 |
| TCGA.62.A46P.01 | 0.250 | -0.55 | -0.02 | -0.09 | -0.33 | -0.06 | -0.12 | -0.15 | 0.03 | -0.27 | -0.15 | -0.15 | -0.02 | -0.24 | -0.20 | -0.22 | -0.19 | -0.16 | -0.19 | -0.27 | 0.06 | -0.30 | -0.11 | -0.26 | 0.05 | -0.12 | -0.16 | -0.19 | 0.10 |
| TCGA.62.A46R.01 | -0.083 | -0.18 | -0.13 | -0.13 | -0.12 | -0.28 | -0.22 | -0.21 | -0.17 | -0.37 | -0.17 | -0.12 | -0.13 | 0.03 | -0.28 | -0.07 | 0.00 | 0.04 | -0.02 | -0.17 | -0.09 | -0.29 | -0.24 | 0.03 | -0.18 | -0.24 | -0.12 | -0.31 | -0.06 |
| TCGA.62.A46S.01 | 0.229 | 0.01 | 0.44 | 0.25 | -0.03 | 0.33 | 0.36 | 0.12 | -0.01 | 0.22 | 0.16 | 0.03 | 0.44 | -0.03 | 0.24 | 0.33 | 0.13 | 0.07 | 0.10 | 0.16 | 0.36 | 0.10 | 0.24 | 0.22 | 0.38 | 0.32 | 0.23 | 0.11 | -0.21 |
| TCGA.62.A46V.01 | -0.357 | -0.55 | -0.12 | -0.35 | -0.30 | -0.37 | -0.36 | -0.13 | -0.34 | -0.34 | -0.40 | -0.16 | -0.12 | -0.35 | -0.31 | -0.41 | -0.47 | -0.46 | -0.42 | -0.37 | -0.20 | -0.32 | -0.39 | -0.32 | -0.29 | -0.25 | -0.40 | -0.33 | -0.04 |
| TCGA.62.A46Y.01 | -0.264 | -0.52 | -0.42 | -0.26 | -0.18 | -0.24 | -0.27 | -0.13 | -0.03 | -0.48 | -0.15 | -0.22 | -0.42 | -0.18 | -0.19 | -0.32 | -0.16 | -0.37 | -0.36 | -0.12 | -0.24 | -0.25 | -0.21 | -0.19 | -0.20 | -0.24 | -0.28 | -0.25 | 0.02 |
| TCGA.62.A471.01 | -0.434 | 0.28 | 0.28 | 0.36 | 0.18 | 0.39 | 0.39 | 0.24 | -0.02 | 0.34 | 0.24 | 0.29 | 0.28 | 0.24 | 0.33 | 0.42 | 0.27 | 0.35 | 0.13 | 0.15 | 0.46 | 0.21 | 0.36 | 0.27 | 0.44 | 0.42 | 0.29 | 0.16 | -0.27 |
| TCGA.62.A472.01 | 0.132 | 0.12 | -0.20 | 0.02 | 0.20 | -0.06 | -0.08 | 0.05 | -0.05 | 0.10 | -0.02 | 0.19 | -0.20 | 0.19 | -0.06 | -0.21 | -0.16 | 0.00 | 0.15 | 0.08 | -0.25 | 0.12 | -0.01 | 0.22 | -0.11 | -0.25 | -0.05 | 0.21 | 0.32 |
| TCGA.63.5128.01 | -0.425 | 0.36 | -0.32 | 0.16 | 0.41 | 0.12 | 0.18 | 0.21 | -0.06 | 0.15 | 0.29 | 0.32 | -0.32 | 0.35 | 0.12 | -0.18 | -0.17 | -0.02 | 0.43 | 0.16 | -0.02 | 0.06 | 0.15 | 0.11 | 0.19 | 0.20 | 0.11 | 0.20 | 0.23 |
| TCGA.63.5131.01 | 0.521 | 0.35 | 0.19 | -0.05 | 0.22 | -0.09 | -0.15 | 0.04 | -0.01 | -0.01 | -0.01 | -0.19 | 0.19 | 0.06 | -0.07 | -0.15 | -0.13 | 0.04 | 0.39 | 0.25 | -0.27 | -0.15 | -0.10 | 0.08 | -0.07 | -0.10 | -0.07 | 0.14 | 0.19 |
| TCGA.63.6202.01 | 0.482 | 0.19 | -0.19 | 0.13 | 0.18 | 0.06 | 0.20 | -0.11 | 0.20 | -0.22 | -0.06 | -0.10 | -0.19 | 0.38 | 0.25 | 0.20 | 0.31 | 0.20 | 0.30 | 0.15 | 0.11 | 0.21 | 0.15 | 0.20 | 0.01 | 0.20 | 0.24 | 0.13 | 0.30 |
| TCGA.63.7020.01 | 0.077 | 0.35 | 0.44 | 0.12 | 0.00 | 0.21 | 0.14 | 0.03 | -0.16 | -0.03 | 0.13 | 0.00 | 0.44 | -0.14 | 0.21 | 0.25 | 0.43 | 0.21 | 0.00 | 0.13 | 0.14 | 0.10 | 0.11 | 0.08 | 0.12 | 0.26 | 0.30 | 0.09 | -0.32 |
| TCGA.63.7021.01 | 0.273 | 0.41 | 0.20 | 0.12 | -0.02 | 0.25 | 0.24 | 0.27 | 0.29 | 0.25 | 0.23 | 0.19 | 0.20 | 0.01 | 0.23 | 0.20 | 0.28 | 0.08 | 0.06 | 0.14 | 0.33 | 0.14 | 0.30 | 0.09 | 0.25 | 0.35 | 0.33 | 0.14 | -0.19 |
| TCGA.63.7022.01 | 0.080 | 0.29 | -0.02 | -0.07 | 0.16 | -0.25 | -0.14 | -0.13 | 0.10 | -0.20 | -0.24 | -0.10 | -0.02 | -0.15 | -0.30 | -0.10 | -0.21 | -0.11 | -0.09 | 0.00 | -0.11 | 0.04 | -0.19 | -0.04 | -0.14 | -0.12 | -0.13 | 0.04 | 0.07 |
| TCGA.63.7023.01 | -0.438 | -0.20 | -0.21 | 0.06 | 0.01 | 0.06 | 0.02 | 0.01 | 0.01 | -0.11 | -0.13 | -0.02 | -0.21 | 0.15 | -0.08 | 0.21 | 0.19 | 0.01 | 0.11 | -0.15 | 0.18 | -0.23 | -0.02 | 0.02 | -0.01 | -0.10 | 0.12 | -0.04 | 0.00 |
| TCGA.63.A5MB.01 | -0.226 | -0.37 | 0.04 | -0.21 | 0.01 | -0.17 | -0.26 | -0.19 | -0.14 | -0.07 | -0.26 | -0.21 | 0.04 | -0.21 | -0.08 | -0.29 | -0.34 | -0.31 | -0.21 | -0.06 | -0.26 | -0.01 | -0.11 | -0.24 | -0.29 | -0.19 | -0.25 | -0.04 | 0.05 |
| TCGA.63.A5MG.01 | -0.421 | -0.01 | 0.03 | 0.10 | 0.07 | 0.13 | 0.16 | 0.09 | 0.27 | 0.35 | 0.15 | 0.11 | 0.03 | -0.21 | 0.26 | 0.10 | -0.20 | -0.03 | -0.27 | 0.11 | 0.28 | 0.28 | 0.13 | -0.01 | 0.25 | 0.29 | -0.06 | 0.03 | -0.23 |
| TCGA.63.A5MH.01 | -0.200 | -0.66 | 0.04 | -0.30 | -0.43 | -0.29 | -0.31 | -0.28 | -0.39 | -0.40 | -0.25 | -0.27 | 0.04 | -0.33 | -0.36 | -0.21 | -0.09 | -0.10 | -0.28 | -0.31 | -0.20 | -0.47 | -0.42 | -0.28 | -0.22 | -0.33 | -0.23 | -0.33 | -0.16 |
| TCGA.63.A5MI.01 | -0.130 | -0.49 | -0.03 | -0.06 | -0.27 | -0.03 | -0.10 | -0.04 | -0.48 | -0.24 | -0.03 | -0.01 | -0.03 | -0.33 | 0.07 | -0.11 | -0.12 | -0.23 | -0.34 | -0.16 | 0.08 | -0.28 | -0.21 | -0.08 | 0.03 | 0.06 | -0.11 | -0.25 | -0.17 |
| TCGA.63.A5MJ.01 | -0.329 | -0.36 | -0.08 | -0.06 | -0.19 | -0.12 | -0.19 | -0.21 | -0.39 | -0.51 | -0.09 | -0.21 | -0.08 | -0.09 | 0.01 | 0.01 | 0.07 | 0.26 | -0.06 | -0.18 | -0.22 | -0.27 | -0.20 | -0.11 | -0.08 | -0.13 | -0.11 | -0.31 | 0.05 |
| TCGA.63.A5ML.01 | 0.051 | 0.05 | -0.08 | -0.18 | -0.14 | -0.15 | -0.17 | -0.23 | -0.03 | -0.10 | -0.07 | 0.04 | -0.08 | -0.26 | -0.13 | -0.16 | 0.02 | 0.05 | -0.08 | 0.01 | -0.34 | -0.07 | -0.20 | 0.02 | -0.15 | -0.24 | -0.17 | 0.09 | 0.02 |
| TCGA.63.A5MM.01 | 0.131 | 0.25 | -0.55 | -0.02 | 0.20 | -0.09 | -0.11 | 0.32 | 0.04 | 0.24 | 0.24 | 0.35 | -0.55 | 0.15 | 0.05 | -0.21 | -0.03 | -0.07 | 0.14 | 0.15 | -0.16 | 0.06 | -0.15 | 0.15 | -0.04 | 0.01 | 0.01 | 0.14 | 0.25 |
| TCGA.63.A5MN.01 | -0.278 | -0.32 | 0.42 | 0.18 | -0.14 | 0.23 | 0.09 | -0.17 | 0.04 | 0.09 | -0.16 | -0.24 | 0.42 | 0.10 | 0.11 | 0.26 | 0.05 | 0.05 | -0.21 | -0.12 | 0.26 | 0.06 | 0.24 | -0.08 | 0.16 | 0.19 | 0.06 | -0.16 | -0.21 |
| TCGA.63.A5MP.01 | -0.575 | -0.20 | -0.02 | 0.09 | -0.03 | 0.01 | -0.05 | 0.03 | 0.04 | 0.00 | -0.01 | 0.01 | -0.02 | 0.01 | 0.14 | 0.17 | 0.13 | 0.07 | -0.25 | -0.03 | 0.15 | -0.14 | 0.06 | -0.17 | 0.12 | 0.01 | 0.03 | -0.12 | -0.28 |
| TCGA.63.A5MR.01 | -0.448 | -0.31 | -0.36 | 0.08 | 0.18 | -0.09 | -0.12 | 0.01 | -0.06 | -0.14 | 0.05 | 0.12 | -0.36 | 0.14 | -0.14 | 0.14 | 0.11 | 0.31 | 0.15 | -0.08 | -0.11 | -0.17 | -0.01 | -0.12 | -0.03 | -0.26 | -0.06 | -0.06 | -0.18 |
| TCGA.63.A5MS.01 | -0.205 | -0.24 | 0.52 | 0.13 | -0.21 | 0.21 | 0.15 | -0.03 | -0.10 | 0.27 | -0.10 | -0.23 | 0.52 | -0.04 | 0.09 | 0.36 | 0.45 | 0.19 | -0.13 | -0.10 | 0.43 | 0.04 | 0.28 | -0.03 | 0.28 | 0.25 | 0.08 | -0.24 | -0.25 |
| TCGA.63.A5MT.01 | -0.538 | -0.21 | 0.41 | 0.18 | -0.13 | 0.29 | 0.21 | 0.11 | 0.04 | 0.11 | 0.08 | 0.03 | 0.41 | 0.09 | 0.29 | 0.36 | 0.37 | 0.15 | -0.04 | 0.11 | 0.42 | 0.10 | 0.28 | -0.02 | 0.30 | 0.32 | 0.23 | -0.06 | -0.43 |
| TCGA.63.A5MU.01 | -0.483 | -0.11 | 0.17 | -0.02 | 0.17 | -0.03 | -0.09 | 0.20 | 0.19 | 0.23 | -0.06 | 0.14 | 0.17 | 0.16 | -0.05 | -0.09 | -0.22 | -0.07 | 0.07 | 0.03 | -0.06 | 0.17 | 0.07 | 0.17 | 0.10 | -0.05 | -0.02 | 0.13 | 0.03 |
| TCGA.63.A5MV.01 | -0.327 | -0.22 | -0.15 | -0.07 | -0.21 | -0.11 | -0.15 | -0.32 | -0.27 | -0.44 | -0.25 | -0.24 | -0.15 | -0.06 | -0.20 | 0.15 | 0.27 | 0.26 | 0.23 | -0.09 | -0.13 | -0.18 | -0.24 | -0.21 | -0.24 | -0.31 | -0.06 | -0.24 | 0.06 |
| TCGA.63.A5MW.01 | -0.346 | -0.17 | -0.42 | -0.10 | 0.11 | -0.19 | -0.19 | 0.03 | 0.35 | 0.11 | 0.07 | 0.10 | -0.42 | 0.01 | 0.01 | -0.33 | -0.21 | -0.25 | -0.23 | 0.03 | -0.34 | -0.01 | -0.22 | 0.01 | -0.09 | -0.25 | -0.28 | 0.12 | 0.04 |
| TCGA.63.A5MY.01 | -0.094 | 0.03 | -0.06 | -0.25 | 0.01 | -0.30 | -0.25 | -0.29 | -0.04 | -0.15 | -0.13 | -0.13 | -0.06 | -0.07 | -0.31 | -0.38 | -0.33 | -0.20 | 0.15 | -0.07 | -0.36 | -0.13 | -0.31 | -0.17 | -0.19 | -0.34 | -0.32 | 0.02 | 0.21 |
| TCGA.64.1676.01 | -0.529 | -0.24 | 0.23 | -0.06 | 0.05 | -0.04 | -0.01 | 0.13 | -0.03 | -0.13 | -0.12 | -0.02 | 0.23 | 0.09 | -0.02 | -0.09 | -0.07 | -0.16 | 0.23 | -0.11 | 0.01 | -0.15 | 0.08 | -0.05 | -0.03 | -0.11 | -0.03 | -0.08 | -0.12 |
| TCGA.64.1677.01 | -0.090 | -0.11 | 0.28 | 0.29 | 0.02 | 0.30 | 0.27 | 0.04 | -0.14 | 0.24 | 0.11 | -0.01 | 0.28 | 0.14 | 0.21 | 0.38 | 0.41 | 0.28 | -0.07 | 0.07 | 0.31 | 0.03 | 0.29 | 0.15 | 0.35 | 0.25 | 0.11 | -0.07 | -0.24 |
| TCGA.64.1678.01 | -0.336 | -0.60 | -0.25 | -0.30 | -0.45 | -0.26 | -0.32 | -0.25 | -0.41 | -0.41 | 0.04 | -0.17 | -0.25 | -0.37 | -0.17 | -0.42 | -0.44 | -0.41 | -0.04 | -0.21 | -0.22 | -0.43 | -0.42 | -0.36 | -0.27 | -0.26 | -0.28 | -0.17 | -0.01 |
| TCGA.64.1679.01 | -0.173 | -0.09 | 0.37 | 0.11 | 0.01 | 0.11 | 0.05 | 0.08 | 0.05 | -0.06 | -0.04 | 0.00 | 0.37 | -0.01 | 0.16 | 0.25 | 0.31 | 0.25 | 0.06 | 0.07 | 0.19 | 0.05 | 0.16 | -0.05 | 0.10 | 0.10 | 0.21 | -0.08 | -0.27 |
| TCGA.64.1680.01 | -0.010 | -0.53 | -0.13 | -0.24 | -0.23 | -0.27 | -0.34 | -0.26 | -0.41 | -0.30 | -0.29 | -0.31 | -0.13 | -0.31 | -0.35 | -0.20 | -0.30 | -0.29 | -0.30 | -0.27 | -0.12 | -0.27 | -0.34 | -0.28 | -0.25 | -0.29 | -0.23 | -0.30 | -0.10 |
| TCGA.64.1681.01 | 0.079 | 0.22 | -0.08 | 0.10 | -0.15 | 0.16 | 0.18 | -0.10 | -0.05 | 0.15 | -0.15 | -0.09 | -0.08 | -0.18 | 0.01 | 0.08 | -0.06 | 0.00 | -0.10 | -0.19 | 0.18 | -0.17 | -0.04 | -0.03 | 0.11 | 0.19 | -0.03 | -0.11 | -0.04 |
| TCGA.64.5774.01 | 0.140 | -0.42 | -0.02 | -0.20 | -0.20 | -0.18 | -0.30 | -0.12 | 0.09 | 0.01 | -0.22 | -0.35 | -0.02 | -0.07 | -0.24 | -0.16 | 0.16 | 0.05 | -0.12 | -0.06 | -0.26 | 0.16 | -0.16 | -0.03 | -0.20 | -0.08 | -0.24 | 0.01 | 0.11 |
| TCGA.64.5775.01 | 0.213 | 0.44 | -0.37 | -0.02 | -0.10 | 0.01 | 0.15 | -0.08 | 0.23 | 0.14 | -0.06 | 0.02 | -0.37 | 0.03 | -0.05 | -0.01 | 0.08 | 0.15 | -0.06 | -0.14 | -0.02 | -0.12 | 0.10 | -0.04 | -0.14 | -0.03 | 0.16 | -0.02 | 0.08 |
| TCGA.64.5778.01 | -0.129 | -0.25 | 0.44 | -0.15 | -0.12 | -0.09 | -0.14 | -0.11 | -0.21 | 0.09 | -0.17 | -0.24 | 0.44 | -0.03 | 0.00 | 0.13 | 0.28 | 0.01 | -0.02 | -0.04 | -0.11 | 0.16 | -0.02 | -0.08 | -0.16 | -0.17 | -0.15 | -0.11 | -0.02 |
| TCGA.64.5781.01 | 0.370 | -0.20 | -0.02 | 0.03 | -0.29 | -0.08 | -0.11 | -0.04 | -0.27 | -0.42 | -0.10 | 0.07 | -0.02 | -0.01 | -0.07 | 0.05 | 0.21 | 0.12 | -0.21 | -0.13 | -0.19 | -0.46 | -0.16 | -0.15 | -0.16 | -0.13 | 0.09 | -0.23 | -0.31 |
| TCGA.64.5815.01 | 0.087 | 0.45 | 0.24 | 0.34 | 0.32 | 0.37 | 0.39 | 0.24 | 0.32 | 0.45 | 0.26 | 0.28 | 0.24 | 0.23 | 0.42 | 0.46 | 0.26 | 0.35 | 0.11 | 0.27 | 0.43 | 0.36 | 0.39 | 0.36 | 0.43 | 0.46 | 0.28 | 0.24 | -0.21 |
| TCGA.66.2734.01 | 0.531 | -0.17 | -0.04 | -0.33 | -0.36 | -0.30 | -0.34 | -0.35 | -0.19 | -0.45 | -0.38 | -0.36 | -0.04 | -0.26 | -0.40 | -0.22 | -0.06 | -0.18 | -0.21 | -0.24 | -0.23 | -0.27 | -0.35 | -0.25 | -0.20 | -0.32 | -0.30 | -0.11 | -0.02 |
| TCGA.66.2742.01 | 0.280 | 0.16 | -0.11 | -0.08 | 0.17 | 0.02 | -0.01 | 0.07 | 0.05 | -0.02 | -0.01 | 0.06 | -0.11 | -0.14 | 0.03 | -0.08 | -0.04 | 0.02 | 0.19 | 0.16 | -0.10 | -0.12 | -0.04 | -0.05 | -0.11 | -0.07 | 0.00 | 0.10 | -0.09 |
| TCGA.66.2757.01 | 0.597 | 0.49 | -0.16 | 0.02 | 0.18 | -0.09 | 0.09 | 0.05 | 0.16 | 0.12 | 0.16 | 0.09 | -0.16 | 0.18 | 0.08 | -0.05 | -0.21 | -0.06 | 0.20 | 0.22 | -0.07 | 0.14 | 0.00 | 0.11 | -0.08 | 0.04 | 0.06 | 0.23 | 0.33 |
| TCGA.66.2758.01 | -0.349 | -0.34 | 0.05 | -0.07 | -0.20 | -0.04 | -0.08 | 0.09 | -0.15 | 0.18 | -0.13 | -0.20 | 0.05 | -0.22 | -0.15 | 0.02 | -0.06 | -0.21 | -0.33 | -0.18 | 0.20 | 0.08 | -0.07 | -0.26 | 0.06 | 0.02 | -0.18 | -0.28 | -0.29 |
| TCGA.66.2759.01 | 0.248 | 0.16 | -0.09 | -0.04 | 0.15 | -0.06 | -0.02 | 0.08 | 0.03 | 0.13 | 0.10 | 0.09 | -0.09 | 0.22 | 0.03 | -0.23 | -0.39 | -0.25 | 0.33 | 0.14 | -0.19 | 0.21 | 0.00 | 0.16 | -0.08 | -0.04 | 0.01 | 0.30 | 0.36 |
| TCGA.66.2771.01 | -0.136 | -0.19 | 0.29 | 0.02 | -0.24 | 0.06 | -0.03 | 0.01 | -0.17 | -0.02 | -0.10 | -0.15 | 0.29 | -0.14 | -0.20 | 0.23 | 0.37 | 0.26 | -0.22 | -0.15 | 0.18 | 0.05 | 0.03 | -0.03 | -0.03 | -0.01 | -0.07 | -0.24 | -0.05 |
| TCGA.66.2778.01 | -0.217 | -0.38 | -0.05 | -0.16 | 0.01 | -0.28 | -0.27 | -0.07 | -0.12 | -0.34 | -0.30 | -0.09 | -0.05 | 0.11 | -0.24 | -0.23 | -0.11 | -0.16 | 0.12 | -0.16 | -0.22 | -0.17 | -0.17 | -0.19 | -0.23 | -0.32 | -0.29 | -0.11 | 0.08 |
| TCGA.66.2782.01 | 0.474 | 0.07 | -0.19 | -0.12 | -0.33 | 0.01 | 0.08 | -0.22 | -0.06 | 0.07 | -0.14 | -0.30 | -0.19 | -0.15 | 0.02 | -0.13 | -0.11 | -0.17 | 0.08 | -0.19 | 0.24 | 0.04 | 0.13 | -0.16 | 0.03 | 0.17 | -0.02 | -0.18 | 0.20 |
| TCGA.66.2783.01 | -0.072 | -0.16 | 0.16 | 0.03 | -0.02 | 0.02 | 0.07 | 0.19 | 0.03 | -0.09 | 0.13 | 0.13 | 0.16 | 0.17 | 0.14 | 0.35 | 0.42 | 0.32 | 0.29 | 0.14 | 0.22 | -0.07 | 0.11 | 0.00 | 0.15 | 0.17 | 0.15 | 0.13 | -0.18 |
| TCGA.66.2786.01 | -0.011 | 0.15 | 0.22 | 0.16 | 0.21 | 0.18 | 0.12 | 0.08 | 0.16 | 0.03 | 0.03 | 0.07 | 0.22 | 0.22 | 0.11 | 0.18 | 0.25 | 0.23 | 0.25 | -0.07 | 0.12 | -0.03 | 0.17 | 0.08 | 0.15 | 0.00 | 0.13 | -0.06 | -0.08 |
| TCGA.66.2787.01 | 0.277 | 0.48 | -0.21 | 0.19 | 0.34 | 0.11 | 0.14 | 0.01 | 0.22 | -0.03 | 0.22 | 0.12 | -0.21 | 0.11 | 0.06 | 0.10 | -0.03 | 0.28 | 0.28 | 0.15 | -0.02 | -0.11 | 0.02 | -0.09 | -0.03 | -0.24 | 0.22 | 0.21 | 0.20 |
| TCGA.66.2788.01 | -0.191 | -0.60 | 0.28 | -0.16 | -0.33 | -0.18 | -0.15 | -0.18 | -0.17 | -0.35 | -0.29 | -0.27 | 0.28 | -0.18 | -0.14 | -0.21 | -0.04 | -0.27 | -0.27 | -0.34 | 0.07 | -0.33 | -0.20 | -0.25 | -0.04 | -0.17 | -0.19 | -0.29 | -0.05 |
| TCGA.66.2790.01 | -0.159 | 0.18 | 0.10 | -0.01 | 0.30 | -0.07 | -0.01 | 0.22 | 0.11 | 0.04 | 0.09 | 0.23 | 0.10 | 0.13 | 0.00 | -0.02 | -0.15 | 0.04 | 0.22 | 0.11 | -0.08 | 0.22 | 0.07 | 0.21 | -0.18 | -0.08 | -0.02 | 0.30 | 0.21 |
| TCGA.66.2792.01 | 0.428 | 0.37 | -0.30 | 0.00 | 0.19 | 0.05 | 0.08 | 0.03 | 0.36 | 0.16 | -0.02 | 0.05 | -0.30 | 0.06 | 0.13 | -0.02 | -0.13 | -0.11 | -0.08 | 0.02 | -0.17 | 0.25 | 0.00 | 0.23 | -0.09 | 0.05 | -0.02 | 0.07 | 0.16 |
| TCGA.66.2794.01 | 0.158 | -0.35 | 0.17 | -0.13 | -0.01 | -0.05 | -0.15 | -0.06 | -0.10 | 0.01 | -0.01 | -0.02 | 0.17 | 0.09 | 0.00 | 0.01 | 0.00 | 0.00 | -0.02 | 0.05 | -0.13 | -0.04 | -0.10 | 0.21 | -0.02 | -0.21 | -0.04 | 0.09 | 0.09 |
| TCGA.66.2800.01 | -0.383 | 0.29 | 0.21 | 0.31 | 0.22 | 0.32 | 0.39 | 0.30 | 0.26 | 0.29 | 0.18 | 0.27 | 0.21 | 0.26 | 0.35 | 0.28 | 0.01 | 0.15 | 0.05 | 0.22 | 0.44 | 0.27 | 0.39 | 0.21 | 0.41 | 0.41 | 0.19 | 0.23 | -0.14 |
| TCGA.67.6215.01 | -0.237 | 0.18 | 0.00 | -0.10 | 0.14 | -0.07 | 0.01 | 0.05 | 0.11 | 0.11 | 0.08 | 0.10 | 0.00 | 0.02 | 0.08 | -0.31 | -0.23 | -0.12 | 0.14 | -0.03 | -0.07 | 0.13 | 0.09 | 0.05 | -0.08 | -0.11 | -0.09 | 0.21 | 0.03 |
| TCGA.67.6216.01 | 0.042 | -0.14 | 0.12 | 0.00 | 0.06 | 0.00 | -0.09 | -0.08 | -0.14 | -0.24 | -0.21 | -0.20 | 0.12 | 0.07 | -0.23 | 0.00 | 0.23 | 0.11 | -0.02 | -0.13 | -0.05 | -0.11 | -0.22 | 0.03 | -0.12 | -0.17 | -0.02 | -0.19 | -0.21 |
| TCGA.67.6217.01 | 0.342 | 0.22 | -0.15 | 0.16 | 0.00 | 0.06 | 0.14 | 0.04 | -0.38 | -0.02 | 0.14 | 0.16 | -0.15 | 0.36 | 0.10 | 0.25 | 0.34 | 0.37 | 0.17 | 0.20 | 0.01 | -0.10 | 0.17 | 0.21 | -0.07 | -0.01 | 0.26 | -0.10 | 0.19 |
| TCGA.68.7755.01 | 0.288 | 0.32 | 0.46 | 0.06 | 0.10 | 0.16 | 0.10 | 0.04 | 0.16 | -0.11 | 0.01 | -0.04 | 0.46 | -0.01 | 0.13 | 0.28 | 0.22 | 0.03 | -0.03 | -0.13 | 0.20 | 0.09 | 0.13 | -0.01 | 0.03 | 0.05 | 0.02 | -0.01 | -0.37 |
| TCGA.68.7756.01 | -0.570 | -0.27 | -0.11 | -0.14 | -0.10 | -0.21 | -0.23 | -0.04 | 0.07 | -0.18 | -0.11 | -0.05 | -0.11 | 0.11 | -0.31 | -0.28 | -0.14 | 0.04 | 0.09 | -0.06 | -0.22 | -0.17 | -0.19 | -0.25 | -0.18 | -0.32 | -0.22 | 0.02 | 0.07 |
| TCGA.68.7757.01 | 0.248 | 0.18 | -0.34 | 0.16 | 0.20 | 0.09 | 0.13 | -0.19 | -0.01 | -0.09 | -0.04 | -0.20 | -0.34 | 0.17 | -0.08 | 0.21 | 0.13 | 0.22 | 0.16 | 0.02 | -0.07 | -0.22 | -0.03 | 0.19 | -0.11 | -0.07 | 0.07 | -0.04 | 0.07 |
| TCGA.68.8250.01 | 0.134 | 0.26 | -0.33 | -0.02 | 0.14 | -0.07 | -0.17 | 0.01 | -0.05 | 0.14 | 0.05 | -0.08 | -0.33 | 0.25 | -0.12 | -0.10 | -0.14 | -0.08 | 0.19 | 0.10 | -0.18 | 0.19 | -0.07 | 0.03 | -0.11 | -0.11 | -0.07 | 0.12 | 0.24 |
| TCGA.68.8251.01 | 0.037 | -0.31 | -0.33 | -0.19 | -0.17 | -0.26 | -0.22 | -0.09 | -0.22 | -0.13 | -0.18 | -0.04 | -0.33 | -0.11 | -0.27 | -0.37 | -0.45 | -0.27 | -0.06 | -0.21 | -0.31 | -0.26 | -0.32 | -0.08 | -0.23 | -0.25 | -0.27 | -0.06 | 0.17 |
| TCGA.68.A59I.01 | 0.178 | 0.32 | 0.21 | 0.09 | 0.32 | 0.15 | 0.21 | 0.31 | 0.23 | 0.19 | 0.30 | 0.25 | 0.21 | 0.12 | 0.10 | 0.20 | 0.14 | 0.03 | 0.20 | 0.22 | 0.26 | 0.18 | 0.18 | 0.03 | 0.27 | 0.09 | 0.15 | 0.07 | -0.06 |
| TCGA.68.A59J.01 | -0.378 | 0.14 | -0.11 | -0.02 | 0.08 | -0.05 | -0.16 | -0.01 | -0.06 | 0.00 | 0.02 | 0.00 | -0.11 | 0.10 | 0.01 | 0.08 | 0.31 | 0.22 | 0.20 | 0.11 | -0.12 | -0.06 | -0.04 | -0.08 | -0.09 | -0.24 | 0.05 | 0.11 | -0.06 |
| TCGA.69.7760.01 | -0.260 | -0.11 | -0.06 | -0.12 | -0.01 | -0.14 | -0.15 | 0.08 | -0.21 | -0.03 | 0.12 | -0.02 | -0.06 | -0.19 | -0.09 | -0.18 | -0.25 | -0.10 | 0.02 | -0.10 | -0.02 | -0.08 | -0.20 | -0.25 | 0.02 | 0.06 | -0.11 | -0.01 | 0.11 |
| TCGA.69.7763.01 | -0.475 | -0.46 | 0.11 | -0.26 | -0.10 | -0.26 | -0.28 | -0.14 | -0.07 | -0.17 | -0.22 | -0.18 | 0.11 | -0.15 | -0.30 | -0.11 | 0.02 | 0.07 | -0.06 | -0.16 | -0.20 | -0.08 | -0.22 | -0.27 | -0.16 | -0.33 | -0.33 | -0.28 | 0.02 |
| TCGA.69.7764.01 | 0.240 | -0.09 | 0.24 | 0.00 | 0.10 | -0.01 | 0.03 | -0.02 | 0.08 | -0.18 | -0.08 | -0.04 | 0.24 | 0.18 | 0.03 | 0.00 | 0.22 | 0.11 | 0.22 | 0.19 | -0.12 | 0.09 | 0.10 | 0.11 | -0.20 | -0.03 | 0.00 | 0.02 | 0.12 |
| TCGA.69.7765.01 | -0.035 | 0.12 | -0.37 | -0.05 | 0.14 | -0.04 | 0.02 | 0.06 | 0.15 | -0.02 | -0.06 | -0.09 | -0.37 | -0.03 | 0.00 | -0.18 | -0.09 | -0.02 | 0.16 | -0.08 | -0.24 | 0.17 | -0.07 | -0.02 | -0.11 | -0.05 | -0.05 | 0.14 | 0.20 |
| TCGA.69.7973.01 | -0.105 | 0.36 | 0.26 | 0.37 | 0.29 | 0.37 | 0.37 | 0.35 | 0.03 | 0.33 | 0.28 | 0.19 | 0.26 | 0.23 | 0.31 | 0.44 | 0.48 | 0.33 | 0.08 | 0.21 | 0.41 | 0.14 | 0.39 | 0.29 | 0.41 | 0.39 | 0.36 | -0.09 | -0.31 |
| TCGA.69.7974.01 | 0.095 | 0.31 | 0.25 | 0.18 | 0.25 | 0.15 | 0.26 | -0.10 | 0.19 | -0.01 | -0.01 | 0.01 | 0.25 | 0.16 | 0.15 | 0.31 | 0.29 | 0.32 | 0.28 | 0.02 | 0.16 | 0.32 | 0.20 | 0.23 | 0.00 | 0.07 | 0.15 | 0.03 | -0.01 |
| TCGA.69.7978.01 | -0.537 | 0.27 | 0.18 | 0.36 | 0.15 | 0.37 | 0.36 | 0.28 | 0.01 | 0.31 | 0.34 | 0.30 | 0.18 | 0.14 | 0.34 | 0.47 | 0.35 | 0.37 | 0.11 | 0.15 | 0.45 | 0.22 | 0.32 | 0.21 | 0.42 | 0.42 | 0.33 | 0.05 | -0.32 |
| TCGA.69.7980.01 | -0.017 | 0.29 | 0.25 | 0.00 | 0.19 | 0.02 | 0.01 | -0.24 | 0.22 | -0.15 | -0.18 | -0.18 | 0.25 | 0.21 | -0.18 | 0.12 | 0.19 | 0.15 | 0.08 | 0.10 | -0.09 | 0.33 | -0.02 | 0.09 | -0.17 | -0.15 | -0.06 | -0.06 | -0.06 |
| TCGA.69.8253.01 | -0.381 | -0.23 | 0.12 | -0.05 | 0.16 | -0.03 | -0.11 | 0.18 | -0.05 | 0.29 | -0.02 | 0.20 | 0.12 | 0.10 | -0.20 | -0.17 | -0.15 | -0.25 | -0.20 | 0.00 | -0.13 | 0.01 | -0.09 | -0.09 | -0.05 | -0.24 | -0.11 | 0.06 | -0.19 |
| TCGA.69.8254.01 | 0.570 | -0.17 | 0.00 | -0.02 | 0.22 | -0.03 | -0.06 | -0.13 | 0.09 | 0.24 | -0.10 | -0.22 | 0.00 | -0.10 | -0.12 | 0.13 | 0.21 | 0.24 | -0.11 | 0.02 | -0.05 | 0.25 | -0.04 | 0.06 | 0.08 | 0.06 | -0.12 | -0.02 | -0.06 |
| TCGA.69.8255.01 | -0.036 | 0.58 | -0.16 | 0.01 | 0.16 | -0.03 | 0.08 | 0.27 | 0.15 | 0.01 | 0.31 | 0.30 | -0.16 | 0.00 | 0.06 | 0.12 | -0.09 | 0.12 | 0.16 | 0.15 | 0.09 | -0.11 | 0.02 | -0.11 | 0.03 | 0.06 | 0.28 | 0.06 | -0.20 |
| TCGA.69.A59K.01 | 0.444 | -0.26 | 0.11 | -0.17 | -0.27 | -0.16 | -0.05 | -0.28 | -0.32 | -0.44 | -0.19 | -0.22 | 0.11 | 0.01 | -0.18 | 0.01 | 0.05 | 0.11 | 0.01 | -0.13 | -0.28 | -0.21 | -0.18 | 0.17 | -0.24 | -0.28 | -0.19 | 0.02 | 0.15 |
| TCGA.6A.AB49.01 | -0.497 | 0.47 | 0.21 | 0.36 | 0.24 | 0.37 | 0.37 | 0.26 | 0.22 | 0.31 | 0.35 | 0.33 | 0.21 | 0.16 | 0.37 | 0.48 | 0.36 | 0.39 | 0.22 | 0.17 | 0.48 | 0.27 | 0.35 | 0.29 | 0.44 | 0.47 | 0.28 | 0.25 | -0.31 |
| TCGA.70.6722.01 | 0.580 | 0.71 | -0.08 | 0.22 | 0.35 | 0.16 | 0.27 | 0.21 | 0.27 | 0.41 | 0.31 | 0.32 | -0.08 | 0.24 | 0.21 | 0.32 | 0.31 | 0.43 | 0.26 | 0.23 | 0.03 | 0.30 | 0.20 | 0.33 | 0.19 | 0.16 | 0.31 | 0.27 | 0.22 |
| TCGA.70.6723.01 | -0.086 | 0.24 | 0.24 | 0.20 | 0.01 | 0.27 | 0.17 | -0.04 | -0.25 | 0.00 | -0.02 | -0.12 | 0.24 | 0.28 | 0.18 | 0.33 | 0.37 | 0.31 | 0.15 | 0.04 | 0.32 | 0.08 | 0.27 | 0.23 | 0.23 | 0.24 | 0.22 | -0.12 | -0.12 |
| TCGA.71.6725.01 | -0.239 | -0.27 | 0.18 | -0.06 | -0.03 | -0.03 | -0.10 | 0.05 | -0.06 | -0.27 | -0.03 | -0.03 | 0.18 | 0.26 | -0.17 | 0.08 | 0.09 | 0.06 | 0.01 | -0.03 | 0.04 | -0.09 | 0.10 | -0.11 | -0.03 | -0.13 | 0.00 | -0.18 | -0.11 |
| TCGA.71.8520.01 | -0.135 | -0.44 | 0.22 | -0.08 | -0.25 | -0.12 | -0.16 | -0.19 | -0.25 | -0.10 | -0.38 | -0.28 | 0.22 | -0.16 | -0.20 | -0.08 | 0.22 | -0.14 | -0.21 | -0.23 | -0.03 | -0.12 | -0.05 | -0.11 | -0.04 | -0.12 | -0.20 | -0.21 | -0.01 |
| TCGA.73.4658.01 | 0.317 | 0.05 | -0.16 | -0.06 | -0.04 | -0.07 | -0.04 | 0.00 | -0.16 | -0.16 | 0.07 | 0.07 | -0.16 | 0.22 | 0.12 | -0.17 | -0.36 | -0.13 | 0.03 | 0.15 | -0.21 | -0.20 | -0.08 | 0.08 | -0.17 | -0.04 | 0.05 | 0.10 | 0.19 |
| TCGA.73.4659.01 | -0.565 | -0.49 | 0.08 | -0.24 | -0.22 | -0.26 | -0.25 | 0.02 | -0.13 | -0.26 | -0.20 | -0.20 | 0.08 | -0.02 | -0.24 | -0.31 | -0.28 | -0.16 | -0.29 | -0.18 | -0.18 | -0.22 | -0.25 | -0.24 | -0.21 | -0.25 | -0.33 | -0.24 | -0.11 |
| TCGA.73.4662.01 | -0.283 | -0.34 | -0.29 | -0.15 | -0.07 | -0.16 | -0.08 | 0.01 | -0.14 | -0.02 | 0.02 | 0.12 | -0.29 | -0.03 | -0.12 | -0.26 | -0.24 | -0.23 | -0.01 | -0.06 | -0.17 | -0.06 | -0.12 | -0.06 | -0.07 | -0.06 | -0.04 | 0.10 | 0.01 |
| TCGA.73.4666.01 | -0.326 | 0.41 | -0.23 | 0.16 | 0.20 | 0.09 | 0.03 | 0.13 | 0.00 | 0.21 | 0.17 | 0.03 | -0.23 | 0.00 | 0.17 | 0.19 | 0.05 | 0.20 | -0.02 | 0.18 | 0.16 | 0.07 | 0.12 | -0.06 | 0.13 | 0.23 | 0.18 | 0.01 | -0.15 |
| TCGA.73.4668.01 | 0.278 | -0.45 | -0.40 | -0.28 | 0.01 | -0.23 | -0.31 | -0.09 | 0.03 | -0.05 | -0.20 | -0.18 | -0.40 | -0.32 | -0.21 | -0.25 | -0.21 | -0.43 | -0.30 | -0.16 | -0.35 | 0.03 | -0.22 | -0.27 | -0.28 | -0.34 | -0.32 | -0.07 | 0.21 |
| TCGA.73.4670.01 | -0.152 | 0.56 | -0.22 | 0.07 | 0.09 | 0.14 | 0.18 | 0.16 | 0.28 | 0.12 | 0.29 | 0.18 | -0.22 | -0.11 | 0.08 | 0.23 | 0.08 | 0.17 | 0.02 | 0.21 | -0.06 | -0.10 | 0.13 | 0.18 | 0.03 | 0.04 | 0.22 | 0.09 | 0.00 |
| TCGA.73.4675.01 | -0.418 | -0.48 | -0.20 | -0.18 | -0.34 | -0.17 | -0.25 | -0.20 | -0.44 | -0.34 | -0.12 | -0.26 | -0.20 | -0.25 | -0.13 | 0.00 | 0.12 | 0.13 | -0.22 | -0.15 | -0.17 | -0.33 | -0.29 | -0.15 | -0.24 | -0.19 | -0.13 | -0.33 | 0.04 |
| TCGA.73.4676.01 | -0.015 | 0.08 | 0.22 | -0.08 | 0.03 | -0.04 | 0.06 | -0.18 | -0.33 | -0.35 | -0.08 | -0.05 | 0.22 | -0.05 | -0.06 | -0.08 | -0.07 | 0.07 | 0.08 | -0.13 | 0.04 | 0.00 | -0.06 | 0.01 | 0.03 | 0.01 | 0.07 | -0.09 | 0.04 |
| TCGA.73.4677.01 | 0.470 | 0.15 | -0.16 | 0.09 | 0.07 | 0.09 | 0.13 | 0.18 | 0.06 | 0.26 | 0.17 | 0.14 | -0.16 | 0.05 | 0.10 | 0.34 | 0.41 | 0.29 | 0.15 | 0.06 | 0.02 | 0.17 | 0.14 | 0.11 | 0.06 | 0.22 | 0.30 | 0.07 | -0.30 |
| TCGA.73.7498.01 | -0.500 | -0.19 | 0.37 | 0.22 | -0.04 | 0.28 | 0.31 | 0.17 | 0.06 | 0.28 | 0.08 | 0.16 | 0.37 | -0.03 | 0.22 | 0.28 | 0.05 | -0.05 | -0.19 | -0.02 | 0.41 | 0.09 | 0.24 | -0.03 | 0.39 | 0.28 | 0.13 | -0.03 | -0.39 |
| TCGA.73.7499.01 | 0.043 | -0.59 | -0.28 | -0.35 | -0.35 | -0.29 | -0.26 | -0.33 | -0.43 | -0.48 | -0.41 | -0.44 | -0.28 | -0.34 | -0.33 | -0.25 | -0.14 | -0.23 | -0.31 | -0.38 | -0.33 | -0.39 | -0.34 | -0.32 | -0.32 | -0.30 | -0.29 | -0.35 | 0.01 |
| TCGA.73.A9RS.01 | 0.115 | 0.25 | -0.09 | -0.04 | 0.15 | -0.05 | 0.04 | 0.10 | 0.13 | 0.40 | 0.18 | 0.08 | -0.09 | 0.19 | 0.02 | -0.23 | -0.18 | -0.09 | 0.36 | 0.20 | -0.13 | 0.11 | 0.11 | 0.10 | -0.05 | -0.01 | 0.04 | 0.22 | 0.19 |
| TCGA.75.5122.01 | 0.342 | 0.04 | 0.24 | 0.15 | -0.04 | 0.19 | 0.18 | -0.31 | -0.17 | 0.01 | -0.19 | -0.31 | 0.24 | 0.16 | 0.10 | 0.44 | 0.51 | 0.44 | 0.15 | 0.09 | 0.13 | 0.24 | 0.24 | 0.25 | 0.18 | 0.16 | 0.14 | -0.23 | 0.10 |
| TCGA.75.5125.01 | -0.135 | -0.39 | -0.10 | -0.19 | -0.06 | -0.26 | -0.27 | 0.07 | 0.08 | 0.14 | -0.19 | -0.03 | -0.10 | -0.36 | -0.31 | -0.36 | -0.35 | -0.38 | -0.24 | -0.29 | -0.28 | -0.19 | -0.35 | -0.22 | -0.25 | -0.23 | -0.23 | -0.19 | 0.05 |
| TCGA.75.5126.01 | -0.035 | 0.15 | 0.23 | 0.16 | -0.04 | 0.19 | 0.26 | -0.13 | -0.21 | -0.07 | 0.02 | -0.13 | 0.23 | -0.01 | 0.16 | 0.38 | 0.41 | 0.37 | 0.22 | -0.04 | 0.26 | -0.15 | 0.07 | 0.16 | 0.19 | 0.19 | 0.24 | -0.19 | -0.14 |
| TCGA.75.5146.01 | 0.260 | 0.37 | 0.44 | 0.24 | 0.14 | 0.25 | 0.29 | 0.16 | 0.13 | 0.28 | 0.26 | 0.22 | 0.44 | 0.03 | 0.16 | 0.39 | 0.42 | 0.41 | 0.29 | 0.19 | 0.30 | 0.05 | 0.19 | 0.13 | 0.23 | 0.23 | 0.37 | -0.04 | -0.30 |
| TCGA.75.5147.01 | 0.379 | -0.52 | -0.32 | -0.33 | -0.42 | -0.31 | -0.35 | -0.37 | -0.43 | -0.33 | -0.33 | -0.42 | -0.32 | -0.38 | -0.16 | -0.19 | -0.04 | -0.18 | -0.33 | -0.31 | -0.28 | -0.33 | -0.38 | -0.32 | -0.29 | -0.19 | -0.30 | -0.35 | -0.04 |
| TCGA.75.6203.01 | -0.100 | 0.13 | 0.25 | 0.10 | 0.08 | 0.08 | 0.08 | -0.09 | 0.00 | 0.25 | -0.14 | -0.21 | 0.25 | -0.13 | -0.01 | 0.15 | 0.05 | -0.07 | -0.19 | -0.04 | 0.08 | 0.23 | 0.15 | 0.06 | 0.12 | 0.12 | -0.11 | -0.03 | -0.06 |
| TCGA.75.6205.01 | -0.541 | -0.60 | -0.01 | -0.18 | -0.44 | -0.17 | -0.26 | -0.23 | -0.47 | -0.35 | -0.03 | -0.26 | -0.01 | -0.35 | -0.22 | -0.16 | 0.09 | -0.02 | -0.29 | -0.30 | -0.13 | -0.45 | -0.34 | -0.29 | -0.18 | -0.28 | -0.21 | -0.40 | -0.11 |
| TCGA.75.6206.01 | 0.341 | 0.09 | 0.06 | 0.09 | -0.08 | 0.07 | 0.13 | 0.22 | 0.18 | 0.19 | 0.21 | 0.20 | 0.06 | 0.01 | 0.08 | 0.06 | -0.22 | -0.21 | -0.02 | 0.04 | 0.22 | 0.05 | 0.06 | 0.00 | 0.14 | 0.19 | 0.10 | 0.11 | -0.14 |
| TCGA.75.6207.01 | -0.034 | -0.36 | -0.11 | -0.11 | -0.12 | -0.26 | -0.29 | -0.15 | -0.07 | -0.37 | -0.20 | -0.01 | -0.11 | -0.02 | -0.27 | -0.25 | -0.22 | -0.17 | -0.07 | -0.12 | -0.34 | 0.02 | -0.20 | -0.13 | -0.31 | -0.33 | -0.23 | -0.06 | -0.10 |
| TCGA.75.6211.01 | -0.078 | -0.07 | 0.02 | -0.05 | 0.33 | -0.03 | -0.10 | -0.02 | -0.09 | 0.02 | -0.15 | 0.09 | 0.02 | 0.08 | 0.00 | -0.36 | -0.10 | -0.16 | 0.14 | 0.05 | -0.20 | 0.08 | -0.04 | 0.07 | -0.09 | -0.19 | -0.17 | 0.21 | 0.36 |
| TCGA.75.6212.01 | 0.494 | 0.02 | -0.15 | -0.09 | -0.07 | -0.06 | -0.16 | -0.04 | 0.31 | -0.20 | -0.10 | 0.05 | -0.15 | 0.05 | -0.25 | -0.13 | -0.04 | -0.03 | 0.06 | -0.17 | -0.25 | -0.06 | -0.20 | 0.02 | -0.32 | -0.25 | -0.17 | -0.05 | 0.11 |
| TCGA.75.6214.01 | -0.320 | -0.28 | 0.28 | 0.18 | -0.13 | 0.14 | 0.11 | -0.07 | 0.22 | -0.01 | 0.01 | -0.06 | 0.28 | 0.21 | 0.07 | 0.44 | 0.53 | 0.37 | 0.16 | -0.01 | 0.28 | 0.05 | 0.23 | 0.01 | 0.04 | 0.07 | 0.26 | -0.24 | -0.26 |
| TCGA.75.7025.01 | -0.194 | 0.01 | 0.00 | -0.19 | 0.11 | -0.22 | -0.23 | -0.08 | -0.23 | -0.09 | -0.15 | -0.03 | 0.00 | -0.12 | -0.11 | -0.30 | -0.34 | -0.14 | 0.20 | 0.03 | -0.35 | -0.10 | -0.22 | -0.10 | -0.30 | -0.17 | -0.16 | 0.24 | 0.32 |
| TCGA.75.7027.01 | 0.340 | -0.14 | 0.04 | -0.01 | -0.03 | -0.07 | -0.11 | 0.06 | -0.08 | -0.07 | -0.06 | 0.05 | 0.04 | -0.16 | -0.13 | -0.12 | -0.15 | -0.16 | 0.18 | 0.02 | -0.09 | -0.23 | -0.05 | 0.06 | -0.12 | -0.18 | 0.05 | -0.02 | -0.13 |
| TCGA.75.7030.01 | -0.416 | 0.18 | 0.12 | -0.05 | -0.02 | 0.03 | -0.03 | -0.23 | -0.37 | -0.05 | 0.15 | -0.17 | 0.12 | -0.28 | -0.01 | 0.19 | -0.04 | -0.05 | -0.06 | -0.01 | 0.01 | 0.12 | -0.03 | -0.13 | -0.09 | -0.04 | 0.04 | -0.17 | -0.16 |
| TCGA.75.7031.01 | -0.170 | -0.37 | 0.38 | -0.14 | -0.29 | -0.18 | -0.14 | -0.11 | -0.27 | -0.28 | -0.02 | -0.24 | 0.38 | -0.31 | -0.08 | -0.15 | -0.11 | -0.21 | 0.04 | -0.11 | 0.01 | -0.36 | -0.11 | -0.16 | 0.00 | 0.07 | -0.08 | -0.04 | -0.14 |
| TCGA.77.6842.01 | 0.425 | 0.38 | -0.29 | 0.06 | 0.18 | -0.17 | -0.03 | 0.28 | 0.11 | 0.14 | 0.27 | 0.30 | -0.29 | -0.03 | -0.03 | -0.07 | -0.25 | 0.22 | 0.10 | 0.23 | -0.20 | 0.04 | -0.15 | 0.04 | 0.05 | 0.00 | 0.02 | 0.16 | -0.03 |
| TCGA.77.6843.01 | -0.144 | 0.51 | -0.50 | 0.03 | 0.18 | -0.06 | 0.06 | 0.30 | 0.27 | 0.21 | 0.28 | 0.34 | -0.50 | 0.10 | 0.10 | -0.16 | -0.24 | -0.15 | 0.13 | 0.06 | -0.20 | 0.12 | 0.03 | -0.06 | 0.01 | -0.06 | 0.09 | 0.19 | 0.26 |
| TCGA.77.6844.01 | 0.313 | -0.02 | 0.11 | 0.15 | 0.07 | 0.17 | 0.14 | 0.20 | 0.08 | 0.39 | 0.24 | 0.19 | 0.11 | 0.24 | 0.14 | 0.42 | 0.48 | 0.32 | 0.10 | -0.04 | 0.26 | 0.01 | 0.23 | 0.08 | 0.19 | 0.21 | 0.27 | -0.10 | -0.21 |
| TCGA.77.6845.01 | -0.121 | 0.29 | 0.22 | 0.26 | 0.15 | 0.31 | 0.33 | -0.01 | 0.16 | 0.35 | 0.11 | 0.13 | 0.22 | 0.09 | 0.35 | 0.27 | -0.04 | 0.19 | 0.05 | 0.18 | 0.30 | 0.34 | 0.32 | 0.30 | 0.30 | 0.41 | 0.15 | 0.23 | -0.13 |
| TCGA.77.7138.01 | -0.337 | -0.33 | -0.29 | -0.16 | -0.16 | -0.23 | -0.10 | -0.37 | -0.18 | -0.35 | -0.21 | -0.26 | -0.29 | -0.24 | -0.36 | -0.09 | 0.09 | 0.06 | 0.10 | -0.10 | -0.31 | -0.18 | -0.21 | -0.10 | -0.21 | -0.28 | -0.21 | 0.05 | 0.28 |
| TCGA.77.7139.01 | -0.253 | -0.10 | 0.22 | -0.15 | -0.02 | -0.12 | -0.18 | -0.12 | -0.06 | -0.02 | -0.01 | 0.06 | 0.22 | -0.22 | -0.25 | -0.21 | -0.22 | -0.19 | -0.09 | -0.22 | -0.08 | -0.15 | -0.09 | -0.21 | -0.24 | -0.18 | 0.00 | -0.13 | 0.16 |
| TCGA.77.7140.01 | -0.249 | 0.08 | 0.11 | -0.05 | 0.10 | -0.09 | -0.16 | -0.22 | 0.03 | 0.10 | -0.18 | -0.11 | 0.11 | 0.09 | -0.09 | -0.20 | -0.31 | -0.27 | -0.21 | 0.13 | -0.20 | 0.26 | -0.07 | 0.05 | -0.15 | -0.02 | -0.16 | 0.15 | 0.18 |
| TCGA.77.7141.01 | -0.280 | 0.33 | 0.24 | 0.20 | 0.03 | 0.28 | 0.31 | 0.09 | 0.24 | 0.40 | 0.19 | 0.09 | 0.24 | 0.04 | 0.32 | 0.13 | -0.12 | -0.03 | -0.05 | 0.18 | 0.32 | 0.36 | 0.33 | 0.17 | 0.31 | 0.40 | 0.19 | 0.10 | -0.15 |
| TCGA.77.7142.01 | 0.309 | 0.29 | -0.20 | 0.01 | 0.17 | -0.02 | 0.09 | 0.13 | -0.04 | 0.13 | 0.14 | 0.18 | -0.20 | -0.02 | 0.05 | 0.06 | 0.00 | 0.08 | 0.38 | 0.18 | -0.20 | -0.10 | -0.06 | 0.06 | -0.10 | 0.01 | 0.13 | 0.07 | 0.23 |
| TCGA.77.7335.01 | -0.045 | 0.20 | -0.01 | 0.11 | -0.11 | 0.22 | 0.24 | 0.33 | 0.04 | 0.08 | 0.41 | 0.22 | -0.01 | -0.01 | 0.18 | 0.24 | 0.09 | -0.05 | 0.00 | 0.07 | 0.21 | -0.28 | 0.04 | 0.17 | 0.25 | 0.22 | 0.21 | -0.15 | -0.30 |
| TCGA.77.7337.01 | -0.258 | -0.52 | 0.10 | -0.18 | -0.36 | -0.19 | -0.19 | -0.34 | -0.35 | -0.45 | -0.40 | -0.38 | 0.10 | -0.33 | -0.32 | -0.20 | -0.14 | -0.33 | -0.27 | -0.31 | 0.01 | -0.39 | -0.30 | -0.30 | -0.17 | -0.28 | -0.24 | -0.31 | -0.22 |
| TCGA.77.7338.01 | 0.249 | 0.06 | -0.10 | -0.15 | 0.06 | -0.18 | -0.20 | -0.07 | 0.03 | -0.04 | -0.11 | 0.00 | -0.10 | 0.21 | -0.03 | 0.05 | 0.04 | 0.03 | 0.19 | 0.20 | -0.21 | 0.07 | -0.19 | 0.09 | -0.16 | -0.09 | -0.14 | 0.21 | 0.25 |
| TCGA.77.7463.01 | -0.057 | 0.29 | 0.19 | 0.27 | 0.12 | 0.34 | 0.38 | 0.22 | 0.19 | 0.47 | 0.26 | 0.22 | 0.19 | 0.08 | 0.34 | 0.26 | 0.12 | 0.01 | -0.01 | 0.14 | 0.40 | 0.33 | 0.36 | 0.21 | 0.33 | 0.38 | 0.22 | 0.10 | -0.21 |
| TCGA.77.8008.01 | 0.064 | -0.32 | -0.20 | -0.27 | 0.02 | -0.27 | -0.22 | -0.24 | -0.29 | -0.37 | -0.13 | -0.27 | -0.20 | -0.01 | -0.02 | -0.38 | -0.39 | -0.31 | 0.21 | 0.04 | -0.27 | -0.15 | -0.22 | -0.06 | -0.28 | -0.24 | -0.21 | -0.07 | 0.21 |
| TCGA.77.8009.01 | 0.274 | -0.37 | -0.35 | -0.15 | -0.06 | -0.18 | -0.07 | -0.24 | -0.03 | -0.25 | -0.07 | -0.13 | -0.35 | 0.15 | -0.28 | -0.15 | -0.13 | 0.09 | 0.06 | -0.16 | -0.30 | -0.22 | -0.16 | 0.21 | -0.20 | -0.24 | -0.13 | 0.05 | 0.24 |
| TCGA.77.8128.01 | -0.301 | -0.22 | -0.11 | -0.20 | -0.06 | -0.24 | -0.19 | -0.27 | 0.01 | -0.23 | -0.31 | -0.30 | -0.11 | -0.22 | -0.18 | -0.34 | -0.43 | -0.39 | -0.27 | -0.26 | -0.15 | 0.04 | -0.26 | -0.21 | -0.24 | -0.22 | -0.25 | -0.09 | 0.30 |
| TCGA.77.8130.01 | -0.281 | -0.04 | 0.09 | 0.11 | -0.10 | 0.23 | 0.27 | 0.05 | -0.33 | -0.02 | 0.22 | 0.05 | 0.09 | -0.07 | 0.29 | 0.18 | -0.05 | -0.08 | -0.05 | 0.07 | 0.29 | -0.02 | 0.06 | 0.18 | 0.30 | 0.33 | 0.13 | -0.08 | -0.18 |
| TCGA.77.8133.01 | 0.041 | 0.59 | 0.05 | 0.44 | 0.35 | 0.41 | 0.46 | 0.14 | 0.33 | 0.38 | 0.32 | 0.24 | 0.05 | 0.44 | 0.43 | 0.52 | 0.48 | 0.51 | 0.35 | 0.28 | 0.40 | 0.36 | 0.41 | 0.39 | 0.38 | 0.43 | 0.31 | 0.27 | 0.00 |
| TCGA.77.8136.01 | 0.060 | 0.12 | 0.27 | 0.14 | -0.04 | 0.22 | 0.19 | -0.07 | -0.14 | -0.09 | -0.28 | -0.28 | 0.27 | -0.03 | 0.07 | 0.11 | 0.17 | -0.17 | 0.16 | -0.01 | 0.34 | -0.20 | 0.10 | 0.03 | 0.24 | 0.16 | 0.21 | -0.04 | -0.35 |
| TCGA.77.8138.01 | 0.442 | 0.53 | 0.14 | 0.25 | 0.20 | 0.10 | 0.27 | 0.28 | 0.34 | 0.27 | 0.09 | 0.23 | 0.14 | 0.31 | 0.11 | 0.23 | 0.26 | 0.34 | 0.26 | 0.11 | 0.17 | -0.16 | 0.18 | 0.10 | 0.20 | 0.11 | 0.30 | -0.07 | -0.21 |
| TCGA.77.8139.01 | 0.075 | -0.34 | -0.10 | -0.04 | -0.07 | 0.00 | -0.03 | 0.02 | -0.24 | -0.30 | -0.16 | -0.07 | -0.10 | 0.00 | -0.08 | -0.09 | 0.14 | 0.11 | 0.04 | -0.16 | 0.00 | -0.27 | 0.05 | -0.17 | 0.05 | -0.20 | -0.03 | -0.21 | -0.12 |
| TCGA.77.8140.01 | 0.269 | 0.13 | 0.09 | 0.00 | -0.08 | 0.11 | 0.11 | 0.14 | -0.03 | 0.06 | 0.11 | 0.13 | 0.09 | 0.07 | 0.15 | -0.28 | -0.12 | -0.09 | 0.18 | 0.27 | 0.19 | 0.10 | 0.02 | 0.09 | 0.10 | 0.26 | 0.14 | 0.22 | 0.14 |
| TCGA.77.8143.01 | 0.012 | 0.28 | 0.14 | 0.14 | 0.09 | 0.06 | 0.08 | -0.03 | 0.15 | -0.20 | -0.15 | 0.02 | 0.14 | 0.27 | -0.07 | 0.30 | 0.33 | 0.32 | 0.13 | -0.01 | 0.06 | 0.09 | 0.16 | 0.17 | 0.05 | 0.10 | 0.11 | -0.09 | 0.15 |
| TCGA.77.8144.01 | -0.155 | -0.24 | 0.17 | 0.06 | -0.10 | 0.01 | 0.09 | 0.10 | 0.37 | 0.12 | 0.05 | 0.18 | 0.17 | -0.18 | 0.00 | -0.07 | 0.02 | 0.12 | -0.12 | -0.17 | 0.11 | -0.03 | -0.02 | -0.20 | 0.03 | 0.09 | 0.04 | -0.04 | 0.03 |
| TCGA.77.8145.01 | 0.432 | 0.25 | -0.16 | -0.09 | 0.13 | -0.05 | -0.07 | 0.12 | 0.01 | 0.03 | 0.07 | 0.29 | -0.16 | -0.18 | -0.07 | -0.03 | -0.29 | -0.25 | -0.06 | 0.14 | -0.19 | 0.21 | -0.15 | 0.02 | -0.07 | -0.03 | -0.12 | 0.25 | 0.31 |
| TCGA.77.8146.01 | -0.170 | -0.15 | 0.32 | 0.06 | 0.09 | 0.10 | 0.25 | 0.30 | 0.23 | 0.00 | 0.08 | 0.31 | 0.32 | 0.13 | 0.00 | 0.05 | 0.08 | 0.13 | 0.11 | 0.11 | 0.22 | -0.09 | 0.18 | 0.00 | 0.11 | 0.02 | 0.27 | 0.05 | -0.28 |
| TCGA.77.8148.01 | 0.149 | -0.48 | 0.10 | -0.19 | 0.12 | -0.26 | -0.22 | -0.01 | 0.16 | -0.12 | -0.03 | 0.10 | 0.10 | -0.19 | -0.13 | -0.35 | -0.23 | -0.21 | 0.10 | -0.13 | -0.15 | -0.01 | -0.13 | 0.03 | -0.04 | -0.12 | -0.24 | 0.16 | 0.19 |
| TCGA.77.8150.01 | -0.462 | -0.22 | 0.31 | 0.04 | -0.04 | 0.05 | 0.04 | 0.03 | 0.00 | -0.18 | -0.03 | 0.08 | 0.31 | 0.04 | 0.09 | 0.10 | 0.09 | 0.03 | 0.00 | 0.00 | 0.16 | -0.16 | 0.04 | -0.03 | 0.11 | 0.03 | 0.16 | -0.07 | -0.23 |
| TCGA.77.8153.01 | 0.072 | 0.14 | -0.01 | 0.03 | 0.22 | -0.08 | -0.04 | 0.10 | 0.18 | 0.05 | 0.03 | 0.16 | -0.01 | 0.20 | -0.05 | -0.13 | -0.34 | -0.14 | 0.21 | 0.04 | -0.10 | -0.09 | 0.06 | 0.06 | 0.01 | -0.20 | 0.03 | 0.16 | 0.18 |
| TCGA.77.8154.01 | 0.346 | 0.36 | 0.35 | 0.39 | 0.31 | 0.42 | 0.43 | 0.25 | 0.27 | 0.41 | 0.36 | 0.17 | 0.35 | 0.31 | 0.44 | 0.49 | 0.47 | 0.46 | 0.30 | 0.29 | 0.49 | 0.35 | 0.45 | 0.34 | 0.48 | 0.52 | 0.40 | 0.16 | -0.10 |
| TCGA.77.8156.01 | -0.051 | 0.16 | 0.01 | 0.01 | -0.10 | 0.16 | 0.06 | 0.03 | -0.36 | 0.10 | 0.24 | 0.06 | 0.01 | -0.20 | 0.00 | 0.27 | 0.08 | 0.03 | 0.07 | 0.17 | 0.18 | 0.04 | 0.08 | 0.08 | 0.16 | 0.23 | 0.12 | 0.00 | -0.05 |
| TCGA.77.A5FZ.01 | 0.066 | 0.58 | 0.23 | 0.34 | 0.18 | 0.35 | 0.41 | 0.23 | 0.09 | 0.20 | 0.38 | 0.24 | 0.23 | 0.28 | 0.30 | 0.37 | 0.32 | 0.31 | 0.35 | 0.27 | 0.39 | 0.04 | 0.32 | 0.26 | 0.37 | 0.42 | 0.40 | -0.03 | -0.08 |
| TCGA.77.A5G1.01 | -0.220 | -0.43 | -0.19 | -0.26 | -0.24 | -0.20 | -0.20 | -0.36 | -0.28 | -0.34 | -0.39 | -0.34 | -0.19 | -0.15 | -0.22 | -0.37 | -0.37 | -0.33 | 0.01 | -0.29 | -0.08 | -0.27 | -0.15 | -0.28 | -0.12 | -0.12 | -0.21 | 0.16 | 0.34 |
| TCGA.77.A5G3.01 | 0.177 | -0.07 | 0.17 | -0.11 | -0.14 | 0.01 | 0.04 | -0.01 | 0.24 | -0.19 | 0.02 | -0.07 | 0.17 | -0.19 | -0.18 | -0.17 | -0.24 | -0.18 | -0.30 | -0.25 | 0.10 | -0.13 | -0.24 | -0.09 | 0.19 | 0.17 | 0.00 | -0.30 | -0.25 |
| TCGA.77.A5G6.01 | 0.301 | -0.15 | 0.11 | -0.06 | -0.15 | 0.03 | -0.01 | 0.00 | -0.14 | 0.06 | -0.12 | -0.05 | 0.11 | -0.08 | 0.01 | -0.03 | 0.02 | -0.18 | -0.07 | -0.22 | 0.14 | -0.13 | -0.03 | -0.19 | -0.07 | -0.04 | 0.06 | -0.16 | -0.05 |
| TCGA.77.A5G7.01 | -0.396 | -0.12 | 0.14 | -0.04 | -0.03 | -0.03 | -0.17 | -0.07 | -0.28 | -0.16 | 0.08 | 0.01 | 0.14 | -0.04 | 0.01 | 0.15 | 0.10 | 0.01 | 0.04 | 0.09 | -0.13 | -0.12 | -0.14 | -0.12 | -0.19 | -0.18 | 0.05 | -0.02 | -0.08 |
| TCGA.77.A5GA.01 | 0.169 | 0.14 | 0.26 | 0.20 | 0.10 | 0.29 | 0.24 | 0.21 | 0.11 | 0.25 | 0.21 | 0.07 | 0.26 | 0.03 | 0.26 | 0.31 | 0.33 | 0.20 | -0.06 | -0.02 | 0.34 | -0.09 | 0.26 | 0.11 | 0.30 | 0.30 | 0.33 | -0.19 | -0.18 |
| TCGA.77.A5GB.01 | 0.226 | -0.36 | 0.09 | -0.16 | -0.19 | -0.11 | -0.12 | -0.19 | -0.08 | -0.27 | -0.16 | -0.21 | 0.09 | -0.21 | -0.17 | -0.05 | 0.05 | -0.06 | -0.08 | -0.05 | -0.07 | 0.08 | -0.20 | -0.15 | -0.21 | -0.26 | -0.21 | -0.02 | -0.10 |
| TCGA.77.A5GF.01 | 0.239 | 0.05 | -0.24 | 0.10 | 0.30 | -0.02 | -0.03 | 0.12 | -0.09 | -0.16 | 0.06 | 0.25 | -0.24 | 0.34 | 0.09 | -0.19 | -0.15 | 0.16 | 0.21 | 0.18 | -0.12 | 0.07 | 0.05 | 0.22 | 0.01 | 0.06 | 0.06 | 0.31 | 0.19 |
| TCGA.77.A5GH.01 | 0.118 | 0.08 | -0.19 | 0.03 | -0.12 | -0.02 | 0.13 | 0.26 | 0.23 | 0.10 | 0.17 | 0.20 | -0.19 | -0.06 | 0.09 | -0.08 | -0.09 | -0.10 | -0.11 | -0.02 | 0.21 | -0.30 | 0.00 | -0.23 | 0.20 | 0.03 | 0.05 | -0.20 | -0.30 |
| TCGA.78.7143.01 | -0.101 | -0.24 | -0.32 | -0.28 | -0.01 | -0.27 | -0.30 | -0.08 | 0.13 | 0.27 | -0.16 | -0.08 | -0.32 | -0.09 | -0.18 | -0.35 | -0.41 | -0.36 | -0.11 | -0.11 | -0.34 | 0.15 | -0.11 | -0.07 | -0.27 | -0.34 | -0.34 | 0.23 | 0.24 |
| TCGA.78.7145.01 | 0.308 | 0.22 | -0.14 | 0.01 | 0.06 | -0.14 | -0.05 | 0.19 | 0.14 | 0.18 | 0.31 | 0.18 | -0.14 | 0.05 | 0.28 | -0.22 | -0.24 | -0.12 | 0.02 | 0.21 | -0.03 | 0.13 | 0.11 | 0.07 | -0.08 | 0.14 | 0.09 | 0.25 | -0.05 |
| TCGA.78.7146.01 | 0.257 | 0.25 | -0.15 | 0.02 | 0.08 | -0.13 | -0.07 | -0.19 | 0.04 | -0.17 | -0.11 | -0.10 | -0.15 | -0.22 | -0.11 | -0.14 | -0.05 | 0.12 | 0.16 | 0.11 | -0.18 | 0.08 | -0.23 | 0.07 | -0.28 | -0.20 | -0.01 | 0.12 | 0.05 |
| TCGA.78.7147.01 | 0.493 | 0.51 | -0.07 | 0.06 | 0.23 | -0.02 | 0.05 | 0.13 | 0.32 | 0.11 | 0.21 | 0.11 | -0.07 | 0.12 | 0.12 | -0.12 | -0.12 | 0.14 | 0.13 | 0.25 | -0.11 | 0.11 | -0.03 | 0.06 | -0.03 | -0.01 | 0.00 | 0.09 | 0.05 |
| TCGA.78.7148.01 | -0.545 | 0.21 | 0.27 | 0.28 | 0.16 | 0.32 | 0.32 | 0.13 | 0.12 | 0.30 | 0.26 | 0.24 | 0.27 | 0.06 | 0.28 | 0.39 | 0.02 | 0.27 | -0.04 | 0.12 | 0.39 | 0.24 | 0.26 | 0.21 | 0.37 | 0.41 | 0.17 | 0.12 | -0.32 |
| TCGA.78.7149.01 | 0.051 | -0.22 | 0.19 | -0.02 | -0.01 | -0.09 | -0.01 | -0.04 | -0.25 | -0.37 | 0.01 | -0.06 | 0.19 | -0.11 | 0.17 | 0.08 | 0.04 | 0.25 | 0.25 | 0.00 | -0.08 | -0.13 | -0.10 | 0.10 | -0.05 | 0.04 | 0.05 | -0.08 | 0.07 |
| TCGA.78.7150.01 | -0.364 | -0.34 | -0.19 | -0.15 | -0.27 | -0.14 | -0.14 | -0.31 | -0.03 | -0.28 | -0.34 | -0.33 | -0.19 | -0.06 | -0.13 | -0.27 | -0.18 | -0.26 | 0.12 | -0.25 | -0.18 | -0.14 | -0.17 | -0.25 | -0.23 | -0.28 | -0.14 | 0.02 | 0.19 |
| TCGA.78.7152.01 | -0.126 | -0.53 | -0.18 | -0.32 | -0.40 | -0.31 | -0.37 | -0.31 | -0.37 | -0.51 | -0.40 | -0.37 | -0.18 | -0.28 | -0.33 | -0.23 | -0.08 | -0.25 | -0.35 | -0.35 | -0.34 | -0.39 | -0.47 | -0.34 | -0.35 | -0.42 | -0.30 | -0.38 | -0.10 |
| TCGA.78.7153.01 | 0.375 | -0.49 | 0.05 | -0.15 | -0.23 | -0.21 | -0.15 | -0.04 | -0.21 | -0.11 | 0.11 | -0.03 | 0.05 | -0.28 | -0.12 | -0.43 | -0.35 | -0.41 | -0.25 | -0.09 | -0.10 | -0.18 | -0.32 | -0.16 | -0.14 | -0.02 | -0.25 | -0.08 | -0.01 |
| TCGA.78.7156.01 | -0.432 | 0.15 | 0.31 | 0.18 | 0.03 | 0.21 | 0.24 | 0.25 | -0.04 | 0.25 | 0.16 | 0.19 | 0.31 | 0.12 | 0.09 | 0.35 | 0.35 | 0.27 | -0.15 | 0.03 | 0.33 | -0.18 | 0.20 | 0.15 | 0.29 | 0.16 | 0.25 | -0.04 | -0.43 |
| TCGA.78.7158.01 | -0.039 | 0.02 | -0.28 | -0.04 | -0.22 | -0.11 | 0.00 | 0.15 | 0.20 | -0.28 | 0.08 | 0.03 | -0.28 | -0.15 | -0.18 | -0.13 | 0.00 | -0.01 | -0.17 | -0.19 | 0.08 | -0.10 | -0.02 | -0.03 | -0.06 | -0.03 | 0.00 | -0.20 | -0.10 |
| TCGA.78.7159.01 | 0.211 | -0.01 | -0.11 | -0.13 | -0.16 | 0.00 | -0.12 | -0.01 | 0.14 | 0.16 | -0.07 | -0.07 | -0.11 | -0.21 | -0.14 | -0.24 | -0.01 | -0.07 | -0.04 | -0.03 | -0.04 | -0.09 | -0.04 | -0.15 | -0.01 | 0.15 | -0.04 | 0.01 | 0.02 |
| TCGA.78.7160.01 | -0.103 | -0.21 | -0.21 | 0.03 | 0.18 | -0.15 | -0.18 | 0.20 | 0.30 | 0.03 | -0.06 | 0.21 | -0.21 | 0.14 | -0.17 | -0.29 | -0.16 | -0.03 | -0.10 | -0.05 | -0.18 | 0.08 | -0.04 | 0.05 | -0.15 | -0.11 | -0.18 | 0.07 | 0.31 |
| TCGA.78.7161.01 | -0.338 | -0.22 | 0.38 | 0.04 | -0.18 | -0.07 | -0.07 | -0.06 | -0.27 | -0.29 | -0.20 | -0.13 | 0.38 | 0.00 | -0.18 | 0.23 | 0.23 | 0.05 | -0.10 | -0.14 | 0.21 | -0.02 | -0.02 | -0.21 | 0.07 | 0.04 | -0.02 | -0.29 | 0.10 |
| TCGA.78.7162.01 | 0.172 | 0.15 | -0.04 | 0.00 | 0.30 | 0.01 | -0.07 | 0.12 | 0.24 | 0.13 | 0.18 | 0.23 | -0.04 | 0.18 | 0.03 | -0.27 | -0.31 | -0.13 | 0.24 | 0.10 | -0.20 | 0.07 | 0.04 | 0.03 | -0.08 | -0.18 | -0.02 | 0.28 | 0.23 |
| TCGA.78.7163.01 | 0.569 | -0.51 | -0.20 | -0.20 | -0.04 | -0.23 | -0.21 | -0.12 | -0.10 | -0.16 | -0.27 | -0.21 | -0.20 | -0.05 | -0.35 | -0.32 | -0.14 | -0.07 | -0.09 | -0.08 | -0.25 | 0.06 | -0.31 | 0.15 | -0.19 | -0.18 | -0.27 | 0.15 | 0.36 |
| TCGA.78.7166.01 | 0.535 | -0.11 | 0.27 | 0.00 | -0.04 | 0.06 | 0.02 | 0.01 | 0.07 | 0.00 | 0.12 | -0.01 | 0.27 | -0.04 | 0.11 | 0.25 | 0.34 | 0.18 | 0.06 | 0.13 | 0.00 | 0.03 | 0.08 | 0.15 | -0.02 | 0.14 | 0.11 | 0.06 | 0.13 |
| TCGA.78.7167.01 | 0.526 | 0.51 | 0.03 | 0.17 | 0.24 | 0.09 | 0.07 | 0.10 | 0.16 | 0.03 | 0.10 | 0.19 | 0.03 | 0.21 | 0.27 | 0.16 | 0.20 | 0.10 | 0.22 | 0.08 | 0.08 | 0.11 | 0.14 | 0.37 | 0.02 | 0.09 | 0.10 | 0.17 | 0.07 |
| TCGA.78.7220.01 | 0.197 | -0.10 | -0.22 | -0.11 | 0.16 | -0.20 | -0.04 | 0.09 | 0.10 | 0.12 | 0.15 | 0.10 | -0.22 | -0.03 | 0.04 | -0.38 | -0.33 | -0.29 | -0.07 | -0.05 | -0.07 | -0.06 | -0.03 | -0.04 | -0.13 | -0.09 | -0.04 | 0.08 | 0.28 |
| TCGA.78.7535.01 | 0.055 | 0.06 | 0.14 | -0.01 | 0.15 | 0.01 | -0.03 | 0.14 | 0.19 | 0.13 | -0.03 | 0.18 | 0.14 | -0.20 | -0.11 | -0.15 | -0.26 | -0.24 | 0.06 | 0.00 | 0.06 | -0.09 | -0.10 | -0.18 | -0.04 | -0.13 | -0.02 | 0.06 | 0.03 |
| TCGA.78.7536.01 | 0.069 | 0.17 | -0.39 | -0.18 | -0.08 | -0.16 | -0.14 | -0.17 | 0.10 | -0.17 | -0.01 | -0.13 | -0.39 | -0.27 | -0.05 | -0.34 | -0.16 | 0.03 | -0.02 | 0.08 | -0.30 | 0.10 | -0.26 | 0.06 | -0.13 | 0.06 | -0.18 | 0.31 | 0.32 |
| TCGA.78.7537.01 | -0.399 | -0.17 | 0.27 | -0.29 | -0.21 | -0.25 | -0.25 | -0.34 | -0.44 | -0.20 | 0.01 | -0.28 | 0.27 | -0.29 | -0.18 | -0.20 | -0.27 | -0.28 | -0.16 | -0.17 | -0.18 | -0.06 | -0.22 | -0.30 | -0.28 | -0.21 | -0.17 | -0.29 | 0.03 |
| TCGA.78.7539.01 | -0.229 | 0.12 | 0.35 | 0.28 | 0.10 | 0.27 | 0.30 | 0.19 | 0.10 | 0.23 | 0.15 | 0.19 | 0.35 | 0.01 | 0.25 | 0.40 | 0.27 | 0.26 | 0.10 | 0.28 | 0.31 | 0.21 | 0.29 | 0.24 | 0.33 | 0.37 | 0.30 | 0.14 | -0.17 |
| TCGA.78.7540.01 | 0.499 | 0.16 | 0.19 | -0.03 | 0.18 | 0.08 | 0.10 | -0.09 | 0.01 | 0.00 | 0.13 | -0.02 | 0.19 | 0.14 | 0.04 | -0.13 | -0.11 | 0.02 | -0.23 | -0.05 | -0.02 | 0.33 | 0.15 | 0.17 | 0.02 | 0.01 | -0.11 | 0.13 | 0.02 |
| TCGA.78.8640.01 | -0.327 | 0.05 | 0.12 | 0.27 | -0.01 | 0.29 | 0.36 | 0.27 | 0.18 | 0.36 | 0.19 | 0.25 | 0.12 | 0.15 | 0.32 | 0.14 | 0.02 | 0.01 | -0.09 | 0.14 | 0.36 | 0.21 | 0.30 | 0.20 | 0.35 | 0.34 | 0.10 | 0.14 | -0.24 |
| TCGA.78.8648.01 | -0.013 | -0.36 | 0.00 | 0.09 | -0.32 | 0.10 | 0.05 | -0.19 | -0.29 | -0.25 | -0.18 | -0.24 | 0.00 | -0.12 | -0.05 | -0.04 | 0.13 | 0.02 | 0.00 | -0.23 | 0.07 | -0.27 | -0.11 | -0.09 | -0.01 | -0.02 | -0.07 | -0.19 | 0.13 |
| TCGA.78.8655.01 | 0.349 | -0.22 | -0.37 | -0.28 | -0.02 | -0.21 | -0.25 | -0.31 | -0.01 | -0.18 | -0.16 | -0.11 | -0.37 | -0.06 | -0.32 | -0.26 | -0.41 | -0.23 | -0.01 | -0.07 | -0.36 | 0.18 | -0.26 | -0.08 | -0.21 | -0.34 | -0.30 | 0.20 | 0.26 |
| TCGA.78.8660.01 | 0.130 | 0.19 | -0.20 | -0.07 | 0.08 | -0.13 | -0.17 | -0.02 | 0.08 | 0.19 | -0.19 | 0.06 | -0.20 | -0.03 | -0.12 | -0.26 | -0.33 | -0.02 | 0.04 | 0.10 | -0.30 | 0.12 | -0.11 | 0.00 | -0.17 | -0.22 | -0.16 | 0.16 | 0.18 |
| TCGA.78.8662.01 | -0.409 | -0.07 | -0.06 | 0.06 | 0.02 | 0.03 | -0.04 | -0.03 | 0.05 | -0.16 | -0.30 | -0.06 | -0.06 | 0.19 | -0.04 | -0.18 | -0.25 | -0.30 | -0.21 | -0.26 | 0.13 | -0.07 | -0.05 | -0.07 | -0.04 | 0.01 | -0.11 | -0.12 | 0.08 |
| TCGA.79.5596.01 | 0.234 | 0.45 | -0.07 | 0.21 | -0.06 | 0.22 | 0.28 | 0.24 | 0.27 | 0.09 | 0.33 | 0.31 | -0.07 | 0.11 | 0.19 | 0.30 | 0.30 | 0.35 | 0.24 | 0.09 | 0.27 | -0.19 | 0.15 | 0.19 | 0.17 | 0.25 | 0.37 | -0.05 | -0.29 |
| TCGA.80.5607.01 | 0.252 | 0.13 | -0.08 | 0.07 | 0.37 | 0.11 | 0.08 | 0.23 | 0.15 | 0.24 | 0.19 | 0.18 | -0.08 | 0.21 | 0.04 | -0.11 | -0.09 | 0.04 | 0.22 | 0.14 | 0.06 | 0.22 | 0.15 | 0.26 | 0.13 | -0.01 | 0.05 | 0.34 | 0.26 |
| TCGA.80.5608.01 | -0.279 | -0.48 | 0.25 | -0.25 | -0.43 | -0.28 | -0.25 | -0.26 | -0.20 | -0.43 | -0.26 | -0.24 | 0.25 | -0.33 | -0.31 | -0.29 | -0.20 | -0.15 | -0.18 | -0.31 | -0.14 | -0.40 | -0.41 | -0.28 | -0.19 | -0.28 | -0.17 | -0.33 | -0.14 |
| TCGA.80.5611.01 | 0.230 | -0.13 | -0.15 | -0.26 | -0.08 | -0.23 | -0.24 | 0.10 | 0.28 | 0.12 | -0.01 | 0.08 | -0.15 | -0.11 | -0.07 | -0.36 | -0.47 | -0.38 | -0.24 | -0.03 | -0.26 | -0.06 | -0.26 | -0.08 | -0.24 | -0.12 | -0.23 | 0.11 | 0.12 |
| TCGA.83.5908.01 | -0.195 | 0.36 | 0.04 | 0.04 | 0.17 | 0.08 | 0.08 | -0.13 | 0.09 | -0.02 | -0.13 | -0.08 | 0.04 | 0.12 | 0.11 | 0.33 | 0.34 | 0.32 | -0.05 | 0.14 | -0.09 | 0.15 | 0.09 | 0.15 | -0.12 | -0.02 | 0.15 | 0.00 | 0.00 |
| TCGA.85.6175.01 | 0.318 | 0.56 | 0.13 | 0.30 | 0.31 | 0.31 | 0.31 | 0.36 | 0.46 | 0.54 | 0.37 | 0.42 | 0.13 | 0.25 | 0.26 | 0.40 | 0.27 | 0.28 | 0.23 | 0.31 | 0.17 | 0.33 | 0.32 | 0.24 | 0.23 | 0.28 | 0.37 | 0.17 | 0.25 |
| TCGA.85.6560.01 | 0.087 | -0.37 | -0.16 | -0.15 | 0.02 | -0.10 | -0.09 | -0.11 | -0.35 | -0.43 | -0.07 | 0.00 | -0.16 | 0.17 | 0.12 | 0.11 | -0.02 | 0.13 | 0.22 | 0.01 | -0.17 | -0.18 | -0.16 | 0.15 | -0.10 | -0.13 | 0.00 | -0.13 | 0.19 |
| TCGA.85.6561.01 | 0.086 | -0.17 | -0.27 | -0.02 | 0.23 | -0.10 | -0.09 | -0.06 | -0.13 | -0.08 | 0.07 | 0.09 | -0.27 | 0.17 | -0.15 | -0.09 | -0.28 | -0.07 | 0.17 | -0.13 | -0.23 | -0.14 | -0.05 | -0.05 | -0.08 | -0.26 | -0.08 | 0.13 | 0.08 |
| TCGA.85.6798.01 | 0.469 | 0.26 | -0.22 | 0.18 | 0.14 | 0.03 | 0.11 | 0.22 | 0.16 | 0.32 | 0.22 | 0.25 | -0.22 | 0.25 | 0.06 | -0.12 | -0.29 | -0.03 | 0.27 | 0.05 | -0.03 | 0.04 | 0.23 | 0.19 | 0.00 | 0.05 | 0.19 | 0.19 | 0.30 |
| TCGA.85.7696.01 | 0.066 | -0.13 | -0.06 | 0.01 | -0.05 | -0.03 | -0.03 | 0.12 | -0.24 | 0.18 | 0.05 | 0.05 | -0.06 | -0.24 | -0.15 | -0.15 | 0.08 | -0.21 | -0.18 | -0.10 | 0.13 | -0.20 | -0.15 | -0.02 | -0.07 | -0.07 | 0.03 | -0.19 | -0.20 |
| TCGA.85.7697.01 | -0.394 | 0.26 | -0.05 | 0.23 | 0.10 | 0.24 | 0.21 | 0.38 | 0.26 | 0.31 | 0.35 | 0.32 | -0.05 | 0.16 | 0.20 | 0.41 | 0.35 | 0.32 | 0.07 | 0.19 | 0.27 | 0.16 | 0.25 | -0.02 | 0.19 | 0.24 | 0.35 | -0.07 | -0.18 |
| TCGA.85.7698.01 | 0.462 | 0.53 | -0.07 | 0.08 | 0.31 | 0.21 | 0.17 | 0.22 | 0.39 | 0.41 | 0.24 | 0.22 | -0.07 | 0.24 | 0.30 | 0.27 | 0.23 | 0.12 | 0.17 | 0.32 | -0.03 | 0.33 | 0.25 | 0.18 | -0.06 | 0.10 | 0.12 | 0.24 | 0.28 |
| TCGA.85.7699.01 | 0.596 | 0.17 | -0.10 | -0.03 | -0.01 | 0.02 | -0.05 | -0.16 | 0.26 | 0.14 | -0.10 | -0.20 | -0.10 | 0.05 | 0.09 | -0.16 | -0.19 | -0.11 | 0.23 | 0.18 | -0.12 | 0.15 | 0.12 | 0.25 | 0.04 | 0.24 | -0.01 | 0.34 | 0.41 |
| TCGA.85.7710.01 | 0.080 | 0.34 | -0.36 | -0.07 | 0.19 | -0.02 | -0.01 | -0.10 | 0.21 | 0.01 | 0.21 | 0.06 | -0.36 | -0.08 | 0.03 | -0.01 | 0.05 | 0.18 | 0.16 | 0.15 | -0.34 | 0.09 | -0.06 | -0.04 | -0.25 | -0.23 | -0.01 | 0.16 | 0.19 |
| TCGA.85.7844.01 | 0.466 | 0.44 | 0.21 | 0.07 | 0.33 | 0.14 | 0.25 | 0.12 | 0.33 | 0.34 | 0.30 | 0.22 | 0.21 | 0.17 | 0.24 | 0.13 | -0.18 | 0.12 | 0.33 | 0.22 | -0.04 | 0.26 | 0.32 | 0.17 | -0.01 | 0.19 | 0.27 | 0.22 | 0.15 |
| TCGA.85.7950.01 | -0.316 | 0.00 | 0.24 | -0.01 | 0.10 | -0.04 | -0.15 | -0.09 | -0.07 | -0.26 | -0.35 | -0.18 | 0.24 | 0.08 | -0.20 | -0.02 | 0.13 | -0.04 | -0.06 | -0.11 | -0.05 | 0.02 | -0.20 | -0.15 | -0.18 | -0.16 | -0.08 | -0.18 | -0.02 |
| TCGA.85.8048.01 | -0.126 | 0.30 | 0.29 | 0.18 | 0.10 | 0.19 | 0.16 | 0.17 | 0.19 | 0.22 | 0.05 | -0.03 | 0.29 | 0.05 | 0.15 | 0.43 | 0.46 | 0.43 | 0.06 | 0.08 | 0.22 | -0.06 | 0.22 | -0.09 | 0.23 | 0.22 | 0.27 | -0.15 | -0.29 |
| TCGA.85.8049.01 | -0.132 | 0.02 | 0.17 | -0.03 | -0.08 | -0.06 | -0.03 | -0.02 | -0.04 | 0.12 | -0.20 | -0.05 | 0.17 | -0.05 | -0.05 | 0.02 | 0.15 | 0.25 | 0.03 | 0.04 | -0.05 | 0.25 | -0.02 | 0.09 | -0.09 | 0.13 | -0.08 | -0.04 | 0.12 |
| TCGA.85.8052.01 | 0.468 | 0.31 | -0.32 | -0.07 | -0.21 | -0.23 | -0.25 | -0.11 | -0.16 | -0.48 | -0.06 | -0.06 | -0.32 | 0.12 | -0.19 | -0.24 | -0.11 | 0.15 | 0.29 | 0.10 | -0.28 | -0.13 | -0.22 | 0.05 | -0.30 | 0.00 | -0.07 | 0.19 | 0.26 |
| TCGA.85.8070.01 | -0.183 | -0.11 | 0.51 | 0.29 | -0.03 | 0.28 | 0.25 | -0.11 | -0.02 | 0.12 | -0.01 | -0.20 | 0.51 | 0.18 | 0.24 | 0.33 | 0.32 | 0.20 | -0.08 | 0.06 | 0.40 | 0.15 | 0.32 | 0.04 | 0.28 | 0.26 | 0.11 | 0.00 | -0.08 |
| TCGA.85.8071.01 | 0.177 | 0.34 | 0.31 | 0.28 | 0.21 | 0.30 | 0.35 | 0.33 | 0.19 | 0.25 | 0.26 | 0.26 | 0.31 | 0.10 | 0.30 | 0.33 | 0.35 | 0.25 | 0.35 | 0.22 | 0.42 | -0.16 | 0.30 | 0.23 | 0.33 | 0.38 | 0.38 | 0.09 | -0.24 |
| TCGA.85.8072.01 | -0.099 | -0.46 | 0.32 | -0.04 | 0.00 | -0.09 | -0.07 | 0.01 | 0.14 | 0.08 | -0.28 | 0.07 | 0.32 | 0.00 | 0.00 | -0.24 | -0.02 | -0.14 | -0.03 | -0.12 | -0.06 | -0.03 | 0.05 | -0.08 | -0.07 | -0.10 | 0.03 | 0.07 | 0.18 |
| TCGA.85.8276.01 | 0.253 | 0.17 | 0.01 | 0.04 | -0.10 | -0.01 | -0.07 | -0.19 | 0.10 | -0.11 | -0.27 | -0.12 | 0.01 | -0.12 | -0.09 | -0.08 | -0.08 | -0.18 | -0.03 | -0.01 | -0.05 | 0.16 | -0.15 | 0.08 | -0.15 | -0.13 | -0.10 | 0.13 | 0.15 |
| TCGA.85.8277.01 | 0.462 | -0.32 | 0.10 | -0.20 | 0.10 | -0.25 | -0.20 | -0.07 | 0.08 | -0.07 | -0.25 | -0.07 | 0.10 | -0.15 | 0.00 | -0.20 | -0.03 | -0.11 | 0.15 | -0.09 | -0.17 | 0.00 | -0.12 | 0.02 | -0.25 | -0.09 | -0.09 | 0.16 | 0.12 |
| TCGA.85.8287.01 | 0.304 | 0.25 | 0.16 | 0.00 | 0.20 | -0.06 | -0.05 | 0.12 | 0.22 | -0.11 | 0.05 | 0.18 | 0.16 | 0.25 | 0.06 | -0.27 | -0.32 | -0.17 | 0.17 | 0.03 | -0.22 | 0.14 | 0.02 | 0.21 | -0.14 | -0.07 | -0.09 | 0.27 | 0.33 |
| TCGA.85.8288.01 | -0.062 | 0.26 | -0.27 | 0.20 | -0.01 | 0.14 | 0.18 | -0.14 | 0.07 | 0.31 | 0.11 | -0.06 | -0.27 | 0.19 | 0.03 | 0.34 | 0.47 | 0.43 | 0.14 | 0.08 | 0.04 | 0.18 | 0.19 | 0.04 | 0.09 | 0.12 | 0.23 | 0.03 | -0.20 |
| TCGA.85.8350.01 | 0.119 | -0.17 | -0.18 | -0.26 | -0.06 | -0.22 | -0.09 | 0.17 | 0.19 | 0.21 | 0.19 | 0.18 | -0.18 | -0.13 | -0.17 | -0.37 | -0.46 | -0.44 | -0.20 | -0.13 | -0.26 | 0.17 | -0.23 | 0.09 | -0.17 | -0.15 | -0.11 | 0.04 | 0.24 |
| TCGA.85.8351.01 | 0.432 | -0.42 | 0.02 | -0.24 | -0.09 | -0.29 | -0.22 | -0.14 | -0.26 | -0.21 | -0.25 | -0.16 | 0.02 | -0.08 | -0.15 | -0.25 | -0.15 | -0.25 | -0.04 | -0.20 | -0.35 | -0.20 | -0.23 | -0.05 | -0.36 | -0.26 | -0.22 | 0.08 | 0.17 |
| TCGA.85.8352.01 | -0.278 | 0.39 | -0.41 | 0.02 | -0.04 | 0.08 | 0.14 | 0.34 | 0.01 | 0.44 | 0.46 | 0.28 | -0.41 | 0.00 | 0.12 | -0.04 | -0.29 | -0.24 | 0.08 | 0.16 | 0.10 | -0.02 | 0.04 | 0.04 | 0.18 | 0.18 | 0.23 | -0.03 | 0.07 |
| TCGA.85.8353.01 | -0.380 | -0.22 | -0.46 | -0.15 | 0.03 | -0.22 | -0.25 | -0.07 | -0.03 | -0.12 | -0.15 | -0.01 | -0.46 | 0.16 | -0.15 | -0.24 | -0.36 | -0.17 | 0.00 | -0.02 | -0.33 | -0.01 | -0.24 | -0.03 | -0.20 | -0.30 | -0.26 | -0.03 | 0.09 |
| TCGA.85.8354.01 | -0.089 | 0.04 | -0.08 | -0.12 | -0.02 | -0.21 | -0.23 | -0.13 | 0.20 | -0.15 | -0.18 | -0.11 | -0.08 | -0.05 | -0.12 | -0.34 | -0.36 | -0.25 | 0.19 | -0.15 | -0.19 | 0.06 | -0.16 | -0.20 | -0.27 | -0.29 | -0.19 | 0.12 | 0.10 |
| TCGA.85.8355.01 | 0.342 | -0.55 | -0.31 | -0.24 | -0.25 | -0.26 | -0.22 | -0.28 | -0.09 | -0.36 | -0.05 | -0.17 | -0.31 | -0.11 | -0.10 | -0.42 | -0.42 | -0.40 | -0.15 | -0.11 | -0.22 | -0.23 | -0.29 | -0.12 | -0.09 | -0.14 | -0.25 | 0.00 | 0.13 |
| TCGA.85.8481.01 | -0.022 | -0.11 | -0.06 | 0.15 | -0.02 | 0.23 | 0.20 | 0.04 | 0.01 | -0.05 | 0.30 | 0.22 | -0.06 | -0.11 | -0.13 | 0.16 | 0.27 | 0.12 | -0.01 | -0.03 | 0.12 | -0.01 | 0.08 | 0.20 | 0.11 | -0.04 | 0.14 | -0.23 | -0.32 |
| TCGA.85.8580.01 | -0.065 | -0.47 | -0.05 | -0.14 | -0.14 | -0.15 | -0.19 | -0.07 | 0.02 | -0.19 | -0.28 | -0.10 | -0.05 | -0.23 | -0.32 | -0.26 | -0.26 | -0.27 | -0.27 | -0.17 | -0.15 | -0.11 | -0.19 | -0.18 | -0.12 | -0.24 | -0.27 | -0.09 | 0.15 |
| TCGA.85.8582.01 | 0.065 | 0.39 | -0.37 | 0.10 | 0.19 | -0.01 | 0.04 | 0.26 | 0.36 | -0.14 | 0.30 | 0.27 | -0.37 | 0.24 | 0.07 | 0.11 | 0.09 | 0.26 | 0.14 | 0.12 | -0.11 | -0.06 | 0.12 | 0.20 | 0.02 | 0.10 | 0.12 | 0.03 | 0.17 |
| TCGA.85.8664.01 | 0.445 | 0.05 | -0.42 | -0.02 | 0.15 | -0.16 | -0.14 | 0.16 | 0.07 | 0.09 | -0.09 | 0.12 | -0.42 | 0.07 | -0.09 | -0.19 | -0.08 | 0.06 | 0.06 | -0.05 | -0.23 | -0.14 | -0.12 | -0.11 | -0.12 | -0.26 | -0.09 | 0.16 | 0.09 |
| TCGA.85.8666.01 | 0.507 | -0.29 | -0.40 | -0.24 | -0.16 | -0.29 | -0.26 | -0.15 | -0.07 | -0.22 | -0.27 | -0.27 | -0.40 | 0.07 | -0.15 | -0.40 | -0.05 | 0.00 | -0.24 | -0.11 | -0.31 | 0.05 | -0.09 | 0.06 | -0.22 | -0.08 | -0.34 | 0.15 | 0.21 |
| TCGA.85.A4CL.01 | -0.217 | -0.42 | 0.01 | -0.25 | -0.24 | -0.22 | -0.16 | -0.34 | -0.36 | -0.44 | -0.48 | -0.48 | 0.01 | -0.16 | -0.38 | -0.19 | -0.11 | -0.05 | -0.07 | -0.31 | -0.20 | -0.07 | -0.20 | -0.27 | -0.22 | -0.44 | -0.31 | -0.13 | 0.25 |
| TCGA.85.A4CN.01 | -0.315 | -0.27 | -0.27 | -0.22 | 0.03 | -0.14 | -0.25 | -0.28 | -0.19 | -0.31 | -0.42 | -0.28 | -0.27 | 0.05 | -0.38 | -0.34 | -0.25 | -0.15 | -0.07 | -0.27 | -0.32 | -0.05 | -0.18 | -0.32 | -0.34 | -0.44 | -0.28 | -0.27 | 0.14 |
| TCGA.85.A4JB.01 | -0.204 | 0.01 | -0.42 | -0.04 | 0.16 | -0.09 | -0.14 | 0.26 | 0.19 | 0.00 | 0.28 | 0.29 | -0.42 | 0.06 | -0.26 | -0.02 | -0.03 | 0.06 | 0.04 | -0.13 | -0.13 | -0.12 | 0.00 | -0.17 | -0.11 | -0.25 | -0.05 | -0.09 | 0.15 |
| TCGA.85.A4JC.01 | 0.277 | -0.13 | -0.08 | 0.02 | -0.05 | -0.06 | 0.03 | -0.27 | 0.11 | -0.32 | -0.22 | -0.29 | -0.08 | 0.19 | -0.03 | 0.19 | 0.10 | 0.08 | 0.12 | -0.05 | -0.05 | -0.20 | 0.02 | 0.00 | -0.05 | 0.10 | -0.02 | 0.06 | 0.14 |
| TCGA.85.A4PA.01 | -0.472 | -0.41 | 0.23 | 0.06 | -0.11 | 0.06 | -0.07 | -0.18 | -0.18 | 0.01 | -0.26 | -0.39 | 0.23 | 0.11 | 0.10 | 0.23 | 0.48 | 0.22 | -0.14 | 0.03 | 0.12 | 0.20 | 0.07 | -0.03 | 0.03 | 0.00 | -0.11 | -0.17 | -0.13 |
| TCGA.85.A4QQ.01 | -0.111 | 0.15 | 0.01 | -0.03 | 0.01 | 0.10 | 0.08 | 0.14 | 0.21 | 0.45 | 0.21 | -0.01 | 0.01 | -0.04 | 0.08 | 0.12 | 0.14 | -0.07 | -0.01 | -0.14 | 0.18 | -0.01 | 0.15 | -0.25 | 0.12 | 0.05 | 0.09 | -0.11 | -0.07 |
| TCGA.85.A4QR.01 | 0.498 | 0.31 | -0.16 | -0.04 | 0.13 | -0.01 | 0.11 | -0.23 | 0.26 | -0.10 | -0.16 | -0.19 | -0.16 | 0.17 | -0.08 | 0.06 | 0.00 | -0.10 | -0.07 | 0.10 | -0.04 | 0.27 | -0.01 | 0.19 | -0.04 | -0.08 | -0.10 | 0.14 | 0.37 |
| TCGA.85.A50M.01 | -0.304 | 0.49 | -0.29 | 0.20 | 0.24 | 0.16 | 0.28 | 0.36 | 0.30 | 0.35 | 0.29 | 0.39 | -0.29 | 0.33 | 0.04 | 0.31 | 0.20 | 0.35 | 0.02 | 0.12 | 0.03 | 0.34 | 0.24 | 0.26 | 0.30 | 0.08 | 0.18 | 0.08 | 0.01 |
| TCGA.85.A50Z.01 | -0.099 | -0.27 | 0.10 | 0.06 | -0.11 | 0.15 | 0.01 | 0.19 | 0.04 | -0.05 | -0.01 | 0.11 | 0.10 | -0.11 | -0.04 | 0.16 | 0.20 | -0.10 | -0.19 | -0.12 | 0.20 | 0.04 | -0.07 | 0.00 | 0.13 | 0.09 | 0.05 | -0.23 | -0.14 |
| TCGA.85.A510.01 | -0.418 | -0.48 | -0.28 | -0.25 | -0.17 | -0.19 | -0.21 | -0.21 | -0.07 | -0.13 | -0.21 | -0.30 | -0.28 | -0.25 | -0.20 | -0.34 | -0.19 | -0.36 | -0.17 | -0.11 | -0.17 | -0.27 | -0.31 | -0.35 | -0.33 | -0.29 | -0.25 | -0.29 | 0.11 |
| TCGA.85.A511.01 | -0.432 | -0.27 | 0.02 | -0.08 | -0.21 | 0.00 | -0.10 | 0.05 | -0.15 | 0.09 | 0.01 | -0.03 | 0.02 | -0.20 | 0.06 | -0.08 | -0.09 | -0.29 | -0.30 | -0.09 | 0.09 | -0.09 | -0.10 | -0.21 | 0.11 | 0.20 | -0.08 | -0.21 | -0.23 |
| TCGA.85.A512.01 | 0.612 | -0.08 | -0.17 | -0.24 | 0.06 | -0.25 | -0.22 | -0.24 | -0.06 | -0.24 | -0.03 | -0.13 | -0.17 | 0.00 | -0.11 | -0.24 | -0.28 | -0.24 | 0.15 | 0.11 | -0.33 | 0.01 | -0.16 | 0.07 | -0.21 | -0.23 | -0.17 | 0.11 | 0.39 |
| TCGA.85.A513.01 | -0.464 | -0.24 | 0.15 | -0.09 | -0.07 | -0.06 | -0.19 | -0.06 | -0.24 | -0.02 | -0.15 | -0.17 | 0.15 | -0.15 | -0.27 | 0.04 | 0.06 | -0.11 | -0.23 | -0.17 | 0.09 | -0.16 | -0.14 | -0.16 | -0.06 | -0.27 | -0.14 | -0.27 | -0.25 |
| TCGA.85.A53L.01 | 0.036 | -0.44 | -0.18 | -0.11 | -0.23 | -0.11 | -0.12 | -0.10 | 0.11 | 0.03 | -0.04 | -0.20 | -0.18 | -0.12 | -0.08 | -0.26 | -0.14 | -0.17 | -0.05 | -0.11 | 0.03 | -0.09 | 0.02 | -0.08 | 0.01 | 0.04 | -0.09 | 0.02 | 0.23 |
| TCGA.85.A5B5.01 | -0.332 | -0.35 | -0.10 | -0.13 | -0.18 | -0.22 | -0.23 | 0.01 | 0.12 | 0.09 | -0.18 | 0.04 | -0.10 | -0.01 | -0.21 | -0.34 | -0.40 | -0.34 | -0.17 | -0.09 | -0.14 | -0.04 | -0.18 | -0.22 | -0.27 | -0.27 | -0.17 | -0.02 | 0.20 |
| TCGA.86.6562.01 | -0.079 | 0.16 | 0.03 | 0.13 | -0.02 | 0.19 | 0.21 | 0.36 | 0.37 | 0.35 | 0.37 | 0.32 | 0.03 | -0.10 | 0.14 | 0.21 | -0.04 | -0.10 | -0.08 | 0.04 | 0.33 | 0.24 | 0.15 | 0.06 | 0.27 | 0.34 | 0.20 | 0.14 | -0.27 |
| TCGA.86.6851.01 | 0.420 | 0.27 | 0.19 | -0.02 | 0.02 | 0.05 | 0.03 | -0.21 | 0.18 | 0.26 | -0.32 | -0.25 | 0.19 | 0.16 | -0.07 | 0.20 | 0.37 | 0.12 | -0.02 | 0.04 | -0.08 | 0.32 | 0.04 | 0.04 | -0.13 | -0.02 | -0.02 | 0.05 | 0.15 |
| TCGA.86.7701.01 | 0.109 | 0.47 | -0.41 | 0.11 | 0.24 | 0.09 | 0.11 | 0.22 | 0.15 | 0.32 | 0.24 | 0.19 | -0.41 | 0.10 | 0.22 | 0.19 | 0.14 | 0.21 | 0.29 | 0.21 | -0.02 | 0.26 | 0.11 | 0.22 | 0.07 | 0.11 | 0.19 | 0.26 | 0.24 |
| TCGA.86.7711.01 | 0.318 | -0.04 | -0.29 | 0.02 | -0.14 | -0.09 | -0.09 | 0.00 | 0.11 | 0.08 | -0.13 | 0.09 | -0.29 | -0.04 | -0.08 | -0.23 | -0.22 | -0.19 | -0.14 | -0.11 | 0.01 | -0.12 | -0.16 | -0.03 | -0.05 | 0.00 | -0.16 | 0.07 | 0.18 |
| TCGA.86.7713.01 | -0.244 | -0.52 | 0.32 | -0.21 | -0.16 | -0.09 | -0.07 | -0.07 | 0.17 | -0.09 | -0.33 | -0.11 | 0.32 | -0.12 | -0.06 | -0.21 | -0.08 | -0.26 | -0.28 | -0.22 | -0.04 | 0.05 | 0.05 | -0.11 | -0.27 | -0.14 | -0.13 | -0.12 | -0.03 |
| TCGA.86.7714.01 | 0.370 | -0.31 | -0.37 | -0.19 | 0.05 | -0.28 | -0.31 | -0.09 | -0.22 | -0.22 | -0.13 | -0.01 | -0.37 | -0.03 | -0.06 | -0.33 | -0.25 | -0.15 | -0.06 | 0.03 | -0.30 | -0.13 | -0.28 | 0.11 | -0.17 | -0.25 | -0.30 | 0.17 | 0.36 |
| TCGA.86.7953.01 | 0.057 | -0.02 | 0.21 | 0.04 | -0.28 | 0.13 | 0.12 | -0.01 | -0.13 | 0.24 | -0.21 | -0.21 | 0.21 | -0.09 | -0.06 | 0.00 | 0.09 | -0.08 | -0.20 | -0.21 | 0.19 | -0.12 | -0.03 | -0.13 | 0.00 | 0.13 | -0.09 | -0.26 | -0.14 |
| TCGA.86.7954.01 | -0.421 | 0.35 | -0.31 | -0.14 | 0.15 | -0.12 | -0.16 | -0.20 | -0.22 | -0.06 | -0.22 | -0.01 | -0.31 | -0.01 | -0.26 | -0.03 | 0.19 | 0.09 | -0.22 | -0.14 | -0.22 | 0.05 | -0.26 | -0.06 | -0.23 | -0.27 | -0.10 | -0.06 | 0.03 |
| TCGA.86.7955.01 | -0.515 | -0.46 | 0.09 | -0.11 | -0.23 | -0.16 | -0.16 | -0.19 | -0.04 | -0.18 | -0.23 | -0.07 | 0.09 | -0.08 | -0.24 | 0.02 | 0.10 | 0.06 | -0.13 | -0.14 | -0.10 | -0.09 | -0.29 | -0.20 | -0.15 | -0.26 | -0.20 | -0.19 | -0.08 |
| TCGA.86.8055.01 | -0.300 | 0.10 | 0.26 | 0.12 | 0.04 | 0.23 | 0.24 | 0.02 | 0.01 | 0.39 | 0.03 | 0.02 | 0.26 | -0.16 | 0.26 | 0.28 | 0.03 | -0.01 | -0.15 | 0.08 | 0.31 | 0.30 | 0.25 | 0.19 | 0.29 | 0.36 | 0.07 | 0.09 | -0.21 |
| TCGA.86.8073.01 | 0.465 | 0.07 | -0.29 | 0.06 | 0.18 | 0.06 | 0.18 | 0.21 | -0.02 | 0.24 | 0.06 | 0.22 | -0.29 | 0.17 | 0.06 | -0.12 | -0.24 | -0.12 | -0.07 | 0.02 | 0.01 | 0.17 | 0.07 | 0.10 | -0.02 | 0.17 | 0.03 | 0.02 | 0.15 |
| TCGA.86.8075.01 | 0.441 | -0.33 | 0.07 | -0.12 | -0.22 | -0.14 | -0.20 | -0.06 | -0.20 | -0.11 | -0.16 | -0.12 | 0.07 | -0.08 | -0.10 | -0.16 | 0.00 | -0.16 | -0.26 | -0.21 | -0.26 | 0.01 | -0.22 | 0.12 | -0.25 | -0.08 | -0.25 | -0.17 | 0.09 |
| TCGA.86.8076.01 | -0.102 | -0.22 | 0.09 | -0.04 | -0.18 | -0.02 | -0.14 | -0.11 | 0.03 | 0.03 | -0.24 | -0.26 | 0.09 | -0.11 | -0.06 | 0.20 | 0.31 | 0.18 | -0.09 | -0.06 | -0.06 | 0.01 | -0.08 | 0.00 | -0.04 | 0.08 | -0.11 | -0.10 | -0.24 |
| TCGA.86.8279.01 | -0.033 | 0.02 | -0.41 | -0.04 | -0.18 | -0.13 | -0.07 | 0.07 | -0.01 | 0.11 | -0.09 | 0.01 | -0.41 | -0.03 | -0.24 | -0.30 | -0.21 | -0.11 | -0.11 | -0.02 | -0.20 | 0.03 | -0.16 | -0.06 | -0.11 | -0.15 | -0.13 | 0.21 | 0.33 |
| TCGA.86.8280.01 | -0.172 | 0.00 | 0.03 | -0.21 | 0.04 | -0.18 | -0.23 | -0.08 | 0.05 | 0.07 | -0.04 | -0.07 | 0.03 | 0.01 | 0.00 | -0.01 | 0.14 | 0.08 | -0.15 | 0.10 | -0.31 | 0.22 | -0.13 | -0.13 | -0.21 | -0.24 | -0.23 | -0.04 | 0.30 |
| TCGA.86.8358.01 | 0.194 | 0.35 | 0.02 | 0.04 | 0.20 | 0.07 | 0.03 | 0.03 | 0.04 | -0.12 | 0.15 | 0.09 | 0.02 | 0.13 | 0.15 | -0.07 | 0.10 | 0.18 | 0.35 | 0.26 | -0.11 | 0.02 | 0.05 | 0.22 | 0.05 | 0.02 | 0.08 | 0.27 | 0.25 |
| TCGA.86.8585.01 | 0.024 | 0.14 | -0.08 | -0.01 | 0.11 | -0.09 | -0.07 | 0.26 | 0.17 | 0.24 | 0.29 | 0.26 | -0.08 | -0.30 | 0.13 | -0.17 | -0.30 | -0.29 | -0.05 | 0.17 | -0.22 | 0.18 | -0.11 | 0.18 | 0.02 | -0.01 | 0.01 | 0.20 | -0.16 |
| TCGA.86.8668.01 | -0.468 | 0.02 | 0.09 | 0.02 | -0.17 | 0.10 | 0.13 | 0.04 | 0.07 | -0.05 | 0.03 | 0.12 | 0.09 | 0.10 | -0.17 | 0.02 | -0.01 | -0.09 | -0.20 | -0.15 | 0.17 | 0.07 | -0.09 | -0.04 | 0.04 | -0.10 | -0.01 | -0.21 | -0.18 |
| TCGA.86.8669.01 | -0.012 | -0.02 | 0.28 | 0.27 | -0.02 | 0.29 | 0.25 | 0.12 | 0.09 | 0.38 | 0.01 | 0.04 | 0.28 | 0.15 | -0.05 | 0.26 | 0.06 | -0.07 | -0.02 | -0.07 | 0.31 | 0.05 | 0.25 | -0.09 | 0.25 | 0.09 | 0.21 | -0.22 | -0.38 |
| TCGA.86.8671.01 | 0.243 | -0.29 | -0.37 | -0.32 | -0.12 | -0.25 | -0.33 | -0.28 | 0.04 | -0.01 | -0.07 | -0.22 | -0.37 | -0.23 | -0.12 | -0.24 | -0.28 | -0.38 | -0.22 | -0.05 | -0.35 | -0.13 | -0.22 | -0.20 | -0.32 | -0.26 | -0.20 | 0.03 | 0.14 |
| TCGA.86.8672.01 | 0.048 | -0.09 | 0.01 | -0.23 | 0.17 | -0.20 | -0.27 | -0.11 | -0.14 | -0.03 | 0.03 | 0.03 | 0.01 | -0.18 | -0.20 | -0.29 | -0.37 | -0.35 | -0.14 | 0.04 | -0.39 | -0.04 | -0.12 | -0.05 | -0.28 | -0.23 | -0.23 | 0.11 | 0.27 |
| TCGA.86.8673.01 | -0.041 | 0.23 | 0.12 | 0.35 | 0.22 | 0.36 | 0.42 | 0.21 | 0.18 | 0.28 | 0.19 | 0.21 | 0.12 | 0.26 | 0.32 | 0.34 | 0.20 | 0.30 | 0.05 | 0.24 | 0.35 | 0.31 | 0.30 | 0.27 | 0.33 | 0.38 | 0.19 | 0.21 | 0.03 |
| TCGA.86.8674.01 | -0.053 | -0.09 | -0.30 | 0.13 | -0.05 | 0.06 | 0.14 | 0.23 | -0.05 | -0.28 | -0.02 | 0.18 | -0.30 | 0.18 | 0.00 | 0.11 | 0.24 | 0.23 | 0.20 | -0.08 | 0.18 | -0.30 | -0.12 | 0.07 | 0.15 | 0.03 | 0.23 | -0.22 | -0.03 |
| TCGA.86.A4D0.01 | -0.078 | 0.11 | 0.30 | 0.20 | -0.06 | 0.17 | 0.09 | 0.13 | -0.03 | 0.31 | -0.14 | -0.03 | 0.30 | -0.10 | -0.08 | 0.26 | 0.26 | 0.31 | -0.24 | -0.08 | 0.29 | -0.07 | 0.13 | -0.06 | 0.24 | 0.01 | 0.20 | -0.15 | -0.29 |
| TCGA.86.A4JF.01 | 0.508 | 0.32 | -0.44 | -0.05 | -0.09 | -0.13 | -0.06 | 0.11 | 0.03 | 0.22 | 0.26 | 0.19 | -0.44 | -0.04 | -0.06 | -0.19 | -0.26 | -0.09 | 0.08 | 0.18 | -0.28 | -0.15 | -0.08 | 0.00 | -0.07 | -0.15 | -0.03 | 0.10 | -0.06 |
| TCGA.86.A4P7.01 | 0.400 | -0.04 | 0.25 | -0.07 | 0.04 | -0.03 | 0.02 | -0.19 | 0.16 | 0.27 | -0.12 | -0.33 | 0.25 | -0.01 | -0.08 | 0.17 | 0.33 | 0.28 | -0.11 | -0.02 | -0.19 | 0.21 | 0.03 | 0.03 | 0.00 | 0.02 | -0.10 | -0.01 | 0.15 |
| TCGA.86.A4P8.01 | -0.478 | -0.02 | 0.32 | 0.23 | 0.12 | 0.25 | 0.27 | 0.03 | 0.13 | 0.18 | 0.00 | 0.03 | 0.32 | 0.16 | 0.23 | 0.30 | 0.33 | 0.17 | -0.15 | 0.13 | 0.32 | 0.28 | 0.32 | 0.00 | 0.31 | 0.28 | 0.05 | 0.01 | -0.18 |
| TCGA.90.6837.01 | 0.268 | 0.57 | -0.11 | 0.19 | 0.25 | 0.26 | 0.23 | 0.21 | 0.38 | 0.14 | 0.29 | 0.24 | -0.11 | 0.33 | 0.23 | 0.30 | 0.36 | 0.36 | 0.26 | 0.19 | 0.04 | 0.25 | 0.31 | 0.21 | 0.06 | 0.23 | 0.33 | 0.10 | 0.14 |
| TCGA.90.7766.01 | -0.314 | 0.06 | 0.01 | 0.00 | -0.04 | -0.10 | -0.03 | -0.05 | 0.15 | -0.08 | -0.18 | -0.07 | 0.01 | 0.23 | -0.13 | -0.01 | -0.01 | 0.00 | -0.06 | -0.11 | 0.09 | -0.14 | -0.07 | -0.04 | -0.03 | -0.16 | -0.07 | -0.04 | 0.03 |
| TCGA.90.7769.01 | -0.439 | -0.28 | 0.27 | -0.21 | -0.13 | -0.23 | -0.16 | -0.04 | -0.14 | -0.29 | -0.26 | -0.06 | 0.27 | -0.21 | -0.20 | -0.25 | -0.25 | -0.13 | 0.08 | -0.18 | -0.04 | -0.20 | -0.20 | -0.16 | -0.14 | -0.20 | -0.20 | -0.19 | -0.16 |
| TCGA.90.7964.01 | 0.407 | 0.22 | -0.01 | 0.11 | 0.22 | 0.12 | 0.03 | -0.27 | -0.06 | 0.02 | -0.19 | -0.15 | -0.01 | -0.01 | 0.19 | 0.06 | 0.09 | 0.01 | -0.14 | 0.11 | -0.08 | 0.33 | 0.17 | 0.15 | 0.14 | 0.13 | -0.08 | 0.11 | 0.15 |
| TCGA.90.A4ED.01 | -0.504 | -0.24 | -0.34 | -0.23 | -0.09 | -0.17 | -0.14 | 0.18 | 0.12 | 0.30 | 0.22 | 0.01 | -0.34 | -0.29 | -0.23 | -0.30 | -0.32 | -0.40 | -0.28 | -0.10 | -0.15 | -0.11 | -0.10 | -0.33 | -0.06 | -0.26 | -0.13 | -0.10 | -0.12 |
| TCGA.90.A4EE.01 | -0.488 | -0.48 | -0.03 | -0.11 | -0.14 | -0.14 | -0.20 | -0.19 | -0.31 | 0.05 | -0.36 | -0.29 | -0.03 | 0.04 | -0.18 | 0.18 | 0.39 | 0.21 | -0.11 | -0.10 | -0.19 | 0.01 | -0.07 | -0.19 | -0.25 | -0.26 | -0.10 | -0.14 | -0.22 |
| TCGA.90.A59Q.01 | -0.205 | 0.19 | 0.07 | -0.21 | 0.06 | -0.15 | -0.16 | -0.26 | -0.07 | -0.04 | -0.38 | -0.25 | 0.07 | -0.06 | -0.21 | -0.09 | -0.28 | -0.26 | 0.07 | -0.23 | -0.02 | 0.12 | -0.14 | -0.26 | -0.17 | -0.17 | -0.12 | -0.08 | 0.00 |
| TCGA.91.6828.01 | -0.184 | -0.13 | 0.35 | 0.21 | -0.09 | 0.28 | 0.20 | -0.10 | -0.12 | 0.14 | 0.02 | -0.24 | 0.35 | 0.12 | 0.18 | 0.48 | 0.52 | 0.38 | 0.02 | 0.09 | 0.33 | 0.09 | 0.28 | 0.05 | 0.32 | 0.27 | 0.23 | -0.21 | -0.25 |
| TCGA.91.6829.01 | -0.367 | 0.38 | 0.33 | 0.29 | 0.19 | 0.31 | 0.34 | 0.16 | 0.13 | 0.35 | 0.17 | 0.14 | 0.33 | 0.10 | 0.30 | 0.28 | 0.01 | 0.13 | -0.13 | 0.15 | 0.37 | 0.32 | 0.33 | 0.27 | 0.34 | 0.41 | 0.15 | 0.22 | -0.16 |
| TCGA.91.6830.01 | -0.291 | -0.38 | 0.15 | -0.11 | -0.32 | -0.21 | -0.22 | 0.01 | -0.19 | -0.21 | -0.26 | -0.11 | 0.15 | -0.16 | -0.21 | -0.07 | 0.03 | -0.19 | -0.31 | -0.25 | -0.08 | -0.24 | -0.22 | -0.10 | -0.16 | -0.21 | -0.16 | -0.23 | -0.11 |
| TCGA.91.6831.01 | -0.421 | 0.17 | 0.34 | 0.24 | 0.04 | 0.30 | 0.28 | 0.10 | 0.04 | 0.15 | 0.12 | 0.06 | 0.34 | 0.00 | 0.27 | 0.32 | 0.29 | 0.25 | -0.17 | 0.11 | 0.35 | 0.29 | 0.30 | 0.17 | 0.35 | 0.36 | 0.08 | 0.03 | -0.25 |
| TCGA.91.6835.01 | -0.154 | -0.54 | -0.05 | -0.11 | -0.26 | -0.16 | -0.16 | -0.06 | -0.24 | -0.41 | -0.35 | -0.29 | -0.05 | -0.18 | -0.24 | -0.19 | -0.08 | -0.09 | -0.04 | -0.24 | 0.00 | -0.33 | -0.29 | -0.23 | -0.05 | -0.12 | -0.11 | -0.25 | 0.03 |
| TCGA.91.6836.01 | 0.463 | 0.21 | -0.28 | 0.06 | 0.12 | 0.09 | 0.01 | 0.00 | 0.09 | -0.10 | 0.20 | 0.07 | -0.28 | 0.34 | 0.15 | 0.16 | 0.33 | 0.16 | 0.22 | 0.06 | -0.04 | -0.23 | 0.16 | -0.03 | 0.06 | 0.00 | 0.19 | 0.02 | 0.02 |
| TCGA.91.6840.01 | 0.177 | 0.15 | 0.19 | -0.09 | -0.09 | -0.17 | -0.12 | -0.14 | -0.06 | -0.19 | 0.17 | -0.15 | 0.19 | -0.20 | -0.02 | -0.25 | -0.28 | -0.24 | -0.06 | 0.04 | -0.06 | 0.12 | -0.20 | -0.13 | -0.09 | -0.06 | -0.09 | 0.14 | 0.05 |
| TCGA.91.6847.01 | -0.344 | -0.42 | -0.21 | -0.10 | 0.02 | -0.05 | -0.11 | 0.09 | 0.00 | -0.25 | -0.20 | 0.11 | -0.21 | -0.02 | -0.03 | -0.30 | -0.23 | -0.18 | -0.03 | -0.16 | -0.02 | -0.13 | -0.03 | -0.05 | -0.11 | -0.16 | -0.04 | -0.01 | -0.08 |
| TCGA.91.6848.01 | -0.144 | 0.12 | -0.01 | 0.16 | 0.18 | 0.19 | 0.06 | 0.26 | 0.22 | 0.18 | 0.22 | 0.22 | -0.01 | 0.15 | 0.22 | -0.11 | 0.08 | 0.02 | 0.18 | 0.20 | -0.10 | 0.14 | 0.17 | 0.16 | -0.09 | -0.01 | 0.12 | 0.22 | 0.18 |
| TCGA.91.7771.01 | -0.035 | -0.20 | 0.27 | 0.00 | -0.06 | 0.19 | 0.08 | 0.12 | -0.29 | 0.07 | 0.17 | 0.15 | 0.27 | -0.24 | 0.13 | 0.23 | 0.26 | 0.08 | -0.13 | 0.10 | 0.28 | 0.15 | 0.03 | -0.04 | 0.29 | 0.35 | 0.12 | -0.20 | -0.27 |
| TCGA.91.8496.01 | 0.027 | 0.51 | 0.08 | 0.34 | 0.27 | 0.28 | 0.35 | 0.18 | 0.34 | 0.39 | 0.26 | 0.24 | 0.08 | 0.22 | 0.36 | 0.36 | 0.31 | 0.34 | 0.03 | 0.28 | 0.32 | 0.41 | 0.35 | 0.34 | 0.37 | 0.41 | 0.18 | 0.30 | 0.09 |
| TCGA.91.8497.01 | -0.343 | -0.13 | -0.39 | -0.19 | -0.17 | -0.23 | -0.16 | -0.30 | -0.16 | -0.03 | -0.02 | -0.26 | -0.39 | -0.16 | -0.19 | -0.28 | -0.04 | -0.19 | 0.19 | -0.03 | -0.29 | -0.04 | -0.15 | -0.16 | -0.20 | -0.16 | -0.21 | 0.01 | 0.11 |
| TCGA.91.8499.01 | 0.070 | 0.54 | -0.37 | 0.06 | 0.23 | 0.01 | 0.13 | -0.09 | 0.30 | -0.08 | -0.01 | 0.04 | -0.37 | 0.29 | 0.06 | 0.15 | 0.36 | 0.23 | 0.32 | 0.23 | -0.03 | 0.18 | 0.09 | 0.27 | -0.12 | -0.05 | 0.17 | 0.18 | 0.27 |
| TCGA.91.A4BD.01 | -0.125 | 0.51 | -0.17 | 0.15 | 0.20 | 0.08 | 0.16 | 0.10 | 0.22 | 0.07 | 0.31 | 0.14 | -0.17 | 0.00 | -0.02 | 0.05 | -0.16 | -0.03 | 0.31 | 0.10 | 0.05 | -0.01 | -0.04 | 0.11 | 0.12 | 0.08 | 0.20 | 0.13 | 0.15 |
| TCGA.92.7340.01 | 0.033 | 0.23 | 0.21 | 0.25 | 0.13 | 0.30 | 0.30 | 0.20 | 0.14 | 0.14 | 0.18 | 0.13 | 0.21 | 0.22 | 0.31 | 0.33 | 0.40 | 0.33 | 0.14 | 0.25 | 0.39 | 0.12 | 0.34 | 0.39 | 0.34 | 0.42 | 0.32 | 0.10 | -0.17 |
| TCGA.92.7341.01 | 0.226 | 0.22 | 0.13 | 0.29 | 0.30 | 0.31 | 0.32 | 0.34 | 0.39 | 0.21 | 0.31 | 0.30 | 0.13 | 0.18 | 0.20 | 0.31 | 0.29 | 0.29 | 0.35 | 0.19 | 0.30 | 0.15 | 0.36 | -0.02 | 0.15 | 0.25 | 0.37 | 0.09 | -0.17 |
| TCGA.92.8063.01 | -0.241 | -0.23 | -0.11 | -0.17 | -0.17 | -0.21 | -0.19 | -0.25 | -0.07 | -0.29 | 0.06 | -0.24 | -0.11 | -0.20 | -0.19 | -0.33 | -0.25 | -0.27 | -0.07 | -0.04 | -0.01 | -0.09 | -0.16 | -0.29 | 0.02 | -0.01 | -0.20 | -0.01 | 0.07 |
| TCGA.92.8064.01 | 0.133 | -0.11 | -0.29 | -0.07 | 0.00 | -0.09 | 0.09 | 0.07 | 0.21 | 0.30 | 0.27 | 0.07 | -0.29 | -0.21 | 0.08 | -0.35 | -0.45 | -0.39 | -0.17 | -0.19 | -0.03 | -0.10 | 0.02 | -0.13 | 0.14 | 0.10 | -0.06 | 0.04 | 0.06 |
| TCGA.92.8065.01 | -0.453 | -0.39 | 0.25 | -0.03 | -0.24 | -0.01 | -0.01 | 0.05 | 0.01 | -0.18 | -0.20 | -0.15 | 0.25 | -0.22 | -0.21 | -0.05 | 0.05 | -0.08 | -0.31 | -0.31 | 0.19 | -0.19 | -0.05 | -0.23 | 0.06 | -0.12 | -0.01 | -0.32 | -0.32 |
| TCGA.93.7347.01 | -0.186 | -0.47 | -0.01 | -0.13 | -0.27 | -0.07 | -0.15 | -0.16 | -0.43 | -0.37 | -0.19 | -0.22 | -0.01 | -0.22 | -0.26 | -0.07 | -0.31 | -0.30 | -0.16 | -0.31 | 0.03 | -0.35 | -0.28 | -0.19 | -0.04 | -0.16 | -0.13 | -0.33 | -0.13 |
| TCGA.93.7348.01 | -0.034 | -0.37 | 0.15 | -0.05 | -0.17 | -0.14 | -0.16 | -0.09 | -0.06 | -0.18 | -0.28 | -0.15 | 0.15 | -0.18 | -0.21 | -0.14 | 0.09 | -0.07 | -0.10 | -0.14 | -0.02 | -0.10 | -0.17 | -0.19 | -0.16 | -0.02 | -0.14 | -0.03 | -0.14 |
| TCGA.93.8067.01 | -0.565 | -0.11 | 0.28 | 0.22 | 0.04 | 0.30 | 0.34 | 0.17 | 0.04 | 0.30 | 0.08 | 0.21 | 0.28 | 0.04 | 0.30 | 0.27 | 0.03 | 0.09 | -0.18 | 0.02 | 0.41 | 0.15 | 0.28 | 0.11 | 0.36 | 0.35 | 0.13 | -0.04 | -0.42 |
| TCGA.93.A4JO.01 | 0.007 | 0.52 | -0.03 | 0.06 | 0.19 | 0.11 | 0.04 | -0.02 | 0.20 | -0.06 | 0.04 | 0.09 | -0.03 | 0.11 | -0.04 | -0.20 | -0.20 | 0.02 | 0.07 | -0.03 | -0.13 | 0.03 | 0.01 | 0.31 | -0.02 | -0.11 | 0.03 | 0.13 | 0.23 |
| TCGA.93.A4JP.01 | -0.282 | -0.08 | 0.45 | 0.11 | -0.03 | 0.15 | 0.18 | 0.03 | 0.01 | 0.23 | -0.01 | -0.06 | 0.45 | -0.16 | 0.03 | 0.14 | -0.14 | 0.00 | -0.20 | 0.07 | 0.24 | 0.13 | 0.24 | 0.04 | 0.30 | 0.27 | 0.01 | 0.16 | 0.05 |
| TCGA.94.7033.01 | -0.336 | 0.35 | 0.23 | 0.24 | 0.08 | 0.33 | 0.32 | 0.26 | 0.05 | 0.38 | 0.30 | 0.26 | 0.23 | -0.09 | 0.22 | 0.29 | 0.00 | 0.13 | -0.08 | 0.24 | 0.37 | 0.32 | 0.29 | 0.19 | 0.35 | 0.38 | 0.22 | 0.18 | -0.16 |
| TCGA.94.7557.01 | -0.324 | 0.13 | -0.22 | -0.17 | 0.09 | -0.20 | -0.20 | -0.13 | 0.06 | 0.00 | 0.06 | 0.07 | -0.22 | -0.22 | -0.01 | -0.35 | -0.23 | -0.20 | 0.07 | -0.07 | -0.26 | 0.10 | -0.21 | -0.09 | -0.26 | -0.26 | -0.19 | 0.08 | 0.25 |
| TCGA.94.7943.01 | 0.340 | -0.30 | -0.23 | -0.18 | -0.21 | -0.12 | -0.07 | -0.16 | 0.15 | -0.30 | -0.07 | -0.16 | -0.23 | -0.14 | -0.17 | 0.08 | 0.32 | 0.21 | -0.04 | -0.21 | -0.03 | 0.19 | -0.18 | -0.18 | -0.15 | -0.17 | -0.21 | -0.15 | 0.22 |
| TCGA.94.8035.01 | 0.446 | 0.19 | 0.08 | 0.18 | 0.26 | 0.21 | 0.14 | 0.18 | 0.03 | 0.38 | 0.04 | 0.02 | 0.08 | 0.11 | 0.18 | -0.03 | 0.02 | 0.07 | -0.05 | 0.06 | 0.20 | 0.37 | 0.22 | 0.24 | 0.27 | 0.34 | 0.14 | 0.26 | 0.10 |
| TCGA.94.8491.01 | -0.563 | -0.29 | -0.25 | 0.06 | 0.11 | 0.07 | -0.06 | -0.05 | 0.10 | -0.33 | -0.24 | -0.01 | -0.25 | 0.14 | 0.06 | 0.01 | 0.24 | 0.13 | -0.18 | 0.03 | -0.05 | -0.05 | 0.04 | 0.11 | 0.08 | -0.05 | -0.07 | -0.04 | 0.05 |
| TCGA.94.A4VJ.01 | 0.537 | 0.16 | 0.23 | 0.18 | 0.13 | 0.19 | 0.17 | 0.22 | 0.15 | 0.29 | 0.27 | 0.09 | 0.23 | 0.15 | 0.31 | 0.19 | 0.32 | 0.02 | -0.05 | 0.16 | 0.25 | 0.26 | 0.30 | 0.18 | 0.08 | 0.25 | 0.20 | 0.17 | 0.04 |
| TCGA.94.A5I4.01 | 0.511 | 0.07 | -0.22 | -0.22 | -0.18 | -0.19 | -0.16 | 0.06 | 0.17 | 0.13 | 0.16 | 0.14 | -0.22 | -0.25 | -0.16 | -0.30 | -0.35 | -0.33 | -0.12 | 0.06 | -0.33 | 0.03 | -0.25 | -0.10 | -0.29 | -0.23 | -0.20 | -0.04 | 0.21 |
| TCGA.94.A5I6.01 | 0.166 | -0.39 | -0.34 | -0.31 | -0.28 | -0.30 | -0.29 | -0.29 | 0.01 | -0.27 | -0.15 | -0.26 | -0.34 | -0.14 | -0.09 | -0.39 | -0.43 | -0.37 | -0.14 | -0.25 | -0.21 | -0.06 | -0.30 | -0.18 | -0.24 | -0.18 | -0.32 | -0.04 | 0.25 |
| TCGA.95.7039.01 | -0.229 | 0.27 | -0.15 | 0.10 | 0.11 | 0.04 | 0.15 | 0.14 | 0.27 | -0.22 | 0.02 | 0.09 | -0.15 | 0.33 | -0.01 | 0.01 | -0.32 | -0.06 | 0.02 | -0.02 | 0.17 | -0.19 | 0.10 | 0.04 | 0.03 | 0.10 | 0.17 | 0.04 | -0.06 |
| TCGA.95.7043.01 | 0.222 | 0.23 | 0.14 | -0.06 | 0.00 | -0.15 | -0.08 | 0.14 | -0.14 | 0.08 | 0.20 | 0.17 | 0.14 | -0.19 | -0.04 | -0.15 | -0.19 | -0.07 | 0.02 | -0.05 | -0.19 | -0.04 | -0.12 | -0.04 | -0.22 | -0.09 | -0.08 | -0.02 | -0.02 |
| TCGA.95.7562.01 | 0.623 | 0.23 | -0.01 | -0.03 | 0.10 | -0.04 | 0.13 | 0.03 | 0.33 | 0.22 | -0.10 | 0.06 | -0.01 | 0.05 | 0.13 | -0.17 | -0.42 | -0.26 | 0.02 | -0.01 | -0.04 | 0.26 | 0.03 | 0.08 | 0.00 | 0.17 | -0.03 | 0.16 | 0.21 |
| TCGA.95.7567.01 | -0.455 | 0.31 | 0.36 | 0.34 | 0.09 | 0.35 | 0.29 | 0.03 | -0.08 | 0.26 | 0.12 | -0.04 | 0.36 | 0.40 | 0.29 | 0.43 | 0.45 | 0.36 | 0.09 | 0.15 | 0.41 | 0.17 | 0.38 | 0.24 | 0.31 | 0.37 | 0.31 | -0.05 | -0.17 |
| TCGA.95.7944.01 | 0.188 | 0.27 | -0.31 | -0.15 | -0.24 | -0.19 | -0.19 | -0.23 | -0.19 | -0.16 | -0.14 | -0.21 | -0.31 | -0.10 | -0.09 | 0.18 | 0.35 | 0.09 | -0.22 | -0.22 | -0.18 | -0.10 | -0.14 | -0.20 | -0.22 | -0.21 | -0.02 | -0.19 | -0.15 |
| TCGA.95.7947.01 | 0.515 | -0.21 | -0.02 | -0.23 | -0.02 | -0.18 | -0.24 | -0.22 | 0.09 | 0.05 | -0.37 | -0.31 | -0.02 | -0.25 | -0.10 | -0.16 | 0.02 | -0.11 | -0.29 | 0.04 | -0.24 | 0.25 | -0.11 | 0.09 | -0.17 | -0.14 | -0.26 | 0.18 | 0.30 |
| TCGA.95.7948.01 | 0.305 | 0.51 | 0.17 | 0.26 | 0.36 | 0.11 | 0.14 | 0.25 | 0.27 | 0.17 | 0.10 | 0.20 | 0.17 | 0.35 | -0.09 | 0.25 | 0.13 | 0.27 | 0.13 | 0.19 | -0.01 | 0.01 | 0.23 | 0.14 | 0.02 | -0.13 | 0.16 | 0.09 | 0.30 |
| TCGA.95.8494.01 | -0.317 | 0.30 | 0.29 | 0.30 | 0.13 | 0.31 | 0.38 | 0.22 | 0.22 | 0.44 | 0.20 | 0.24 | 0.29 | 0.06 | 0.25 | 0.28 | 0.10 | 0.19 | -0.06 | 0.22 | 0.43 | 0.28 | 0.36 | 0.24 | 0.34 | 0.37 | 0.21 | 0.18 | -0.20 |
| TCGA.95.A4VK.01 | 0.366 | 0.53 | 0.10 | 0.22 | 0.27 | 0.24 | 0.23 | 0.23 | 0.25 | 0.23 | 0.21 | 0.23 | 0.10 | 0.26 | 0.32 | 0.34 | 0.27 | 0.26 | 0.29 | 0.27 | 0.10 | 0.34 | 0.37 | 0.32 | 0.03 | 0.30 | 0.32 | 0.30 | 0.04 |
| TCGA.95.A4VN.01 | 0.351 | 0.25 | 0.07 | -0.14 | -0.06 | -0.14 | -0.21 | -0.13 | 0.10 | 0.13 | 0.02 | -0.19 | 0.07 | -0.20 | -0.09 | -0.24 | -0.34 | -0.20 | -0.07 | 0.04 | -0.29 | 0.15 | -0.22 | -0.09 | -0.15 | -0.14 | -0.17 | 0.20 | 0.15 |
| TCGA.96.7544.01 | 0.291 | 0.15 | -0.03 | 0.00 | 0.29 | 0.04 | -0.05 | 0.05 | 0.13 | 0.07 | -0.13 | -0.04 | -0.03 | 0.30 | 0.04 | 0.15 | 0.37 | 0.25 | 0.21 | 0.20 | -0.16 | 0.29 | -0.05 | 0.25 | -0.06 | -0.16 | -0.07 | 0.13 | 0.24 |
| TCGA.96.7545.01 | 0.367 | 0.52 | 0.32 | 0.38 | 0.28 | 0.38 | 0.47 | 0.31 | 0.41 | 0.48 | 0.31 | 0.28 | 0.32 | 0.24 | 0.40 | 0.34 | 0.17 | 0.26 | 0.15 | 0.32 | 0.38 | 0.42 | 0.43 | 0.32 | 0.39 | 0.47 | 0.35 | 0.30 | 0.07 |
| TCGA.96.8169.01 | -0.258 | 0.21 | 0.04 | 0.10 | -0.05 | 0.03 | -0.04 | 0.08 | -0.10 | 0.25 | 0.16 | 0.07 | 0.04 | 0.01 | -0.06 | 0.21 | 0.38 | 0.32 | 0.09 | 0.18 | 0.02 | -0.03 | 0.05 | -0.11 | 0.00 | 0.01 | 0.14 | 0.02 | -0.22 |
| TCGA.96.8170.01 | 0.360 | 0.33 | 0.05 | -0.06 | -0.04 | -0.10 | 0.02 | -0.14 | -0.25 | -0.19 | -0.07 | -0.15 | 0.05 | -0.30 | -0.13 | -0.27 | -0.29 | -0.19 | 0.08 | -0.05 | -0.03 | -0.05 | -0.25 | -0.16 | -0.14 | -0.03 | -0.03 | 0.00 | 0.11 |
| TCGA.96.A4JK.01 | 0.041 | -0.18 | 0.09 | -0.19 | -0.05 | -0.20 | -0.23 | 0.09 | 0.19 | 0.04 | -0.23 | 0.04 | 0.09 | -0.19 | -0.07 | -0.16 | -0.21 | -0.24 | 0.04 | -0.12 | -0.13 | -0.04 | 0.00 | -0.03 | -0.15 | -0.22 | -0.10 | 0.05 | -0.02 |
| TCGA.96.A4JL.01 | -0.405 | -0.14 | 0.29 | -0.03 | -0.21 | 0.04 | -0.06 | -0.01 | 0.11 | 0.31 | -0.14 | -0.10 | 0.29 | 0.03 | -0.14 | 0.21 | 0.42 | 0.18 | -0.29 | 0.02 | -0.01 | 0.07 | 0.09 | -0.14 | 0.00 | -0.15 | -0.01 | -0.15 | -0.20 |
| TCGA.97.7547.01 | -0.019 | -0.15 | -0.32 | -0.14 | 0.06 | -0.14 | -0.12 | -0.09 | -0.07 | -0.18 | -0.27 | -0.03 | -0.32 | 0.16 | -0.12 | -0.19 | -0.13 | -0.19 | -0.09 | -0.16 | -0.06 | 0.01 | 0.03 | -0.13 | -0.08 | -0.19 | -0.08 | -0.18 | 0.07 |
| TCGA.97.7552.01 | -0.422 | -0.11 | -0.20 | 0.02 | -0.14 | 0.09 | 0.19 | 0.04 | -0.01 | -0.13 | 0.07 | 0.05 | -0.20 | 0.16 | 0.11 | 0.07 | 0.00 | -0.08 | 0.17 | -0.08 | 0.21 | -0.24 | 0.14 | -0.02 | 0.12 | 0.16 | 0.19 | -0.12 | -0.25 |
| TCGA.97.7553.01 | 0.455 | 0.27 | -0.03 | -0.13 | -0.02 | -0.20 | -0.11 | -0.12 | 0.22 | -0.16 | -0.14 | -0.11 | -0.03 | -0.14 | -0.28 | -0.15 | -0.25 | -0.02 | 0.10 | -0.18 | -0.26 | -0.26 | -0.27 | -0.03 | -0.16 | -0.29 | -0.25 | -0.07 | 0.13 |
| TCGA.97.7554.01 | -0.603 | -0.33 | 0.36 | -0.12 | -0.21 | -0.14 | -0.22 | -0.18 | -0.28 | -0.14 | -0.28 | -0.24 | 0.36 | -0.26 | -0.27 | -0.12 | -0.06 | -0.26 | -0.19 | -0.33 | 0.09 | -0.17 | -0.13 | -0.25 | -0.03 | -0.12 | -0.15 | -0.29 | -0.33 |
| TCGA.97.7937.01 | -0.122 | 0.40 | 0.10 | 0.30 | 0.22 | 0.35 | 0.39 | 0.29 | 0.18 | 0.41 | 0.31 | 0.26 | 0.10 | 0.10 | 0.41 | 0.33 | 0.28 | 0.35 | -0.02 | 0.24 | 0.42 | 0.35 | 0.33 | 0.30 | 0.38 | 0.50 | 0.26 | 0.14 | -0.21 |
| TCGA.97.7938.01 | -0.370 | 0.15 | 0.48 | 0.15 | -0.13 | 0.20 | 0.13 | 0.08 | 0.01 | 0.50 | -0.02 | -0.05 | 0.48 | -0.03 | -0.04 | 0.21 | 0.14 | -0.02 | -0.26 | -0.14 | 0.24 | -0.05 | 0.14 | -0.07 | 0.27 | 0.02 | 0.04 | -0.10 | -0.41 |
| TCGA.97.8171.01 | 0.193 | 0.02 | -0.03 | -0.22 | -0.25 | -0.24 | -0.25 | -0.05 | 0.09 | -0.28 | -0.38 | -0.31 | -0.03 | -0.11 | -0.30 | -0.37 | -0.41 | -0.34 | -0.17 | -0.18 | -0.32 | -0.05 | -0.35 | -0.16 | -0.29 | -0.23 | -0.28 | 0.00 | 0.31 |
| TCGA.97.8174.01 | -0.444 | -0.22 | 0.14 | -0.11 | -0.09 | 0.04 | -0.15 | -0.17 | -0.34 | -0.06 | -0.12 | -0.23 | 0.14 | -0.17 | -0.26 | 0.11 | 0.09 | -0.23 | -0.16 | -0.15 | 0.03 | -0.03 | -0.20 | -0.07 | 0.01 | -0.10 | -0.11 | -0.23 | -0.20 |
| TCGA.97.8175.01 | -0.106 | -0.33 | -0.28 | -0.16 | -0.30 | -0.21 | -0.26 | -0.12 | -0.41 | -0.31 | 0.14 | -0.04 | -0.28 | -0.14 | -0.05 | -0.16 | -0.18 | -0.11 | -0.18 | -0.08 | -0.19 | -0.29 | -0.23 | -0.24 | -0.23 | -0.21 | -0.09 | -0.32 | -0.04 |
| TCGA.97.8177.01 | 0.469 | 0.28 | -0.26 | 0.06 | 0.01 | 0.10 | 0.09 | -0.01 | 0.28 | 0.15 | 0.35 | 0.08 | -0.26 | -0.06 | 0.25 | -0.03 | 0.03 | 0.09 | 0.30 | 0.25 | 0.02 | 0.09 | 0.09 | 0.04 | 0.06 | 0.23 | 0.12 | 0.16 | 0.16 |
| TCGA.97.8552.01 | -0.382 | -0.40 | 0.36 | -0.07 | -0.34 | -0.09 | -0.02 | -0.09 | -0.23 | 0.17 | -0.24 | -0.27 | 0.36 | -0.22 | -0.20 | -0.03 | -0.08 | -0.28 | -0.37 | -0.27 | 0.30 | -0.14 | -0.02 | -0.23 | 0.12 | -0.03 | -0.16 | -0.30 | -0.22 |
| TCGA.97.A4LX.01 | -0.006 | -0.08 | 0.28 | 0.12 | 0.16 | 0.20 | 0.17 | 0.27 | 0.16 | 0.31 | 0.35 | 0.26 | 0.28 | 0.03 | 0.18 | 0.18 | 0.43 | 0.04 | 0.03 | 0.14 | 0.24 | 0.12 | 0.27 | 0.13 | 0.28 | 0.25 | 0.15 | -0.14 | -0.28 |
| TCGA.97.A4M0.01 | 0.509 | 0.60 | 0.00 | 0.34 | 0.44 | 0.30 | 0.32 | 0.31 | 0.27 | 0.28 | 0.33 | 0.26 | 0.00 | 0.43 | 0.42 | 0.43 | 0.35 | 0.47 | 0.44 | 0.39 | 0.28 | 0.27 | 0.40 | 0.30 | 0.27 | 0.38 | 0.41 | 0.21 | 0.07 |
| TCGA.97.A4M1.01 | -0.253 | -0.39 | 0.35 | -0.05 | -0.31 | -0.03 | -0.02 | -0.08 | -0.14 | -0.14 | -0.33 | -0.25 | 0.35 | -0.15 | -0.15 | -0.12 | -0.37 | -0.35 | -0.36 | -0.31 | 0.12 | -0.15 | 0.01 | -0.23 | 0.11 | -0.11 | -0.20 | -0.16 | -0.02 |
| TCGA.97.A4M2.01 | -0.359 | -0.44 | 0.40 | 0.00 | -0.16 | 0.06 | -0.08 | -0.12 | -0.22 | -0.12 | -0.26 | -0.22 | 0.40 | -0.04 | -0.02 | 0.19 | 0.16 | -0.09 | -0.29 | -0.25 | 0.24 | 0.10 | 0.12 | -0.16 | 0.12 | 0.08 | -0.16 | -0.22 | -0.12 |
| TCGA.97.A4M3.01 | -0.149 | 0.34 | -0.02 | 0.15 | -0.08 | 0.19 | 0.12 | 0.09 | 0.08 | 0.16 | 0.19 | 0.10 | -0.02 | -0.03 | 0.04 | 0.18 | 0.10 | -0.05 | 0.09 | -0.06 | 0.30 | 0.06 | 0.13 | 0.06 | 0.06 | 0.16 | 0.22 | 0.03 | -0.23 |
| TCGA.97.A4M5.01 | 0.066 | 0.36 | -0.03 | 0.32 | 0.24 | 0.33 | 0.31 | 0.20 | 0.15 | 0.35 | 0.36 | 0.26 | -0.03 | 0.26 | 0.31 | 0.47 | 0.46 | 0.37 | 0.26 | 0.24 | 0.19 | 0.20 | 0.37 | 0.31 | 0.29 | 0.34 | 0.32 | 0.28 | -0.12 |
| TCGA.97.A4M6.01 | -0.055 | 0.18 | 0.19 | 0.23 | 0.21 | 0.17 | 0.17 | 0.30 | 0.25 | 0.28 | 0.30 | 0.22 | 0.19 | 0.20 | 0.28 | 0.20 | 0.18 | 0.24 | 0.25 | 0.08 | 0.21 | 0.10 | 0.24 | 0.10 | 0.19 | 0.23 | 0.25 | 0.06 | -0.31 |
| TCGA.97.A4M7.01 | 0.242 | -0.13 | 0.17 | -0.13 | 0.19 | 0.01 | -0.10 | -0.09 | -0.19 | -0.05 | -0.07 | 0.01 | 0.17 | -0.08 | -0.02 | -0.17 | -0.23 | -0.29 | -0.07 | 0.09 | -0.19 | 0.21 | 0.02 | 0.01 | -0.22 | -0.14 | -0.04 | 0.24 | 0.14 |
| TCGA.98.7454.01 | 0.399 | 0.30 | 0.23 | 0.19 | 0.04 | 0.24 | 0.19 | -0.01 | -0.15 | 0.16 | 0.15 | -0.06 | 0.23 | -0.04 | 0.21 | 0.35 | 0.32 | 0.28 | 0.11 | 0.17 | 0.16 | 0.15 | 0.14 | 0.10 | 0.15 | 0.26 | 0.30 | 0.10 | -0.12 |
| TCGA.98.8020.01 | -0.527 | 0.10 | 0.37 | 0.27 | 0.10 | 0.32 | 0.35 | 0.15 | -0.12 | 0.35 | 0.22 | 0.21 | 0.37 | 0.09 | 0.33 | 0.44 | 0.36 | 0.32 | -0.06 | 0.13 | 0.40 | 0.25 | 0.34 | 0.19 | 0.40 | 0.38 | 0.24 | 0.01 | -0.37 |
| TCGA.98.8021.01 | 0.143 | 0.06 | -0.10 | 0.02 | 0.07 | 0.10 | 0.12 | 0.18 | 0.05 | 0.19 | 0.20 | 0.28 | -0.10 | 0.11 | 0.10 | -0.15 | -0.18 | -0.13 | -0.20 | 0.00 | 0.19 | -0.13 | 0.12 | -0.05 | 0.19 | 0.06 | 0.14 | -0.04 | -0.19 |
| TCGA.98.8022.01 | 0.157 | -0.41 | -0.04 | -0.18 | -0.27 | -0.10 | -0.06 | -0.01 | 0.18 | -0.28 | -0.12 | -0.15 | -0.04 | -0.10 | -0.11 | -0.34 | -0.47 | -0.39 | -0.17 | -0.32 | 0.10 | -0.25 | -0.13 | -0.20 | -0.02 | -0.01 | -0.16 | -0.20 | -0.05 |
| TCGA.98.8023.01 | -0.210 | 0.11 | -0.21 | 0.00 | -0.29 | 0.07 | -0.04 | -0.01 | 0.02 | -0.05 | 0.04 | -0.02 | -0.21 | -0.02 | -0.06 | 0.22 | 0.43 | 0.33 | 0.01 | -0.09 | 0.06 | -0.16 | 0.08 | -0.10 | -0.03 | -0.06 | 0.16 | -0.14 | -0.19 |
| TCGA.98.A538.01 | -0.292 | 0.40 | 0.25 | 0.27 | 0.15 | 0.34 | 0.38 | 0.25 | 0.05 | 0.33 | 0.25 | 0.27 | 0.25 | 0.05 | 0.33 | 0.38 | 0.13 | 0.12 | -0.01 | 0.26 | 0.35 | 0.30 | 0.29 | 0.23 | 0.41 | 0.43 | 0.21 | 0.19 | -0.19 |
| TCGA.98.A539.01 | -0.204 | 0.07 | 0.10 | 0.12 | 0.28 | 0.08 | 0.09 | 0.08 | 0.07 | -0.03 | -0.03 | 0.15 | 0.10 | 0.23 | 0.12 | 0.03 | 0.16 | 0.18 | 0.09 | 0.01 | 0.06 | -0.03 | 0.12 | 0.17 | -0.01 | 0.05 | 0.03 | 0.09 | 0.06 |
| TCGA.98.A53A.01 | -0.129 | -0.36 | 0.02 | -0.22 | 0.05 | -0.14 | -0.12 | 0.02 | 0.05 | 0.22 | -0.10 | 0.05 | 0.02 | 0.02 | -0.19 | -0.34 | -0.34 | -0.36 | -0.03 | -0.09 | -0.26 | -0.20 | -0.09 | -0.20 | -0.10 | -0.28 | -0.19 | 0.03 | 0.07 |
| TCGA.98.A53B.01 | 0.446 | 0.20 | -0.51 | 0.05 | 0.14 | -0.08 | -0.11 | 0.15 | 0.15 | 0.37 | 0.09 | 0.25 | -0.51 | 0.14 | -0.07 | 0.02 | 0.02 | -0.08 | 0.10 | 0.14 | -0.37 | 0.00 | -0.06 | 0.10 | -0.09 | -0.22 | -0.01 | 0.13 | 0.24 |
| TCGA.98.A53C.01 | -0.319 | 0.06 | 0.41 | 0.23 | 0.00 | 0.36 | 0.30 | 0.01 | -0.06 | 0.14 | 0.20 | -0.04 | 0.41 | 0.22 | 0.27 | 0.47 | 0.37 | 0.31 | -0.03 | 0.16 | 0.37 | 0.29 | 0.38 | 0.05 | 0.28 | 0.33 | 0.23 | -0.09 | -0.33 |
| TCGA.98.A53D.01 | -0.392 | 0.18 | -0.35 | 0.02 | 0.05 | -0.03 | -0.11 | 0.04 | -0.10 | 0.16 | 0.15 | 0.04 | -0.35 | 0.10 | 0.06 | 0.14 | 0.31 | 0.23 | -0.01 | 0.14 | -0.19 | -0.05 | -0.02 | -0.17 | -0.15 | -0.09 | 0.12 | 0.09 | 0.01 |
| TCGA.98.A53H.01 | -0.343 | -0.15 | 0.40 | 0.10 | -0.07 | 0.25 | 0.16 | 0.07 | 0.00 | 0.23 | -0.01 | -0.17 | 0.40 | 0.02 | 0.26 | 0.35 | 0.32 | 0.21 | -0.29 | 0.03 | 0.41 | 0.24 | 0.29 | -0.03 | 0.31 | 0.35 | 0.18 | -0.25 | -0.36 |
| TCGA.98.A53I.01 | -0.058 | 0.06 | -0.29 | 0.08 | 0.13 | -0.06 | 0.00 | 0.14 | 0.00 | -0.12 | 0.08 | 0.03 | -0.29 | 0.25 | 0.09 | 0.20 | 0.16 | 0.28 | 0.03 | 0.16 | -0.11 | -0.11 | -0.05 | 0.10 | 0.04 | -0.13 | -0.02 | -0.07 | 0.08 |
| TCGA.98.A53J.01 | -0.339 | -0.33 | 0.29 | -0.04 | 0.19 | -0.10 | -0.12 | 0.00 | -0.04 | 0.10 | -0.08 | 0.05 | 0.29 | 0.00 | -0.03 | -0.20 | -0.15 | 0.03 | 0.09 | -0.04 | -0.21 | 0.03 | 0.00 | -0.12 | -0.07 | -0.28 | -0.18 | 0.03 | 0.09 |
| TCGA.99.7458.01 | -0.113 | 0.25 | 0.12 | 0.08 | 0.00 | 0.13 | 0.04 | -0.02 | 0.02 | 0.34 | 0.01 | -0.04 | 0.12 | -0.06 | 0.03 | 0.21 | 0.25 | 0.05 | -0.10 | -0.04 | 0.19 | 0.20 | 0.03 | 0.04 | 0.13 | 0.16 | 0.05 | -0.10 | -0.21 |
| TCGA.99.8025.01 | -0.189 | -0.19 | -0.22 | -0.19 | -0.02 | -0.14 | -0.11 | -0.14 | 0.17 | -0.25 | -0.22 | -0.12 | -0.22 | -0.20 | -0.16 | -0.34 | -0.31 | -0.26 | -0.12 | -0.29 | -0.12 | -0.05 | -0.13 | -0.26 | -0.19 | -0.13 | -0.13 | -0.16 | 0.10 |
| TCGA.99.8028.01 | -0.320 | -0.04 | -0.17 | -0.16 | -0.05 | -0.13 | -0.17 | 0.05 | 0.02 | 0.02 | -0.01 | -0.05 | -0.17 | -0.05 | -0.06 | -0.27 | -0.37 | -0.35 | 0.05 | -0.20 | 0.09 | -0.11 | -0.11 | -0.18 | 0.02 | -0.14 | -0.03 | -0.16 | -0.22 |
| TCGA.99.8032.01 | 0.434 | -0.31 | -0.12 | -0.16 | -0.32 | -0.08 | -0.12 | 0.00 | 0.07 | -0.03 | 0.15 | -0.08 | -0.12 | -0.30 | 0.01 | -0.31 | -0.18 | -0.36 | -0.14 | -0.09 | 0.12 | -0.17 | 0.04 | -0.23 | -0.01 | 0.24 | -0.05 | 0.00 | -0.04 |
| TCGA.99.8033.01 | 0.336 | -0.27 | -0.03 | -0.19 | -0.15 | -0.27 | -0.22 | 0.07 | 0.12 | -0.33 | -0.08 | -0.04 | -0.03 | -0.11 | -0.26 | -0.25 | -0.18 | -0.13 | -0.27 | -0.18 | -0.24 | -0.26 | -0.29 | -0.12 | -0.24 | -0.15 | -0.25 | -0.06 | 0.15 |
| TCGA.99.AA5R.01 | 0.463 | -0.19 | -0.06 | -0.11 | -0.08 | -0.09 | -0.14 | 0.03 | 0.16 | 0.20 | -0.04 | -0.17 | -0.06 | -0.25 | -0.08 | -0.27 | -0.37 | -0.27 | -0.02 | 0.03 | -0.20 | 0.06 | -0.07 | 0.06 | -0.01 | 0.21 | -0.05 | 0.25 | 0.29 |
| TCGA.J1.A4AH.01 | -0.140 | 0.49 | -0.10 | 0.08 | 0.21 | 0.07 | 0.03 | -0.06 | 0.12 | 0.15 | 0.01 | -0.01 | -0.10 | 0.04 | 0.20 | 0.13 | 0.15 | 0.12 | 0.14 | 0.13 | -0.03 | 0.25 | 0.03 | 0.10 | -0.11 | 0.00 | 0.12 | 0.13 | 0.12 |
| TCGA.J2.8192.01 | 0.010 | 0.47 | -0.02 | 0.14 | 0.07 | 0.17 | 0.13 | 0.07 | 0.00 | 0.39 | 0.07 | 0.02 | -0.02 | -0.15 | -0.01 | 0.29 | 0.10 | 0.30 | 0.01 | 0.11 | 0.15 | 0.25 | 0.18 | 0.18 | 0.00 | 0.14 | 0.19 | -0.10 | 0.09 |
| TCGA.J2.8194.01 | 0.529 | 0.38 | 0.15 | 0.28 | 0.42 | 0.25 | 0.29 | 0.31 | 0.26 | 0.27 | 0.31 | 0.36 | 0.15 | 0.38 | 0.27 | 0.07 | 0.04 | 0.08 | 0.23 | 0.23 | 0.11 | 0.30 | 0.28 | 0.33 | 0.23 | 0.26 | 0.24 | 0.26 | -0.02 |
| TCGA.J2.A4AD.01 | -0.171 | -0.31 | -0.25 | -0.16 | -0.20 | -0.23 | -0.23 | -0.22 | -0.10 | -0.29 | -0.12 | -0.13 | -0.25 | -0.24 | -0.33 | -0.33 | -0.36 | -0.29 | -0.28 | -0.18 | -0.14 | -0.01 | -0.28 | -0.13 | -0.13 | -0.31 | -0.24 | -0.16 | 0.01 |
| TCGA.J2.A4AE.01 | -0.353 | 0.25 | -0.01 | -0.04 | -0.03 | -0.20 | -0.16 | 0.11 | 0.19 | 0.10 | 0.10 | 0.09 | -0.01 | -0.07 | -0.20 | 0.01 | 0.10 | 0.06 | 0.29 | 0.13 | -0.24 | 0.03 | -0.12 | -0.07 | -0.07 | -0.17 | -0.10 | 0.14 | 0.18 |
| TCGA.J2.A4AG.01 | 0.378 | 0.04 | -0.30 | -0.11 | 0.11 | -0.09 | 0.05 | -0.04 | 0.11 | -0.02 | -0.03 | -0.03 | -0.30 | 0.08 | -0.16 | -0.16 | -0.24 | -0.01 | 0.20 | 0.07 | -0.34 | -0.12 | -0.06 | 0.17 | -0.05 | -0.11 | -0.11 | 0.19 | 0.19 |
| TCGA.L3.A4E7.01 | -0.350 | -0.24 | 0.02 | -0.02 | 0.04 | -0.03 | 0.01 | 0.03 | 0.20 | -0.04 | -0.13 | -0.04 | 0.02 | 0.15 | -0.15 | 0.02 | 0.30 | 0.26 | 0.11 | -0.08 | 0.04 | -0.05 | 0.07 | -0.18 | -0.13 | -0.24 | -0.03 | -0.05 | -0.07 |
| TCGA.L3.A524.01 | 0.249 | -0.18 | -0.18 | -0.04 | 0.15 | -0.23 | -0.13 | 0.14 | 0.26 | -0.06 | 0.07 | 0.12 | -0.18 | -0.04 | -0.21 | -0.31 | -0.42 | -0.33 | 0.05 | -0.03 | -0.17 | -0.04 | -0.08 | -0.13 | -0.24 | -0.24 | -0.12 | 0.00 | -0.05 |
| TCGA.L4.A4E5.01 | -0.190 | 0.01 | -0.21 | -0.06 | -0.33 | -0.01 | -0.07 | -0.06 | -0.25 | 0.11 | 0.09 | -0.16 | -0.21 | -0.20 | -0.12 | 0.10 | 0.12 | 0.00 | -0.10 | -0.13 | -0.03 | -0.17 | -0.03 | -0.16 | -0.15 | -0.05 | 0.02 | -0.32 | -0.10 |
| TCGA.L4.A4E6.01 | 0.251 | 0.27 | -0.25 | -0.09 | -0.02 | -0.13 | -0.13 | -0.20 | -0.04 | -0.41 | -0.33 | -0.26 | -0.25 | 0.29 | -0.01 | -0.06 | -0.20 | -0.10 | 0.21 | -0.09 | 0.04 | 0.12 | -0.09 | 0.11 | -0.10 | -0.05 | -0.07 | 0.07 | 0.24 |
| TCGA.L9.A443.01 | -0.516 | -0.30 | 0.12 | 0.08 | 0.01 | -0.03 | 0.02 | -0.03 | -0.16 | -0.30 | -0.12 | -0.12 | 0.12 | 0.15 | -0.08 | -0.05 | -0.19 | -0.15 | -0.23 | -0.21 | 0.18 | -0.14 | -0.01 | -0.12 | 0.06 | -0.07 | -0.01 | -0.21 | -0.02 |
| TCGA.L9.A444.01 | 0.327 | 0.14 | -0.03 | -0.03 | -0.09 | -0.05 | 0.05 | 0.03 | 0.02 | -0.30 | 0.01 | 0.05 | -0.03 | 0.11 | 0.15 | 0.02 | 0.10 | 0.14 | 0.12 | 0.18 | -0.13 | -0.19 | -0.09 | 0.16 | -0.04 | 0.06 | 0.06 | 0.05 | 0.14 |
| TCGA.L9.A50W.01 | -0.365 | -0.43 | 0.41 | -0.04 | -0.10 | -0.06 | -0.14 | 0.07 | 0.04 | 0.25 | -0.22 | -0.05 | 0.41 | -0.21 | -0.07 | -0.13 | -0.12 | -0.07 | -0.35 | -0.17 | 0.25 | 0.21 | -0.04 | -0.10 | 0.19 | 0.13 | -0.18 | -0.01 | -0.25 |
| TCGA.L9.A5IP.01 | -0.265 | -0.20 | -0.08 | -0.18 | -0.12 | -0.16 | -0.01 | -0.13 | 0.08 | -0.03 | -0.12 | -0.18 | -0.08 | -0.08 | 0.07 | -0.15 | -0.14 | -0.36 | -0.09 | -0.04 | -0.02 | 0.10 | -0.09 | -0.05 | -0.10 | -0.06 | -0.17 | -0.02 | 0.03 |
| TCGA.L9.A743.01 | -0.479 | -0.10 | -0.04 | -0.09 | 0.04 | -0.21 | -0.31 | -0.07 | 0.11 | -0.22 | -0.19 | -0.04 | -0.04 | -0.10 | -0.21 | -0.22 | -0.34 | -0.17 | 0.00 | -0.07 | -0.25 | -0.02 | -0.18 | -0.09 | -0.15 | -0.30 | -0.26 | 0.09 | 0.07 |
| TCGA.L9.A7SV.01 | 0.360 | 0.12 | 0.20 | -0.01 | -0.05 | -0.05 | 0.02 | 0.02 | 0.15 | 0.06 | 0.26 | 0.08 | 0.20 | -0.16 | 0.03 | -0.14 | -0.10 | -0.13 | 0.12 | 0.08 | 0.03 | -0.02 | -0.21 | 0.16 | 0.17 | 0.19 | 0.07 | 0.18 | -0.06 |
| TCGA.L9.A8F4.01 | -0.059 | -0.11 | -0.28 | -0.09 | 0.09 | -0.17 | -0.24 | 0.05 | 0.09 | -0.06 | -0.11 | 0.09 | -0.28 | -0.04 | -0.27 | -0.13 | -0.02 | 0.15 | 0.14 | -0.05 | -0.27 | -0.24 | -0.19 | -0.08 | -0.18 | -0.34 | -0.10 | 0.01 | 0.10 |
| TCGA.LA.A446.01 | 0.226 | -0.21 | -0.12 | -0.23 | -0.09 | -0.28 | -0.23 | 0.06 | 0.19 | 0.27 | -0.09 | 0.03 | -0.12 | -0.21 | -0.09 | -0.35 | -0.24 | -0.29 | -0.27 | -0.15 | -0.25 | 0.09 | -0.15 | -0.08 | -0.22 | -0.23 | -0.26 | 0.16 | 0.13 |
| TCGA.LA.A7SW.01 | 0.322 | 0.30 | 0.52 | 0.26 | 0.27 | 0.30 | 0.29 | 0.31 | 0.36 | 0.33 | 0.27 | 0.21 | 0.52 | 0.22 | 0.32 | 0.36 | 0.44 | 0.39 | 0.13 | 0.12 | 0.37 | 0.20 | 0.41 | 0.15 | 0.32 | 0.35 | 0.36 | 0.08 | -0.20 |
| TCGA.MF.A522.01 | -0.465 | -0.11 | 0.01 | -0.01 | 0.01 | -0.07 | -0.15 | -0.27 | 0.09 | -0.11 | -0.28 | -0.32 | 0.01 | 0.23 | -0.26 | -0.02 | 0.04 | 0.01 | 0.09 | -0.12 | -0.19 | -0.03 | -0.19 | -0.16 | -0.22 | -0.27 | -0.13 | -0.11 | -0.05 |
| TCGA.MN.A4N1.01 | -0.375 | -0.14 | 0.34 | 0.21 | -0.05 | 0.28 | 0.30 | 0.09 | 0.10 | 0.19 | 0.00 | 0.05 | 0.34 | 0.03 | 0.28 | 0.26 | 0.07 | 0.10 | -0.17 | 0.02 | 0.37 | 0.12 | 0.25 | 0.16 | 0.38 | 0.36 | 0.14 | -0.05 | -0.32 |
| TCGA.MN.A4N4.01 | -0.279 | 0.27 | 0.16 | 0.23 | 0.13 | 0.26 | 0.29 | -0.02 | 0.10 | 0.31 | -0.10 | 0.03 | 0.16 | 0.32 | 0.05 | 0.30 | 0.08 | 0.05 | -0.05 | 0.03 | 0.27 | 0.05 | 0.29 | 0.09 | 0.21 | 0.18 | 0.22 | -0.01 | -0.08 |
| TCGA.MN.A4N5.01 | -0.304 | 0.22 | 0.12 | -0.01 | 0.04 | -0.10 | -0.17 | -0.21 | -0.18 | -0.19 | -0.36 | -0.19 | 0.12 | 0.03 | -0.31 | -0.04 | 0.01 | -0.15 | -0.26 | -0.18 | -0.01 | 0.15 | -0.04 | 0.00 | -0.22 | -0.19 | -0.13 | -0.27 | 0.15 |
| TCGA.MP.A4SV.01 | 0.181 | 0.05 | 0.11 | -0.17 | -0.12 | -0.16 | -0.07 | -0.15 | 0.08 | -0.30 | -0.18 | -0.21 | 0.11 | -0.24 | -0.13 | -0.38 | -0.41 | -0.39 | -0.19 | -0.28 | -0.01 | -0.31 | -0.23 | -0.19 | -0.12 | -0.18 | -0.16 | -0.21 | 0.21 |
| TCGA.MP.A4SW.01 | 0.006 | -0.14 | 0.31 | -0.05 | 0.02 | -0.04 | -0.09 | -0.31 | -0.16 | -0.24 | -0.34 | -0.28 | 0.31 | 0.02 | -0.27 | 0.10 | 0.14 | 0.05 | -0.09 | -0.15 | 0.03 | 0.02 | -0.04 | 0.02 | 0.01 | -0.14 | -0.09 | -0.09 | 0.01 |
| TCGA.MP.A4SY.01 | -0.537 | 0.11 | 0.31 | -0.09 | 0.07 | 0.00 | -0.07 | -0.12 | -0.30 | 0.16 | 0.06 | -0.05 | 0.31 | -0.29 | -0.05 | 0.01 | -0.21 | -0.21 | -0.31 | -0.02 | 0.03 | 0.21 | -0.02 | -0.15 | 0.02 | -0.02 | -0.08 | -0.09 | -0.22 |
| TCGA.MP.A4T4.01 | -0.318 | 0.18 | 0.14 | 0.01 | 0.07 | 0.06 | 0.07 | -0.11 | 0.13 | -0.17 | -0.28 | -0.20 | 0.14 | 0.04 | -0.08 | 0.18 | 0.29 | 0.15 | -0.10 | -0.20 | 0.22 | 0.06 | 0.13 | -0.21 | -0.04 | -0.09 | 0.01 | -0.19 | -0.29 |
| TCGA.MP.A4T6.01 | -0.059 | -0.45 | 0.12 | -0.26 | -0.20 | -0.30 | -0.29 | -0.30 | 0.06 | -0.10 | -0.40 | -0.39 | 0.12 | -0.22 | -0.34 | -0.23 | -0.12 | -0.20 | -0.39 | -0.35 | -0.22 | -0.14 | -0.29 | -0.23 | -0.23 | -0.30 | -0.36 | -0.10 | 0.09 |
| TCGA.MP.A4T7.01 | 0.052 | 0.17 | -0.11 | -0.09 | 0.09 | -0.09 | -0.13 | -0.06 | 0.17 | -0.04 | -0.18 | 0.05 | -0.11 | -0.04 | -0.18 | -0.15 | -0.20 | -0.03 | -0.16 | 0.07 | -0.23 | 0.05 | -0.04 | 0.03 | -0.16 | -0.32 | -0.08 | 0.00 | 0.31 |
| TCGA.MP.A4T8.01 | -0.094 | -0.02 | 0.16 | -0.15 | -0.06 | -0.17 | -0.23 | 0.20 | 0.11 | -0.18 | 0.16 | 0.13 | 0.16 | -0.17 | -0.12 | -0.27 | -0.15 | -0.09 | -0.14 | 0.14 | -0.12 | 0.07 | -0.07 | -0.13 | -0.05 | -0.07 | -0.04 | 0.14 | 0.15 |
| TCGA.MP.A4T9.01 | -0.533 | -0.27 | 0.34 | 0.26 | -0.06 | 0.26 | 0.26 | 0.15 | 0.03 | 0.05 | 0.07 | -0.01 | 0.34 | 0.18 | 0.25 | 0.35 | 0.37 | 0.22 | -0.11 | 0.03 | 0.34 | 0.12 | 0.25 | -0.07 | 0.30 | 0.25 | 0.14 | -0.18 | -0.19 |
| TCGA.MP.A4TA.01 | -0.122 | -0.38 | 0.04 | -0.32 | -0.31 | -0.36 | -0.33 | -0.36 | -0.27 | -0.43 | -0.35 | -0.31 | 0.04 | -0.26 | -0.30 | -0.31 | -0.06 | -0.01 | -0.35 | -0.25 | -0.31 | -0.27 | -0.37 | -0.32 | -0.35 | -0.32 | -0.32 | -0.27 | -0.21 |
| TCGA.MP.A4TC.01 | 0.208 | 0.29 | -0.03 | 0.01 | 0.00 | -0.07 | -0.06 | -0.14 | -0.05 | 0.21 | -0.08 | 0.06 | -0.03 | -0.14 | 0.00 | -0.18 | -0.20 | -0.16 | -0.25 | -0.08 | -0.14 | 0.14 | 0.01 | 0.13 | -0.12 | 0.09 | -0.15 | 0.07 | 0.08 |
| TCGA.MP.A4TD.01 | -0.547 | 0.17 | 0.43 | 0.27 | 0.01 | 0.31 | 0.34 | 0.16 | 0.16 | 0.30 | 0.21 | 0.18 | 0.43 | 0.01 | 0.30 | 0.32 | 0.11 | 0.11 | -0.12 | 0.07 | 0.42 | 0.15 | 0.34 | 0.05 | 0.37 | 0.33 | 0.22 | -0.04 | -0.39 |
| TCGA.MP.A4TE.01 | 0.327 | 0.02 | -0.32 | -0.07 | -0.12 | -0.16 | -0.24 | -0.24 | -0.31 | -0.37 | -0.33 | -0.28 | -0.32 | 0.17 | -0.20 | 0.01 | 0.13 | 0.16 | 0.07 | 0.02 | -0.26 | -0.11 | -0.18 | 0.06 | -0.25 | -0.15 | -0.14 | 0.02 | 0.22 |
| TCGA.MP.A4TF.01 | -0.410 | 0.20 | -0.02 | -0.14 | -0.03 | -0.12 | -0.25 | -0.11 | -0.06 | -0.13 | -0.11 | -0.03 | -0.02 | 0.01 | -0.14 | -0.05 | -0.04 | 0.00 | -0.15 | -0.10 | -0.25 | -0.25 | -0.16 | -0.29 | -0.20 | -0.23 | -0.05 | -0.14 | -0.32 |
| TCGA.MP.A4TH.01 | -0.052 | -0.38 | 0.08 | -0.20 | -0.21 | -0.21 | -0.24 | -0.23 | -0.19 | -0.23 | -0.25 | -0.17 | 0.08 | -0.21 | -0.17 | 0.00 | -0.02 | -0.12 | -0.05 | -0.07 | -0.23 | -0.09 | -0.20 | -0.17 | -0.26 | -0.32 | -0.18 | -0.15 | -0.20 |
| TCGA.MP.A4TI.01 | 0.523 | 0.06 | -0.25 | -0.13 | -0.02 | -0.14 | -0.14 | 0.08 | -0.03 | 0.18 | 0.18 | 0.19 | -0.25 | -0.05 | 0.12 | -0.29 | -0.24 | -0.25 | 0.01 | 0.02 | -0.24 | -0.01 | -0.05 | -0.01 | -0.23 | -0.01 | -0.04 | 0.15 | 0.25 |
| TCGA.MP.A4TK.01 | 0.288 | 0.20 | -0.05 | -0.12 | 0.16 | -0.14 | -0.22 | -0.21 | 0.11 | -0.14 | -0.08 | -0.23 | -0.05 | 0.21 | -0.24 | -0.37 | -0.29 | -0.22 | 0.14 | -0.13 | -0.31 | 0.07 | -0.19 | -0.07 | -0.22 | -0.21 | -0.25 | 0.13 | 0.39 |
| TCGA.MP.A5C7.01 | 0.247 | 0.50 | 0.22 | 0.29 | 0.26 | 0.36 | 0.40 | 0.30 | 0.24 | 0.50 | 0.29 | 0.28 | 0.22 | 0.15 | 0.41 | 0.21 | 0.01 | -0.05 | 0.02 | 0.25 | 0.41 | 0.44 | 0.40 | 0.35 | 0.38 | 0.47 | 0.25 | 0.27 | -0.05 |
| TCGA.NC.A5HD.01 | -0.211 | -0.31 | -0.04 | -0.02 | 0.36 | -0.09 | -0.08 | -0.10 | -0.05 | -0.06 | -0.21 | 0.03 | -0.04 | 0.25 | 0.10 | -0.37 | -0.12 | -0.02 | 0.05 | 0.00 | -0.25 | 0.00 | -0.05 | 0.02 | -0.07 | -0.16 | -0.17 | 0.14 | 0.22 |
| TCGA.NC.A5HE.01 | -0.098 | 0.01 | 0.44 | -0.14 | -0.27 | 0.07 | 0.05 | -0.17 | -0.19 | 0.03 | 0.02 | -0.27 | 0.44 | -0.24 | 0.00 | 0.27 | 0.28 | 0.03 | -0.12 | 0.02 | 0.15 | 0.03 | 0.10 | -0.17 | -0.04 | 0.01 | 0.01 | -0.09 | -0.19 |
| TCGA.NC.A5HF.01 | -0.515 | -0.12 | -0.12 | -0.11 | 0.05 | -0.16 | -0.20 | -0.06 | 0.13 | -0.23 | -0.11 | 0.00 | -0.12 | 0.16 | -0.16 | -0.32 | -0.20 | -0.15 | 0.02 | -0.10 | -0.09 | -0.11 | -0.02 | -0.15 | -0.08 | -0.20 | -0.15 | 0.02 | 0.11 |
| TCGA.NC.A5HG.01 | 0.396 | -0.37 | -0.01 | -0.18 | -0.25 | -0.14 | -0.18 | -0.09 | -0.10 | -0.42 | -0.19 | -0.12 | -0.01 | -0.15 | -0.26 | -0.14 | 0.13 | 0.04 | 0.02 | -0.20 | -0.26 | -0.24 | -0.20 | -0.20 | -0.34 | -0.21 | -0.18 | -0.22 | 0.11 |
| TCGA.NC.A5HH.01 | -0.044 | 0.59 | -0.13 | 0.27 | 0.32 | 0.17 | 0.20 | 0.35 | 0.29 | 0.18 | 0.36 | 0.39 | -0.13 | 0.37 | 0.28 | 0.10 | -0.04 | 0.15 | 0.29 | 0.16 | 0.25 | 0.02 | 0.21 | 0.23 | 0.23 | 0.22 | 0.29 | 0.06 | -0.07 |
| TCGA.NC.A5HI.01 | -0.265 | -0.39 | 0.20 | -0.22 | -0.24 | -0.14 | -0.17 | -0.41 | -0.34 | -0.32 | -0.44 | -0.42 | 0.20 | -0.03 | -0.26 | -0.13 | 0.03 | -0.15 | -0.26 | -0.31 | -0.04 | -0.19 | -0.14 | -0.16 | -0.23 | -0.23 | -0.27 | -0.26 | -0.23 |
| TCGA.NC.A5HJ.01 | 0.105 | -0.39 | -0.12 | -0.14 | -0.31 | -0.08 | -0.13 | -0.10 | -0.23 | -0.24 | -0.29 | -0.26 | -0.12 | -0.34 | -0.30 | -0.06 | -0.12 | -0.28 | -0.27 | -0.36 | 0.03 | -0.43 | -0.25 | -0.26 | 0.02 | -0.20 | -0.13 | -0.26 | -0.16 |
| TCGA.NC.A5HK.01 | 0.060 | -0.08 | -0.28 | -0.08 | -0.17 | -0.19 | -0.21 | -0.25 | -0.03 | -0.26 | -0.23 | -0.14 | -0.28 | -0.12 | -0.27 | -0.17 | -0.01 | 0.05 | -0.32 | -0.16 | -0.24 | -0.02 | -0.28 | -0.23 | -0.25 | -0.30 | -0.14 | -0.20 | 0.19 |
| TCGA.NC.A5HL.01 | 0.007 | 0.15 | -0.04 | 0.01 | 0.13 | 0.05 | -0.08 | 0.07 | -0.31 | -0.06 | 0.05 | 0.17 | -0.04 | 0.20 | 0.17 | -0.08 | 0.06 | 0.00 | 0.18 | 0.24 | -0.12 | -0.03 | 0.06 | 0.23 | -0.10 | -0.01 | 0.09 | 0.13 | 0.12 |
| TCGA.NC.A5HM.01 | -0.060 | -0.44 | -0.29 | -0.24 | -0.12 | -0.30 | -0.24 | -0.18 | 0.05 | -0.38 | -0.24 | -0.17 | -0.29 | -0.15 | -0.38 | -0.18 | -0.09 | 0.12 | 0.05 | -0.17 | -0.38 | -0.41 | -0.26 | -0.27 | -0.22 | -0.43 | -0.21 | -0.14 | 0.05 |
| TCGA.NC.A5HN.01 | -0.151 | -0.26 | 0.07 | -0.24 | 0.04 | -0.18 | -0.28 | -0.28 | 0.09 | -0.29 | -0.41 | -0.31 | 0.07 | -0.08 | -0.31 | -0.39 | -0.38 | -0.26 | -0.10 | -0.26 | -0.26 | -0.16 | -0.17 | -0.28 | -0.21 | -0.31 | -0.28 | -0.08 | -0.07 |
| TCGA.NC.A5HO.01 | 0.226 | -0.16 | -0.32 | -0.16 | -0.20 | -0.14 | -0.19 | -0.13 | 0.10 | -0.04 | 0.01 | -0.16 | -0.32 | -0.29 | -0.10 | -0.21 | -0.18 | -0.16 | -0.01 | -0.08 | -0.13 | -0.15 | -0.14 | -0.17 | -0.13 | 0.07 | -0.15 | 0.06 | 0.16 |
| TCGA.NC.A5HP.01 | -0.189 | -0.43 | 0.16 | -0.11 | 0.00 | -0.11 | -0.19 | -0.02 | -0.29 | -0.11 | 0.03 | 0.09 | 0.16 | -0.05 | -0.06 | -0.02 | 0.09 | 0.14 | 0.14 | -0.08 | -0.06 | -0.18 | -0.07 | 0.03 | 0.03 | -0.19 | 0.03 | 0.11 | -0.09 |
| TCGA.NC.A5HQ.01 | -0.142 | -0.26 | -0.16 | -0.05 | -0.08 | -0.05 | -0.09 | 0.00 | 0.24 | 0.09 | 0.11 | -0.02 | -0.16 | -0.11 | 0.00 | -0.04 | 0.23 | 0.02 | -0.21 | -0.17 | 0.00 | -0.04 | 0.09 | -0.13 | 0.05 | 0.00 | 0.00 | 0.11 | 0.04 |
| TCGA.NC.A5HR.01 | -0.139 | 0.20 | -0.34 | -0.17 | -0.17 | -0.19 | -0.22 | 0.04 | 0.05 | -0.06 | 0.12 | 0.14 | -0.34 | -0.15 | -0.01 | -0.40 | -0.13 | -0.20 | -0.16 | 0.00 | -0.10 | 0.08 | -0.11 | -0.19 | -0.24 | 0.04 | -0.11 | 0.09 | 0.14 |
| TCGA.NC.A5HT.01 | -0.124 | -0.01 | -0.15 | -0.14 | -0.20 | -0.22 | -0.27 | -0.21 | -0.15 | -0.26 | -0.31 | -0.12 | -0.15 | -0.09 | -0.18 | -0.07 | 0.16 | 0.25 | 0.16 | -0.16 | -0.31 | -0.12 | -0.30 | -0.21 | -0.36 | -0.21 | -0.14 | 0.03 | 0.19 |
| TCGA.NJ.A4YF.01 | 0.081 | -0.21 | 0.41 | 0.17 | -0.17 | 0.24 | 0.15 | -0.13 | -0.30 | -0.10 | -0.09 | -0.21 | 0.41 | -0.03 | 0.18 | 0.34 | 0.30 | 0.22 | 0.02 | -0.05 | 0.35 | -0.05 | 0.13 | 0.18 | 0.27 | 0.28 | 0.05 | -0.11 | -0.07 |
| TCGA.NJ.A4YG.01 | -0.130 | -0.43 | 0.08 | 0.07 | -0.30 | 0.03 | 0.10 | -0.07 | -0.21 | -0.03 | -0.34 | -0.20 | 0.08 | -0.15 | -0.09 | -0.02 | 0.15 | -0.14 | -0.20 | -0.18 | 0.28 | -0.23 | 0.01 | -0.18 | 0.21 | 0.12 | -0.03 | -0.27 | -0.37 |
| TCGA.NJ.A4YP.01 | 0.111 | 0.13 | -0.09 | -0.13 | -0.10 | -0.11 | -0.19 | -0.17 | 0.09 | -0.13 | -0.16 | -0.14 | -0.09 | -0.02 | -0.21 | -0.12 | -0.06 | 0.06 | -0.22 | -0.21 | -0.28 | -0.01 | -0.10 | -0.08 | -0.28 | -0.13 | -0.16 | -0.03 | 0.25 |
| TCGA.NJ.A4YQ.01 | 0.432 | -0.09 | 0.18 | 0.06 | 0.05 | 0.10 | 0.20 | -0.08 | -0.16 | -0.01 | 0.12 | 0.03 | 0.18 | -0.24 | -0.12 | 0.12 | 0.09 | 0.17 | 0.11 | 0.08 | 0.16 | 0.09 | 0.13 | 0.10 | 0.13 | 0.15 | 0.12 | 0.04 | -0.15 |
| TCGA.NJ.A55R.01 | -0.019 | 0.09 | -0.31 | 0.09 | 0.21 | 0.06 | 0.01 | 0.17 | -0.09 | -0.13 | 0.16 | 0.20 | -0.31 | 0.26 | 0.06 | 0.15 | 0.17 | 0.32 | 0.10 | 0.15 | -0.16 | -0.06 | -0.04 | 0.10 | -0.05 | -0.09 | 0.06 | -0.15 | 0.15 |
| TCGA.NJ.A7XG.01 | -0.049 | 0.02 | 0.24 | -0.06 | -0.17 | 0.04 | -0.03 | -0.02 | -0.20 | 0.20 | -0.22 | -0.15 | 0.24 | -0.17 | -0.02 | 0.09 | -0.09 | -0.15 | -0.27 | -0.24 | 0.18 | 0.00 | -0.07 | -0.02 | 0.10 | 0.03 | -0.08 | -0.16 | -0.15 |
| TCGA.NK.A5CR.01 | 0.370 | 0.10 | 0.16 | -0.01 | 0.16 | -0.11 | -0.10 | 0.13 | -0.05 | 0.27 | 0.17 | 0.07 | 0.16 | 0.07 | -0.13 | -0.30 | -0.26 | -0.15 | 0.16 | 0.04 | -0.19 | 0.08 | 0.03 | 0.02 | -0.07 | -0.24 | -0.09 | 0.01 | 0.16 |
| TCGA.NK.A5CT.01 | -0.270 | -0.18 | 0.31 | 0.12 | -0.14 | 0.11 | 0.18 | 0.19 | -0.06 | 0.16 | 0.09 | 0.07 | 0.31 | -0.09 | 0.13 | 0.23 | 0.12 | -0.07 | -0.02 | -0.05 | 0.33 | -0.21 | 0.18 | -0.30 | 0.20 | 0.14 | 0.24 | -0.21 | -0.40 |
| TCGA.NK.A5CX.01 | -0.024 | 0.00 | -0.48 | -0.13 | 0.15 | -0.09 | -0.11 | -0.07 | 0.31 | 0.05 | -0.01 | -0.07 | -0.48 | 0.04 | 0.14 | -0.15 | -0.24 | -0.38 | -0.13 | 0.07 | -0.24 | -0.05 | -0.01 | -0.20 | -0.17 | 0.01 | -0.12 | 0.07 | 0.14 |
| TCGA.NK.A5D1.01 | 0.424 | -0.05 | 0.38 | -0.05 | -0.22 | 0.04 | 0.05 | -0.23 | 0.06 | -0.11 | -0.26 | -0.34 | 0.38 | -0.05 | -0.01 | 0.40 | 0.45 | 0.23 | 0.09 | -0.02 | 0.14 | -0.03 | 0.09 | -0.02 | -0.10 | -0.01 | 0.01 | -0.30 | -0.28 |
| TCGA.NK.A7XE.01 | 0.431 | 0.08 | -0.45 | -0.08 | -0.05 | -0.11 | -0.08 | 0.11 | 0.08 | 0.25 | 0.19 | 0.21 | -0.45 | -0.18 | -0.11 | -0.16 | 0.04 | -0.14 | 0.04 | 0.05 | -0.24 | -0.16 | -0.26 | -0.04 | -0.02 | -0.03 | -0.06 | 0.14 | 0.03 |
| TCGA.O1.A52J.01 | 0.065 | -0.38 | 0.35 | -0.17 | -0.14 | -0.13 | -0.17 | -0.12 | -0.19 | -0.08 | -0.32 | -0.10 | 0.35 | -0.13 | -0.10 | 0.12 | 0.22 | 0.22 | 0.07 | 0.00 | -0.12 | 0.19 | -0.06 | 0.02 | -0.12 | -0.16 | -0.11 | -0.04 | 0.17 |
| TCGA.O2.A52N.01 | -0.368 | -0.40 | -0.19 | -0.16 | -0.42 | -0.13 | -0.21 | -0.29 | -0.47 | -0.45 | -0.19 | -0.33 | -0.19 | -0.20 | -0.11 | -0.07 | -0.02 | -0.10 | -0.17 | -0.28 | -0.02 | -0.41 | -0.28 | -0.25 | -0.15 | -0.21 | -0.10 | -0.43 | -0.20 |
| TCGA.O2.A52Q.01 | 0.076 | 0.02 | -0.37 | -0.09 | 0.05 | -0.20 | -0.28 | 0.01 | -0.25 | 0.06 | -0.05 | 0.02 | -0.37 | -0.05 | -0.20 | -0.31 | -0.12 | -0.15 | -0.04 | 0.09 | -0.31 | -0.05 | -0.29 | -0.14 | -0.25 | -0.19 | -0.14 | 0.01 | 0.01 |
| TCGA.O2.A52S.01 | 0.371 | 0.40 | -0.42 | -0.10 | -0.01 | -0.06 | -0.02 | 0.20 | 0.15 | 0.29 | 0.23 | 0.15 | -0.42 | -0.04 | 0.02 | 0.18 | 0.24 | -0.02 | -0.01 | 0.07 | -0.14 | 0.01 | 0.03 | -0.03 | -0.12 | -0.15 | 0.08 | 0.02 | -0.15 |
| TCGA.O2.A52V.01 | 0.425 | 0.30 | -0.28 | 0.02 | 0.14 | -0.04 | -0.05 | -0.04 | 0.09 | -0.22 | 0.04 | 0.06 | -0.28 | 0.18 | 0.06 | 0.00 | 0.12 | 0.08 | 0.27 | 0.03 | -0.24 | -0.07 | -0.09 | 0.16 | -0.09 | -0.09 | -0.02 | 0.13 | 0.35 |
| TCGA.O2.A52W.01 | 0.136 | 0.22 | 0.13 | 0.05 | -0.06 | 0.19 | 0.05 | -0.07 | -0.16 | -0.04 | 0.12 | 0.04 | 0.13 | -0.11 | 0.01 | -0.14 | -0.07 | -0.09 | -0.29 | 0.13 | 0.15 | 0.15 | -0.01 | -0.03 | 0.21 | 0.25 | 0.05 | -0.06 | -0.14 |
| TCGA.O2.A5IB.01 | -0.149 | -0.25 | -0.50 | -0.19 | 0.04 | -0.29 | -0.29 | -0.12 | 0.23 | 0.14 | -0.04 | -0.10 | -0.50 | -0.30 | -0.20 | -0.19 | -0.43 | -0.42 | -0.11 | -0.05 | -0.36 | 0.01 | -0.23 | -0.29 | -0.25 | -0.18 | -0.18 | 0.04 | 0.19 |
| TCGA.S2.AA1A.01 | 0.273 | -0.21 | 0.30 | 0.02 | -0.28 | 0.15 | 0.04 | -0.09 | -0.11 | 0.03 | -0.06 | -0.22 | 0.30 | -0.16 | 0.04 | 0.20 | 0.39 | 0.17 | -0.29 | -0.18 | 0.24 | 0.00 | 0.14 | -0.24 | 0.20 | 0.13 | 0.08 | -0.29 | -0.36 |
| TCGA.XC.AA0X.01 | -0.418 | 0.07 | 0.34 | 0.23 | 0.25 | 0.26 | 0.21 | 0.18 | 0.14 | 0.21 | 0.17 | 0.20 | 0.34 | 0.16 | 0.23 | 0.34 | 0.23 | 0.18 | 0.25 | 0.13 | 0.30 | 0.19 | 0.24 | 0.02 | 0.22 | 0.09 | 0.23 | 0.04 | -0.24 |
