# Supplementary material for: ST6GAL1 promotes cancer stem-like cell-associated paclitaxel resistance through the EGFR-mTOR-SOX2/BMI1 axis in non-small cell lung cancer
Source: Cancer Drug Resist. 2026 Aug 31;9:32. doi: 10.20517/cdr.2026.66 (PMC13589886; doi:10.20517/cdr.2026.66)
Supplement: Supplementary file 3 [file cdr-9-32-Supplementaryfiles.zip › Supplementary File 5-clinical cancer tissue information.docx]

| **ST6GAL1 staining in lung cancer tissues and clincial cancer tissues information** | | | | | | | | |
| --- | --- | --- | --- | --- | --- | --- | --- | --- |
| **No.** | **Tissue Type** | **ST6GAL1 intensity** | **Group** | **Survival (M)** | **Therapeutics** | **No.** | **Tissue Type** | **ST6GAL1 intensity** |
| A1 | Cancer | 0.0657 | high | 57 | N.A | A2 | adjacent non-tumor tissue | 0.0179 |
| A3 | Cancer | 0.0433 | low | 28 | N.A | A4 | adjacent non-tumor tissue | 0.0208 |
| A5 | Cancer | 0.0261 | low | 24 | N.A | A6 | adjacent non-tumor tissue | 0.0251 |
| A7 | Cancer | 0.0314 | low | 125+ | N.A | A8 | adjacent non-tumor tissue | 0.0188 |
| A11 | Cancer | 0.0424 | low | 29 | N.A | A12 | adjacent non-tumor tissue | 0.0351 |
| B1 | Cancer | 0.058 | high | 35 | Pemetrexed + cisplatin | B2 | adjacent non-tumor tissue | 0.0138 |
| B3 | Cancer | 0.07 | high | 82 | Pemetrexed + nedaplatin | B4 | adjacent non-tumor tissue | 0.0138 |
| B5 | Cancer | 0.0482 | high | 75 | N.A | B6 | adjacent non-tumor tissue | 0.0168 |
| B7 | Cancer | 0.0823 | high | 65 | N.A | B8 | adjacent non-tumor tissue | 0.0885 |
| B11 | Cancer | 0.0274 | low | 91 | N.A | B12 | adjacent non-tumor tissue | 0.0174 |
| C1 | Cancer | 0.0571 | high | 36 | N.A | C2 | adjacent non-tumor tissue | 0.0291 |
| C3 | Cancer | 0.0499 | high | 36 | N.A | C4 | adjacent non-tumor tissue | 0.0396 |
| C5 | Cancer | 0.043 | low | 13 | N.A | C6 | adjacent non-tumor tissue | 0.0195 |
| C7 | Cancer | 0.081 | high | 83 | Pemetrexed + nedaplatin | C8 | adjacent non-tumor tissue | 0.0309 |
| C9 | Cancer | 0.045 | low | 131 | N.A | C10 | adjacent non-tumor tissue | 0.0149 |
| C11 | Cancer | 0.011 | low | 152+ | N.A | C12 | adjacent non-tumor tissue | 0.011 |
| D1 | Cancer | 0.0424 | low | 42 | N.A | D2 | adjacent non-tumor tissue | 0.0351 |
| D3 | Cancer | 0.0565 | high | 37 | Gemcitabine + vinorelbine | D4 | adjacent non-tumor tissue | 0.0184 |
| D9 | Cancer | 0.0731 | high | 17 | Gemcitabine + cisplatin | D10 | adjacent non-tumor tissue | 0.0186 |
| D11 | Cancer | 0.0798 | high | 34 | Gemcitabine + vinorelbine | D12 | adjacent non-tumor tissue | 0.0216 |
| E1 | Cancer | 0.0761 | high | 146+ | Gemcitabine + oxaliplatin | E2 | adjacent non-tumor tissue | 0.0578 |
| E3 | Cancer | 0.0761 | high | 20 | N.A | E4 | adjacent non-tumor tissue | 0.0329 |
| E7 | Cancer | 0.038 | low | 35 | Vinorelbine + gemcitabine | E8 | adjacent non-tumor tissue | 0.0231 |
| E9 | Cancer | 0.0717 | high | 11 | Docetaxel + carboplatin | E10 | adjacent non-tumor tissue | 0.0218 |
| E11 | Cancer | 0.0358 | low | 34 | N.A | E12 | adjacent non-tumor tissue | 0.016 |
| F1 | Cancer | 0.0331 | low | 3 | N.A | F2 | adjacent non-tumor tissue | 0.0349 |
| F3 | Cancer | 0.0651 | high | 79 | N.A | F4 | adjacent non-tumor tissue | 0.022 |
| F5 | Cancer | 0.0492 | high | 8 | N.A | F6 | adjacent non-tumor tissue | 0.0426 |
| F7 | Cancer | 0.0399 | low | 119 | Vinorelbine + gemcitabine | F8 | adjacent non-tumor tissue | 0.0499 |
| F9 | Cancer | 0.0401 | low | 119 | N.A | F10 | adjacent non-tumor tissue | 0.0083 |
| F11 | Cancer | 0.039 | low | 129 | N.A | F12 | adjacent non-tumor tissue | 0.0103 |
| G1 | Cancer | 0.0424 | low | 140+ | Docetaxel + carboplatin | G2 | adjacent non-tumor tissue | 0.0107 |
| G3 | Cancer | 0.0524 | high | 15 | N.A | G4 | adjacent non-tumor tissue | 0.0318 |
| G5 | Cancer | 0.0251 | low | 24 | N.A | G6 | adjacent non-tumor tissue | 0.0129 |
| G7 | Cancer | 0.0296 | low | 80 | N.A | G8 | adjacent non-tumor tissue | 0.0245 |
| G9 | Cancer | 0.0567 | high | 101 | N.A | G10 | adjacent non-tumor tissue | 0.0163 |
| G11 | Cancer | 0.059 | high | 100 | N.A | G12 | adjacent non-tumor tissue | 0.0093 |
| H1 | Cancer | 0.0272 | low | 38 | Carboplatin + pemetrexed | H2 | adjacent non-tumor tissue | 0.0422 |
| H3 | Cancer | 0.0336 | low | 81 | Pemetrexed | H4 | adjacent non-tumor tissue | 0.0156 |
| H5 | Cancer | 0.0511 | high | 25 | N.A | H6 | adjacent non-tumor tissue | 0.0238 |
| H7 | Cancer | 0.0467 | high | 115 | N.A | H8 | adjacent non-tumor tissue | 0.0161 |
| H9 | Cancer | 0.0812 | high | 76 | N.A | H10 | adjacent non-tumor tissue | 0.0105 |
| H11 | Cancer | 0.0854 | high | 116 | N.A | H12 | adjacent non-tumor tissue | 0.0083 |
| I1 | Cancer | 0.0355 | low | 74 | N.A | I2 | adjacent non-tumor tissue | 0.0163 |
| I5 | Cancer | 0.039 | low | 102 | Vinorelbine + gemcitabine | I6 | adjacent non-tumor tissue | 0.0177 |
| I7 | Cancer | 0.0318 | low | 23 | N.A | I8 | adjacent non-tumor tissue | 0.0325 |
| M, month; +, over | | | |  |  |  |  |  |
