# Supplementary material for: ST6GAL1 promotes cancer stem-like cell-associated paclitaxel resistance through the EGFR-mTOR-SOX2/BMI1 axis in non-small cell lung cancer
Source: Cancer Drug Resist. 2026 Aug 31;9:32. doi: 10.20517/cdr.2026.66 (PMC13589886; doi:10.20517/cdr.2026.66)
Supplement: Supplementary file 3 [file cdr-9-32-Supplementaryfiles.zip › Supplementary File 1-Table S1.docx]

**Table S1**: The sequence of qPCR primers and shRNA target

| **Gene** | **primer Sequence** |
| --- | --- |
| *GAPDH* | F: 5'- AGCCACATCGCTCAGACAC -3' |
|  | R: 5'- GAGGCATTGCTGATGATCTTG -3' |
| *BMI1* | F: 5'- AGATACTTACGATGCCCAGCAG -3' |
|  | R: 5'- CAGTCCATCTCTCTGGTGACTG -3' |
| *MYC* | F: 5'- TCTGAGGAGGAACAAGAAGATG -3' |
|  | R: 5'- GCGTAGTTGTGCTGATGTGTG -3' |
| *NANOG* | F: 5'- ATAGCAATGGTGTGACGCAG -3' |
|  | R: 5'- CCAGGTTGAATTGTTCCAGGTC -3' |
| *KLF4* | F: 5'- CATGCCAGAGGAGCCCAAG -3' |
|  | R: 5'- ATTTCCATCCACAGCCGTCC -3' |
| *SOX2* | F: 5'- GGGAAATGGGAGGGGTGC -3' |
|  | R: 5'- TGCGTGAGTGTGGATGGG -3' |
| *ALG1* | F: 5'- GTGTGTGCTGTGAACTTCAAGT -3' |
|  | R: 5'- TTTGCCCTGAGACTCCAAGAAG -3' |
| *ALG3* | F: 5'- CCCCTTACACCCAACCAGAT -3' |
|  | R: 5'- GTTCCAGGAGAGCTCGATGA -3' |
| *ALG12* | F: 5'- GGTGTGTCTCGGTTTCTCC -3' |
|  | R: 5'- GCTTTGTCTGCAGGTGGAC -3' |
| *B4GALT2* | F: 5'- GGTGGCGAGGATGATGACA -3' |
|  | R: 5'- AAGAGTGGTTGCCGAGACA -3' |
| *DDOST* | F: 5'- GGTGGCAAATACAGTGTTCAGT -3' |
|  | R: 5'- GCAAAACAAAACCACCAATCCT -3' |
| *DOLK* | F: 5'- GTGGGTGATACTGTGGCCT -3' |
|  | R: 5'- GGAGGGACACAGTGCTGAT -3' |
| *GANAB* | F: 5'- AGCGGGTGGTGATAATAGGG -3' |
|  | R: 5'- TCTCAGGGTCATGCTGGAAG -3' |
| *HEXA* | F: 5'- TGATTGGTGGAGAGGCTTGT -3' |
|  | R: 5'- GGGCTCAGGTCTGTTCAAAC -3' |
| *MAN1B1* | F: 5'- TTCTACCTGTACCGCGTCAC -3' |
|  | R: 5'- CTAGGCAGGGGTCCAGATAG -3' |
| *MAN1C1* | F: 5'- CTACTACATCCTCCGGCCAG -3' |
|  | R: 5'- TCCAGGGAGAGCAAGTCATC -3' |
| *MGAT4A* | F: 5'- TGTTGCAGAAGGAATGGTGG -3' |
|  | R: 5'- TCAGTTCCCGTGGCTGTAG -3' |
| *MAGT5* | F: 5'- ACCTCAGAGCAGCTTCACAT -3' |
|  | R: 5'- GGACAGTACATTTGGACGGC -3' |
| *MOGS* | F: 5'- GTGAACTTGGTGACTTCCGC -3' |
|  | R: 5'- GGGTCACCTGCTGTATCAAG -3' |
| *ST6GAL1* | F: 5'- GTAAGCTGCACCCCAATCAG -3' |
|  | R: 5'- GGTCACACAGCGTCATCATG -3' |
| *DOLPP1* | F: 5'- TACCTGCTGTACCACACCTG -3' |
|  | R: 5'- CTGGTCACTGCAGTTTCGTC -3' |
| *shST6GAL1-1* | F: 5'- CGCTGCTCTATGAGAAGAATT -3' |
| *shST6GAL1-2* | F: 5'- CCTAAGCATGAACAAGTACAA -3' |

Note: F, forward primer; R, reverse primer
